# Genetic association with overall survival of taxane-treated lung cancer patients - a genome-wide association study in human lymphoblastoid cell lines followed by a clinical association study

**DOI:** 10.1186/1471-2407-12-422

**Published:** 2012-09-24

**Authors:** Nifang Niu, Daniel J Schaid, Ryan P Abo, Krishna Kalari, Brooke L Fridley, Qiping Feng, Gregory Jenkins, Anthony Batzler, Abra G Brisbin, Julie M Cunningham, Liang Li, Zhifu Sun, Ping Yang, Liewei Wang

**Affiliations:** 1 Division of Clinical Pharmacology, Department of Molecular Pharmacology and Experimental Therapeutics, Mayo Clinic, 200 First Street SW, Rochester, MN 55905, USA; 2 Division of Biostatistics and Informatics, Department of Health Sciences Research, Mayo Clinic, Rochester, MN, USA; 3 Department of Biology, Massachusetts Institute of Technology, Cambridge, MA, USA; 4 Division of Clinical Pharmacology, Department of Medicine, Vanderbilt University Medical Center, Nashville, TN, USA; 5 Department of Laboratory Medicine and Pathology, Mayo Clinic, Rochester, MN, USA; 6 Division of Epidemiology, Department of Health Sciences Research, Mayo Clinic, Rochester, MN, USA

**Keywords:** Taxane, Genome-wide association, Lymphoblastoid cell line, Lung cancer, Overall survival

## Abstract

**Background:**

Taxane is one of the first line treatments of lung cancer. In order to identify novel single nucleotide polymorphisms (SNPs) that might contribute to taxane response, we performed a genome-wide association study (GWAS) for two taxanes, paclitaxel and docetaxel, using 276 lymphoblastoid cell lines (LCLs), followed by genotyping of top candidate SNPs in 874 lung cancer patient samples treated with paclitaxel.

**Methods:**

GWAS was performed using 1.3 million SNPs and taxane cytotoxicity IC50 values for 276 LCLs. The association of selected SNPs with overall survival in 76 small or 798 non-small cell lung cancer (SCLC, NSCLC) patients were analyzed by Cox regression model, followed by integrated SNP-microRNA-expression association analysis in LCLs and siRNA screening of candidate genes in SCLC (H196) and NSCLC (A549) cell lines.

**Results:**

147 and 180 SNPs were associated with paclitaxel or docetaxel IC50s with p-values <10^-4^ in the LCLs, respectively. Genotyping of 153 candidate SNPs in 874 lung cancer patient samples identified 8 SNPs (p-value < 0.05) associated with either SCLC or NSCLC patient overall survival. Knockdown of *PIP4K2A*, *CCT5*, *CMBL, EXO1*, *KMO* and *OPN3*, genes within 200 kb up-/downstream of the 3 SNPs that were associated with SCLC overall survival (rs1778335, rs2662411 and rs7519667), significantly desensitized H196 to paclitaxel. SNPs rs2662411 and rs1778335 were associated with mRNA expression of *CMBL* or *PIP4K2A* through microRNA (miRNA) hsa-miR-584 or hsa-miR-1468.

**Conclusions:**

GWAS in an LCL model system, joined with clinical translational and functional studies, might help us identify genetic variations associated with overall survival of lung cancer patients treated paclitaxel.

## Background

Taxanes are an important class of chemotherapeutic agents that disrupt the dynamics of microtubules by enhancing tubulin assembly and inhibiting depolymerisation [1]. Two taxanes, paclitaxel (Taxol®) and docetaxel (Taxotere®), are widely used for a broad spectrum of cancers, including lung cancer, one of the most common cancer types and the leading cause of cancer mortality in the US in 2012 [2,3]. However, as a first-line therapy for non-small cell lung cancer (NSCLC) and a second-line therapy for small cell lung cancer (SCLC) [4,5], large inter-individual variations have been observed in response to taxane therapy, in both efficacy and toxicity. One major side effect of taxanes, especially paclitaxel, is peripheral neuropathy, which limits dose escalation for optimal treatment with taxanes in the clinic [6]. Response rates for a single treatment with paclitaxel in patients with advanced NSCLC or extensive stage of SCLC are 24% and 34%, respectively [7]. Overall response rates for taxane-platinum combination treatment were 17-32%, and the incidence of grade 3/4 peripheral neuropathy was 1-13% in advanced NSCLC [8].

A great deal of effort has been devoted to the identification of biomarkers for response to these agents. Genetic polymorphisms in *CYP3A4*, *ABCB1*, *ERCC1*, *ERCC2*, and *XPD1* were found to be associated with inter-individual differences in taxane response in NSCLC patients [9-11], while other variants in *CYP2C8*, *CYP3A5* and *ABCB1* were related to variability in taxane-mediated neurotoxicity [12,13]. These observations may relate to the effect of genetic polymorphisms on the alteration of either taxane pharmacokinetic or pharmacodynamic profiles through influence on gene expression or enzyme activities [14,15]. In addition, a genome-wide linkage study using 427 lymphoblastoid cell lines (LCLs) from 38 Centre d’Etude du Polymorphisme Humain (CEPH) reference pedigrees identified two loci, 5q11-21 and 9q13-22, associated with docetaxel-induced cytotoxicity [16]. Another study using breast cancer cell lines showed that increasing ABCC3 expression was highly associated with paclitaxel resistance [17]. Recently, a clinical GWAS with 1040 patients treated with paclitaxel identified 3 SNPs located in the *EPHA5*, *FGD4* and *NDRG1* genes that were associated with peripheral neuropathy [18]. All of these results suggest that genetic variation plays an important role in inter-individual variation in taxane response.

**Figure 1:**
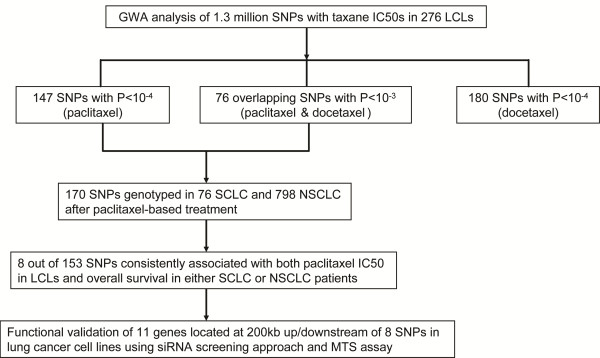
**Schematic diagram of the experimental strategy.** Genome-wide association studies were performed for paclitaxel or docetaxel IC50 using 1.3 million SNPs. 147 SNPs were associated with paclitaxel IC50 with p-values < 10^-4^ and 76 SNPs were associated with IC50 values for both taxanes with p-values < 10^-3^. Those SNPs were genotyped in 874 lung cancer patients treated with paclitaxel-based chemotherapy. 8 SNPs were found to be consistently associated with both paclitaxel IC50 in LCLs and lung cancer overall survival. Eleven genes which were close to those 8 SNPs were functionally validated using lung cancer cell lines.

In the present study, we tested the hypothesis that genetic variation may contribute to inter-individual variation in overall survival of lung cancer patients treated with paclitaxel-based therapy. As a first step to identify additional novel quantitative trait loci (QTL) contributing to taxane response, we performed pharmacogenomic studies with both paclitaxel and docetaxel using a genome-wide association (GWA) approach with 276 LCLs, a cell line model system that has been used successfully in many previous pharmacogenomic studies to identify genetic variation related to drug or radiation response phenotypes [19-21]. We then genotyped 874 Caucasian lung cancer patients (76 SCLC and 798 NSCLC) for the 170 most significant candidate SNPs identified during the association studies with the 276 LCLs. Eight SNPs were found to be consistently associated with both paclitaxel IC50 in LCLs and overall survival in SCLC or NSCLC patients. Finally, 11 candidate genes, located within 200 kb up-/downstream of those 8 SNPs, were subjected to functional validation in lung cancer cell lines by using siRNA screening and MTS assays (Figure 1). In addition, we also performed SNP-expression association analysis and integrated SNP-miRNA-expression association analysis using those 8 SNPs, expression of 11 candidate genes and 226 miRNAs from LCLs.

## Methods

### Cell lines

As described in our previous publication [21], EBV-transformed LCLs from 96 African-American (AA), 96 Caucasian-American (CA), and 96 Han Chinese-American (HCA) unrelated subjects (sample sets HD100AA, HD100CAU, HD100CHI) were purchased from the Coriell Cell Repository (Camden, NJ). These samples had been anonymized by NIGMS, and all subjects had provided written consent for their experimental use. This study was reviewed and approved by Mayo Clinic Institutional Review Board. Human SCLC cell line H196 and NSCLC cell line A549 were obtained from the American Type Culture Collection (Manassas, VA). LCLs were cultured in RPMI 1640 medium (Mediatech, Manassas, VA) supplemented with 15% heat-inactivated Fetal Bovine Serum (FBS) (Mediatech). H196 and A549 cell lines were cultured in RPMI 1640 medium containing 10% FBS.

### Lung cancer patient samples

A total of 874 lung cancer patients treated with taxane-based therapy, including 76 SCLC and 798 NSCLC, were identified and enrolled between 1997 and 2008 at the Mayo Clinic (Rochester, MN). Details regarding clinical characteristics of these patients, patient enrollment, and data collection procedures were described previously [22,23]. Briefly, each case was identified through the Mayo Clinic pathologic diagnostic (Co-Path) system. After obtaining written informed consent, blood samples were collected from patients. The characteristics of patients were abstracted from the medical record, including demographics, lung cancer pathology, anatomic site, and types and timing of treatment and chemotherapeutic agents. The clinical staging and recurrence or progression data were determined by results from available chest radiography, computerized tomography, bone scans, position emission tomography scans, and magnetic resonance imaging. All patients were actively followed up during the initial six months after diagnosis, with subsequent annual follow-up by mailed questionnaires and annual verification of the patients’ vital status. These research protocols were also approved by the Mayo Clinic Institutional Review Board.

In addition to paclitaxel, many patients were also treated with radiation therapy and/or surgery, as well as 4 other classes of anticancer drugs: platinum agents, gemcitabine, EGFR inhibitors and etoposide. Detailed information is listed in Table 1.

**Table 1:** Clinical characteristics of 874 lung cancer patients treated with paclitaxel-based chemotherapy


**Description of patients who received taxane-based chemotherapy**	
	**Total (N = 874)**
**Age at Diagnosis**	
Mean (SD)	61.8 (10.4)
Median (range)	63.0 (34.0-86.0)
**Gender**	
Female	392 (44.9%)
Male	482 (55.1%)
**Cigarette smoking status**	
Never smokers	158 (18.1%)
Former smokers	428 (49%)
Current smokers	273 (31.2%)
Some smokers	15 (1.7%)
**Stage**	
** SCLC**	
Limited	37 (4.2%)
Extensive	39 (4.5%)
** NSCLC**	
Stage I and II	153 (17.5%)
Stage III	328 (37.5%)
Stage IV	317 (36.3%)
**Histologic cell type**	
Adenocarcinoma/bronchioloalveolar carcinoma	465 (53.2%)
Squamous cell carcinoma	162 (18.5%)
Small cell carcinoma	76 (8.5%)
Large cell carcinoma	28 (3.2%)
Mixed and unspecified NSCLC	131 (15.0%)
Others	12 (1.4%)
**Tumor differentiation grade**	
Well differentiated	76 (8.7%)
Moderately differentiated	332 (38.0%)
Poor/undifferentiated	395 (45.2%)
Nongradable or unknown	71 (8.1%)
**Surgery, chemotherapy, radiation**	
Chemotherapy	238 (27.2%)
Surgery & chemotherapy	157 (18%)
Radiation & chemotherapy	311 (35.6%)
Surgery & radiation & chemotherapy	168 (19.2%)

Stage for NSCLC is described on the basis of the tumor-node-metastasis classification. The small cell lung cancer stage is described as suggested by the American Cancer Society as either “Extensive” or “Limited.” Abbreviations: SCLC, small cell lung cancer, NSCLC, non-small cell lung cancer.

### Cell proliferation assay

Paclitaxel and docetaxel were purchased from Sigma-Aldrich (Milwaukee, WI). Drugs were dissolved in DMSO and aliquots of stock solutions were frozen at −80°C. Cell proliferation assays were performed in triplicate at each drug concentration. Specifically, 90 μl of cells (5 × 10^5^ cells/ml) were plated into each well of 96-well plates (Corning, Lowell, MA) [19] and were treated with 10 μl of paclitaxel or docetaxel at final concentrations of 0, 0.1, 1, 10, 15, 20, 100, 1000, 5000 nmol/L for paclitaxel and 0, 0.1, 1, 5, 7.5, 15, 100, 1000, 10000 nmol/L for docetaxel. 72 hours later, 20 μl of CellTiter 96 AQueous Non-Radioactive Cell Proliferation Assay solution (Promega Corporation, Madison, WI) were added to each well and incubated for an additional 3 hours. Plates were then read in a Safire2 microplate reader (Tecan AG, Switzerland). Experiments were successfully performed for 276 LCLs (93 AA, 87 CA and 96 HCA). The cytotoxicity assays for the lung cancer cell lines were conducted in a similar fashion except paclitaxel was added after the cells were incubated overnight. The final concentrations of paclitaxel were 0, 0.1, 1, 10, 25, 50, 100, 1000, 5000 nmol/L.

### Genome-wide SNPs in LCLs

Illumina HumanHap 550 K and 510S BeadArrays, containing 561,298 and 493,750 SNPs respectively, were used to genotype DNA samples from the LCLs in the Genotype Shared Resource (GSR) at Mayo Clinic, Rochester, MN. Publicly available Affymetrix SNP Array 6.0 Chip SNP data were also obtained for the same cell lines, which assayed 643,600 SNPs not covered on the Illumina BeadChips. The genotyping data were used in our previous studies [20,21] and are public available from NCBI Gene Expression Omnibus under SuperSeries accession No. GSE24277. SNPs that deviated from Hardy-Weinberg Equilibrium (HWE) based on the minimum p-value from an exact test for HWE [24] and the stratified test for HWE [25] (p-values < 0.001); SNPs with call rates < 95%; or SNPs with minor allele frequencies (MAFs) < 5% were removed from the analysis.

### Expression array assays in LCLs

Total RNA was extracted from each of the cell lines using Qiagen RNeasy Mini kits (QIAGEN, Inc.). RNA quality was tested using an Agilent 2100 Bioanalyzer, followed by hybridization to Affymetrix U133 Plus 2.0 Gene-Chips. The expression array data was used in our previous studies [19-21] and is public available from NCBI Gene Expression Omnibus under SuperSeries accession no. GSE24277 and accession No. GSE23120.

### MiRNA array assays in LCLs

Total RNA including miRNA from each LCL was extracted using mirVana^TM^ miRNA isolation kit (Ambion, Austin, TX). RNA quality was measured using RiboGreen® RNA Quantitation Kit (Molecular Probes, Eugene, OR) in an Agilent 2100 Bioanalyzer. Like described before [26], miRNA array assay was performed using Illumina’s human miRNA BeadArray according to the workflow on Illumina website. Briefly, total RNA were polyadenylated and converted to cDNA using a biotinylated oligo-dT primer with a universal PCR sequenced at its 5' end, followed by the annealing and extension of miRNA-specific oligonucleotide pool (MSO), which consists of a universal PCR priming site at the 5' end, an address sequence complementary to a capture sequence on the BeadArray and a microRNA-specific sequence at the 3' end. Then cDNA was amplified and subsequently hybridized to Illumina Sentrix Array Matrix (SAM)-Bead microarray chips. The SAMs were imaged using an Illumina BeadArray Reader, and microarray data were processed and analyzed using Illumina BeadStudio version 3.1.1. Probe-samples with a signal that was significantly higher than the background detection level were retained (at a significance level of 0.01). Probes with missingness ≥ 80% and individuals with missingness ≥ 50% were removed. The log2 expression levels were adjusted for an observed plate effect; there was no evidence of differential expression by ethnicity. Probes which had expression levels with standard deviation < 0.40 were deemed insufficiently variable to be informative, and potentially reflected only a background level of intensity. These were removed, leaving a final set of 226 probes and 282 individuals.

### Genotyping in lung cancer patients

The 170 top SNPs selected from our taxane GWAS in LCLs were used to genotype 874 lung cancer patient DNA samples using a custom-designed Illumina Golden Gate platform at Mayo Clinic, Rochester, MN. The concordance rate among three genomic control DNA samples (CEPH family trio, Coriell Institute) present in duplicate on each 96 well plate was 100%. After removing the subjects with call rates < 90%, SNPs with call rates < 95% and monomorphic SNPs, 153 SNPs (90%) were used for the analysis.

### Transient transfection and RNA interference

siRNA pools for candidate genes and negative control were purchased from Dharmacon (Chicago, IL). Reverse transfection of siRNA was performed in 96-well plates with a mixture of either non-small cell lung cancer, A549 cells, or small cell lung cancer, H196 cells and 0.3 μL of lipofectamine^TM^ RNAi-MAX reagent (Invitrogen), as well as 30 nmol/L siRNA pools.

### Real-time quantitative reverse transcription-PCR (qRT-PCR)

Total RNA was isolated from cultured cells transfected with negative control or specific siRNA pools using Quick-RNA^TM^ MiniPrep kit (Zymo Research, Orange, CA), followed by qRT-PCR performed with the Power SYBR_®_ Green RNA-to-C_T_^TM^ 1-Step Kit (AB Foster CA). Specifically, primers purchased from QIAGEN were used to perform qRT-PCR using the Stratagene Mx3005P Real-Time PCR detection system (Stratagene). All experiments were performed with beta-actin as an internal control.

### Statistical methods

#### Genome-wide analysis in LCLs

The taxane cytotoxicity phenotype IC50, indicating the drug concentration which inhibits half of maximal cell growth, was calculated based on the Brain–Cousen model [27,28] using the R package “drc” for each individual cell line for each drug separately. As described previously [21], prior to association the SNPs and IC50 values, the Van der Waerden (rank) transformed IC50 and SNPs were adjusted for gender, race and population stratification. To perform the SNP and mRNA gene expression associations, the mRNA expression array data were normalized using GCRMA, log_2_ transformed, and adjusted for gender, race, population stratification, and batch effect [29]. For miRNA and mRNA gene expression analyses, the normalized, log_2_ transformed mRNA expression array data were only adjusted for gender, race, and batch, while the miRNA expression array data were transformed using a Van der Waerden (rank) transformation and adjusted for gender, race and batch. The miRNA and SNP associations used genotype and Van der Waerden (rank) transformed miRNA expression data that were both adjusted for gender, race and population stratification.

To quantify the association of the adjusted IC50 phenotype with genome-wide SNPs (imputed and genotyped), Pearson correlations were calculated with adjusted SNPs. Likewise, the associations of SNPs with mRNA expression, SNPs with miRNA expression, as well as miRNA with mRNA expression were quantified by Pearson correlations using adjusted SNPs, mRNA and miRNA expression. SNPs in regions of interest were imputed with MACH v1.0 [30] using the HapMap Release 22 (Phase II) phased haplotype data as the reference. Specifically, SNPs for AA were imputed using both CEU (Utah residents with Northern and Western European ancestry from the CEPH collection) and YRI (Yoruba in Ibadan, Nigeria) data, SNPs for CA were imputed based on CEU data, and SNPs for HCA were imputed based on the CHB (Han Chinese in Beijing, China) and JPT (Japanese in Tokyo, Japan) data.

#### Clinical lung cancer patient analysis

The overall survival time was used as the primary endpoint, defined as the time from lung cancer diagnosis to either death or the last known date alive. Patients known to be alive were censored at the time of last contact. To test for the effect of SNP genotypes on overall survival, we used the Cox regression model that included the effects of a SNP genotype dosage (count of minor allele). A total of 153 SNPs were included in this analysis, and the association was performed for NSCLC and SCLC separately because of the significant differences between the two diseases. To correct for multiple testing of the 153 SNPs in the two lung cancer subsets (a total of 306 tests), a Bonferroni corrected p-value threshold of 0.0001 was used to determine statistically significant associations. To determine whether associations with SNPs should be adjusted for the clinical covariates of age at diagnosis, gender, smoking status, disease stage, and treatment, backward selection was performed. The disease stage was included in the final multivariate Cox-regression model as it was significantly associated with the overall survival of lung cancer patients. The disease stage was divided into five categories: small cell lung cancer with stages limited versus extensive; NSCLC with stages I + II, versus III versus IV. Since the effect of the SNPs on overall survival might be influenced by histologic subtypes among NSCLC patients, the association of three major histologic cell types (Adenocarcinoma/bronchioloalveolar carcinoma, squamous cell carcinoma and mixed and unspecified NSCLC) with overall survival was also tested with adjustment of disease stage and no significant association was found (p-value = 0.86). We used 0.05 as a cutoff for p-values (not adjusted for multiple testing) to select SNPs/genes for further functional validation.

## Results

### Paclitaxel and docetaxel cytotoxicity in LCLs

As both taxanes are used in clinical practice and share common mechanisms of action, cytotoxicity assays were performed for both drugs to determine the range of variation in individual drug response. We used IC50 as a phenotype to indicate the drug sensitivity for each cell line. The range of IC50 values for paclitaxel and docetaxel were 3.98-21.36 nmol/L and 1.54-13.32 nmol/L, respectively, and the median values were 9.35 nmol/L and 4.29 nmol/L. There was no evidence of differences in IC50 between genders (p = 0.82, 0.71) or races (p = 0.45, 0.14) in the paclitaxel and docetaxel experiments, respectively.

### Genome-wide SNP associations with IC50 values for two taxanes

**Figure 2:**
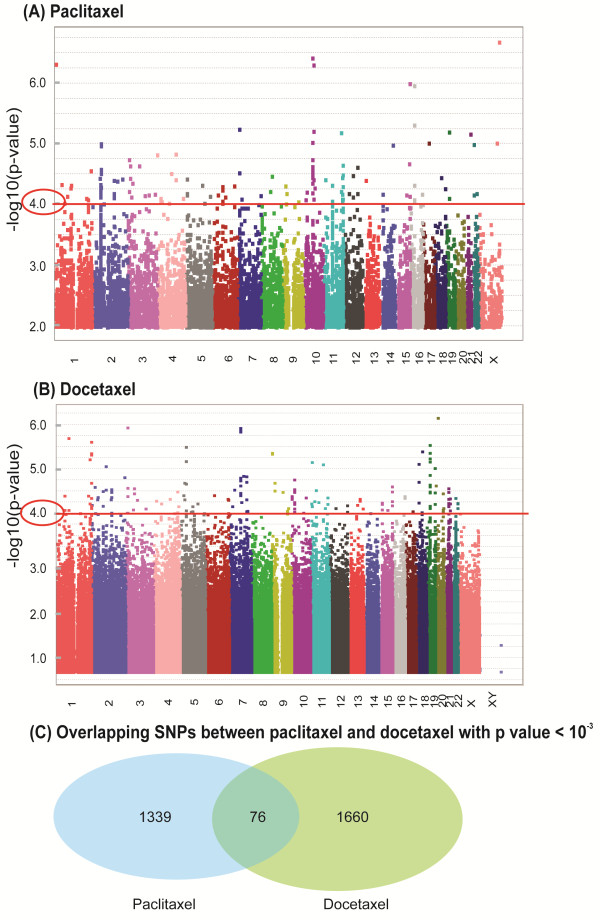
**1.3 million genome-wide SNPs associations with taxane IC50s in LCLs.** (**A** and **B**) Genome-wide SNP association with paclitaxel (**A**) or docetaxel IC50 values (**B**). The y-axis represents -log_10_(p-values) for the association of each SNP with paclitaxel/docetaxel IC50 values. SNPs are plotted on the x-axis based on their chromosomal locations. A p-value of 10^-4^ is highlighted with a red line. (**C**) The number of overlapping SNPs associated with IC50s for both paclitaxel and docetaxel with p-value < 10^-3^.

As described previously [21], after the quality control of all SNPs genotyped with the Illumina HumanHap 550 K, 510S BeadChips and Affymetrix SNP Array 6.0 Chip, approximately 1.3 million SNPs were used for the association analyses between genome-wide SNPs and IC50 values for paclitaxel and docetaxel to identify SNPs that might contribute to variation in drug cytotoxicity phenotypes. As shown in Figure 2A-B and Additional file 1: Tables S1–S2, none of the SNPs remained significant after Bonferroni correction. The most significant SNPs associated with paclitaxel or docetaxel IC50, rs10521792 and rs6044112, had p-values of 2.04 × 10^-7^ and 6.90 × 10^-7^, respectively. The rs10521792 SNP is 300 kb upstream from the 5^′^-end of the *FGF13* gene and the rs6044112 SNP is within an intron of *C20orf23*. For paclitaxel, 147 SNPs within or near 88 unique genes had p-values < 10^-4^ for association with IC50, while docetaxel had 180 SNPs within 102 unique genes meeting these criteria. One thousand and fifteen and 1736 SNPs had association p-values < 10^-3^ for paclitaxel and docetaxel, respectively (Additional file 1: Tables S1–S2, Figure 2C). As paclitaxel and docetaxel belong to the same class of antimicrotubule agents, we also compared the set of SNPs with p-value < 10^-3^ between these two drugs, of which 76 SNPs in 55 genes were in common between the top set of SNPs for both drugs (Additional file 1: Table S3, Figure 2C).

### Association study for lung cancer patients treated with taxane-based therapy

Taxanes are one of the most commonly used chemotherapeutic agents in the treatment of lung cancer patients, either alone or in combination with other anticancer drugs. We wanted to determine whether the top candidate SNPs identified during our GWAS using the cell line model system might be associated with overall survival of patients treated with taxanes. We took advantage of 874 germline DNA samples collected from lung cancer patients treated with paclitaxel at Mayo Clinic, including 76 SCLC and 798 NSCLC with well characterized phenotypes, to test this hypothesis. Detailed patient characteristics are described in Table 1.

Since almost all of the 874 lung cancer patient included in the study were treated with paclitaxel, we selected the top SNPs identified from our paclitaxel GWAS in LCLs for genotyping in those 874 lung cancer patients, including 147 SNPs associated with paclitaxel IC50 with p-value < 10^-4^ and 76 “overlapping SNPs” associated with both taxane IC50s with p-value <10^-3^ (Figure 1). After removing SNPs with lower Illumina design scores and SNPs with absolute linkage disequilibrium (r^2^ = 1), 170 SNPs were genotyped using the Illumina Golden Gate platform. For quality control, we excluded SNPs with low call rate and monomorphic SNPs, which resulted in 153 SNPs being analyzed (Additional file 1: Table S4). As shown in Table 2, the Cox regression analysis indicated that 11 SNPs were associated with SCLC or NSCLC overall survival with p-value < 0.05, although none of them were statistically significant after Bonferroni correction. The most significant SNPs, rs1106697 and rs11079337, were associated with SCLC or NSCLC overall survival with p-values of 0.007. SNP rs1106697, which was located on chromosome 7 and with a MAF of 0.106, was associated with overall survival in both NSCLC (p = 0.016, HR = 1.237) and SCLC (p = 0.007, HR = 1.875) patients. The hazard ratio (HR) > 1 means that patients carrying the minor allele had poor survival. In other words, the same SNP would be expected to be associated with higher IC50 values in our LCLs, and the cells carrying this SNP would be expected to be more resistant to paclitaxel. Therefore, we compared the association direction for clinical overall survival with that of the LCL results. We found that 8 out of 11 SNPs showed concordant association directions between the two phenotypes (Table 2).

**Table 2:** 8 SNPs associated with paclitaxel response in both LCLs and lung cancer patients

**SNP**	**Lung cancer patients**					**LCL**					**Chr**	**Position**	**Gene symbol**	**Location**	**Location relative to gene (bp)**
	**NSCLC**		**SCLC**		**MAF**	**Paclitaxel**		**Docetaxel**		**MAF**					
	**P value**	**HR**	**P value**	**HR**		**P value**	**R value**	**P value**	**R value**						
rs7519667	---	---	0.028	0.543	0.178	2.71E-05	−0.261	---	---	0.308	1	239,951,930	WDR64	intron	−1,292
rs10193067	---	---	0.039	0.372	0.043	3.61E-05	0.254	---	---	0.085	2	52,543,917	ASB3	flanking_3UTR	−1,206,705
rs2700868	0.028	1.193	---	---	0.157	6.56E-05	0.248	---	---	0.260	3	183,922,829	ATP11B	upstream	71,156
rs2662411	---	---	0.039	0.666	0.417	6.36E-05	−0.246	---	---	0.333	5	10,186,704	FAM173B	downstream	92,734
rs1106697	0.016	1.237	0.007	1.875	0.106	7.00E-05	0.245	---	---	0.092	7	155,365,705	SHH	upstream	67,977
rs1778335	---	---	0.019	1.602	0.308	6.04E-05	0.248	---	---	0.211	10	22,972,154	PIP4K2A	intron	0
rs11629576	---	---	0.011	0.637	0.447	6.-0577E	0.245	---	---	0.466	15	76,294,371	ACSBG1	intron	−6,872
rs11079337	0.007	1.168	---	---	0.366	5.10E-04	−0.215	7.85E-04	−0.208	0.338	17	53,517,495	DYNLL2	intron	−1,452
rs17079623	0.016	1.234	---	---	0.122	6.94E-04	0.210	3.32E-04	0.222	0.190	18	64,544,220	TXNDC10	flanking_5UTR	−10,887
rs7260598	0.020	0.821	---	---	0.162	6.27E-06	−0.277	2.46E-05	−0.259	0.165	19	24,014,626	ZNF254	upstream	47,190
rs17304569	0.019	0.818	---	---	0.161	7.76E-05	−0.243	6.74E-05	−0.245	0.163	19	24,032,745	ZNF254	upstream	29,071

11 of 153 SNPs were associated with SCLC or NSCLC overall survival with p-value < 0.05. The hazard ratio (HR) > 1 means that patients carrying the minor allele had poor survival. In other words, the same SNP would be expected to be associated with higher IC50 values in our LCLs, i.e., r-value > 0. After comparing the association direction for clinical overall survival with that of the LCL results, 8 out of 11 SNPs showed concordant association directions between the two phenotypes. The 3 SNPs showed opposite association directions were rs10193067, rs11629576 and rs11079337.

### Follow-up analyses

#### Imputation analysis in LCLs

In order to identify the causal SNPs or additional SNPs that were in strong linkage with the causal SNPs contributing to paclitaxel response, we imputed SNPs based on HapMap data using the genotyping results of LCLs for a region containing 200 kb up-/downstream of the 8 SNPs that were consistently associated with both paclitaxel IC50 in LCLs and overall survival of lung cancer patients. As shown in Additional file 1: Figure S1, imputed SNPs were found to have p-values of association with paclitaxel IC50 < 10^-3^ in the region 200 kb up-/downstream of SNPs rs1778335, rs2662411, rs7260598, rs17304569 and rs7519667. However, none of the imputed SNPs showed a stronger association with paclitaxel IC50 than did the observed SNPs.

#### SNP-expression association analysis in LCLs

SNPs might influence paclitaxel response through the regulation of gene expression in a cis-regulation manner. Therefore, we limited our SNP-Expression analyses to 11 genes which encompassed the 8 SNPs of interest noted previously by being within 200 kb of the location of the genes. We further excluded expression probes that were deemed not to be expressed, defined as an expression level of less than 50. None of the SNPs which had p-values for association with paclitaxel IC50 of < 10^-3^ were found to be associated with this set (possibly cis regulating) of expression probes (data not show).

#### Integrated SNP-miRNA-mRNA expression association analysis in LCLs

In addition to the direct effect of SNP on gene expression, SNP might alter gene expression through influence on miRNA expression [31]. We further performed the integrated SNP-miRNA-expression association analysis using these 8 SNPs, expression of 11 genes and 226 microRNAs. SNP rs2662411, close to gene *CMBL*, was associated with miRNA expression of hsa-miR-584 with p-value = 3.05 × 10^-5^ (r-value = 0.254). The hsa-miR-584 was also associated with *CMBL* mRNA levels with p-value = 7.46 × 10^-4^ (r-value = 0.301). Similarly, SNP rs1778335, close to gene *PIP4K2A*, was associated with the expression of hsa-miR-1468 with p-value = 1.57 × 10^-3^ (r-value = 0.194), and this microRNA was associated with mRNA expression of *PIP4K2A* with p-value = 8.24 × 10^-3^ (r-value = −0.267).

### SiRNA screening in lung cancer cell lines

**Figure 3:**
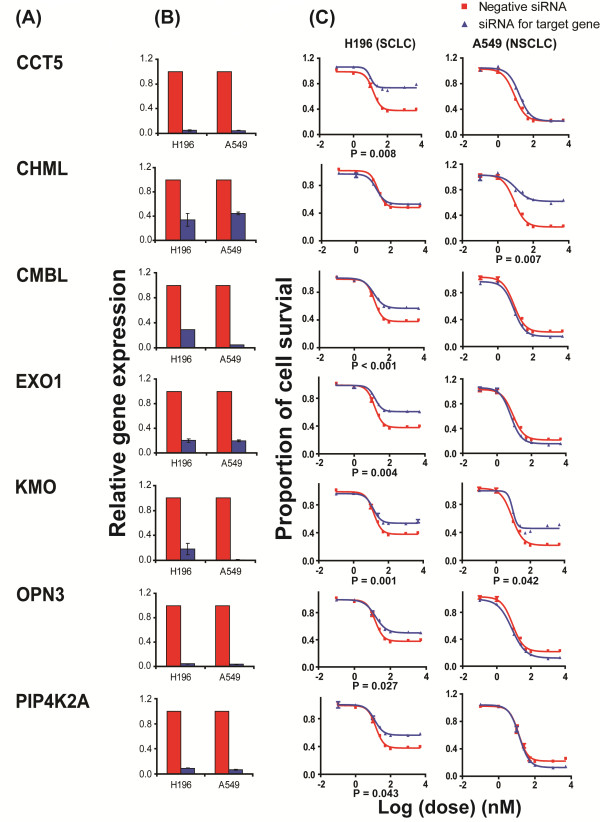
**siRNA screening of candidate genes by MTS assay in lung cancer cell lines.** Data are shown for 7 of the 11 candidate genes that were functionally validated in a SCLC cell line, H196, and an NSCLC cell line, A549, by MTS assay after knockdown with specific siRNA pools. (Red) Data for negative control siRNA; (blue) data for specific siRNAs. “Significance” was defined as a gene with a significant change in apparent area under the curve (AUC) in comparison with control siRNA as indicated by the p-values. For those genes with significant change in paclitaxel response, at least three independent experiments were performed in triplicate. Error bar represents standard error of the mean (SEM) for all of the experiments. (**A**) Candidate gene symbols. (**B**) qRT-PCR. The y-axis indicates relative gene expression after siRNA knockdown when compared with negative control siRNA. (C) MTS assays. The x-axis indicates the log transformed paclitaxel dose, and the y-axis indicates proportion survival after drug treatment.

As shown in Table 1, except for paclitaxel, these 874 lung cancer patients were also treated with one or several of the following drugs: platinum compounds, gemcitabine, EGFR inhibitors or etoposide. Although the genotyped SNPs were selected based on their association with taxane IC50 values in LCLs, the SNP effects on lung cancer overall survival might be influenced by other treatments. To further validate the association results, we also investigated mechanisms by which those 8 SNPs might have an effect on paclitaxel response. One of the mechanisms by which SNPs might affect phenotypes is through their influences on transcription regulation in either a *cis* or a *trans*- manner. Unfortunately, we did not have enough power to assess the trans-regulation. Although none of the 8 SNPs showed a significant *cis* effect, there could also be other SNPs in LD with these 8 SNPs that we did not genotype or SNPs with low allele frequencies that could. Therefore, we tested the possible effect of the 11 genes close to those 8 SNPs on drug response by performing knockdown experiments in a SCLC cell line, H196, and a NSCLC cell line, A549, to determine if changing gene expression could influence paclitaxel-induced cytotoxicity. As shown in Figure 3 and Table 3, MTS assay indicated that knockdown of *PIP4K2A*, *CCT5*, *CMBL, EXO1*, *KMO* and *OPN3*, which were close to SNPs rs1778335, rs2662411 and rs7519667, significantly desensitized paclitaxel-induced cytotoxicity in the SCLC cell line H196, and those 3 SNPs were also associated with SCLC overall survival with p-value < 0.05 (Table 2). In addition, in the NSCLC cell line, A549, knockdown of the genes, *CHML* and *KMO*, which were close to rs7519667, also had a significant effect on paclitaxel cytotoxicity.

**Table 3:** siRNA screening of 11 candidate genes by MTS assay

**SNP**	**Basis for selection**				**MTS assay**	
	**SNP vs paclitaxel and/or docetaxel IC50 in LCLs**	**SNP vs survival in SCLC patients**	**SNP vs survival in NSCLC patients**	**Gene symbol**	**SCLC**	**NSCLC**
	**(p < 10**^**-3**^**)**	**(p < 0.05)**	**(p < 0.05)**		**H196**	**A549**
rs7519667	Yes	Yes		*CHML*	---	Yes
				*EXO1*	Yes	---
				*KMO*	Yes	Yes
				*OPN3*	Yes	---
rs2700868	Yes		Yes	*ATP11B*	---	---
rs2662411	Yes	Yes		*CCT5*	Yes	---
				*CMBL*	Yes	---
rs1106697	Yes	Yes	Yes	*RBM33*	---	---
rs1778335	Yes	Yes		*PIP4K2A*	Yes	---
rs17079623	Yes		Yes	*TMX3*	---	---
rs17304569 & rs7260598	Yes		Yes	*ZNF254*	---	---

“Yes” under “Basis for selection” indicates individual candidate genes with the p-value listed. For the MTS assay, “Yes” indicates that knockdown of the gene altered taxane cytotoxicity when compared with control siRNA. All of the experiments with significant changes were performed in triplicate and were replicated at least three times.

## Discussion

Taxanes, including paclitaxel and docetaxel, are microtubule-stabilizing anticancer agents commonly used in the treatment of SCLC and NSCLC. Large inter-individual variation in taxane response has been observed in lung cancer patients in both efficacy and toxicities associated with taxane, such as peripheral neuropathy [32,33]. This large variation is caused by many different factors, including: tumor genetics, host genetics as well as the microenvironment [34]. Many previous studies have demonstrated that germline genetic polymorphisms can play a significant role in individual variability in taxane-induced efficacy and toxicity [9-13,15-18].

In order to understand biological mechanisms underlying the variation in response to taxane and to identify novel biomarkers which might be helpful for individualized taxane chemotherapy, we performed pharmacogenomic studies of paclitaxel and docetaxel in 276 LCLs, followed by association studies of candidate SNPs identified during the analysis in LCLs using DNA samples from NSCLC and SCLC patients treated with paclitaxel. We then performed functional studies of candidate genes by siRNA knockdown in lung cancer cell lines. In this study, we mainly focused on genes that might influence the mechanism of drug action, i.e., pharmacodynamics, as genes involved in taxane pharmacokinetic pathways, such as *CYP2C8*, *CYP3A4/A5,* which have been well studied in previous taxane pharmacogenomic studies, were not highly expressed in our LCLs.

Genome-wide analyses were performed using 1.3 million genome-wide SNPs and paclitaxel or docetaxel IC50 values for 276 LCLs. The analyses resulted in the identification of a series of candidate SNPs that were associated with cytotoxicity phenotypes for two taxanes (Figure 2 and Additional file 1: Tables S1–S3). Although none of the SNPs maintained statistical significance after Bonferroni correction, 147 and 180 SNPs had p-values for association with paclitaxel or docetaxel IC50 of < 10^-4^, and 76 SNPs overlapped between the two taxanes with p-values < 10^-3^. A previous GWAS from an ongoing phase III clinical trial, CALGB 40101, identified 3 top SNPs located in the *EPHA5*, *FGD4* and *NDRG1* genes that were associated with paclitaxel-induced peripheral neuropathy, although none reached genome-wide significance [18]. In our study, 3 SNPs located 200-300 kb upstream of *EPHA5* genes and 1 SNP ~14 kb upstream of *NDRG1* were also found to be associated with paclitaxel IC50 values with p-value < 10^-3^ in LCLs, but not with docetaxel IC50. Therefore, these SNPs were not included in the subsequent genotyping study with lung cancer patient samples based on our selection criteria.

Like all model systems, the LCL model system has limitations. The variation of taxane response in LCLs might be influenced by EBV transformation-induced cellular changes and non-genetic factors such as cell growth rate or baseline ATP levels [35-37]. To further test whether any of these candidate SNPs might be associated with overall survival for lung cancer patients treated with taxanes, we genotyped the 147 top SNPs associated with paclitaxel IC50 and 76 SNPs which overlapped between the two taxanes in LCLs using DNA samples from 76 SCLC and 798 NSCLC patients after paclitaxel-based chemotherapy. In this study, instead of specific taxane response outcomes, overall survival was used as the clinical phenotype. Therefore, the association results could be affected by many other factors, such as histology, stage, performance and treatment. In order to adjust for those confounding factors, Cox regression analysis was performed to test the effect of clinical covariates on overall survival, including age at diagnosis, gender, smoking status, disease stage, and treatment. As a result, disease stage was included in the final multivariate Cox-regression model as it was significantly associated with the overall survival of lung cancer patients. That association study identified 8 SNPs that were associated with SCLC or NSCLC overall survival with p-values < 0.05, although none of the SNPs were statistically significant after Bonferroni correction (Table 2). The statistical power for association with overall survival of SCLC patients was low. Therefore, we did functional studies by using siRNA knockdown, followed by MTS assays, in a SCLC cell line, H196, and a NSCLC cell line, A549, for 11 candidate genes chosen based on their proximity to the 8 SNPs and their expression levels in LCLs. Knockdown of *PIP4K2A*, *CCT5*, *CMBL, EXO1*, *KMO* and *OPN3*, 6 genes that were close to the 3 SNPs (rs1778335, rs2662411 and rs7519667) associated with SCLC overall survival, significantly desensitized H196 cells to paclitaxel (Figure 3). Knockdown of *CHML* and *KMO,* two genes that were close to rs7519667, also had a significant effect on paclitaxel response in A549 cells (Figure 3). Integrated SNP-miRNA-mRNA expression association analysis indicated that SNPs rs2662411 and rs1778335 were associated with mRNA expression of *CMBL* or *PIP4K2A* through hsa-miR-584 or hsa-miR-1468. In our study, SNP rs2662411 was associated with higher miRNA expression of hsa-miR-584 (r-value = 0.254, p-value = 3.05 × 10^-5^), which was associated with higher mRNA expression of *CMBL* (r-value = 0.301, p-value = 7.46 × 10^-4^); in SCLC cell line, knockdown of *CMBL* caused paclitaxel resistance; those results were consistent with the association of SNP rs2662411 with lower paclitaxel IC50 in LCLs (r-value = −0.245, p-value = 6.36 × 10^-5^) and better overall survival in SCLC patients (HR = 0.666, p-value = 0.039) (Table 1). Similarly, in LCLs SNP rs1778335 was associated with higher expression of hsa-miR-1468 (r-value = 0.194, p-value = 1.57 × 10^-3^), which was associated with lower expression of *PIP4K2A* (r-value = −0.267, p-value = 8.24 × 10^-3^); in SCLC cell line knockdown of *PIP4K2A* resulted in paclitaxel resistance; those results were consistent with the association of SNP rs1778335 with higher paclitaxel IC50 in LCLs (r-value = 0.248, p-value = 6.04 × 10^-5^) and worse overall survival in SCLC patients (HR = 1.602, p-value = 0.019). However, no corresponding miRNA binding sites was found in either *CMBL* or *PIP4K2A* from microRNA public database, future experiment will be performed to validate these results.

Previous studies indicated that an individual miRNA could affect expression of multiple genes and an individual mRNA might also be regulated by multiple miRNAs, which was mainly through miRNA targeting 3^′^ untranslated region (3^′^-UTR) of mRNA [38]. SNPs located in the miRNA-coding genes or miRNA-binding site of mRNA could influence the pathogenesis of disease or drug response through affecting the biogenesis of miRNA or binding of miRNA with mRNA [31,39]. SNP 829C > T in the 3^′^UTR of dihydrofolate reductase (DHFR), which was located in the miR24 microRNA binding site, has been reported that it caused the loss of miR24 function and resulted in DHFR overexpression and methotrexate (MTX) resistance [40]. *CMBL* gene encoded carboxymethylenebutenolidase homolog (CMBL), which was a cysteine hydrolase of the dienelactone hydrolase family and was involved in the metabolism of prodrug olmesartan medoxomil [41]. The homology of CMBL protein among human, mouse and rat were more than 80%. In human CMBL was widely expressed in many tissues, especially in liver and intestine [41]. A proteomic study by Yang et al. found that CMBL was an H2AX-interacting protein [42], which suggested that CMBL might be involved in cellular responses to DNA damage and DNA repair.

*PIP4K2A* gene encoded phosphatidylinositol-5-phosphate 4-kinase, type II, alpha (PtdIns5P 4-kinase α). As a major type of type II PtdIns5P 4-kinases, it was involved in the conversion of phophatidylinositol-5-phosphate (PtdIns5*P*) into phosphatidylinosital-4,5-bisphosphate [PtdIns(4,5)*P*_2_[43]. Since the cellular level of phophatidylinositol-4-phosphate (PtdIns4*P*), which was another source to form PtdIns(4,5)*P*_2_, was approximately ten times higher than that of PtdIns5*P*, the major function of type II PtdIns5*P* 4-kinases was most probably to regulate the level of PtdIns5*P*[44,45]. There were three mammalian isoforms for type II PtdIns5*P* 4-kinases: α, β and γ [46]. PtdIns5*P* 4-kinase α was located in both cytoplasm and nucleus, and could form homodimer or heterodimer with PtdIns5*P* 4-kinase β or γ [45,47]. In vitro assays indicated that PtdIns5*P* 4-kinase α had the highest enzyme activity [43], and knockdown of PtdIns5*P* 4-kinase α significantly enhanced the tyrosine-kinase regulated PtdIns5*P* production [48]. Although no obvious phenotype was found for knockout of PtdIns5*P* 4-kinase α, the double knockout of PtdIns5*P* 4-kinase α and β was found to be embryonic lethal [43]. Several previous studies also demonstrated that PtdIns5*P* was a second messenger in cellular signaling, PtdIns5*P* could activate PI 3-kinase/Akt pathway [49] and protect Akt from dephosphorylation through inhibition of PP2A phosphatise [50], and in nucleus PtdIns5*P* could bind to inhibitor of growth protein-2 (ING2) and regulate p53-mediated response to DNA damage [51]. However, the mechanisms of those two genes involved in paclitaxel response still remain unknown, further mechanistic studies will be required.

For the other four genes (*CCT5*, *EXO1, KMO* and *OPN3*) that were close to the 2 SNPs (rs2662411 and rs7519667), our association analyses and knockdown experiments suggested a potential function in paclitaxel response. However, no significant *cis* relationship was found by either SNP-expression association analysis or integrated SNP-miRNA-mRNA expression association analysis. One possibility is that there might be rare variants in LD with those 2 SNPs that might be the causal SNPs regulating gene expression. In addition, the effect of SNP on gene expression was tissue specific. Therefore, future deep resequencing of these regions might help to identify rare variants to test this hypothesis, and we also need to perform the SNP-expression association analysis using lung cancer tissue samples.

## Conclusions

In summary, our GWAS in LCLs, together with translational studies with DNA samples from lung cancer patients, followed by functional studies in lung cancer cell lines showed that 6 genes, *PIP4K2A*, *CCT5*, *CMBL, EXO1*, *KMO* and *OPN3*, genes that are close to 3 SNPs associated with SCLC overall survival (rs1778335, rs2662411 and rs7519667), significantly altered paclitaxel cytotoxicity in the SCLC cell line, H196. SNPs rs2662411 and rs1778335 might regulate mRNA expression of *CMBL* and *PIP4K2A* through influence on miRNA expression of hsa-miR-584 or hsa-miR-1468. These results provide additional insight into genes that may contribute to variation in response to taxanes and genetic variations that may be associated with overall survival of paclitaxel-treated lung cancer patients. We acknowledge that the patient population used in the association study is heterogenous and that our phenotype, overall survival, could be influenced by multiple factors other than the treatment. Although we adjusted for all the known factors during the association studies, we cannot exclude the possibility that the genetic variations identified might be prognostic factors rather than taxane predictive factors. Further confirmation of these findings using specific taxane response outcome in additional homogeneous patient cohorts would seem to be warranted.

## Abbreviations

SNPs: Single nucleotide polymorphisms; GWAS: Genome-wide association study; LCLs: Lymphoblastoid cell lines; SCLC: Small cell lung cancer; NSCLC: Non-small cell lung cancer; miRNA: microRNA; CEPH: Centre d’Etude du Polymorhpisme Humain; QTL: Quantitative trait loci; GWA: Genome-wide association; AA: African-American; CA: Caucasian-American; HCA: Han Chinese-American; FBS: Fetal Bovine Serum; GSR: Genotype Shared Resource; HWE: Hardy-Weinberg Equilibrium; MAFs: Minor allele frequencies; MSO: miRNA-specific oligonucleotide pool; SAM: Sentrix Array Matrix; qRT-PCR: Real-time Quantitative Reverse Transcription-PCR; CEU: Utah residents with Northern and Western European ancestry from the CEPH collection; YRI: Yoruba in Ibadan, Nigeria; CHB: Han Chinese in Beijing, China; JPT: Japanese in Tokyo, Japan; HR: Hazard ratio; 3’-UTR: 3’ untranslated region; DHFR: Dihydrofolate reductase; MTX: Methotrexate; CMBL: Carboxymethylenebutenolidase homolog; PtdIns5P 4-kinase α: Phosphatidylinositol-5-phosphate 4-kinase, type II, alpha; PtdIns5P: Phophatidylinositol-5-phosphate; PtdIns(4,5)P2: Phosphatidylinosital-4,5-bisphosphate; PtdIns4P: Phophatidylinositol-4-phosphate; ING2: Inhibitor of growth protein-2; AUC: Area under the curve; SEM: Standard error of the mean.

## Competing interests

The authors declare that they have no competing interest.

## Authors’ contributions

NN and LW designed the study and wrote the manuscript. DJS, RPA, BLF, GJ, AB and AGB performed the statistical analyses. RPA and KK conducted bioinformatic analysis. QF and JMC did the miRNA array assay. LL helped with the optimization of cell proliferation assay and functional validation of candidate genes. ZS and PY collected clinical lung cancer patient samples. All the authors read, revised the draft manuscript and approved the final version.

## Pre-publication history

The pre-publication history for this paper can be accessed here:

http://www.biomedcentral.com/1471-2407/12/422/prepub

## Supplementary Material

Additional file 1: Figure S1 Imputation analysis for 8 SNPs associated with both paclitaxel IC50 in LCLs and overall survival in lung cancer patients. SNPs within 200 kb up-/downstream of those 8 SNPs were imputed. Black circle indicate SNPs observed by genotyping, while red triangle indicate imputed SNPs. The y-axis represents –log_10_(p-value) for the association of each SNP with paclitaxel IC50, and the x-axis represents the chromosome location of the SNPs. **Table S1.** Top 1415 SNPs that were associated with paclitaxel IC50 with p-values < 10^-3^ and top 147 SNPs that were significantly associated with paclitaxel IC50 values with p-values < 10^-4^. R values represent correlation coefficients for the associations. **Table S2.** Top 1736 SNPs that were associated with docetaxel IC50 with p-values <10^-3^ and top 180 SNPs were significantly associated with docetaxel IC50 values with p-values <10^-4^. R values represent correlation coefficients for the association. **Table S3.** 76 SNPs were associated with IC50s for both paclitaxel and docetaxel with p-values < 10^-3^. **Table S4.** Results of Cox regression analysis with overall survival for either SCLC or NSCLC patients. The SNPs associated with overall survival with p-value < 0.05 are highlighted. Click here for file

## References

[B1] McGrogan BT Gilmartin B Carney DN McCann A Taxanes, microtubules and chemoresistant breast cancer Biochim Biophys Acta 2008 1785 2 96 132 18068131 10.1016/j.bbcan.2007.10.004

[B2] Zhao J Kim JE Reed E Li QQ Molecular mechanism of antitumor activity of taxanes in lung cancer (Review) Int J Oncol 2005 27 1 247 256 15942666

[B3] Cancer Facts & Figures 2012 2012 Atlanta: American Cancer Society

[B4] Goffin J Lacchetti C Ellis PM Ung YC Evans WK First-line systemic chemotherapy in the treatment of advanced non-small cell lung cancer: a systematic review J Thorac Oncol 2010 5 2 260 274 10.1097/JTO.0b013e3181c6f035 20101151

[B5] Rodriguez E Lilenbaum RC Small cell lung cancer: past, present, and future Curr Oncol Rep 2010 12 5 327 334 10.1007/s11912-010-0120-5 20632219

[B6] Lee JJ Swain SM Peripheral neuropathy induced by microtubule-stabilizing agents J Clin Oncol 2006 24 10 1633 1642 10.1200/JCO.2005.04.0543 16575015

[B7] Rowinsky EK Donehower RC Paclitaxel (taxol) N Engl J Med 1995 332 15 1004 1014 10.1056/NEJM199504133321507 7885406

[B8] Rigas JR Taxane-platinum combinations in advanced non-small cell lung cancer: a review Oncologist 2004 9 Suppl 2 16 23 15161987 10.1634/theoncologist.9-suppl_2-16

[B9] Rosell R Taron M Alberola V Massuti B Felip E Genetic testing for chemotherapy in non-small cell lung cancer Lung Cancer 2003 41 Suppl 1 S97 102 12867068 10.1016/s0169-5002(03)00151-x

[B10] Isla D Sarries C Rosell R Alonso G Domine M Taron M Lopez-Vivanco G Camps C Botia M Nunez L Single nucleotide polymorphisms and outcome in docetaxel-cisplatin-treated advanced non-small-cell lung cancer Ann Oncol 2004 15 8 1194 1203 10.1093/annonc/mdh319 15277258

[B11] Gandara DR Kawaguchi T Crowley J Moon J Furuse K Kawahara M Teramukai S Ohe Y Kubota K Williamson SK Japanese-US common-arm analysis of paclitaxel plus carboplatin in advanced non-small-cell lung cancer: a model for assessing population-related pharmacogenomics J Clin Oncol 2009 27 21 3540 3546 10.1200/JCO.2008.20.8793 19470925 PMC2717760

[B12] Leskela S Jara C Leandro-Garcia LJ Martinez A Garcia-Donas J Hernando S Hurtado A Vicario JC Montero-Conde C Landa I Polymorphisms in cytochromes P450 2C8 and 3A5 are associated with paclitaxel neurotoxicity Pharmacogenomics J 2010 [Epub ahead of print] 10.1038/tpj.2010.13 20212519

[B13] Sissung TM Mross K Steinberg SM Behringer D Figg WD Sparreboom A Mielke S Association of ABCB1 genotypes with paclitaxel-mediated peripheral neuropathy and neutropenia Eur J Cancer 2006 42 17 2893 2896 10.1016/j.ejca.2006.06.017 16950614 PMC1647318

[B14] Rochat B Role of cytochrome P450 activity in the fate of anticancer agents and in drug resistance: focus on tamoxifen, paclitaxel and imatinib metabolism Clin Pharmacokinet 2005 44 4 349 366 10.2165/00003088-200544040-00002 15828850

[B15] Spratlin J Sawyer MB Pharmacogenetics of paclitaxel metabolism Crit Rev Oncol Hematol 2007 61 3 222 229 10.1016/j.critrevonc.2006.09.006 17092739

[B16] Watters JW Kraja A Meucci MA Province MA McLeod HL Genome-wide discovery of loci influencing chemotherapy cytotoxicity Proc Natl Acad Sci U S A 2004 101 32 11809 11814 10.1073/pnas.0404580101 15282376 PMC511056

[B17] O'Brien C Cavet G Pandita A Hu X Haydu L Mohan S Toy K Rivers CS Modrusan Z Amler LC Functional genomics identifies ABCC3 as a mediator of taxane resistance in HER2-amplified breast cancer Cancer Res 2008 68 13 5380 5389 10.1158/0008-5472.CAN-08-0234 18593940

[B18] Kroetz DL Baldwin RM Owzar K Jiang C Zembutsu H Kubo M Nakamura Y Shulman LN Ratain MJ Inherited genetic variation in EPHA5, FGD4, and NRDG1 and paclitaxel (P)-induced peripheral neuropathy (PN): results from a genome-wide association study (GWAS) in CALGB 40101 J Clin Oncol 2010 28 15s

[B19] Li L Fridley B Kalari K Jenkins G Batzler A Safgren S Hildebrandt M Ames M Schaid D Wang L Gemcitabine and cytosine arabinoside cytotoxicity: association with lymphoblastoid cell expression Cancer Res 2008 68 17 7050 7058 10.1158/0008-5472.CAN-08-0405 18757419 PMC2562356

[B20] Li L Fridley BL Kalari K Jenkins G Batzler A Weinshilboum RM Wang L Gemcitabine and arabinosylcytosin pharmacogenomics: genome-wide association and drug response biomarkers PLoS One 2009 4 11 e7765 10.1371/journal.pone.0007765 19898621 PMC2770319

[B21] Niu N Qin Y Fridley BL Hou J Kalari KR Zhu M Wu TY Jenkins GD Batzler A Wang L Radiation pharmacogenomics: a genome-wide association approach to identify radiation response biomarkers using human lymphoblastoid cell lines Genome Res 2010 20 11 1482 1492 10.1101/gr.107672.110 20923822 PMC2963812

[B22] Yang P Sun Z Krowka MJ Aubry MC Bamlet WR Wampfler JA Thibodeau SN Katzmann JA Allen MS Midthun DE Alpha1-antitrypsin deficiency carriers, tobacco smoke, chronic obstructive pulmonary disease, and lung cancer risk Arch Intern Med 2008 168 10 1097 1103 10.1001/archinte.168.10.1097 18504338 PMC2562773

[B23] Moyer AM Sun Z Batzler AJ Li L Schaid DJ Yang P Weinshilboum RM Glutathione pathway genetic polymorphisms and lung cancer survival after platinum-based chemotherapy Cancer Epidemiol Biomarkers Prev 2010 19 3 811 821 10.1158/1055-9965.EPI-09-0871 20200426 PMC2837367

[B24] Wigginton JE Cutler DJ Abecasis GR A note on exact tests of Hardy-Weinberg equilibrium Am J Hum Genet 2005 76 5 887 893 10.1086/429864 15789306 PMC1199378

[B25] Schaid DJ Batzler AJ Jenkins GD Hildebrandt MA Exact tests of Hardy-Weinberg equilibrium and homogeneity of disequilibrium across strata Am J Hum Genet 2006 79 6 1071 1080 10.1086/510257 17186465 PMC1698709

[B26] Cunningham JM Oberg AL Borralho PM Kren BT French AJ Wang L Bot BM Morlan BW Silverstein KA Staggs R Evaluation of a new high-dimensional miRNA profiling platform BMC Med Genomics 2009 2 57 10.1186/1755-8794-2-57 19712457 PMC2744682

[B27] Brain P Cousens R An equation to describe dose responses where there is stimulation of growth at low doses Weed Res 1989 29 2 93 96 10.1111/j.1365-3180.1989.tb00845.x

[B28] Van Ewijk PH Hoekstra JA Calculation of the EC50 and its confidence interval when subtoxic stimulus is present Ecotoxicol Environ Saf 1993 25 1 25 32 10.1006/eesa.1993.1003 7682915

[B29] Wu ZJ Irizarry RA Gentleman R Martinez-Murillo F Spencer F A model-based background adjustment for oligonucleotide expression arrays J Am Stat Assoc 2004 99 468 909 917 10.1198/016214504000000683

[B30] Li YAG Mach 1.0: Rapid Haplotype Reconstruction and Missing Genotype Inference Am J Hum Genet 2006 S79 2290

[B31] Bandiera S Hatem E Lyonnet S Henrion-Caude A microRNAs in diseases: from candidate to modifier genes Clin Genet 2010 77 4 306 313 10.1111/j.1399-0004.2010.01370.x 20132241

[B32] Gligorov J Lotz JP Preclinical pharmacology of the taxanes: implications of the differences Oncologist 2004 9 Suppl 2 3 8 15161985 10.1634/theoncologist.9-suppl_2-3

[B33] Mielke S Sparreboom A Mross K Peripheral neuropathy: a persisting challenge in paclitaxel-based regimes Eur J Cancer 2006 42 1 24 30 10.1016/j.ejca.2005.06.030 16293411

[B34] Stewart DJ Tumor and host factors that may limit efficacy of chemotherapy in non-small cell and small cell lung cancer Crit Rev Oncol Hematol 2010 75 3 173 234 10.1016/j.critrevonc.2009.11.006 20047843 PMC2888634

[B35] Sie L Loong S Tan EK Utility of lymphoblastoid cell lines J Neurosci Res 2009 87 9 1953 1959 10.1002/jnr.22000 19224581

[B36] Choy E Yelensky R Bonakdar S Plenge RM Saxena R De Jager PL Shaw SY Wolfish CS Slavik JM Cotsapas C Genetic analysis of human traits in vitro: drug response and gene expression in lymphoblastoid cell lines PLoS Genet 2008 4 11 e1000287 10.1371/journal.pgen.1000287 19043577 PMC2583954

[B37] Stark AL Zhang W Zhou T O'Donnell PH Beiswanger CM Huang RS Cox NJ Dolan ME Population differences in the rate of proliferation of international HapMap cell lines Am J Hum Genet 2010 87 6 829 833 10.1016/j.ajhg.2010.10.018 21109222 PMC2997375

[B38] Cai Y Yu X Hu S Yu J A brief review on the mechanisms of miRNA regulation Genomics Proteomics Bioinformatics 2009 7 4 147 154 10.1016/S1672-0229(08)60044-3 20172487 PMC5054406

[B39] Mishra PJ Mishra PJ Banerjee D Bertino JR MiRSNPs or MiR-polymorphisms, new players in microRNA mediated regulation of the cell: introducing microRNA pharmacogenomics Cell Cycle 2008 7 7 853 858 10.4161/cc.7.7.5666 18414050

[B40] Mishra PJ Humeniuk R Mishra PJ Longo-Sorbello GS Banerjee D Bertino JR A miR-24 microRNA binding-site polymorphism in dihydrofolate reductase gene leads to methotrexate resistance Proc Natl Acad Sci U S A 2007 104 33 13513 13518 10.1073/pnas.0706217104 17686970 PMC1948927

[B41] Ishizuka T Fujimori I Kato M Noji-Sakikawa C Saito M Yoshigae Y Kubota K Kurihara A Izumi T Ikeda T Human carboxymethylenebutenolidase as a bioactivating hydrolase of olmesartan medoxomil in liver and intestine J Biol Chem 2010 285 16 11892 11902 10.1074/jbc.M109.072629 20177059 PMC2852926

[B42] Yang X Zou P Yao J Yun D Bao H Du R Long J Chen X Proteomic dissection of cell type-specific H2AX-interacting protein complex associated with hepatocellular carcinoma J Proteome Res 2010 9 3 1402 1415 10.1021/pr900932y 20000738 PMC3670604

[B43] Clarke JH Irvine RF The activity, evolution and association of phosphatidylinositol 5-phosphate 4-kinases Adv Enzyme Regul 2011 Epub ahead of print 10.1016/j.advenzreg.2011.09.002 21945524

[B44] van den Bout I Divecha N PIP5K-driven PtdIns(4,5)P2 synthesis: regulation and cellular functions J Cell Sci 2009 122 Pt 21 3837 3850 19889969 10.1242/jcs.056127

[B45] Clarke JH Wang M Irvine RF Localization, regulation and function of type II phosphatidylinositol 5-phosphate 4-kinases Adv Enzyme Regul 2010 50 1 12 18 10.1016/j.advenzreg.2009.10.006 19896968 PMC2877797

[B46] Clarke JH Richardson JP Hinchliffe KA Irvine RF Type II PtdInsP kinases: location, regulation and function Biochem Soc Symp 2007 74 149 159 10.1042/BSS0740149 17233588

[B47] Bultsma Y Keune WJ Divecha N PIP4Kbeta interacts with and modulates nuclear localization of the high-activity PtdIns5P-4-kinase isoform PIP4Kalpha Biochem J 2010 430 2 223 235 10.1042/BJ20100341 20583997

[B48] Wilcox A Hinchliffe KA Regulation of extranuclear PtdIns5P production by phosphatidylinositol phosphate 4-kinase 2alpha FEBS Lett 2008 582 9 1391 1394 10.1016/j.febslet.2008.03.022 18364242

[B49] Carricaburu V Lamia KA Lo E Favereaux L Payrastre B Cantley LC Rameh LE The phosphatidylinositol (PI)-5-phosphate 4-kinase type II enzyme controls insulin signaling by regulating PI-3,4,5-trisphosphate degradation Proc Natl Acad Sci U S A 2003 100 17 9867 9872 10.1073/pnas.1734038100 12897244 PMC187868

[B50] Ramel D Lagarrigue F Dupuis-Coronas S Chicanne G Leslie N Gaits-Iacovoni F Payrastre B Tronchere H PtdIns5P protects Akt from dephosphorylation through PP2A inhibition Biochem Biophys Res Commun 2009 387 1 127 131 10.1016/j.bbrc.2009.06.139 19576174

[B51] Jones DR Bultsma Y Keune WJ Halstead JR Elouarrat D Mohammed S Heck AJ D'Santos CS Divecha N Nuclear PtdIns5P as a transducer of stress signaling: an in vivo role for PIP4Kbeta Mol Cell 2006 23 5 685 695 10.1016/j.molcel.2006.07.014 16949365

