# Supplementary material for: Genetic association with overall survival of taxane-treated lung cancer patients - a genome-wide association study in human lymphoblastoid cell lines followed by a clinical association study
Source: BMC Cancer. 2012 Sep 24;12:422. doi: 10.1186/1471-2407-12-422 (PMC3573965; doi:10.1186/1471-2407-12-422)
Supplement: Additional file 1: Figure S1 — Imputation analysis for 8 SNPs associated with both paclitaxel IC50 in LCLs and overall survival in lung cancer patients. SNPs within 200 kb up-/downstream of those 8 SNPs were imputed. Black circle indicate SNPs observed by genotyping, while red triangle indicate imputed SNPs. The y-axis represents –log10(p-value) for the association of each SNP with paclitaxel IC50, and the x-axis represents the chromosome location of the SNPs. Table S1. Top 1415 SNPs that were associated with paclitaxel IC50 with p-values < 10-3 and top 147 SNPs that were significantly associated with paclitaxel IC50 values with p-values < 10-4. R values represent correlation coefficients for the associations. Table S2. Top 1736 SNPs that were associated with docetaxel IC50 with p-values <10-3 and top 180 SNPs were significantly associated with docetaxel IC50 values with p-values <10-4. R values represent correlation coefficients for the association. Table S3. 76 SNPs were associated with IC50s for both paclitaxel and docetaxel with p-values < 10-3. Table S4. Results of Cox regression analysis with overall survival for either SCLC or NSCLC patients. The SNPs associated with overall survival with p-value < 0.05 are highlighted. [file 1471-2407-12-422-S1.pdf]

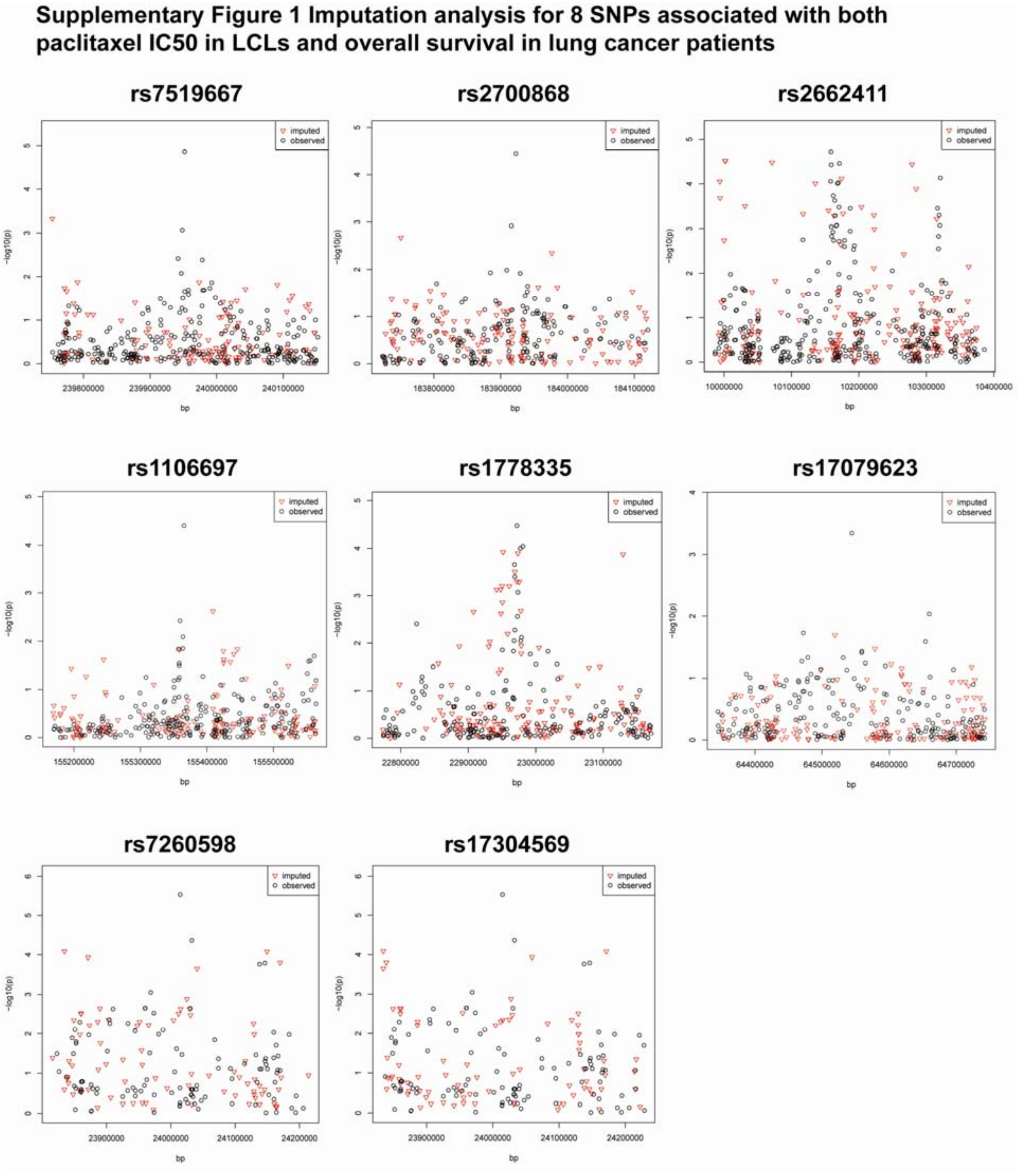

Supplementary Figure 1. Imputation analysis for 8 SNPs associated with both paclitaxel IC50 in LCLs and overall survival in lung cancer patients. SNPs within 200kb up-/downstream of those 8 SNPs were imputed. Black circles indicate SNPs observed by genotyping, while red triangles indicate imputed SNPs. The y-axis represents $-\log_{10}$(p-value) for the association of each SNP with paclitaxel IC50, and the x-axis represents the chromosomal locations of the SNPs.

Supplementary Table 1. Top 1415 SNPs that were associated with paclitaxel IC50 with p-values <10^{-3} and top 147 SNPs that were significantly associated with paclitaxel IC50 values with p-values <10^{-4}. R values represent correlation coefficients for associations.

SNP ID	P value	R value	Q value	MAF	Chr	Position	GeneSymbol	RefSeq ID	Location	Location Relative to Gene (bp)
rs10521792	2.04E-07	-0.317	0.163	0.353	X	137,945,075	*FGF13*	NM_033642.1	flanking_5UTR	-295,894
rs1350172	3.79E-07	0.310	0.163	0.415	10	63,018,773	*C10orf107*	NM_173554.1	flanking_5UTR	-73,952
rs548726	4.78E-07	0.307	0.163	0.203	1	5,836,208	*NPHP4*	NM_015102.2	flanking_3UTR	-9,249
rs1227969	4.85E-07	-0.307	0.163	0.351	10	70,933,270	*TSPAN15*	NM_012339	intron	0
rs184234	9.90E-07	-0.299	0.241	0.161	15	99,069,242	*ASB7*	NM_198243.1	flanking_3UTR	-62,068
rs4787484	1.08E-06	0.299	0.241	0.301	16	29,814,047	*SEZ6L2*	NM_012410.1	intron	-34
rs4787483	4.72E-06	0.281	0.702	0.165	16	29,792,948	*SEZ6L2*	NM_012410.1	intron	-397
rs6967385	5.65E-06	0.280	0.702	0.393	7	12,357,844	*TMEM106B*	NM_018374.2	flanking_3UTR	-114,433
rs1652804	6.11E-06	-0.279	0.702	0.355	10	70,934,331	*TSPAN15*	NM_012339.3	intron	-84
rs7260598	6.27E-06	-0.277	0.702	0.165	19	24,014,626	*ZNF254*	NM_203282	upstream	47,190
rs4936613	6.36E-06	0.279	0.702	0.088	11	120,709,811	*SC5DL*	NM_006918.3	flanking_3UTR	-25,232
rs915832	6.76E-06	-0.276	0.702	0.366	21	42,148,564	*PRDM15*	NM_022115	intron	0
rs12657996	8.96E-06	0.273	0.702	0.315	5	158,836,891	*IL12B*	ENST00000306675	upstream	439,427
rs2787702	9.26E-06	0.272	0.702	0.406	10	62,977,481	*TMEM26*	NM_178505	upstream	94,636
rs9895585	9.37E-06	-0.272	0.702	0.147	17	39,420,513	*PYY*	NM_004160.3	intron	-16,772
rs9698139	9.54E-06	0.274	0.702	0.476	X	122,799,241	*BIRC4*	NM_001167.2	flanking_5UTR	-22,488
rs243016	9.64E-06	0.273	0.702	0.133	2	60,442,217	---	ENST00000363937	upstream	23,027
rs580204	9.93E-06	0.272	0.702	0.469	22	19,321,062	*PCQAP*	NM_001003891.1	flanking_3UTR	-49,143
rs2430363	1.02E-05	0.273	0.702	0.151	14	91,434,804	*FBLN5*	NM_006329.2	intron	-3,635
rs243037	1.04E-05	0.271	0.702	0.167	2	60,452,604	---	ENST00000386566	downstream	677,114
rs2036203	1.44E-05	0.266	0.864	0.290	4	125,894,505	*ANKRD50*	NM_020337.1	flanking_5UTR	-43,123
rs6852065	1.46E-05	-0.266	0.864	0.469	4	1,654,207	*FAM53A*	NM_001013622	upstream	13,941
rs9883082	1.75E-05	-0.264	0.864	0.317	3	7,858,963	*GRM7*	NM_000844.2	flanking_3UTR	-100,746
rs2650741	1.76E-05	0.264	0.864	0.417	10	63,002,133	*C10orf107*	NM_173554.1	flanking_5UTR	-90,592
rs12898337	2.08E-05	0.263	0.864	0.350	15	97,111,878	*IGF1R*	NM_000875.2	intron	-43,019
rs11222869	2.16E-05	-0.261	0.864	0.476	11	131,406,958	*HNT*	NM_016522	intron	0
rs612051	2.25E-05	0.262	0.864	0.090	3	15,050,364	*NR2C2*	NM_003298	intron	0
rs7630682	2.27E-05	0.260	0.864	0.270	3	76,824,325	*ROBO2*	NM_002942	upstream	348,302
rs1869927	2.32E-05	0.260	0.864	0.426	10	62,984,478	*TMEM26*	NM_178505	upstream	101,633
rs2540457	2.37E-05	-0.260	0.864	0.491	12	94,995,995	*LTA4H*	NM_000895.1	flanking_5UTR	-42,499
rs2650717	2.46E-05	0.259	0.864	0.424	10	62,978,075	*TMEM26*	NM_178505	upstream	95,230
rs2787703	2.46E-05	0.259	0.864	0.424	10	62,978,720	*C10orf107*	NM_173554	upstream	114,005
rs243044	2.57E-05	0.259	0.864	0.141	2	60,449,297	*BCL11A*	NM_138559.1	flanking_3UTR	-82,509
rs7519667	2.71E-05	-0.261	0.864	0.308	1	239,951,930	*WDR64*	NM_144625.2	intron	-1,292
rs243050	2.72E-05	0.258	0.864	0.132	2	60,445,531	*BCL11A*	NM_138559.1	flanking_3UTR	-86,275
rs6460895	2.90E-05	0.259	0.864	0.425	7	12,219,065	*TMEM106B*	NM_018374	upstream	1,888
rs2257189	2.91E-05	0.258	0.864	0.425	10	62,988,025	*TMEM26*	NM_178505	upstream	105,180
rs243039	2.98E-05	0.257	0.864	0.151	2	60,451,069	*BCL11A*	NM_138559.1	flanking_3UTR	-80,737
rs7657460	3.01E-05	0.257	0.864	0.063	4	96,076,324	*BMPR1B*	NM_001203	intron	0
rs6460900	3.13E-05	0.260	0.864	0.410	7	12,219,613	*TMEM106B*	ENST00000262042	upstream	576,774
rs1939717	3.16E-05	-0.257	0.864	0.476	11	131,405,946	*HNT*	NM_016522.2	intron	-115,440
rs7995709	3.19E-05	-0.256	0.864	0.120	13	105,810,213	---	ENST00000378993	intron	0
rs1532326	3.21E-05	0.257	0.864	0.334	12	64,203,455	*MSRB3*	NM_001031679	downstream	56,510

rs7812453	3.35E-05	0.255	0.864	0.382	8	72,917,594	*MSC*	NM_005098.2	intron	-58
rs2814032	3.43E-05	0.255	0.864	0.418	10	63,006,689	---	ENST00000389639	intron	0
rs11822830	3.52E-05	-0.255	0.864	0.359	11	128,966,226	*BARX2*	NM_003658	downstream	139,212
rs2606094	3.53E-05	0.258	0.864	0.418	10	62,994,853	*C10orf107*	NM_173554.1	flanking_5UTR	-97,872
rs9961704	3.54E-05	-0.254	0.864	0.413	18	46,072,686	*CXXC1*	NM_014593.2	flanking_5UTR	-4,500
rs7625117	3.60E-05	0.254	0.864	0.087	3	15,137,581	*ZFYVE20*	NM_022340.2	flanking_5UTR	-21,922
rs10193067	3.61E-05	0.254	0.864	0.085	2	52,543,917	*ASB3*	NM_145863.1	flanking_3UTR	-1,206,705
rs11744434	3.64E-05	0.254	0.864	0.361	5	10,164,180	*LOC134145*	NM_199133.1	flanking_3UTR	-115,265
rs16838023	3.66E-05	-0.254	0.864	0.297	2	206,761,661	*GPR1*	NM_005279.2	intron	-11,417
rs313997	3.78E-05	0.254	0.864	0.293	4	125,902,380	*ANKRD50*	NM_020337.1	flanking_5UTR	-50,998
rs972570	3.79E-05	-0.255	0.864	0.409	10	70,942,024	*TSPAN15*	NM_012339.3	flanking_3UTR	-4,595
rs1528657	3.83E-05	0.253	0.864	0.252	11	13,947,120	*SPON1*	NM_006108.1	intron	-5,855
rs9510315	3.90E-05	0.253	0.864	0.082	13	22,218,655	*FTHL7*	NR_002202.1	flanking_3UTR	-49,745
rs6708612	3.92E-05	0.253	0.864	0.091	2	147,882,193	---	ENST00000384496	upstream	187
rs1349497	3.98E-05	-0.253	0.864	0.251	2	173,300,909	*RAPGEF4*	NM_007023	upstream	7,933
rs7653262	4.31E-05	-0.252	0.864	0.356	3	30,418,873	*TGFBR2*	NM_003242.4	flanking_5UTR	-204,125
rs2814027	4.32E-05	-0.252	0.864	0.245	10	63,012,118	*C10orf107*	NM_173554.1	flanking_5UTR	-80,607
rs1098182	4.51E-05	-0.251	0.864	0.116	1	43,558,355	*TIE1*	NM_005424	intron	0
rs1030333	4.59E-05	0.257	0.864	0.354	2	56,554,526	*FANCL*	NM_018062	downstream	1,685,358
rs7189550	4.61E-05	0.251	0.864	0.375	16	29,818,761	*SEZ6L2*	NM_201575.1	flanking_5UTR	-687
rs338466	4.62E-05	0.251	0.864	0.476	1	109,287,719	*CLCC1*	NM_015127.2	coding	[99/41]
rs3797730	4.67E-05	0.251	0.864	0.130	5	111,109,607	*C5orf13*	NM_004772.1	intron	-9,759
rs7931234	4.72E-05	0.254	0.864	0.388	11	58,771,325	---	ENST00000387068	upstream	32,577
rs6914925	4.75E-05	0.251	0.864	0.276	6	149,411,678	*UST*	NM_005715.1	intron	-24,984
rs12377344	4.83E-05	0.250	0.864	0.163	9	19,871,986	*SLC24A2*	NM_020344.1	flanking_5UTR	-95,060
rs17527707	4.91E-05	0.251	0.864	0.246	6	67,424,871	*LOC442229*	XR_000273.2	flanking_3UTR	-868,778
rs1793640	4.95E-05	0.250	0.864	0.487	11	131,426,908	*HNT*	NM_016522.2	intron	-94,478
rs1236904	5.07E-05	-0.255	0.864	0.390	10	70,937,855	*TSPAN15*	ENST00000242462	downstream	63,605
rs243052	5.10E-05	0.249	0.864	0.129	2	60,443,761	*BCL11A*	NM_138559.1	flanking_3UTR	-88,045
rs11578246	5.16E-05	-0.251	0.864	0.447	1	104,897,024	---	ENST00000388703	downstream	2,250,705
rs1885822	5.16E-05	-0.249	0.864	0.480	10	77,284,010	*C10orf11*	NM_032024.2	intron	-71,224
rs10193613	5.21E-05	0.249	0.864	0.346	2	56,549,101	*VRK2*	NM_006296	upstream	1,578,123
rs4614971	5.23E-05	-0.249	0.864	0.486	2	56,541,133	*EFEMP1*	NM_004105.2	flanking_5UTR	-537,451
rs12956238	5.32E-05	0.255	0.864	0.284	18	70,654,268	*ZNF407*	NM_017757.1	intron	-31,822
rs7857311	5.36E-05	0.250	0.864	0.089	9	22,955,874	*ELAVL2*	ENST00000380137	downstream	513,402
rs1037576	5.65E-05	0.248	0.864	0.244	6	67,429,648	---	ENST00000364559	downstream	597,833
rs17038123	5.80E-05	-0.248	0.864	0.473	2	56,482,575	*EFEMP1*	NM_004105.2	flanking_5UTR	-478,893
rs7008176	5.91E-05	0.247	0.864	0.286	8	61,994,553	*CHD7*	NM_017780.2	flanking_3UTR	-52,534
rs2324413	5.93E-05	-0.248	0.864	0.311	3	7,861,455	*GRM7*	NM_000844	downstream	158,341
rs6589877	5.93E-05	0.247	0.864	0.101	11	120,701,161	*SC5DL*	NM_006918.3	flanking_3UTR	-16,582
rs17047986	6.00E-05	-0.247	0.864	0.471	2	56,555,161	*VRK2*	NM_006296	upstream	1,572,063
rs1778335	6.04E-05	0.248	0.864	0.211	10	22,972,154	*PIP4K2A*	NM_005028	intron	0
rs2787716	6.15E-05	0.247	0.864	0.422	10	62,993,359	*C10orf107*	NM_173554	upstream	99,366
rs986787	6.18E-05	-0.247	0.864	0.462	2	56,529,867	*EFEMP1*	NM_004105.2	flanking_5UTR	-526,185
rs987576	6.25E-05	0.247	0.864	0.438	2	56,605,529	*EFEMP1*	NM_004105.2	flanking_5UTR	-601,847
rs196074	6.35E-05	-0.246	0.864	0.196	22	37,162,458	*KCNJ4*	NM_004981.1	intron	-7,464
rs2662411	6.36E-05	-0.246	0.864	0.333	5	10,186,704	---	ENST00000362925	downstream	372,586
rs12367762	6.38E-05	0.247	0.864	0.045	12	39,900,866	---	ENST00000380795	intron	0
rs6746117	6.42E-05	0.246	0.864	0.089	2	147,871,631	*ACVR2A*	NM_001616.3	flanking_5UTR	-447,409

rs10968243	6.46E-05	-0.246	0.864	0.105	9	27,964,789	*LINGO2*	NM_152570	intron	0
rs1561092	6.46E-05	-0.247	0.864	0.277	12	94,728,384	*NTN4*	NM_021229.2	flanking_5UTR	-19,717
rs11647148	6.51E-05	0.246	0.864	0.051	16	81,995,895	*CDH13*	NM_001257.3	intron	-59,783
rs34015250	6.53E-05	0.246	0.864	0.127	14	23,096,333	*THTPA*	NM_024328.2	coding	[526/20]
rs12787230	6.55E-05	-0.248	0.864	0.371	11	128,965,050	*BARX2*	NM_003658.3	flanking_3UTR	-138,025
rs2700868	6.56E-05	0.248	0.864	0.260	3	183,922,829	*ATP11B*	NM_014616	upstream	71,156
rs1117260	6.63E-05	0.246	0.864	0.399	2	56,520,625	*VRK2*	NM_006296	upstream	1,606,599
rs1030334	6.66E-05	0.247	0.864	0.350	2	56,554,449	*FANCL*	NM_018062	downstream	1,685,435
rs12527264	6.67E-05	-0.246	0.864	0.134	6	37,607,820	*C6orf129*	NM_138493.1	flanking_5UTR	-32,144
rs9841889	6.76E-05	-0.245	0.864	0.051	3	142,352,484	*SPSB4*	NM_080862.1	flanking_3UTR	-2,341
rs11629576	6.77E-05	0.245	0.864	0.466	15	76,294,371	*ACSBG1*	NM_015162.3	intron	-6,872
rs661440	6.77E-05	0.245	0.864	0.328	22	19,320,057	*PCQAP*	NM_001003891.1	flanking_3UTR	-48,138
rs10052112	6.78E-05	0.245	0.864	0.357	5	10,164,690	---	ENST00000362925	downstream	350,572
rs6794522	6.84E-05	0.245	0.864	0.134	3	115,120,114	*GRAMD1C*	NM_017577.2	intron	-2,739
rs1106697	7.00E-05	0.245	0.864	0.092	7	155,365,705	*SHH*	NM_000193	upstream	67,977
rs17069617	7.08E-05	0.245	0.864	0.274	5	167,376,943	---	ENST00000388904	intron	0
rs1281400	7.14E-05	-0.246	0.864	0.438	1	82,828,213	*LPHN2*	NM_012302	downstream	597,518
rs1561861	7.17E-05	-0.245	0.864	0.420	15	97,827,013	*LRRC28*	NM_144598	downstream	82,994
rs2869826	7.23E-05	0.247	0.864	0.469	2	56,522,951	*FANCL*	NM_018062	downstream	1,716,933
rs16879998	7.25E-05	-0.244	0.864	0.138	6	88,727,920	*SPACA1*	NM_030960.2	flanking_5UTR	-86,306
rs1159899	7.28E-05	-0.244	0.864	0.168	3	25,399,432	---	ENST00000264330	intron	0
rs1154818	7.37E-05	0.244	0.864	0.236	12	67,127,543	*MDM1*	NM_020128.1	flanking_5UTR	-115,193
rs12208629	7.39E-05	0.244	0.864	0.237	6	67,406,765	---	ENST00000364559	downstream	574,950
rs728621	7.48E-05	0.244	0.864	0.084	2	147,892,269	*ACVR2A*	NM_001616.3	flanking_5UTR	-426,771
rs3774921	7.59E-05	-0.247	0.864	0.361	4	23,419,745	*PPARGC1A*	NM_013261.2	intron	-3,723
rs6837393	7.73E-05	0.244	0.864	0.413	4	175,713,037	*HPGD*	NM_000860.3	flanking_5UTR	-32,851
rs6662529	7.75E-05	-0.244	0.864	0.232	1	209,289,328	*KCNH1*	NM_002238.2	intron	-30,107
rs17304569	7.76E-05	-0.243	0.864	0.163	19	24,032,745	*ZNF254*	NM_203282	upstream	29,071
rs2018361	7.85E-05	0.246	0.864	0.483	11	131,407,387	*HNT*	NM_016522.2	intron	-113,999
rs745382	7.90E-05	-0.244	0.864	0.356	11	128,967,443	*BARX2*	NM_003658.3	flanking_3UTR	-140,418
rs7789880	7.93E-05	0.249	0.864	0.188	7	27,883,619	*JAZF1*	NM_175061	intron	0
rs891562	7.96E-05	-0.243	0.864	0.149	10	70,944,743	*TSPAN15*	NM_012339.3	flanking_3UTR	-7,314
rs1019307	8.05E-05	0.244	0.864	0.454	7	12,218,315	*TMEM106B*	ENST00000262042	upstream	575,476
rs2461153	8.18E-05	-0.243	0.864	0.297	10	18,129,628	*LOC340843*	NM_001013629.1	3UTR	[1934/233]
rs7498372	8.18E-05	0.243	0.864	0.431	16	29,818,226	*SEZ6L2*	NM_201575.1	flanking_5UTR	-152
rs10495194	8.20E-05	0.243	0.864	0.112	1	220,800,594	*TAF1A*	NM_139352.1	intron	-735
rs12917823	8.27E-05	0.243	0.864	0.164	16	29,784,802	*CDIPT*	NM_145752.1	flanking_5UTR	-2,790
rs2510866	8.53E-05	-0.242	0.866	0.438	11	58,012,339	*OR5B12*	NM_001004733	upstream	48,139
rs12201163	8.54E-05	-0.243	0.866	0.416	6	67,545,449	*LOC442229*	XR_000273.2	flanking_3UTR	-989,356
rs4697045	8.59E-05	-0.242	0.866	0.362	4	23,421,855	*PPARGC1A*	NM_013261	intron	0
rs667520	8.70E-05	-0.243	0.866	0.339	6	138,057,818	*OLIG3*	ENST00000369137	downstream	1,464,907
rs10120811	8.72E-05	0.248	0.866	0.298	9	106,278,970	*OR13F1*	NM_001004485	upstream	27,395
rs980038	8.77E-05	-0.242	0.866	0.273	4	31,279,013	*PCDH7*	NM_032457.1	flanking_3UTR	-525,162
rs11820646	8.79E-05	-0.243	0.866	0.366	11	128,966,381	*BARX2*	NM_003658.3	flanking_3UTR	-139,356
rs2509920	8.80E-05	-0.244	0.866	0.438	11	58,093,120	*LPXN*	NM_004811	intron	0
rs12194460	9.17E-05	0.241	0.888	0.250	6	67,392,335	---	ENST00000365187	downstream	103,120
rs13120966	9.28E-05	0.242	0.888	0.274	4	80,763,498	*GDEP*	XR_000540.1	flanking_5UTR	-204,065
rs7705332	9.32E-05	-0.241	0.888	0.467	5	128,623,393	*ISOC1*	NM_016048.1	flanking_3UTR	-145,663
rs2482863	9.34E-05	0.242	0.888	0.124	1	62,160,186	*INADL*	NM_176878.1	intron	-5,804

rs7028484	9.43E-05	-0.241	0.888	0.276	9	23,536,461	*ELAVL2*	NM_004432.1	flanking_3UTR	-145,523
rs17040658	9.50E-05	-0.241	0.888	0.141	2	79,562,026	*CTNNA2*	NM_004389.2	flanking_5UTR	-31,608
rs2515368	9.59E-05	-0.244	0.888	0.437	11	58,020,757	*OR5B21*	NM_001005218.1	flanking_3UTR	-10,468
rs17048108	9.64E-05	-0.240	0.888	0.418	2	56,620,042	*EFEMP1*	NM_004105.2	flanking_5UTR	-616,360
rs12703414	9.67E-05	-0.240	0.888	0.167	7	141,336,135	*MGAM*	NM_004668.1	flanking_5UTR	-6,013
rs2334207	9.68E-05	0.241	0.888	0.487	16	86,138,699	*JPH3*	NM_020655.2	flanking_5UTR	-55,301
rs12886523	1.01E-04	0.240	0.904	0.123	14	23,092,022	*THTPA*	NM_024328.2	flanking_5UTR	-3,049
rs4788692	1.02E-04	-0.240	0.904	0.306	16	71,623,157	*ATBF1*	NM_006885.2	intron	-15,995
rs2936041	1.03E-04	0.240	0.904	0.120	1	220,829,396	*TAF1A*	NM_139352	intron	0
rs766045	1.03E-04	-0.240	0.904	0.084	8	3,968,386	*CSMD1*	NM_033225.3	intron	-91,357
rs7011493	1.03E-04	0.239	0.904	0.101	8	134,004,681	*TG*	NM_003235.4	intron	-84
rs3847	1.03E-04	0.239	0.904	0.397	11	58,733,287	---	ENST00000361050	downstream	1,477
rs17054300	1.04E-04	0.239	0.904	0.219	6	150,412,245	*ULBP3*	NM_024518.1	flanking_3UTR	-15,191
rs12465667	1.05E-04	-0.239	0.904	0.444	2	6,579,328	*FLJ42418*	NM_001001695.1	flanking_3UTR	-207,424
rs17062868	1.06E-04	0.239	0.904	0.100	9	78,471,990	*KIAA0367*	NM_015225.1	intron	-11,752
rs6598542	1.07E-04	0.241	0.904	0.369	15	97,110,579	*IGF1R*	NM_000875	intron	0
rs12921548	1.07E-04	0.239	0.904	0.159	16	6,786,272	---	ENST00000363999	upstream	77,629
rs1465359	1.09E-04	0.239	0.904	0.386	2	56,541,834	*VRK2*	NM_006296	upstream	1,585,390
rs2056992	1.09E-04	0.239	0.904	0.201	16	1,049,519	*FLJ32252*	NM_182510.1	flanking_3UTR	-4,575
rs720659	1.10E-04	0.239	0.904	0.238	7	27,882,743	*JAZF1*	NM_175061.2	intron	-18,621
rs3008653	1.11E-04	0.238	0.904	0.118	1	220,831,414	*TAF1A*	NM_139352	upstream	1,664
rs2518016	1.11E-04	0.238	0.904	0.476	11	131,389,651	*HNT*	NM_016522.2	intron	-102,899
rs2239704	1.12E-04	-0.240	0.904	0.352	6	31,648,120	*LTA*	NM_000595.2	5UTR	[48/82]
rs4917139	1.12E-04	-0.238	0.904	0.203	7	48,232,695	*ABCA13*	NM_152701.2	intron	-1,247
rs13243605	1.12E-04	-0.238	0.904	0.134	7	69,954,500	*AUTS2*	NM_015570.1	flanking_3UTR	-58,710
rs7171291	1.12E-04	-0.239	0.904	0.398	15	97,825,519	*LRRC28*	NM_144598.2	flanking_3UTR	-81,498
rs566390	1.13E-04	-0.238	0.904	0.417	11	131,400,074	*HNT*	NM_016522.2	intron	-113,322
rs2416095	1.13E-04	-0.238	0.904	0.205	14	47,104,610	*MDGA2*	NM_182830	intron	0
rs9859701	1.15E-04	0.238	0.912	0.091	3	124,083,960	*DIRC2*	NM_032839	downstream	2,514
rs11107127	1.16E-04	0.238	0.914	0.185	12	92,530,565	*SOCS2*	NM_003877.3	flanking_3UTR	-36,456
rs13382678	1.17E-04	0.238	0.916	0.353	2	56,542,552	*EFEMP1*	NM_004105.2	flanking_5UTR	-538,870
rs4944716	1.19E-04	-0.238	0.917	0.226	11	86,927,155	*FLJ22104*	NM_022918.2	flanking_3UTR	-214,935
rs35309020	1.19E-04	0.237	0.917	0.125	14	23,093,866	*THTPA*	NM_024328.2	flanking_5UTR	-1,205
rs2098366	1.20E-04	0.238	0.917	0.304	7	12,271,080	*TMEM106B*	NM_018374.2	flanking_3UTR	-27,669
rs10892723	1.20E-04	0.237	0.917	0.094	11	120,700,156	*SC5DL*	NM_006918	downstream	16,725
rs2350491	1.22E-04	-0.237	0.927	0.213	3	141,265,026	*CLSTN2*	NM_022131.1	intron	-112,457
rs3823612	1.24E-04	0.241	0.927	0.404	7	12,225,280	*TMEM106B*	NM_018374	intron	0
rs1938593	1.25E-04	0.237	0.927	0.446	11	58,741,293	*FAM111A*	NM_022074.2	flanking_3UTR	-62,205
rs3849508	1.26E-04	0.236	0.927	0.264	3	76,821,985	*ROBO2*	NM_002942.1	flanking_5UTR	-350,636
rs3008647	1.27E-04	0.236	0.927	0.116	1	220,825,630	*TAF1A*	NM_005681.2	intron	-1,368
rs17124200	1.28E-04	-0.236	0.927	0.089	1	63,232,402	*ATG4C*	NM_032852.2	flanking_3UTR	-129,764
rs6876709	1.28E-04	-0.236	0.927	0.359	5	10,337,270	*CMBL*	ENST00000296658	intron	0
rs579984	1.28E-04	-0.236	0.927	0.480	11	131,417,733	*HNT*	NM_016522	intron	0
rs2150602	1.29E-04	-0.237	0.927	0.120	10	107,931,867	*SORCS1*	NM_001013031.1	flanking_3UTR	-391,544
rs1521826	1.33E-04	0.236	0.927	0.455	8	84,248,116	*LOC138046*	NM_173848.3	flanking_5UTR	-1,010,025
rs10500367	1.33E-04	-0.236	0.927	0.205	16	9,214,168	*PRO0149*	NM_014117.2	flanking_3UTR	-93,112
rs2058991	1.37E-04	-0.235	0.927	0.071	2	214,640,825	*SPAG16*	NM_024532.3	intron	-40,340
rs310318	1.38E-04	-0.235	0.927	0.272	8	23,745,864	*STC1*	NM_003155.2	flanking_3UTR	-9,515
rs278953	1.39E-04	-0.236	0.927	0.124	4	40,202,761	*FLJ20273*	NM_019027.1	intron	-9,880

rs4645464	1.39E-04	-0.236	0.927	0.297	7	155,785,301	*C7orf13*	NM_032625	downstream	339,665
rs10490411	1.40E-04	-0.235	0.927	0.500	2	56,625,841	*FANCL*	NM_018062	downstream	1,614,043
rs10510599	1.40E-04	-0.238	0.927	0.329	3	27,950,267	*EOMES*	NM_005442.2	flanking_5UTR	-211,478
rs7563921	1.41E-04	0.235	0.927	0.480	2	56,472,870	*FANCL*	NM_018062	downstream	1,767,014
rs980816	1.41E-04	-0.235	0.927	0.416	X	7,493,102	*STS*	NM_000351	downstream	210,251
rs839547	1.42E-04	-0.235	0.927	0.489	1	109,295,670	*CLCC1*	NM_015127	intron	0
rs1468804	1.42E-04	0.235	0.927	0.408	7	12,242,033	*TMEM106B*	NM_018374	3UTR	0
rs2866834	1.43E-04	-0.237	0.927	0.130	7	69,967,370	*AUTS2*	ENST00000380812	downstream	573,940
rs6591509	1.43E-04	-0.235	0.927	0.364	11	58,737,125	*FAM111A*	NM_022074.2	flanking_3UTR	-58,037
rs453349	1.44E-04	-0.235	0.927	0.257	20	15,199,031	*C20orf133*	NM_001033086.1	intron	-40,324
rs16886725	1.45E-04	0.234	0.927	0.087	5	18,728,095	*CDH18*	NM_004934.2	flanking_3UTR	-780,803
rs6947878	1.45E-04	-0.234	0.927	0.346	7	12,306,020	---	ENST00000366337	downstream	59,465
rs7580549	1.46E-04	0.234	0.927	0.364	2	56,613,051	*FANCL*	NM_018062	downstream	1,626,833
rs996846	1.47E-04	0.235	0.927	0.451	2	56,618,150	*EFEMP1*	NM_004105.2	flanking_5UTR	-614,468
rs2114590	1.47E-04	-0.234	0.927	0.226	2	230,822,837	*SP140*	NM_007237	intron	0
rs1848069	1.48E-04	-0.235	0.927	0.058	3	43,392,353	*TMEM16K*	NM_018075.2	intron	-8,883
rs28374	1.50E-04	0.234	0.927	0.053	22	32,496,139	*LARGE*	NM_004737	intron	0
rs2121956	1.51E-04	0.239	0.927	0.383	1	104,890,919	*AMY1C*	NM_001008219.1	flanking_3UTR	-788,086
rs884355	1.51E-04	-0.235	0.927	0.411	5	10,162,791	*LOC134145*	NM_199133.1	flanking_3UTR	-116,654
rs1400142	1.51E-04	0.234	0.927	0.344	12	71,728,762	*TRHDE*	NM_013381.1	flanking_3UTR	-383,073
rs547335	1.51E-04	0.234	0.927	0.105	18	70,581,690	*ZNF407*	NM_017757.1	intron	-40,682
rs2828104	1.52E-04	0.236	0.927	0.415	21	23,686,623	*C21orf74*	XR_001010.1	flanking_3UTR	-1,275,908
rs17404627	1.54E-04	-0.234	0.927	0.096	8	3,957,010	*CSMD1*	NM_033225.3	intron	-79,981
rs17153461	1.54E-04	0.235	0.927	0.452	11	58,745,622	---	ENST00000378253	downstream	47,303
rs1438887	1.55E-04	0.240	0.927	0.216	2	147,882,898	*ACVR2A*	NM_001616.3	flanking_5UTR	-436,142
rs1171492	1.55E-04	0.233	0.927	0.203	10	22,981,043	*PIP4K2A*	NM_005028	intron	0
rs10831916	1.56E-04	-0.233	0.927	0.438	11	12,867,812	*TEAD1*	NM_021961.3	intron	-6,564
rs2000875	1.56E-04	0.233	0.927	0.391	11	58,738,162	*FAM111A*	NM_022074.2	flanking_3UTR	-59,074
rs7618590	1.57E-04	-0.235	0.927	0.169	3	25,397,780	---	ENST00000264330	intron	0
rs9567479	1.57E-04	0.233	0.927	0.168	13	44,256,131	*NUFIP1*	NM_012345	downstream	155,253
rs17047946	1.58E-04	0.234	0.927	0.398	2	56,534,424	*VRK2*	NM_006296	upstream	1,592,800
rs966123	1.58E-04	0.234	0.927	0.184	18	70,667,412	*ZNF407*	NM_017757.1	intron	-44,966
rs839854	1.59E-04	-0.233	0.927	0.435	1	109,273,157	*GPSM2*	NM_013296.3	intron	-689
rs10490404	1.60E-04	0.233	0.927	0.074	2	56,555,999	*EFEMP1*	NM_004105.2	flanking_5UTR	-552,317
rs2662415	1.62E-04	-0.234	0.927	0.319	5	10,178,556	---	ENST00000358163	upstream	76,065
rs7938115	1.62E-04	0.233	0.927	0.377	11	58,771,234	---	ENST00000387068	upstream	32,486
rs2306624	1.63E-04	-0.235	0.927	0.183	3	25,399,565	---	ENST00000264330	intron	0
rs12269966	1.64E-04	0.233	0.927	0.136	11	26,607,723	*TMEM16C*	NM_031418	intron	0
rs2680611	1.65E-04	-0.232	0.927	0.062	8	3,972,317	*CSMD1*	NM_033225.3	intron	-95,288
rs1805330	1.65E-04	0.232	0.927	0.100	9	109,124,082	*RAD23B*	NM_002874.3	intron	-3
rs7679437	1.66E-04	-0.232	0.927	0.125	4	66,460,278	*EPHA5*	NM_182472.1	flanking_5UTR	-242,174
rs405615	1.66E-04	-0.232	0.927	0.458	12	42,266,726	*ADAMTS20*	NM_025003.2	flanking_5UTR	-34,735
rs304115	1.66E-04	-0.233	0.927	0.160	15	99,071,080	*ASB7*	NM_198243.1	flanking_3UTR	-63,906
rs1304899	1.67E-04	-0.232	0.927	0.451	2	56,613,367	*VRK2*	NM_006296	upstream	1,513,857
rs10073259	1.67E-04	0.232	0.927	0.348	5	10,170,760	*LOC134145*	NM_199133.1	flanking_3UTR	-108,685
rs1864911	1.70E-04	-0.232	0.927	0.169	3	25,393,012	*RARB*	NM_016152.2	flanking_5UTR	-51,746
rs6863597	1.70E-04	-0.232	0.927	0.223	5	96,509,236	*LIX1*	NM_153234.3	flanking_5UTR	-5,041
rs7852390	1.70E-04	-0.238	0.927	0.445	9	14,762,384	*FREM1*	NM_144966.3	intron	-20,357
rs1920562	1.70E-04	0.232	0.927	0.215	12	113,145,041	*TBX5*	NM_000192.3	flanking_3UTR	-131,077

rs6882064	1.71E-04	0.233	0.927	0.400	5	53,729,928	*HSPB3*	NM_006308.1	flanking_5UTR	-57,274
rs12435011	1.71E-04	-0.236	0.927	0.154	14	20,595,884	*RNASE8*	NM_138331.1	flanking_5UTR	-8
rs1614387	1.72E-04	0.234	0.927	0.203	10	22,977,077	*PIP4K2A*	NM_005028	intron	0
rs10202807	1.74E-04	0.235	0.927	0.396	2	56,520,453	*EFEMP1*	NM_004105.2	flanking_5UTR	-516,771
rs7628541	1.74E-04	-0.234	0.927	0.178	3	25,397,700	*RARB*	NM_016152.2	flanking_5UTR	-47,058
rs2814033	1.77E-04	-0.233	0.927	0.240	10	63,001,600	*C10orf107*	NM_173554	upstream	91,125
rs6583476	1.78E-04	-0.233	0.927	0.202	7	48,214,960	*ABCA13*	NM_152701.2	intron	-6,448
rs17039498	1.79E-04	0.231	0.927	0.092	3	1,471,493	*CNTN6*	NM_014461	downstream	51,215
rs2710767	1.81E-04	-0.232	0.927	0.369	10	92,424,887	*HTR7*	NM_000872.3	flanking_3UTR	-65,668
rs948039	1.81E-04	0.232	0.927	0.485	11	131,407,015	*HNT*	NM_016522	intron	0
rs6101282	1.82E-04	0.231	0.927	0.076	20	59,267,601	*CDH4*	NM_001794	intron	0
rs6101283	1.82E-04	0.231	0.927	0.076	20	59,267,891	*CDH4*	NM_001794	intron	0
rs4591494	1.83E-04	0.231	0.927	0.255	3	7,957,827	*GRM7*	NM_000844.2	flanking_3UTR	-199,610
rs2857602	1.83E-04	-0.231	0.927	0.355	6	31,641,357	*LTA*	NM_000595.2	flanking_5UTR	-6,715
rs3849840	1.85E-04	-0.231	0.927	0.124	8	4,057,903	*CSMD1*	NM_033225.3	intron	-180,874
rs2787720	1.85E-04	-0.232	0.927	0.217	10	63,018,471	*C10orf107*	NM_173554.1	flanking_5UTR	-74,254
rs2488401	1.88E-04	0.230	0.927	0.217	1	195,969,024	*DENND1B*	NM_144977.1	intron	-2,315
rs3733870	1.89E-04	-0.231	0.927	0.198	5	159,411,119	*TTC1*	NM_003314.1	intron	-351
rs13188321	1.90E-04	-0.232	0.927	0.304	5	111,942,005	*FLJ11235*	XR_000545.1	flanking_3UTR	-157,437
rs6673828	1.91E-04	-0.230	0.927	0.217	1	245,205,966	*ZNF695*	NM_020394.2	intron	-8,249
rs10484447	1.91E-04	0.230	0.927	0.395	6	11,251,555	*HERV-FRD*	NM_207582.1	flanking_5UTR	-31,610
rs6591515	1.92E-04	0.230	0.927	0.391	11	58,770,684	*FAM111A*	NM_022074.2	flanking_3UTR	-91,596
rs6125878	1.92E-04	-0.230	0.927	0.304	20	48,098,281	*UBE2V1*	NM_022442.3	flanking_3UTR	-32,787
rs4939263	1.93E-04	0.232	0.927	0.391	11	58,752,972	*FAM111A*	NM_022074.2	flanking_3UTR	-73,884
rs6564074	1.93E-04	0.230	0.927	0.277	16	83,343,751	*USP10*	NM_005153.1	intron	-6,072
rs12063422	1.95E-04	-0.230	0.927	0.391	1	54,358,003	*CDCP2*	NM_201546.2	flanking_3UTR	-19,253
rs4659120	1.97E-04	-0.233	0.927	0.428	1	119,121,328	*TBX15*	NM_152380.2	flanking_3UTR	-105,861
rs16982551	1.97E-04	0.230	0.927	0.084	22	25,379,032	*CRYBA4*	NM_001886	downstream	22,400
rs752216	1.98E-04	-0.231	0.927	0.438	18	35,936,825	*PIK3C3*	NM_002647.2	flanking_5UTR	-1,852,372
rs1407440	1.99E-04	0.232	0.927	0.131	13	49,184,219	*KPNA3*	NM_002267.2	intron	-1,060
rs1539414	2.01E-04	0.232	0.927	0.206	1	196,010,129	*DENND1B*	NM_144977.1	intron	-763
rs754814	2.01E-04	-0.229	0.927	0.208	17	4,603,783	*ZMYND15*	NM_032265.1	flanking_3UTR	-7,626
rs7613165	2.02E-04	-0.230	0.927	0.293	3	2,198,722	*CNTN4*	NM_175607.1	intron	-81,399
rs17150421	2.03E-04	0.229	0.927	0.185	7	24,890,286	*OSBPL3*	NM_015550.2	intron	-8,235
rs2033015	2.04E-04	0.230	0.927	0.340	2	56,556,375	*FANCL*	NM_018062	downstream	1,683,509
rs11885335	2.05E-04	0.229	0.927	0.348	2	56,533,116	*FANCL*	NM_018062	downstream	1,706,768
rs1378884	2.05E-04	0.231	0.927	0.421	10	62,989,938	*TMEM26*	NM_178505	upstream	107,093
rs7580218	2.06E-04	0.229	0.927	0.418	2	56,612,768	*EFEMP1*	NM_004105.2	flanking_5UTR	-609,086
rs6623023	2.06E-04	0.230	0.927	0.405	X	83,560,275	*CXorf43*	NM_144657.2	intron	-21,921
rs741418	2.07E-04	-0.229	0.927	0.457	2	75,216,694	*TACR1*	NM_001058.2	intron	-15,292
rs1145223	2.08E-04	-0.229	0.927	0.127	11	117,221,663	*FXYD6*	NM_022003	intron	0
rs7332385	2.08E-04	0.230	0.927	0.422	13	49,341,419	*KPNA3*	NM_002267	upstream	76,361
rs4672140	2.09E-04	0.233	0.927	0.429	2	56,652,414	*VRK2*	ENST00000364225	downstream	214,230
rs4410626	2.09E-04	-0.229	0.927	0.075	5	81,268,600	*ATG10*	NM_031482.3	flanking_5UTR	-35,030
rs7115326	2.09E-04	-0.229	0.927	0.359	11	58,034,925	*OR5B21*	NM_001005218.1	flanking_5UTR	-2,771
rs4936152	2.09E-04	0.229	0.927	0.475	11	131,404,617	*HNT*	NM_016522	intron	0
rs12437471	2.09E-04	-0.230	0.927	0.172	15	61,075,783	*TPM1*	NM_001018005	upstream	46,108
rs17070928	2.11E-04	0.230	0.927	0.055	4	182,432,130	---	ENST00000378957	upstream	248,677
rs7176023	2.11E-04	-0.230	0.927	0.157	15	62,888,060	*RBPMS2*	NM_194272	upstream	33,221

rs11880464	2.12E-04	-0.229	0.927	0.161	19	4,946,926	*JMJD2B*	NM_015015.1	intron	-20,342
rs4331915	2.14E-04	-0.229	0.927	0.253	5	29,640,656	*CDH6*	NM_004932.2	flanking_5UTR	-1,588,897
rs10438207	2.14E-04	-0.229	0.927	0.146	14	90,633,569	*C14orf159*	NM_024952.4	flanking_5UTR	-17,228
rs11663122	2.14E-04	0.229	0.927	0.120	18	4,074,386	---	ENST00000320108	downstream	1,152,977
rs12041173	2.17E-04	0.229	0.927	0.376	1	211,999,444	*PROX1*	NM_002763.3	flanking_5UTR	-229,039
rs6718973	2.17E-04	0.230	0.927	0.360	2	56,610,361	*EFEMP1*	NM_004105.2	flanking_5UTR	-606,679
rs915378	2.18E-04	-0.228	0.927	0.380	14	100,783,663	*FLJ41170*	NM_001004332.1	flanking_3UTR	-240,541
rs1992205	2.19E-04	-0.228	0.927	0.438	8	4,671,611	*CSMD1*	NM_033225.3	intron	-167,651
rs1815083	2.20E-04	-0.228	0.927	0.225	11	86,915,659	*FLJ22104*	NM_022918.2	flanking_3UTR	-203,439
rs8045112	2.21E-04	0.228	0.927	0.404	16	9,242,819	---	ENST00000363598	downstream	322,929
rs2042703	2.21E-04	0.228	0.927	0.195	18	70,669,389	*ZNF407*	NM_017757.1	intron	-46,943
rs16827299	2.22E-04	0.228	0.927	0.103	2	147,865,430	*ACVR2A*	NM_001616.3	flanking_5UTR	-453,610
rs16827303	2.22E-04	0.228	0.927	0.103	2	147,866,499	---	ENST00000384496	upstream	15,881
rs6935468	2.22E-04	-0.230	0.927	0.352	6	67,510,138	---	ENST00000388382	downstream	723,693
rs918917	2.22E-04	-0.228	0.927	0.189	8	3,942,433	*CSMD1*	NM_033225.3	intron	-65,404
rs1355313	2.23E-04	0.228	0.927	0.266	7	118,009,548	*ANKRD7*	NM_019644.1	flanking_3UTR	-339,575
rs1156672	2.24E-04	-0.228	0.927	0.420	2	56,624,352	*EFEMP1*	NM_004105.2	flanking_5UTR	-620,670
rs1885016	2.24E-04	-0.228	0.927	0.098	6	86,039,298	*NT5E*	NM_002526.1	flanking_5UTR	-177,230
rs2893321	2.24E-04	-0.228	0.927	0.278	13	107,741,035	*TNFSF13B*	NM_006573.3	intron	-3,827
rs34001047	2.24E-04	0.228	0.927	0.054	22	32,526,508	*LARGE*	NM_004737	intron	0
rs139568	2.24E-04	-0.228	0.927	0.422	22	40,540,931	*FLJ22349*	NM_024821.2	intron	-1,159
rs17398246	2.25E-04	0.228	0.927	0.100	2	152,690,073	*STAM2*	NM_005843.3	intron	-913
rs4778652	2.25E-04	-0.228	0.927	0.188	15	79,894,158	*MEX3B*	NM_032246	downstream	227,025
rs12489351	2.26E-04	0.228	0.927	0.069	3	1,470,993	*CNTN6*	NM_014461	downstream	50,715
rs16943614	2.26E-04	0.229	0.927	0.234	18	23,255,109	*CHST9*	NM_031422	upstream	235,830
rs11028449	2.27E-04	-0.230	0.927	0.094	11	25,114,085	*LUZP2*	NM_001009909	downstream	57,305
rs7605943	2.28E-04	-0.228	0.927	0.471	2	56,595,255	*FANCL*	NM_018062	downstream	1,644,629
rs1825924	2.28E-04	-0.228	0.927	0.328	3	7,937,284	*GRM7*	NM_000844.2	flanking_3UTR	-179,067
rs10484047	2.28E-04	-0.228	0.927	0.165	14	94,118,981	*SERPINA5*	NM_000624	upstream	4,472
rs2055713	2.29E-04	-0.233	0.927	0.290	15	78,896,649	*KIAA1199*	NM_018689.1	intron	-37,639
rs7818523	2.30E-04	-0.227	0.927	0.201	8	123,616,329	*ZHX2*	NM_014943.3	flanking_5UTR	-246,753
rs2830448	2.31E-04	-0.227	0.927	0.462	21	27,035,051	*ADAMTS1*	NM_006988	downstream	95,428
rs890099	2.32E-04	-0.227	0.927	0.493	2	56,587,574	*FANCL*	NM_018062	downstream	1,652,310
rs2229463	2.32E-04	-0.231	0.927	0.306	14	23,788,355	*TGM1*	NM_000359.1	UTR	[89/3]
rs4900000	2.33E-04	0.227	0.927	0.255	14	89,044,335	*CHES1*	NM_005197.2	flanking_5UTR	-91,208
rs7885520	2.33E-04	-0.228	0.927	0.413	X	7,468,596	*VCX*	NM_013452	upstream	301,707
rs316942	2.34E-04	0.227	0.927	0.252	1	104,892,261	*AMY1C*	NM_001008219.1	flanking_3UTR	-789,428
rs10107533	2.35E-04	0.229	0.927	0.121	8	19,423,701	*ChGn*	NM_018371.3	intron	-15,780
rs12917044	2.35E-04	-0.228	0.927	0.204	15	75,637,201	*LINGO1*	NM_032808	downstream	56,240
rs13340578	2.37E-04	-0.228	0.927	0.188	8	3,940,374	---	ENST00000387211	downstream	394,873
rs12586263	2.37E-04	-0.227	0.927	0.111	14	79,259,127	*NRXN3*	NM_004796.3	intron	-25,094
rs2314293	2.38E-04	0.230	0.927	0.388	16	9,244,119	---	ENST00000333457	upstream	85,880
rs1173657	2.40E-04	-0.227	0.927	0.207	1	243,692,662	---	ENST00000329504	downstream	91,022
rs1173658	2.40E-04	-0.227	0.927	0.207	1	243,693,543	---	ENST00000294926	upstream	47,384
rs6100868	2.40E-04	-0.227	0.927	0.072	20	58,341,859	*LOC284757*	NM_001004305.1	flanking_3UTR	-8,867
rs2515366	2.41E-04	-0.227	0.927	0.424	11	58,030,946	*OR5B21*	NM_001005218.1	flanking_3UTR	-279
rs397461	2.42E-04	-0.227	0.927	0.399	11	113,592,677	*ZBTB16*	NM_001018011.1	intron	-25,422
rs7952094	2.44E-04	-0.227	0.927	0.375	11	58,043,895	*LPXN*	NM_004811.1	flanking_3UTR	-7,025
rs6460910	2.45E-04	0.226	0.927	0.306	7	12,276,970	---	ENST00000275358	downstream	60,066

rs4820848	2.45E-04	0.227	0.927	0.274	22	29,173,495	*SEC14L3*	NM_174975.3	flanking_3UTR	-11,724
rs2227023	2.46E-04	0.226	0.927	0.078	22	32,543,356	*LARGE*	NM_004737.3	intron	-39,372
rs10737170	2.47E-04	-0.226	0.927	0.085	1	154,330,504	*LMNA*	NM_170708.1	flanking_5UTR	-20,618
rs2081326	2.50E-04	-0.227	0.927	0.250	2	186,046,519	*ZNF804A*	NM_194250	downstream	534,521
rs11175792	2.50E-04	0.226	0.927	0.284	12	64,191,935	*MSRB3*	NM_198080	downstream	44,990
rs1448378	2.51E-04	0.226	0.927	0.395	1	104,893,219	---	ENST00000332554	downstream	790,416
rs4672130	2.51E-04	-0.226	0.927	0.435	2	56,615,502	*EFEMP1*	NM_004105.2	flanking_5UTR	-611,820
rs285001	2.51E-04	0.226	0.927	0.275	16	75,670,065	*CNTNAP4*	NM_033401	downstream	519,604
rs17109039	2.53E-04	0.228	0.927	0.061	1	82,993,840	*LPHN2*	NM_012302	downstream	763,145
rs17268931	2.53E-04	-0.226	0.927	0.471	2	56,603,713	*VRK2*	NM_006296	upstream	1,523,511
rs2686534	2.53E-04	0.226	0.927	0.437	3	5,500,968	*EDEM1*	NM_014674.1	flanking_3UTR	-264,326
rs1156972	2.54E-04	0.229	0.927	0.430	2	56,619,139	*VRK2*	NM_006296	upstream	1,508,085
rs484096	2.54E-04	-0.226	0.927	0.424	11	131,417,990	*HNT*	NM_016522.2	intron	-103,396
rs11125642	2.55E-04	-0.226	0.927	0.471	2	56,667,967	*EFEMP1*	NM_004105.2	flanking_5UTR	-664,285
rs3847173	2.55E-04	0.228	0.927	0.118	8	133,992,096	*TG*	NM_003235	intron	0
rs7158292	2.56E-04	-0.226	0.927	0.114	14	79,260,309	*NRXN3*	NM_004796	intron	0
rs17075630	2.57E-04	-0.226	0.927	0.156	3	43,392,499	*TMEM16K*	NM_018075.2	intron	-9,029
rs4558307	2.57E-04	-0.227	0.927	0.055	13	40,856,476	*NARG1L*	NM_024561.3	flanking_3UTR	-7,314
rs2199226	2.59E-04	0.227	0.927	0.090	4	167,516,159	*TLL1*	NM_012464.3	flanking_3UTR	-271,716
rs17160491	2.60E-04	-0.226	0.927	0.299	19	8,418,940	*HNRPM*	NM_005968.2	intron	-2,945
rs4257593	2.61E-04	-0.231	0.927	0.312	3	98,925,740	---	ENST00000389672	intron	0
rs9964613	2.61E-04	-0.225	0.927	0.203	18	46,221,164	*C18orf24*	NM_145060.1	flanking_3UTR	-46,628
rs2162025	2.62E-04	-0.225	0.927	0.469	2	56,599,900	*VRK2*	NM_006296	upstream	1,527,324
rs17048168	2.62E-04	-0.226	0.927	0.432	2	56,646,010	*EFEMP1*	NM_004105.2	flanking_5UTR	-642,328
rs17620029	2.63E-04	-0.226	0.927	0.135	19	24,146,152	*ZNF539*	NM_203282.1	flanking_3UTR	-41,659
rs879807	2.64E-04	0.226	0.927	0.355	2	56,562,449	*EFEMP1*	NM_004105.2	flanking_5UTR	-558,767
rs7109553	2.64E-04	-0.226	0.927	0.375	11	58,066,490	*LPXN*	NM_004811.1	intron	-7,345
rs6561362	2.64E-04	0.225	0.927	0.328	13	46,724,160	*HTR2A*	NM_000621.2	flanking_5UTR	-355,984
rs13340565	2.65E-04	-0.225	0.927	0.192	8	3,939,796	---	ENST00000387211	downstream	394,295
rs12909385	2.65E-04	0.225	0.927	0.183	15	55,484,367	*CGNL1*	NM_032866.3	intron	-28,310
rs1523921	2.66E-04	-0.225	0.927	0.216	2	225,017,222	*CUL3*	NM_003590.2	flanking_3UTR	-25,891
rs17068975	2.66E-04	-0.225	0.927	0.178	8	3,944,590	---	ENST00000387211	downstream	399,089
rs880506	2.67E-04	0.225	0.927	0.250	2	111,343,771	*ACOXL*	NM_018308.1	intron	-28,318
rs9308691	2.67E-04	0.225	0.927	0.250	2	111,357,609	*ACOXL*	NM_018308.1	intron	-25,228
rs17720559	2.67E-04	-0.226	0.927	0.222	6	125,329,260	*IBRDC1*	NM_152553.2	flanking_5UTR	-16,953
rs10188079	2.69E-04	0.226	0.927	0.248	2	111,360,946	*ACOXL*	NM_018308.1	intron	-21,891
rs28403575	2.69E-04	0.226	0.927	0.121	14	23,095,180	*THTPA*	NM_024328.2	5UTR	[109/626]
rs1298527	2.70E-04	-0.226	0.927	0.346	18	13,820,371	*MC2R*	NM_000529	downstream	51,672
rs1452912	2.73E-04	0.225	0.927	0.369	7	134,158,627	*CALD1*	NM_033157.2	intron	-20,548
rs3027213	2.74E-04	0.225	0.927	0.424	17	7,956,738	*ALOXE3*	NM_021628	intron	0
rs10920357	2.75E-04	-0.227	0.927	0.281	1	200,418,189	*PTPN7*	ENST00000334512	intron	0
rs7553985	2.77E-04	-0.227	0.927	0.467	1	104,908,021	*AMY1C*	NM_001008219.1	flanking_3UTR	-805,188
rs779130	2.77E-04	-0.225	0.927	0.116	8	4,036,782	---	ENST00000387211	downstream	491,281
rs4639316	2.78E-04	0.224	0.927	0.257	6	67,459,002	---	ENST00000364559	downstream	627,187
rs9381017	2.79E-04	-0.224	0.927	0.219	6	41,057,911	*LRFN2*	NM_020737	upstream	394,807
rs10500223	2.80E-04	-0.226	0.927	0.128	19	24,137,864	*ZNF539*	NM_203282.1	flanking_3UTR	-33,371
rs12589656	2.81E-04	-0.224	0.927	0.143	14	79,262,615	*NRXN3*	NM_004796.3	intron	-28,582
rs677903	2.81E-04	0.224	0.927	0.232	19	15,660,366	*CYP4F12*	NM_023944	intron	0
rs13397156	2.82E-04	0.225	0.927	0.453	2	56,536,051	*EFEMP1*	NM_004105.2	flanking_5UTR	-532,369

rs17286565	2.82E-04	0.224	0.927	0.060	16	82,003,805	*CDH13*	NM_001257	intron	0
rs10820123	2.83E-04	0.224	0.927	0.236	9	104,085,547	---	ENST00000374803	downstream	207,549
rs9876387	2.84E-04	-0.224	0.927	0.181	3	188,116,768	*ST6GAL1*	NM_173217.1	flanking_5UTR	-14,442
rs4649384	2.87E-04	-0.224	0.927	0.469	1	230,460,886	---	ENST00000366656	intron	0
rs2670610	2.87E-04	0.224	0.927	0.103	2	124,146,012	*CNTNAP5*	NM_138996.1	flanking_5UTR	-353,322
rs12537903	2.87E-04	0.224	0.927	0.366	7	34,395,753	*AAA1*	NM_207287.1	intron	-27,963
rs2844484	2.88E-04	-0.225	0.927	0.358	6	31,644,203	*LTA*	NM_000595.2	flanking_5UTR	-3,869
rs10232196	2.88E-04	-0.224	0.927	0.344	7	12,304,074	---	ENST00000275358	downstream	32,962
rs890564	2.88E-04	-0.224	0.927	0.364	8	112,233,494	---	ENST00000385958	upstream	217,143
rs7113375	2.88E-04	0.225	0.927	0.223	11	18,224,573	*SAA2*	NM_030754	intron	0
rs8022660	2.88E-04	-0.225	0.927	0.235	14	21,605,102	*GeneID:28661*	-	flanking_5UTR	-3,691
rs698611	2.88E-04	0.224	0.927	0.263	18	44,782,268	*DYM*	NM_017653.2	flanking_3UTR	-41,902
rs5985950	2.90E-04	0.224	0.927	0.377	X	28,997,642	*IL1RAPL1*	NM_014271	intron	0
rs7788803	2.91E-04	0.224	0.927	0.239	7	144,341,453	*TPK1*	NM_022445.2	flanking_5UTR	-177,387
rs7814130	2.91E-04	0.224	0.927	0.495	8	70,717,243	*SULF1*	NM_015170.1	intron	-1,613
rs11623535	2.91E-04	-0.224	0.927	0.301	14	71,532,134	*RGS6*	NM_004296	intron	0
rs6802154	2.92E-04	0.224	0.927	0.136	3	134,902,495	*TOPBP1*	NM_007027.2	flanking_5UTR	-39,115
rs2120295	2.92E-04	-0.224	0.927	0.219	12	52,639,103	*HOXC12*	NM_173860.1	flanking_3UTR	-2,486
rs9535310	2.92E-04	0.224	0.927	0.139	13	49,194,027	*KPNA3*	NM_002267.2	intron	-65
rs5968890	2.92E-04	0.224	0.927	0.134	X	85,463,916	*DACH2*	NM_053281	intron	0
rs7499959	2.95E-04	0.225	0.927	0.229	16	86,667,923	*BANP*	NM_079837	3UTR	0
rs1421106	2.97E-04	-0.223	0.927	0.176	12	14,017,001	*GRIN2B*	NM_000834.2	intron	-7,128
rs12429115	2.98E-04	-0.224	0.927	0.182	13	37,494,233	*TRPC4*	NM_016179	upstream	152,294
rs4893555	2.98E-04	-0.223	0.927	0.303	X	28,782,000	*IL1RAPL1*	NM_014271	intron	0
rs4893599	2.98E-04	-0.223	0.927	0.303	X	28,782,529	*IL1RAPL1*	ENST00000300057	upstream	10,101
rs2130835	3.00E-04	-0.224	0.927	0.418	X	7,480,499	*STS*	NM_000351.3	flanking_3UTR	-197,817
rs1944054	3.01E-04	-0.224	0.927	0.434	11	58,088,500	*LPXN*	NM_004811	intron	0
rs2830987	3.02E-04	0.225	0.927	0.245	21	27,798,286	---	ENST00000324988	upstream	509,286
rs737030	3.03E-04	0.223	0.927	0.377	2	56,697,109	*EFEMP1*	NM_004105.2	flanking_5UTR	-693,427
rs13148238	3.03E-04	-0.223	0.927	0.051	4	144,067,928	*USP38*	NM_032557	upstream	257,620
rs2254096	3.03E-04	-0.223	0.927	0.210	10	62,978,165	*TMEM26*	NM_178505.4	flanking_5UTR	-94,951
rs17038122	3.04E-04	0.224	0.927	0.476	2	56,482,597	*EFEMP1*	NM_004105.2	flanking_5UTR	-478,915
rs1876152	3.04E-04	0.224	0.927	0.458	5	10,166,778	*LOC134145*	NM_199133.1	flanking_3UTR	-112,667
rs7724279	3.04E-04	-0.224	0.927	0.292	5	31,993,642	*PDZK3*	NM_015022.2	intron	-25,376
rs1990393	3.04E-04	0.225	0.927	0.373	7	123,641,931	*SPAM1*	NM_003117.3	flanking_3UTR	-243,231
rs9633568	3.05E-04	0.224	0.927	0.382	10	67,163,419	*LRRTM3*	NM_178011	upstream	1,192,400
rs2160954	3.06E-04	-0.223	0.927	0.357	3	7,861,502	*GRM7*	NM_181875	downstream	153,596
rs928815	3.07E-04	-0.223	0.927	0.355	6	31,639,194	*NFKBIL1*	NM_005007.2	flanking_3UTR	-4,609
rs6101284	3.08E-04	0.223	0.927	0.075	20	59,269,784	*CDH4*	NM_001794.2	intron	-6,396
rs17160495	3.09E-04	-0.223	0.927	0.303	19	8,422,278	*HNRPM*	NM_005968	intron	0
rs5996039	3.11E-04	-0.223	0.927	0.252	22	40,311,903	*PMM1*	NM_002676.1	intron	-123
rs8139993	3.11E-04	-0.223	0.927	0.252	22	40,325,281	*D15Wsu75e*	NM_015704.1	flanking_3UTR	-1,601
rs2443780	3.12E-04	-0.224	0.927	0.201	8	109,113,763	*RSPO2*	NM_178565.3	intron	-43,115
rs2528630	3.13E-04	-0.223	0.927	0.054	2	159,294,834	---	ENST00000342892	intron	0
rs17190394	3.16E-04	0.222	0.927	0.074	2	56,624,834	*EFEMP1*	NM_004105.2	flanking_5UTR	-621,152
rs7998555	3.16E-04	0.224	0.927	0.318	13	46,727,135	---	ENST00000386918	upstream	297,697
rs759394	3.17E-04	-0.223	0.927	0.398	12	94,983,473	*LTA4H*	NM_000895.1	flanking_5UTR	-29,977
rs17162040	3.18E-04	0.222	0.927	0.103	1	27,755,814	*AHDC1*	NM_001029882.1	intron	-1,990
rs9543882	3.18E-04	-0.223	0.927	0.182	13	74,733,860	*TBC1D4*	NM_014832.1	flanking_3UTR	-23,394

rs17098542	3.18E-04	-0.222	0.927	0.241	14	61,028,043	*PRKCH*	NM_006255.3	intron	-5,916
rs9888482	3.19E-04	0.229	0.927	0.071	13	37,826,976	*UFM1*	NM_016617.1	intron	-543
rs11653737	3.19E-04	-0.222	0.927	0.172	17	14,545,494	*FLJ45831*	NM_001001684.1	flanking_3UTR	-66,131
rs17619763	3.20E-04	0.222	0.927	0.062	12	109,130,212	*IFT81*	NM_014055.2	intron	-1,147
rs17187412	3.20E-04	0.222	0.927	0.062	12	109,251,150	*ATP2A2*	NM_001681.2	intron	-945
rs1057452	3.20E-04	0.223	0.927	0.124	16	29,741,215	*MVP*	NM_005115.3	intron	-1,883
rs11900604	3.22E-04	-0.222	0.927	0.138	2	79,569,387	*REG3A*	NM_138937	upstream	329,000
rs2467440	3.22E-04	0.223	0.927	0.196	16	1,049,436	*FLJ32252*	NM_182510	downstream	5,864
rs13324989	3.25E-04	-0.222	0.927	0.328	3	2,205,964	*CNTN4*	NM_175607.1	intron	-88,641
rs980135	3.25E-04	-0.222	0.927	0.161	10	27,948,130	*RAB18*	NM_021252	downstream	79,032
rs12752688	3.26E-04	-0.222	0.927	0.096	1	169,418,262	*FMO2*	NM_001460.2	flanking_5UTR	-2,750
rs11184211	3.27E-04	-0.222	0.927	0.465	1	104,916,711	---	ENST00000332554	downstream	813,908
rs12656418	3.27E-04	-0.227	0.927	0.262	5	160,122,710	*PTTG1*	NM_004219.2	flanking_3UTR	-334,386
rs4822050	3.28E-04	-0.222	0.927	0.320	22	40,507,399	*RP5-821D11.2*	NM_152513	intron	0
rs7097617	3.30E-04	-0.222	0.927	0.473	10	77,174,293	*C10orf11*	NM_032024.2	flanking_5UTR	-38,232
rs12592342	3.30E-04	0.222	0.927	0.447	15	29,536,628	*OTUD7A*	NM_130901	downstream	25,993
rs11247163	3.33E-04	0.222	0.927	0.344	15	96,592,516	*FLJ39743*	NM_182562.1	flanking_3UTR	-205,398
rs28655569	3.33E-04	0.222	0.927	0.263	17	1,092,256	*TUSC5*	NM_172367.1	flanking_5UTR	-37,451
rs17025957	3.34E-04	0.222	0.927	0.168	1	110,863,572	*KCNA10*	NM_005549.2	flanking_5UTR	-252
rs1927432	3.34E-04	0.222	0.927	0.146	6	167,121,023	*RPS6KA2*	NM_001006932.1	intron	-70,655
rs17791484	3.35E-04	-0.222	0.927	0.135	10	61,282,165	*CCDC6*	NM_005436.2	intron	-152
rs932680	3.36E-04	0.222	0.927	0.350	6	44,609,851	*RUNX2*	ENST00000363368	downstream	362,570
rs7127915	3.37E-04	0.225	0.927	0.114	11	26,609,538	*TMEM16C*	NM_031418	intron	0
rs16961281	3.37E-04	0.221	0.927	0.089	13	102,516,825	*SLC10A2*	NM_000452.1	5UTR	[224/372]
rs1060700	3.38E-04	0.222	0.927	0.407	7	12,242,343	*TMEM106B*	NM_018374.2	3UTR	[4216/1068]
rs10896791	3.38E-04	-0.221	0.927	0.377	11	58,083,371	*LPXN*	NM_004811	intron	0
rs11807023	3.41E-04	0.221	0.927	0.060	1	82,998,367	*LPHN2*	NM_012302.2	flanking_3UTR	-767,672
rs12658195	3.41E-04	-0.222	0.927	0.220	5	88,907,501	*MEF2C*	NM_002397.2	flanking_5UTR	-692,721
rs4147994	3.41E-04	0.221	0.927	0.165	17	64,414,831	*ABCA8*	NM_007168.2	intron	-645
rs2691462	3.42E-04	-0.221	0.927	0.063	1	57,727,004	*DAB1*	NM_021080	intron	0
rs7636197	3.43E-04	0.222	0.927	0.153	3	2,876,065	*CNTN4*	NM_175612	intron	0
rs2140016	3.43E-04	-0.222	0.927	0.435	8	84,237,668	*SNX16*	NM_022133	upstream	1,320,678
rs11012068	3.43E-04	0.222	0.927	0.058	10	20,803,311	*NEBL*	NM_213569	downstream	309,530
rs10474519	3.45E-04	-0.222	0.927	0.127	5	76,929,703	*OTP*	NM_032109.2	flanking_3UTR	-30,590
rs2153252	3.45E-04	0.221	0.927	0.223	6	149,407,757	*UST*	NM_005715.1	intron	-23,447
rs2238275	3.45E-04	0.222	0.927	0.140	14	71,527,118	*RGS6*	NM_004296.3	intron	-25,773
rs9295808	3.50E-04	0.221	0.927	0.060	6	11,290,065	*HERV-FRD*	NM_207582	upstream	76,536
rs1015055	3.50E-04	-0.221	0.927	0.427	11	131,438,551	*HNT*	NM_016522.2	intron	-82,835
rs4671281	3.51E-04	0.221	0.927	0.420	2	56,481,805	*FANCL*	NM_018062	downstream	1,758,079
rs12917431	3.51E-04	-0.221	0.927	0.178	15	75,637,239	*HMG20A*	NM_018200	downstream	72,285
rs653488	3.51E-04	0.221	0.927	0.486	19	7,746,356	*CLEC4M*	NM_214677	downstream	5,866
rs597012	3.51E-04	0.222	0.927	0.227	19	15,661,729	*CYP4F12*	NM_023944	intron	0
rs16982543	3.51E-04	0.221	0.927	0.101	22	25,378,858	*CRYBA4*	NM_001886	downstream	22,226
rs7616053	3.52E-04	-0.221	0.927	0.047	3	128,417,127	*C3orf56*	NM_001007534.1	flanking_3UTR	-17,412
rs12201073	3.52E-04	0.223	0.927	0.292	6	67,545,323	*LOC442229*	XR_000273.2	flanking_3UTR	-989,230
rs3739517	3.53E-04	0.222	0.927	0.062	9	78,306,575	*GCNT1*	NM_001490.3	intron	-400
rs12691592	3.54E-04	-0.221	0.927	0.355	2	141,594,971	*LRP1B*	NM_018557	intron	0
rs10519535	3.54E-04	0.222	0.927	0.157	4	141,469,112	*SCOC*	NM_032547.1	intron	-15,113
rs12067920	3.55E-04	0.222	0.927	0.099	1	95,152,346	*CNN3*	NM_001839.2	intron	-10,678

rs10280443	3.55E-04	-0.221	0.927	0.321	7	12,356,711	*TMEM106B*	NM_018374.2	flanking_3UTR	-113,300
rs4921659	3.55E-04	0.221	0.927	0.130	8	19,444,298	*ChGn*	NM_018371.3	intron	-36,377
rs943196	3.56E-04	0.221	0.927	0.202	10	22,968,520	*PIP5K2A*	NM_005028.3	intron	-29,868
rs7910342	3.56E-04	-0.221	0.927	0.406	10	131,692,523	*EBF3*	NM_001005463.1	flanking_5UTR	-40,442
rs1465360	3.57E-04	0.221	0.927	0.418	2	56,541,768	*EFEMP1*	NM_004105.2	flanking_5UTR	-538,086
rs4896606	3.57E-04	-0.221	0.927	0.237	6	125,310,084	*IBRDC1*	NM_152553.2	flanking_5UTR	-36,129
rs1078446	3.58E-04	0.221	0.927	0.366	19	55,495,215	*MYH14*	NM_024729.2	intron	-1,515
rs2238276	3.59E-04	0.220	0.927	0.136	14	71,525,980	*RGS6*	NM_004296.3	intron	-24,635
rs3767955	3.60E-04	-0.220	0.927	0.288	1	40,877,062	*RIMS3*	NM_014747.2	intron	-2,746
rs10074734	3.60E-04	0.222	0.927	0.291	5	29,632,509	---	ENST00000365278	upstream	352,183
rs4931395	3.65E-04	0.220	0.927	0.181	12	31,019,283	*DDX11*	NM_030655.2	flanking_5UTR	-98,794
rs4414921	3.66E-04	-0.223	0.927	0.122	4	90,719,856	*GPRIN3*	NM_198281	upstream	271,889
rs1540231	3.66E-04	0.220	0.927	0.400	11	130,699,898	*HNT*	NM_016522	upstream	586,209
rs1243500	3.66E-04	-0.220	0.927	0.100	14	94,382,413	*GSC*	NM_173849.2	flanking_5UTR	-76,161
rs9319558	3.66E-04	-0.220	0.927	0.203	16	79,509,800	*CDYL2*	NM_152342	upstream	114,124
rs354064	3.67E-04	-0.221	0.927	0.166	7	148,950,372	*ZNF767*	NM_024910	intron	0
rs16962755	3.67E-04	0.220	0.927	0.129	15	47,777,501	*ATP8B4*	NM_024837	downstream	160,226
rs2084447	3.68E-04	-0.221	0.927	0.467	1	104,903,837	*AMY1C*	NM_001008219.1	flanking_3UTR	-801,004
rs7834743	3.68E-04	0.220	0.927	0.082	8	89,380,901	*MMP16*	NM_005941.2	intron	-27,519
rs1409571	3.69E-04	-0.224	0.927	0.363	13	71,570,466	---	ENST00000363167	downstream	120,121
rs17690152	3.72E-04	-0.221	0.927	0.082	13	101,863,063	*TPP2*	NM_003291	upstream	184,224
rs39731	3.73E-04	-0.220	0.927	0.098	7	78,268,162	*MAGI2*	NM_012301.3	intron	-173,671
rs10103231	3.74E-04	0.221	0.927	0.361	8	60,575,627	*CA8*	NM_004056	downstream	688,350
rs10869613	3.74E-04	0.222	0.927	0.210	9	77,440,080	*PCSK5*	NM_006200.2	flanking_5UTR	-255,361
rs4783057	3.74E-04	0.220	0.927	0.277	16	83,343,173	*USP10*	ENST00000378510	intron	0
rs631193	3.74E-04	0.221	0.927	0.229	19	15,659,394	*CYP4F12*	NM_023944	intron	0
rs1625309	3.75E-04	-0.220	0.927	0.056	8	4,027,866	*CSMD1*	NM_033225.3	intron	-150,837
rs825851	3.75E-04	0.220	0.927	0.335	16	72,093,722	*C16orf47*	NM_207385.1	flanking_5UTR	-357,875
rs9854973	3.77E-04	0.220	0.927	0.426	3	11,702,998	*VGLL4*	NM_014667	intron	0
rs9625870	3.78E-04	0.220	0.927	0.338	22	28,591,219	*MTMR3*	NM_021090.2	flanking_5UTR	-17,980
rs851848	3.80E-04	-0.220	0.927	0.330	3	15,292,942	*SH3BP5*	NM_001018009.1	intron	-6,554
rs9359725	3.80E-04	-0.220	0.927	0.138	6	87,810,036	*HTR1E*	NM_000865.1	flanking_3UTR	-26,920
rs1477521	3.81E-04	-0.220	0.927	0.473	2	56,607,983	*EFEMP1*	NM_004105.2	flanking_5UTR	-604,301
rs10060606	3.81E-04	0.220	0.927	0.457	5	10,168,751	*LOC134145*	NM_199133.1	flanking_3UTR	-110,694
rs9292313	3.82E-04	0.220	0.927	0.071	5	18,749,589	---	ENST00000387352	downstream	166,118
rs4107954	3.83E-04	0.220	0.927	0.360	3	133,075,386	*CPNE4*	NM_130808.1	intron	-31,412
rs2345838	3.83E-04	0.220	0.927	0.218	14	56,528,889	*OTX2*	NM_021728.2	flanking_5UTR	-181,952
rs7034075	3.84E-04	-0.220	0.927	0.241	9	27,982,871	*LINGO2*	NM_152570	intron	0
rs3852585	3.84E-04	-0.219	0.927	0.101	12	118,998,008	*CCDC64*	NM_207311.1	intron	-1,282
rs1808448	3.84E-04	0.219	0.927	0.051	16	87,521,081	*CBFA2T3*	NM_175931.1	intron	-13,848
rs2830991	3.84E-04	0.219	0.927	0.255	21	27,808,439	---	ENST00000324988	upstream	499,133
rs17048179	3.85E-04	-0.219	0.927	0.428	2	56,651,902	*VRK2*	NM_006296	upstream	1,475,322
rs335200	3.85E-04	0.219	0.927	0.069	5	122,538,028	*PPIC*	NM_000943.4	flanking_5UTR	-137,704
rs11629240	3.85E-04	-0.219	0.927	0.089	14	90,332,912	*TTC7B*	NM_001010854	intron	0
rs1790171	3.86E-04	0.219	0.927	0.453	11	131,440,446	*HNT*	ENST00000374791	intron	0
rs4640778	3.88E-04	0.220	0.927	0.136	5	158,963,951	*IL12B*	NM_002187.2	flanking_5UTR	-273,892
rs4354860	3.88E-04	-0.219	0.927	0.310	14	71,533,860	*RGS6*	NM_004296	intron	0
rs4899412	3.88E-04	-0.219	0.927	0.310	14	71,534,015	*RGS6*	NM_004296.3	intron	-32,670
rs10275499	3.90E-04	0.220	0.927	0.277	7	24,392,004	*NPY*	NM_000905.2	flanking_3UTR	-94,002

rs2978141	3.90E-04	0.219	0.927	0.493	8	95,116,371	*PPM2C*	NM_018444	downstream	108,901
rs1381074	3.91E-04	0.219	0.927	0.473	3	133,103,778	*CPNE4*	NM_130808	intron	0
rs5943559	3.91E-04	-0.219	0.927	0.335	X	28,763,125	*IL1RAPL1*	NM_014271.2	intron	-45,662
rs1381073	3.92E-04	0.220	0.927	0.475	3	133,103,999	*CPNE4*	NM_130808.1	intron	-2,799
rs2830992	3.92E-04	0.219	0.927	0.264	21	27,810,551	*C21orf94*	NM_145180.2	flanking_5UTR	-497,021
rs3008654	3.93E-04	0.219	0.927	0.087	1	220,833,232	*TAF1A*	NM_139352.1	flanking_5UTR	-3,354
rs3002130	3.93E-04	0.219	0.927	0.087	1	220,838,762	*TAF1A*	ENST00000366890	upstream	9,012
rs12441329	3.94E-04	-0.219	0.927	0.196	15	29,164,865	*TRPM1*	NM_002420.3	intron	-8,386
rs7637849	3.95E-04	-0.219	0.927	0.082	3	38,634,279	*SCN5A*	NM_198056.1	intron	-3,059
rs9900319	3.95E-04	-0.219	0.927	0.245	17	73,990,962	*DNAH17*	NM_003727.1	flanking_5UTR	-16,498
rs11878982	3.97E-04	-0.219	0.927	0.114	19	4,992,049	*JMJD2B*	NM_015015.1	intron	-99
rs6841569	3.98E-04	0.219	0.927	0.103	4	125,886,169	*ANKRD50*	NM_020337.1	flanking_5UTR	-34,787
rs12501856	4.00E-04	0.219	0.927	0.225	4	25,268,706	*SLC34A2*	NM_006424.1	intron	-2,096
rs11113808	4.00E-04	-0.219	0.927	0.147	12	107,220,980	*CMKLR1*	NM_004072.1	intron	-10,114
rs6073190	4.01E-04	0.219	0.927	0.210	20	41,824,387	*FAM112A*	NM_001008901.1	flanking_5UTR	-35,331
rs4681053	4.02E-04	-0.222	0.927	0.166	3	25,393,650	*RARB*	NM_016152.2	flanking_5UTR	-51,108
rs13023754	4.04E-04	-0.219	0.927	0.167	2	28,589,502	*PLB1*	NM_153021.3	intron	-3,677
rs17658458	4.04E-04	0.219	0.927	0.315	4	176,917,669	*GPM6A*	NM_005277	intron	0
rs10869918	4.05E-04	0.219	0.927	0.149	9	70,935,127	*TJP2*	NM_201629.1	flanking_5UTR	-43,782
rs2839398	4.06E-04	-0.219	0.927	0.219	21	42,159,421	*PRDM15*	NM_022115	intron	0
rs10246939	4.08E-04	0.219	0.927	0.444	7	141,319,073	*TAS2R38*	NM_176817.1	coding	[116/885]
rs7980519	4.08E-04	0.220	0.927	0.073	12	115,826,548	*HRK*	NM_003806	upstream	22,933
rs17576350	4.09E-04	-0.218	0.927	0.141	4	164,665,964	*FLJ11184*	NM_018352	downstream	4,857
rs9468829	4.09E-04	0.219	0.927	0.180	6	30,857,212	*IER3*	NM_052815.1	flanking_5UTR	-36,909
rs17066062	4.09E-04	-0.220	0.927	0.077	8	3,259,949	*CSMD1*	NM_033225	upstream	5,413
rs2130017	4.09E-04	0.224	0.927	0.119	11	89,142,024	*TRIM49*	NM_020358.2	flanking_3UTR	-28,447
rs2274230	4.11E-04	-0.218	0.927	0.056	1	177,350,703	*ABL2*	NM_005158.2	coding	[67/85]
rs9724754	4.11E-04	-0.218	0.927	0.056	1	177,351,031	*ABL2*	NM_005158.2	intron	-243
rs17277358	4.11E-04	-0.218	0.927	0.056	1	177,367,333	*ABL2*	NM_005158.2	intron	-94
rs3753658	4.11E-04	-0.219	0.927	0.119	1	224,079,309	*EPHX1*	NM_000120.2	flanking_5UTR	-290
rs2650731	4.11E-04	-0.218	0.927	0.212	10	63,009,661	---	ENST00000389639	intron	0
rs2606104	4.11E-04	-0.218	0.927	0.212	10	63,012,867	*C10orf107*	NM_173554.1	flanking_5UTR	-79,858
rs2606117	4.11E-04	-0.218	0.927	0.212	10	63,021,809	---	ENST00000389639	intron	0
rs753246	4.12E-04	-0.219	0.927	0.209	10	63,012,493	*C10orf107*	NM_173554.1	flanking_5UTR	-80,232
rs9316474	4.12E-04	0.220	0.927	0.141	13	49,211,346	*KPNA3*	NM_002267.2	intron	-6,163
rs7166999	4.12E-04	0.218	0.927	0.473	15	97,110,328	*IGF1R*	NM_000875.2	intron	-41,469
rs4469455	4.13E-04	0.219	0.927	0.458	8	84,207,495	*SNX16*	NM_152837	upstream	1,290,505
rs1790181	4.13E-04	-0.218	0.927	0.415	11	131,459,357	*HNT*	NM_016522	intron	0
rs10198586	4.14E-04	-0.218	0.927	0.107	2	58,935,942	*FANCL*	NM_018062.2	flanking_5UTR	-613,924
rs962115	4.15E-04	-0.218	0.927	0.141	3	120,082,765	*IGSF11*	NM_001015887	downstream	20,926
rs768654	4.15E-04	-0.218	0.927	0.152	10	20,331,119	*PLXDC2*	NM_032812	intron	0
rs5934006	4.15E-04	0.219	0.927	0.093	X	12,407,177	*FRMPD4*	NM_014728.1	intron	-19,543
rs6843038	4.17E-04	0.219	0.927	0.365	4	182,162,059	*MGC45800*	NM_178838.3	flanking_3UTR	-1,137,738
rs7223183	4.17E-04	-0.219	0.927	0.204	17	4,596,534	*ZMYND15*	NM_032265.1	flanking_3UTR	-377
rs10208385	4.18E-04	0.219	0.927	0.443	2	56,631,513	*FANCL*	NM_018062	downstream	1,608,371
rs201460	4.18E-04	-0.218	0.927	0.246	7	101,528,291	*CUTL1*	NM_001913.2	intron	-790
rs4574338	4.19E-04	0.218	0.927	0.415	3	133,061,191	*CPNE4*	NM_130808	intron	0
rs1908274	4.19E-04	-0.218	0.927	0.158	10	27,946,218	*MKX*	NM_173576	downstream	55,591
rs927171	4.19E-04	-0.218	0.927	0.353	20	55,665,989	*TMEPAI*	NM_199170.1	intron	-2,016

rs12757461	4.20E-04	0.220	0.927	0.228	1	158,682,548	*VANGL2*	NM_020335	downstream	17,460
rs2154631	4.20E-04	-0.218	0.927	0.364	11	58,161,309	*CNTF*	NM_000614.2	flanking_3UTR	-11,531
rs16909808	4.21E-04	-0.218	0.927	0.245	12	14,317,359	*ATF7IP*	NM_018179.3	flanking_5UTR	-92,519
rs12954803	4.22E-04	-0.218	0.927	0.078	18	20,390,567	*HRH4*	ENST00000335188	downstream	459,829
rs2049168	4.23E-04	-0.218	0.927	0.054	3	152,879,418	*IGSF10*	NM_178822	upstream	220,231
rs17056819	4.24E-04	0.218	0.927	0.138	5	158,947,826	*ADRA1B*	ENST00000306675	upstream	356,218
rs6059711	4.24E-04	0.218	0.927	0.165	20	32,244,483	*EIF2S2*	NM_003908	upstream	80,737
rs16943627	4.25E-04	0.218	0.927	0.236	18	23,261,622	*CHST9*	NM_031422.1	flanking_5UTR	-242,445
rs6588628	4.26E-04	0.218	0.927	0.087	1	56,677,799	*PPAP2B*	ENST00000371250	downstream	55,208
rs4948041	4.28E-04	0.218	0.927	0.204	7	55,632,211	*ECOP*	NM_030796.2	flanking_5UTR	-24,570
rs1514882	4.29E-04	0.218	0.927	0.241	7	9,857,994	---	ENST00000361014	downstream	123,554
rs6966915	4.29E-04	0.220	0.927	0.426	7	12,232,513	*TMEM106B*	NM_018374.2	intron	-1,977
rs4812699	4.29E-04	0.218	0.927	0.409	20	41,414,896	*SFRS6*	NM_006275.4	flanking_5UTR	-105,036
rs1448394	4.30E-04	0.219	0.927	0.445	1	104,920,136	---	ENST00000332554	downstream	817,333
rs6817536	4.30E-04	-0.218	0.927	0.083	4	66,466,940	*EPHA5*	NM_182472.1	flanking_5UTR	-248,836
rs6509880	4.30E-04	0.220	0.927	0.369	19	59,709,228	*LAIR2*	NM_021270.2	intron	-1,690
rs4736648	4.31E-04	-0.218	0.927	0.493	8	134,392,519	*NDRG1*	NM_006096.2	flanking_5UTR	-13,839
rs10509313	4.31E-04	-0.218	0.927	0.164	10	70,942,140	*TSPAN15*	NM_012339.3	flanking_3UTR	-4,711
rs1754614	4.31E-04	-0.218	0.927	0.121	10	110,162,502	---	ENST00000363209	downstream	951,085
rs1762059	4.31E-04	-0.218	0.927	0.121	10	110,162,689	---	ENST00000388288	downstream	528,132
rs1202558	4.32E-04	0.218	0.927	0.478	1	229,050,279	*C1orf198*	NM_032800.1	intron	-4,014
rs1534907	4.33E-04	-0.218	0.927	0.244	X	140,914,292	*MAGEC1*	NM_005462.3	flanking_3UTR	-89,443
rs4776338	4.34E-04	0.217	0.927	0.498	15	65,146,937	*SMAD3*	NM_005902.3	intron	-1,185
rs17699214	4.34E-04	-0.220	0.927	0.234	16	74,951,242	*CNTNAP4*	NM_138994.1	intron	-4,333
rs6606683	4.35E-04	0.218	0.927	0.058	12	109,196,595	*ATP2A2*	NM_001681.2	flanking_5UTR	-7,220
rs11158927	4.36E-04	-0.217	0.927	0.264	14	71,535,902	*RGS6*	NM_004296	intron	0
rs6545584	4.37E-04	0.219	0.927	0.460	2	56,702,665	*EFEMP1*	NM_004105.2	flanking_5UTR	-698,983
rs13011654	4.38E-04	-0.217	0.927	0.130	2	155,553,198	*KCNJ3*	NM_002239.2	flanking_3UTR	-131,938
rs17644050	4.38E-04	-0.217	0.927	0.130	2	155,654,905	---	ENST00000385504	upstream	173,631
rs1713478	4.38E-04	-0.217	0.927	0.348	16	12,668,268	*FLJ11151*	NM_018340.1	intron	-1,795
rs3010903	4.39E-04	-0.217	0.927	0.257	1	12,718,631	*LOC126767*	XR_000192.3	flanking_3UTR	-7,324
rs1889107	4.39E-04	0.217	0.927	0.098	9	9,347,065	*PTPRD*	NM_130393.1	flanking_5UTR	-338,330
rs956157	4.40E-04	0.218	0.927	0.453	2	56,526,953	*EFEMP1*	NM_004105.2	flanking_5UTR	-523,271
rs589080	4.40E-04	-0.217	0.927	0.076	5	81,262,304	*ATG10*	NM_031482.3	flanking_5UTR	-41,326
rs3807865	4.44E-04	0.218	0.927	0.469	7	12,216,927	*TMEM106B*	NM_018374.2	flanking_5UTR	-524
rs1726263	4.44E-04	0.217	0.927	0.087	12	123,637,596	*NCOR2*	NM_006312.2	flanking_5UTR	-68,803
rs11562934	4.46E-04	0.217	0.927	0.060	4	118,702,646	*NDST3*	NM_004784.1	flanking_5UTR	-472,302
rs11562866	4.46E-04	0.217	0.927	0.060	4	118,711,700	*NDST3*	NM_004784	upstream	463,360
rs1017272	4.47E-04	0.217	0.927	0.357	2	56,550,282	*EFEMP1*	NM_004105.2	flanking_5UTR	-546,600
rs6840989	4.47E-04	-0.217	0.927	0.078	4	87,037,408	*ARHGAP24*	ENST00000371878	downstream	487,797
rs10245794	4.47E-04	0.217	0.927	0.482	7	20,994,131	*LOC222901*	XR_000588.1	flanking_3UTR	-110,942
rs2461146	4.47E-04	-0.221	0.927	0.468	10	18,143,457	*MRC1*	NM_002438.1	intron	-4,936
rs2515359	4.48E-04	-0.218	0.927	0.438	11	58,073,143	*LPXN*	NM_004811.1	intron	-692
rs12478824	4.50E-04	-0.218	0.927	0.166	2	28,585,206	*PLB1*	NM_153021	intron	0
rs11012077	4.50E-04	0.217	0.927	0.058	10	20,808,104	*PLXDC2*	NM_032812.7	flanking_3UTR	-198,983
rs17625767	4.50E-04	0.217	0.927	0.060	12	109,587,010	*MGC15619*	NM_032369.1	intron	-3,374
rs481067	4.51E-04	-0.217	0.927	0.407	2	169,400,615	*NOSTRIN*	NM_001039724	intron	0
rs9475417	4.51E-04	0.217	0.927	0.264	6	55,796,320	*BMP5*	NM_021073.2	intron	-3,716
rs10960472	4.52E-04	0.217	0.927	0.062	9	1,208,605	*DMRT2*	NM_181872.1	flanking_3UTR	-161,053

rs12428930	4.52E-04	-0.217	0.927	0.287	13	107,737,706	*TNFSF13B*	NM_006573.3	intron	-498
rs2978138	4.53E-04	0.217	0.927	0.478	8	95,119,929	*CDH17*	NM_004063.2	flanking_3UTR	-88,637
rs16982554	4.53E-04	0.217	0.927	0.082	22	25,379,119	*CRYBA4*	NM_001886.1	flanking_3UTR	-22,487
rs9839276	4.54E-04	-0.220	0.927	0.211	3	25,398,770	---	ENST00000264330	intron	0
rs1005996	4.56E-04	0.217	0.927	0.411	3	133,067,827	*CPNE4*	NM_130808.1	intron	-38,971
rs2301284	4.56E-04	0.217	0.927	0.358	19	50,717,228	*VASP*	NM_003370.3	intron	-38
rs6993904	4.57E-04	-0.217	0.927	0.491	8	141,549,512	*NIBP*	NM_031466.3	flanking_5UTR	-11,652
rs2137320	4.58E-04	-0.217	0.927	0.127	11	1,840,918	*LSP1*	NM_001013253.1	intron	-5,561
rs3913369	4.59E-04	-0.217	0.927	0.096	3	55,456,115	*WNT5A*	NM_003392.3	flanking_3UTR	-18,668
rs8181959	4.59E-04	-0.217	0.927	0.353	14	75,119,087	*FLVCR2*	NM_017791	intron	0
rs11733875	4.60E-04	-0.217	0.927	0.303	4	14,836,398	*C1QTNF7*	NM_031911.3	flanking_5UTR	-114,312
rs7981532	4.60E-04	0.217	0.927	0.060	13	71,981,052	*FLJ22624*	NM_024808.2	flanking_5UTR	-218,991
rs974528	4.61E-04	0.217	0.927	0.458	2	137,587,052	---	ENST00000272643	intron	0
rs17429619	4.62E-04	-0.217	0.927	0.095	4	38,610,254	*LOC92689*	NM_138389.1	coding	[3/136]
rs1785394	4.62E-04	-0.217	0.927	0.185	18	5,550,258	*EPB41L3*	NM_012307.2	flanking_5UTR	-16,272
rs1532521	4.63E-04	-0.216	0.927	0.487	12	17,399,544	---	ENST00000386797	upstream	283,838
rs7949324	4.64E-04	-0.216	0.927	0.112	11	80,091,387	*MGC33846*	NM_175885.3	flanking_3UTR	-2,029,307
rs8021868	4.65E-04	-0.218	0.927	0.172	14	56,861,066	*NAT12*	NM_001011713	upstream	65,958
rs12986694	4.66E-04	-0.216	0.927	0.125	2	155,650,216	*KCNJ3*	NM_002239.2	flanking_3UTR	-228,956
rs10900675	4.66E-04	-0.217	0.927	0.218	5	98,969,026	*CHD1*	NM_001270.2	flanking_5UTR	-678,888
rs3016473	4.66E-04	0.216	0.927	0.098	11	122,875,805	---	ENST00000363626	downstream	198,363
rs9989792	4.67E-04	-0.217	0.927	0.242	2	230,823,847	*SP140*	NM_007237.3	intron	-93
rs2884343	4.68E-04	0.217	0.927	0.345	14	52,262,107	*PSMC6*	NM_002806.2	intron	-1,603
rs11918929	4.69E-04	0.216	0.927	0.072	3	104,346,666	---	ENST00000364195	upstream	389,962
rs4384906	4.70E-04	-0.216	0.927	0.154	3	33,215,318	*CRTAP*	NM_006371.3	flanking_3UTR	-51,049
rs6796010	4.70E-04	0.216	0.927	0.060	3	171,096,445	*SAMD7*	NM_182610.1	flanking_5UTR	-15,731
rs17147491	4.70E-04	0.219	0.927	0.061	7	8,337,257	*ICA1*	ENST00000265579	upstream	419,906
rs12676236	4.70E-04	-0.217	0.927	0.198	8	84,251,934	*LOC138046*	NM_173848.3	flanking_5UTR	-1,006,207
rs11223858	4.71E-04	-0.216	0.927	0.091	11	133,855,317	*B3GAT1*	NM_054025	upstream	68,295
rs2222249	4.74E-04	0.216	0.927	0.362	8	60,538,218	*TOX*	NM_014729.2	flanking_5UTR	-343,897
rs1574968	4.75E-04	-0.217	0.927	0.327	5	98,597,609	*CHD1*	NM_001270.2	flanking_5UTR	-307,471
rs7090529	4.75E-04	-0.216	0.927	0.058	10	32,107,718	*ARHGAP12*	NM_018287	downstream	27,513
rs11024408	4.75E-04	0.216	0.927	0.056	11	17,779,872	*SERGEF*	NM_012139.2	intron	-13,336
rs12083196	4.76E-04	0.216	0.927	0.489	1	37,006,017	*CSF3R*	ENST00000373106	upstream	284,551
rs4703858	4.76E-04	-0.217	0.927	0.078	5	81,269,953	*SSBP2*	NM_012446	upstream	187,125
rs616514	4.76E-04	0.216	0.927	0.228	19	15,660,343	*CYP4F12*	NM_023944	intron	0
rs6452348	4.77E-04	0.220	0.927	0.355	5	26,662,551	*CDH9*	NM_016279	downstream	253,915
rs7119134	4.77E-04	-0.216	0.927	0.214	11	86,915,803	*TMEM135*	NM_022918	downstream	203,587
rs6723196	4.78E-04	0.216	0.927	0.355	2	177,421,619	*HNRPA3*	NM_194247.1	flanking_5UTR	-364,155
rs1996848	4.79E-04	0.216	0.927	0.087	2	147,822,979	---	ENST00000386238	downstream	95,556
rs17500483	4.81E-04	-0.217	0.927	0.253	5	145,513,052	*LARS*	NM_020117.8	intron	-69
rs10464904	4.84E-04	0.216	0.927	0.162	8	64,117,714	*TTPA*	NM_000370	downstream	17,212
rs6562760	4.84E-04	0.216	0.927	0.185	13	72,855,682	*KLF12*	NM_007249.3	flanking_3UTR	-302,468
rs7119	4.84E-04	-0.216	0.927	0.358	15	75,564,687	*HMG20A*	NM_018200.2	3UTR	[2320/313]
rs1464467	4.85E-04	0.216	0.927	0.466	8	84,234,876	*LOC138046*	NM_173848.3	flanking_5UTR	-1,023,265
rs4681620	4.86E-04	0.216	0.927	0.469	3	151,664,103	*TSC22D2*	NM_014779.2	flanking_3UTR	-3,782
rs7300729	4.86E-04	0.217	0.927	0.232	12	64,198,788	*MSRB3*	NM_198080	downstream	51,843
rs2252389	4.87E-04	0.216	0.927	0.192	1	159,188,819	*ITLN2*	NM_080878.2	intron	-215
rs2971177	4.87E-04	-0.216	0.927	0.489	12	17,396,780	*FLJ22655*	NM_024730.2	flanking_3UTR	-728,290

rs1407441	4.87E-04	0.216	0.927	0.138	13	49,188,991	*KPNA3*	NM_002267.2	intron	-2,921
rs17764668	4.87E-04	-0.216	0.927	0.082	14	79,311,969	*NRXN3*	NM_004796	intron	0
rs2860508	4.88E-04	0.216	0.927	0.107	11	56,688,408	*OR5AK2*	NM_001005323	downstream	174,514
rs17327860	4.89E-04	-0.216	0.927	0.168	3	55,437,613	*LRTM1*	NM_020678	upstream	500,501
rs7310140	4.89E-04	-0.216	0.927	0.263	12	94,746,318	---	ENST00000378403	upstream	11,626
rs17234990	4.90E-04	-0.216	0.927	0.191	3	7,170,141	*GRM7*	NM_181875.1	intron	-6,786
rs1369574	4.90E-04	-0.216	0.927	0.199	3	120,285,968	*IGSF11*	ENST00000381279	intron	0
rs17256004	4.90E-04	-0.216	0.927	0.074	X	12,735,560	*PRPS2*	NM_002765	intron	0
rs7582078	4.91E-04	0.216	0.927	0.454	2	208,355,096	*FZD5*	NM_003468.2	flanking_5UTR	-12,733
rs1494207	4.91E-04	-0.215	0.927	0.159	10	27,952,307	*IRXL1*	NM_173576.1	flanking_3UTR	-49,502
rs1872062	4.92E-04	0.215	0.927	0.266	2	111,346,960	*ACOXL*	NM_018308.1	intron	-31,507
rs9380590	4.92E-04	-0.216	0.927	0.113	6	36,781,488	*FLJ43093*	NM_207498.1	intron	-1,291
rs10483481	4.92E-04	-0.215	0.927	0.496	14	36,515,582	*SLC25A21*	NM_030631	intron	0
rs825836	4.93E-04	0.215	0.927	0.319	16	72,100,261	*C16orf47*	NM_207385	upstream	364,414
rs4721059	4.94E-04	0.217	0.927	0.423	7	12,234,020	*TMEM106B*	NM_018374	intron	0
rs6678816	4.95E-04	-0.216	0.927	0.102	1	41,258,176	*FLJ23878*	NM_144990.2	intron	-309
rs1326683	4.95E-04	-0.215	0.927	0.355	13	71,570,155	---	ENST00000363167	downstream	119,810
rs1117751	4.96E-04	-0.216	0.927	0.351	3	30,442,159	*TGFBR2*	NM_003242.4	flanking_5UTR	-180,839
rs2509928	4.96E-04	-0.215	0.927	0.442	11	58,162,034	*CNTF*	NM_000614	downstream	12,256
rs13014895	4.97E-04	-0.216	0.927	0.141	2	30,394,337	*LBH*	NM_030915.1	flanking_3UTR	-57,946
rs6741085	4.97E-04	0.216	0.927	0.167	2	80,083,935	*CTNNA2*	NM_004389.2	flanking_3UTR	-93,501
rs901173	4.97E-04	0.215	0.927	0.188	8	25,892,036	---	ENST00000380737	intron	0
rs2004489	4.97E-04	-0.215	0.927	0.156	10	16,850,853	*RSU1*	NM_152724	intron	0
rs3926062	4.97E-04	-0.215	0.927	0.168	12	88,028,143	*DUSP6*	NM_022652.2	flanking_3UTR	-237,825
rs1786923	4.98E-04	0.215	0.927	0.310	10	67,359,195	*CTNNA3*	NM_013266	intron	0
rs8040318	5.00E-04	-0.216	0.927	0.168	15	75,590,715	*HMG20A*	NM_018200.2	flanking_3UTR	-25,715
rs4800889	5.00E-04	0.215	0.927	0.321	18	24,721,368	*CDH2*	NM_001792.2	flanking_5UTR	-710,179
rs11015914	5.01E-04	-0.216	0.927	0.158	10	27,951,677	*IRXL1*	NM_173576.1	flanking_3UTR	-50,132
rs9317289	5.01E-04	-0.215	0.927	0.389	13	62,626,982	*OR7E156P*	NR_002171.1	flanking_5UTR	-582,587
rs7359991	5.01E-04	0.215	0.927	0.105	19	36,059,348	*ZNF536*	NM_014717.1	flanking_3UTR	-318,543
rs10087985	5.02E-04	-0.217	0.927	0.381	8	84,240,387	*RALYL*	ENST00000345957	upstream	1,323,397
rs10197140	5.03E-04	0.215	0.927	0.248	2	111,326,414	*ACOXL*	NM_018308.1	intron	-10,961
rs17440692	5.04E-04	-0.216	0.927	0.133	14	32,276,258	*AKAP6*	NM_004274	intron	0
rs880064	5.04E-04	-0.215	0.927	0.163	20	41,808,846	*FAM112A*	NM_001008901.1	flanking_5UTR	-19,790
rs17075600	5.06E-04	-0.215	0.927	0.136	3	43,376,272	*TMEM16K*	NM_018075.2	flanking_3UTR	-6,572
rs4236026	5.06E-04	0.215	0.927	0.165	6	23,351,541	*HDGFL1*	NM_138574.1	flanking_3UTR	-671,670
rs11069727	5.06E-04	-0.215	0.927	0.289	13	107,735,834	*TNFSF13B*	NM_006573	intron	0
rs7128670	5.07E-04	0.218	0.927	0.091	11	103,409,766	*PDGFD*	NM_033135	intron	0
rs2511506	5.09E-04	0.215	0.927	0.442	11	130,702,761	*C11orf39*	NM_207432	downstream	332,586
rs7745098	5.10E-04	0.215	0.927	0.353	6	135,456,697	*HBS1L*	NM_006620.2	flanking_5UTR	-38,982
rs11079337	5.10E-04	-0.215	0.927	0.338	17	53,517,495	*DYNLL2*	NM_080677.1	intron	-1,452
rs11585691	5.11E-04	0.215	0.927	0.228	1	158,686,660	*VANGL2*	NM_020335.1	flanking_3UTR	-21,572
rs6722231	5.11E-04	0.215	0.927	0.342	2	56,707,850	*EFEMP1*	NM_004105.2	flanking_5UTR	-704,168
rs17801923	5.11E-04	-0.215	0.927	0.071	20	49,604,747	*NFATC2*	NM_173091.2	flanking_5UTR	-12,082
rs11921795	5.12E-04	0.215	0.927	0.365	3	133,076,151	*CPNE4*	NM_130808.1	intron	-30,647
rs2064189	5.14E-04	-0.215	0.927	0.317	22	40,457,945	*RP5-821D11.2*	NM_152513	intron	0
rs5751144	5.14E-04	-0.215	0.927	0.317	22	40,458,043	*RP5-821D11.2*	NM_152513	intron	0
rs17132455	5.15E-04	-0.216	0.927	0.074	4	2,290,735	*ZFYVE28*	NM_020972	intron	0
rs17541774	5.16E-04	-0.215	0.927	0.051	5	16,840,384	*MYO10*	NM_012334.1	intron	-3,806

rs502808	5.17E-04	0.215	0.927	0.446	1	36,985,204	*GRIK3*	NM_000831.2	flanking_3UTR	-53,997
rs1543099	5.18E-04	0.215	0.927	0.375	3	133,002,331	*CPNE4*	NM_130808.1	intron	-77,172
rs12026360	5.19E-04	-0.215	0.927	0.341	1	104,890,308	*AMY1C*	NM_001008219.1	flanking_3UTR	-787,475
rs4922060	5.19E-04	0.217	0.927	0.122	8	19,452,788	*ChGn*	NM_018371	intron	0
rs28603687	5.19E-04	0.215	0.927	0.257	17	1,098,342	*TUSC5*	NM_172367.1	flanking_5UTR	-31,365
rs1513378	5.20E-04	0.215	0.927	0.413	3	133,061,251	*CPNE4*	NM_130808	intron	0
rs13206808	5.20E-04	0.215	0.927	0.076	6	72,639,147	*RIMS1*	ENST00000354545	upstream	234,574
rs4520107	5.20E-04	-0.215	0.927	0.053	7	135,674,717	*MTPN*	NM_145808.1	flanking_5UTR	-362,113
rs7187344	5.20E-04	-0.216	0.927	0.137	16	79,510,552	*C16orf61*	NM_020188	downstream	56,654
rs8003131	5.22E-04	-0.214	0.927	0.498	14	36,514,545	*SLC25A21*	NM_030631.1	intron	-100,585
rs9922687	5.22E-04	-0.214	0.927	0.268	16	86,128,352	*ZCCHC14*	NM_015144.1	flanking_5UTR	-45,386
rs7600814	5.26E-04	0.215	0.927	0.392	2	56,497,459	*VRK2*	NM_006296	upstream	1,629,765
rs722825	5.26E-04	0.214	0.927	0.313	4	176,914,764	*GPM6A*	NM_005277.3	intron	-54,852
rs12280107	5.26E-04	0.214	0.927	0.469	11	58,781,598	---	ENST00000387068	upstream	42,850
rs7671984	5.27E-04	-0.215	0.927	0.369	4	62,926,242	---	ENST00000314406	upstream	439,729
rs13337426	5.27E-04	0.214	0.927	0.078	16	5,499,150	*FAM86A*	NM_201598.1	flanking_5UTR	-411,368
rs11862584	5.27E-04	0.214	0.927	0.379	16	25,692,099	*ZNF694*	NM_001012981.2	flanking_5UTR	-515,756
rs2830988	5.27E-04	0.215	0.927	0.255	21	27,799,163	---	ENST00000324988	upstream	508,409
rs216723	5.28E-04	-0.214	0.927	0.359	7	28,543,291	*CREB5*	NM_001011666	intron	0
rs13286735	5.28E-04	0.215	0.927	0.176	9	4,321,760	*SLC1A1*	NM_004170.4	flanking_5UTR	-158,684
rs184041	5.29E-04	-0.214	0.927	0.241	1	163,728,715	*RXRG*	ENST00000367891	upstream	47,661
rs11792770	5.29E-04	0.214	0.927	0.065	9	15,721,398	*C9orf93*	NM_173550.2	intron	-1,602
rs12902898	5.31E-04	-0.215	0.927	0.187	15	75,636,246	*LINGO1*	NM_032808	downstream	57,195
rs6636623	5.31E-04	-0.215	0.927	0.505	X	141,061,612	*MAGEC2*	ENST00000375037	intron	0
rs12102841	5.34E-04	0.214	0.927	0.196	16	86,630,075	*BANP*	ENST00000377883	upstream	19,896
rs1987950	5.34E-04	0.215	0.927	0.423	21	27,804,888	*C21orf94*	NM_145180.2	flanking_5UTR	-502,684
rs2658109	5.36E-04	-0.219	0.927	0.317	5	10,180,437	*LOC134145*	NM_199133.1	flanking_3UTR	-99,008
rs2870064	5.37E-04	-0.214	0.927	0.475	2	56,614,096	*EFEMP1*	NM_004105.2	flanking_5UTR	-610,414
rs2194794	5.38E-04	0.214	0.927	0.397	2	56,640,604	*EFEMP1*	NM_004105.2	flanking_5UTR	-636,922
rs7696032	5.38E-04	-0.217	0.927	0.409	4	144,249,450	*USP38*	NM_032557	upstream	76,098
rs3845571	5.39E-04	-0.214	0.927	0.187	1	41,488,626	*SCMH1*	NM_012236.1	flanking_5UTR	-8,251
rs11597892	5.39E-04	-0.214	0.927	0.154	10	20,324,089	*PLXDC2*	NM_032812	intron	0
rs13283389	5.40E-04	-0.214	0.927	0.389	9	104,454,298	*CYLC2*	NM_001340.1	flanking_5UTR	-343,186
rs9564010	5.42E-04	-0.214	0.927	0.375	13	62,683,758	*OR7E156P*	NR_002171.1	flanking_5UTR	-525,811
rs17323604	5.42E-04	-0.214	0.927	0.240	X	140,913,831	*MAGEC1*	NM_005462.3	flanking_3UTR	-88,982
rs6743599	5.43E-04	0.214	0.927	0.120	2	68,889,558	*ARHGAP25*	NM_001007231.1	intron	-1,442
rs4946672	5.43E-04	0.214	0.927	0.437	6	106,097,724	*PREP*	NM_002726	upstream	140,062
rs12096661	5.44E-04	0.214	0.927	0.426	1	208,886,269	*HHAT*	NM_018194.1	intron	-22,632
rs4578488	5.44E-04	-0.214	0.927	0.413	12	13,316,196	*EMP1*	NM_001423.1	flanking_3UTR	-55,221
rs17357954	5.45E-04	-0.214	0.927	0.103	1	41,259,924	*FLJ23878*	NM_144990.2	5UTR	[42/52]
rs213753	5.45E-04	-0.214	0.927	0.103	1	41,379,918	*SCMH1*	NM_012236.1	intron	-1,219
rs10820216	5.46E-04	-0.214	0.927	0.391	9	104,445,236	---	ENST00000374801	CDS	0
rs5753220	5.47E-04	0.214	0.927	0.335	22	29,316,350	*PES1*	NM_014303.2	intron	-1,093
rs11545737	5.49E-04	0.216	0.927	0.172	14	73,105,424	*ACOT2*	NM_006821.3	flanking_5UTR	-108
rs10788950	5.50E-04	0.214	0.927	0.188	1	53,485,924	*LRP8*	NM_004631.3	intron	-608
rs851903	5.53E-04	-0.214	0.927	0.185	12	60,611,637	*FAM19A2*	NM_178539.3	intron	-64,163
rs3000777	5.54E-04	-0.214	0.927	0.409	1	225,651,274	*CDC42BPA*	NM_003607	upstream	78,476
rs7712171	5.55E-04	-0.214	0.927	0.326	5	33,140,713	*C5orf23*	NM_024563	downstream	313,137
rs7918637	5.55E-04	-0.214	0.927	0.172	10	107,932,289	*SORCS1*	NM_001013031.1	flanking_3UTR	-391,122

rs3134615	5.56E-04	0.213	0.927	0.105	1	40,134,653	*MYCL1*	NM_001033082.1	3UTR	[968/977]
rs3843011	5.56E-04	-0.214	0.927	0.435	5	10,328,870	*CMBL*	ENST00000384359	upstream	42,315
rs10516088	5.57E-04	-0.213	0.927	0.194	5	171,083,028	*FBXW11*	NM_012300.2	flanking_3UTR	-138,133
rs2516390	5.57E-04	-0.214	0.927	0.353	6	31,637,862	*NFKBIL1*	NM_005007.2	flanking_3UTR	-3,277
rs13322091	5.58E-04	0.213	0.927	0.179	3	152,097,290	*CLRN1*	NM_052995	downstream	29,350
rs2109112	5.58E-04	-0.214	0.927	0.442	12	3,857,734	*PARP11*	NM_020367.3	flanking_5UTR	-4,871
rs4319365	5.59E-04	0.213	0.927	0.426	1	56,680,308	*PPAP2B*	NM_003713.3	flanking_3UTR	-52,713
rs7109110	5.59E-04	-0.213	0.927	0.056	11	108,162,717	*DDX10*	NM_004398	intron	0
rs1793553	5.59E-04	0.213	0.927	0.453	11	130,698,952	*C11orf39*	NM_207432.1	flanking_3UTR	-334,465
rs8008323	5.59E-04	0.213	0.927	0.216	14	49,022,584	*RPS29*	NM_001030001.1	flanking_3UTR	-91,205
rs2849233	5.59E-04	-0.213	0.927	0.274	18	46,585,551	*MRO*	NM_031939.2	coding	[29/153]
rs1353142	5.60E-04	0.213	0.927	0.112	11	26,604,647	*TMEM16C*	NM_031418.1	intron	-2,026
rs2042119	5.61E-04	-0.214	0.927	0.453	2	56,778,162	*EFEMP1*	NM_004105.2	flanking_5UTR	-774,480
rs3111083	5.61E-04	-0.215	0.927	0.290	5	10,194,550	*LOC134145*	NM_199133.1	flanking_3UTR	-84,895
rs3859666	5.61E-04	-0.213	0.927	0.216	20	4,233,532	*PRNP*	NM_000311	upstream	381,492
rs6674883	5.62E-04	0.213	0.927	0.455	1	216,075,411	*SPATA17*	NM_138796	intron	0
rs4965245	5.62E-04	-0.213	0.927	0.399	15	97,825,915	*MEF2A*	NM_005587	upstream	97,823
rs12202855	5.63E-04	-0.213	0.927	0.136	6	134,656,723	---	ENST00000367858	intron	0
rs4607611	5.64E-04	0.215	0.927	0.270	8	14,853,084	*SGCZ*	NM_139167.1	flanking_5UTR	-396,278
rs1477717	5.64E-04	-0.214	0.927	0.154	21	27,536,742	*ADAMTS5*	NM_007038.2	flanking_5UTR	-276,039
rs2420244	5.65E-04	0.213	0.927	0.493	10	117,925,055	*GFRA1*	NM_005264.2	intron	-36,078
rs16967866	5.65E-04	-0.213	0.927	0.080	17	70,918,976	*GRB2*	NM_203506.1	flanking_5UTR	-5,647
rs1642922	5.66E-04	-0.213	0.927	0.203	2	225,014,076	*CUL3*	NM_003590.2	flanking_3UTR	-29,037
rs2201465	5.66E-04	0.214	0.927	0.228	12	42,111,682	*ADAMTS20*	NM_025003.2	intron	-116
rs12026014	5.68E-04	0.214	0.927	0.436	1	38,833,082	*RRAGC*	NM_022157.2	flanking_3UTR	-244,510
rs11008110	5.68E-04	0.213	0.927	0.351	10	30,889,472	*LYZL2*	NM_183058	downstream	51,249
rs1497104	5.68E-04	-0.213	0.927	0.214	11	82,089,283	---	ENST00000357859	downstream	10,825
rs11233281	5.68E-04	-0.213	0.927	0.214	11	82,092,175	---	ENST00000313090	downstream	28,124
rs3108333	5.69E-04	0.214	0.927	0.115	3	127,704,897	*UROC1*	NM_144639	intron	0
rs4939626	5.69E-04	-0.213	0.927	0.147	18	46,094,553	*CXXC1*	NM_014593.2	flanking_5UTR	-26,367
rs1670136	5.70E-04	0.216	0.927	0.348	10	67,371,554	*CTNNA3*	NM_013266.1	intron	-21,173
rs4945334	5.70E-04	0.213	0.927	0.326	11	78,607,351	*NARS2*	NM_024678.3	flanking_5UTR	-643,984
rs7202468	5.70E-04	-0.213	0.927	0.466	16	26,259,883	*TNT*	NM_182831.1	flanking_5UTR	-725,646
rs7082958	5.71E-04	-0.213	0.927	0.255	10	109,784,983	*SORCS1*	NM_052918.3	flanking_5UTR	-870,701
rs1509559	5.71E-04	-0.213	0.927	0.482	15	79,178,466	*C15orf26*	NM_173528.1	flanking_5UTR	-338
rs1509560	5.71E-04	-0.213	0.927	0.482	15	79,178,768	*C15orf26*	NM_173528.1	flanking_5UTR	-36
rs7212762	5.71E-04	-0.213	0.927	0.165	17	76,869,498	*MGC15523*	NM_138570.1	intron	-370
rs4481591	5.72E-04	0.213	0.927	0.457	8	84,213,447	*LOC138046*	NM_173848.3	flanking_5UTR	-1,044,694
rs4512880	5.72E-04	-0.213	0.927	0.223	11	86,955,572	*TMEM135*	NM_022918	downstream	243,356
rs9402684	5.73E-04	0.215	0.927	0.377	6	135,460,998	*HBS1L*	NM_006620.2	flanking_5UTR	-43,283
rs1990622	5.73E-04	0.213	0.927	0.411	7	12,250,312	*TMEM106B*	NM_018374.2	flanking_3UTR	-6,901
rs9998109	5.74E-04	-0.213	0.927	0.230	4	118,464,432	*TRAM1L1*	NM_152402.1	flanking_5UTR	-238,252
rs9294716	5.74E-04	-0.217	0.927	0.246	6	67,448,076	*LOC442229*	XR_000273.2	flanking_3UTR	-891,983
rs10278072	5.74E-04	0.214	0.927	0.333	7	135,547,543	*MTPN*	NM_145808.1	flanking_5UTR	-234,939
rs17124192	5.76E-04	-0.216	0.927	0.076	1	63,229,385	*ATG4C*	NM_178221	downstream	125,613
rs2071590	5.76E-04	-0.215	0.927	0.349	6	31,647,747	*LTA*	NM_000595.2	flanking_5UTR	-325
rs12443577	5.76E-04	0.213	0.927	0.380	16	25,692,421	*HS3ST4*	NM_006040	intron	0
rs10497148	5.77E-04	-0.217	0.927	0.130	2	155,791,590	---	ENST00000385504	upstream	36,946
rs12494229	5.77E-04	0.213	0.927	0.311	3	190,356,633	*FAM79B*	NM_198485.1	flanking_5UTR	-15,824

rs2360644	5.77E-04	0.215	0.927	0.426	12	50,790,305	*KRT80*	ENST00000384531	downstream	1,171,703
rs12909509	5.77E-04	0.213	0.927	0.259	15	55,484,321	*CGNL1*	NM_032866	intron	0
rs10179397	5.78E-04	-0.213	0.927	0.281	2	26,488,832	*C2orf39*	ENST00000288710	intron	0
rs17326775	5.79E-04	0.213	0.927	0.109	2	152,861,801	*FMNL2*	NM_001004422.1	flanking_5UTR	-38,196
rs7933513	5.80E-04	-0.213	0.927	0.098	11	20,418,201	*PRMT3*	NM_005788.1	intron	-12,051
rs2874641	5.80E-04	0.213	0.927	0.480	17	11,400,989	*FLJ45455*	NM_207386.1	intron	-807
rs7549591	5.81E-04	0.214	0.927	0.487	1	36,970,939	*GRIK3*	NM_000831.2	flanking_3UTR	-68,262
rs2356167	5.82E-04	-0.214	0.927	0.289	7	12,327,330	*TMEM106B*	NM_018374.2	flanking_3UTR	-83,919
rs7085698	5.82E-04	0.213	0.927	0.188	10	90,676,023	*STAMBPL1*	NM_020799.2	flanking_3UTR	-2,799
rs13431665	5.83E-04	-0.214	0.927	0.203	2	124,471,212	*CNTNAP5*	NM_138996.1	flanking_5UTR	-28,122
rs11630540	5.83E-04	-0.213	0.927	0.233	15	75,620,680	*HMG20A*	NM_018200.2	flanking_3UTR	-55,680
rs1908275	5.85E-04	-0.213	0.927	0.167	10	27,946,354	*MKX*	NM_173576	downstream	55,455
rs7111680	5.85E-04	0.213	0.927	0.237	11	89,084,402	*PSMAL*	NM_153696	downstream	12,871
rs2826625	5.85E-04	-0.213	0.927	0.194	21	21,262,922	*NCAM2*	NM_004540	upstream	311,845
rs2910642	5.86E-04	-0.213	0.927	0.482	5	58,431,868	*PDE4D*	NM_006203	intron	0
rs2473129	5.86E-04	0.215	0.927	0.463	6	144,637,786	*UTRN*	ENST00000367545	upstream	16,872
rs1559017	5.87E-04	-0.213	0.927	0.384	3	191,779,730	*IL1RAP*	NM_002182	intron	0
rs4472816	5.89E-04	0.213	0.927	0.310	1	208,896,943	*HHAT*	NM_018194.1	intron	-17,310
rs10210929	5.90E-04	0.213	0.927	0.336	2	56,631,864	*EFEMP1*	NM_004105.2	flanking_5UTR	-628,182
---	5.90E-04	0.213	0.927	0.239	10	17,052,237	*CUBN*	NM_001081	intron	0
rs2660844	5.90E-04	-0.213	0.927	0.083	12	94,962,450	*LTA4H*	NM_000895	upstream	8,954
rs1823903	5.92E-04	-0.215	0.927	0.329	9	2,697,420	*KCNV2*	NM_133497	upstream	10,082
rs5754679	5.92E-04	0.215	0.927	0.055	22	32,527,367	*LARGE*	NM_004737	intron	0
rs9837084	5.93E-04	0.212	0.927	0.353	3	152,221,445	*USH3A*	NM_174878.2	flanking_5UTR	-47,969
rs1793630	5.94E-04	0.214	0.927	0.467	11	131,431,408	*HNT*	NM_016522.2	intron	-89,978
rs2277987	5.96E-04	-0.212	0.927	0.304	19	8,458,273	*HNRPM*	NM_005968.2	intron	-314
rs37872	5.97E-04	-0.213	0.927	0.104	7	78,266,677	*MAGI2*	NM_012301	intron	0
rs9599192	5.97E-04	-0.214	0.927	0.114	13	66,626,052	*PCDH9*	NM_203487.1	intron	-71,486
rs534024	5.98E-04	0.212	0.927	0.333	3	171,905,581	*SLC7A14*	NM_020949	upstream	119,029
rs2192828	6.00E-04	0.213	0.927	0.329	7	12,343,336	*TMEM106B*	NM_018374.2	flanking_3UTR	-99,925
rs4664932	6.02E-04	0.212	0.927	0.103	2	158,779,506	*LOC130940*	NM_138803.2	intron	-5,847
rs13302712	6.02E-04	0.212	0.927	0.080	9	98,300,749	*CDC14B*	NM_033332.1	intron	-2,287
rs2830457	6.02E-04	0.213	0.927	0.153	21	27,046,037	*ADAMTS1*	NM_006988.3	flanking_3UTR	-84,440
rs1864549	6.04E-04	-0.215	0.927	0.121	2	79,552,919	*REG3A*	NM_138937	upstream	312,532
rs2163761	6.04E-04	0.212	0.927	0.326	5	168,143,832	*SLIT3*	NM_003062.1	intron	-1,658
rs7891017	6.04E-04	0.212	0.927	0.159	X	135,789,462	*RBMX*	NM_002139	intron	0
rs9321351	6.05E-04	0.212	0.927	0.476	6	132,927,116	*TAAR6*	NM_175067.1	flanking_5UTR	-6,038
rs2223404	6.05E-04	-0.213	0.927	0.416	X	141,059,951	*MAGEC2*	NM_016249.2	flanking_3UTR	-57,845
rs1472823	6.06E-04	0.213	0.927	0.471	1	36,993,308	*CSF3R*	NM_000760	upstream	271,842
rs715364	6.06E-04	0.214	0.927	0.134	11	126,458,703	*PRR10*	NM_173579.1	flanking_3UTR	-77,540
rs12583006	6.06E-04	-0.212	0.927	0.289	13	107,735,453	*TNFSF13B*	ENST00000218706	intron	0
rs13285529	6.08E-04	-0.212	0.927	0.065	9	104,397,700	*CYLC2*	NM_001340.1	flanking_5UTR	-399,784
rs1401191	6.08E-04	0.212	0.927	0.373	18	24,711,165	*CDH2*	NM_001792.2	flanking_5UTR	-699,976
rs7121003	6.10E-04	-0.212	0.927	0.194	11	86,964,252	*FLJ22104*	NM_022918.2	flanking_3UTR	-252,032
rs7982249	6.10E-04	0.213	0.927	0.057	13	71,981,415	*FLJ22624*	NM_024808.2	flanking_5UTR	-218,628
rs11131367	6.11E-04	-0.213	0.927	0.370	4	62,932,638	*LPHN3*	NM_015236.3	flanking_3UTR	-311,875
rs937237	6.12E-04	0.212	0.927	0.054	15	75,534,924	*HMG20A*	NM_018200.2	intron	-2,877
rs5759632	6.12E-04	0.215	0.927	0.439	22	19,312,876	*PCQAP*	NM_001003891.1	flanking_3UTR	-40,957
rs1298582	6.14E-04	-0.212	0.927	0.337	7	141,353,999	*MGAM*	NM_004668	intron	0

rs4629773	6.16E-04	0.212	0.927	0.114	7	152,807,638	*FLJ42291*	NM_207367.1	flanking_5UTR	-65,657
rs583946	6.16E-04	0.216	0.927	0.229	19	15,661,153	*CYP4F12*	NM_023944	intron	0
rs1353885	6.17E-04	0.212	0.927	0.480	5	135,672,914	*TRPC7*	NM_020389.1	intron	-6,270
rs4837218	6.18E-04	0.212	0.927	0.224	9	129,795,514	*C9orf132*	NM_203305.1	flanking_5UTR	-42,700
rs2559534	6.18E-04	0.212	0.927	0.212	10	22,968,874	*PIP5K2A*	NM_005028.3	intron	-30,222
rs3017569	6.18E-04	0.212	0.927	0.091	11	56,681,597	*LRRC55*	NM_001005210.1	flanking_5UTR	-24,200
rs11223888	6.18E-04	0.212	0.927	0.303	11	133,908,599	*B3GAT1*	NM_054025	upstream	121,577
rs1552846	6.19E-04	-0.212	0.927	0.344	17	68,547,208	*SLC39A11*	NM_139177	intron	0
rs1282970	6.20E-04	-0.213	0.927	0.230	3	113,049,928	*PHLDB2*	NM_145753	intron	0
rs2579206	6.21E-04	-0.212	0.927	0.151	12	20,074,000	*PDE3A*	NM_000921	upstream	339,486
rs890100	6.23E-04	-0.212	0.927	0.460	2	56,587,539	*FANCL*	NM_018062	downstream	1,652,345
rs10508940	6.23E-04	0.212	0.927	0.310	10	52,888,932	*PRKG1*	NM_006258.1	intron	-8,557
rs1033815	6.25E-04	-0.212	0.927	0.062	20	19,003,731	*SLC24A3*	NM_020689.3	flanking_5UTR	-137,559
rs6677161	6.26E-04	0.212	0.927	0.143	1	169,063,828	*PRRX1*	NM_022716	downstream	88,663
rs645827	6.27E-04	0.213	0.927	0.228	19	15,658,409	*CYP4F12*	NM_023944	intron	0
rs1587765	6.28E-04	0.213	0.927	0.281	4	70,601,693	*SULT1B1*	NM_014465.2	flanking_3UTR	-25,582
rs8032656	6.28E-04	0.211	0.927	0.409	15	90,403,318	*SLCO3A1*	NM_013272	intron	0
rs6425998	6.29E-04	0.211	0.927	0.487	1	36,970,307	*GRIK3*	NM_000831.2	flanking_3UTR	-68,894
rs8050827	6.30E-04	0.212	0.927	0.432	16	12,027,177	*RUNDC2A*	NM_032167.1	intron	-1,501
rs5979755	6.30E-04	-0.212	0.927	0.372	X	7,449,290	*STS*	NM_000351.3	flanking_3UTR	-166,608
rs2398820	6.31E-04	0.213	0.927	0.473	9	95,036,394	*WNK2*	NM_006648.3	intron	-517
rs11017044	6.32E-04	-0.211	0.927	0.400	10	131,690,491	*EBF3*	NM_001005463	upstream	38,396
rs1440030	6.33E-04	0.211	0.927	0.330	7	12,349,162	*TMEM106B*	NM_018374.2	flanking_3UTR	-105,751
rs6672914	6.34E-04	-0.211	0.927	0.196	1	41,439,516	*SCMH1*	NM_012236.1	intron	-15,007
rs17039629	6.34E-04	-0.211	0.927	0.411	2	56,624,811	*FANCL*	NM_018062	downstream	1,615,073
rs25431	6.35E-04	-0.211	0.927	0.312	15	78,917,942	*KIAA1199*	NM_018689.1	intron	-35,318
rs2477648	6.36E-04	0.211	0.927	0.326	10	18,143,407	*MRC1*	NM_002438.1	intron	-4,886
rs7326604	6.36E-04	0.211	0.927	0.062	13	28,796,086	*KIAA0774*	NM_001033602	intron	0
rs9348404	6.38E-04	0.211	0.927	0.246	6	20,096,632	*ID4*	NM_001546.2	flanking_3UTR	-147,738
rs9295456	6.38E-04	0.211	0.927	0.246	6	20,099,425	*ID4*	NM_001546	downstream	150,531
rs5936023	6.39E-04	0.212	0.927	0.302	X	15,637,898	---	ENST00000380333	downstream	6,505
rs9490529	6.40E-04	0.211	0.927	0.351	6	123,073,087	*PKIB*	NM_181794	intron	0
rs17465162	6.41E-04	0.212	0.927	0.245	2	111,329,616	*ACOXL*	NM_018308.1	intron	-14,163
rs1390027	6.41E-04	0.211	0.927	0.263	8	62,891,127	*ASPH*	NM_004318.2	flanking_5UTR	-101,567
rs12774599	6.42E-04	-0.212	0.927	0.087	10	130,795,782	*MGMT*	NM_002412.2	flanking_5UTR	-359,674
rs9292484	6.43E-04	0.211	0.927	0.261	5	33,189,004	*C5orf23*	NM_024563	downstream	361,428
rs7788841	6.43E-04	-0.211	0.927	0.053	7	135,677,466	*CHRM2*	NM_001006632	upstream	526,490
rs1371040	6.44E-04	0.213	0.927	0.268	2	147,881,623	*ACVR2A*	NM_001616.3	flanking_5UTR	-437,417
rs3925081	6.44E-04	-0.211	0.927	0.221	3	113,038,831	*PLCXD2*	NM_153268.1	intron	-8,527
rs1409314	6.46E-04	-0.211	0.927	0.486	10	120,605,742	*C10orf46*	NM_153810.3	flanking_5UTR	-100,994
rs8087223	6.46E-04	0.211	0.927	0.185	18	8,346,825	*PTPRM*	NM_002845	intron	0
rs11694963	6.47E-04	0.211	0.927	0.429	2	56,492,131	*EFEMP1*	NM_004105.2	flanking_5UTR	-488,449
rs2121665	6.47E-04	-0.212	0.927	0.330	5	98,565,021	*CHD1*	NM_001270.2	flanking_5UTR	-274,883
rs1033981	6.47E-04	-0.214	0.927	0.346	13	71,566,635	*DACH1*	NM_004392.4	flanking_5UTR	-227,304
rs222757	6.48E-04	0.211	0.927	0.476	17	3,516,662	*TAX1BP3*	NM_014604.2	intron	-1,821
rs1366150	6.49E-04	0.211	0.927	0.154	5	162,560,222	*CCNG1*	NM_004060	upstream	236,933
rs1561860	6.50E-04	0.211	0.927	0.496	15	97,826,919	*LYSMD4*	NM_152449	downstream	258,218
rs2256974	6.51E-04	0.211	0.927	0.306	6	31,663,371	*LST1*	NM_007161.2	intron	-26
rs1519167	6.51E-04	-0.211	0.927	0.120	8	4,055,211	---	ENST00000387211	downstream	509,710

rs17624958	6.51E-04	-0.211	0.927	0.263	8	16,998,045	*EFHA2*	NM_181723.1	intron	-2,293
rs2920942	6.51E-04	-0.211	0.927	0.473	8	80,775,269	*STMN2*	NM_007029.2	flanking_3UTR	-35,477
rs10894526	6.51E-04	0.211	0.927	0.241	11	131,642,095	*HNT*	ENST00000374784	intron	0
rs2212815	6.51E-04	0.214	0.927	0.412	21	27,049,551	*CYYR1*	NM_052954	upstream	182,099
rs6721009	6.52E-04	0.211	0.927	0.384	2	56,632,191	*VRK2*	ENST00000320658	upstream	501,822
rs12056715	6.54E-04	-0.213	0.927	0.261	8	16,983,655	*EFHA2*	NM_181723	intron	0
rs1431586	6.54E-04	-0.211	0.927	0.152	8	64,553,283	*YTHDF3*	NM_152758.3	flanking_3UTR	-265,386
rs9816432	6.55E-04	0.212	0.927	0.331	3	88,525,111	*C3orf38*	NM_173824	downstream	235,310
rs11958511	6.55E-04	0.211	0.927	0.141	5	158,945,623	*IL12B*	NM_002187.2	flanking_5UTR	-255,564
rs2063643	6.55E-04	-0.211	0.927	0.176	6	52,586,323	*TRAM2*	NM_012288.3	flanking_5UTR	-36,502
rs10514573	6.55E-04	0.211	0.927	0.072	16	82,011,352	*CDH13*	NM_001257.3	intron	-66,231
rs4732434	6.56E-04	-0.211	0.927	0.082	7	81,664,945	*CACNA2D1*	ENST00000262765	intron	0
rs9692165	6.56E-04	-0.211	0.927	0.082	7	81,681,327	*CACNA2D1*	NM_000722.2	intron	-43,466
rs11676611	6.57E-04	-0.211	0.927	0.049	2	143,845,025	*ARHGAP15*	NM_018460	intron	0
rs2467364	6.57E-04	0.211	0.927	0.101	15	34,539,137	*ATPBD4*	NM_080650	upstream	913,481
rs16878934	6.58E-04	0.211	0.927	0.098	7	34,497,380	*AAA1*	NM_207289	intron	0
rs10244070	6.58E-04	-0.212	0.927	0.095	7	78,241,006	*MAGI2*	NM_012301	intron	0
rs11896499	6.59E-04	0.211	0.927	0.159	2	192,505,280	*SDPR*	NM_004657	upstream	85,054
rs6460929	6.60E-04	0.211	0.927	0.332	7	12,339,607	*TMEM106B*	NM_018374.2	flanking_3UTR	-96,196
rs2942578	6.60E-04	-0.211	0.927	0.154	12	17,342,023	---	ENST00000380420	intron	0
rs338344	6.61E-04	-0.211	0.927	0.245	15	66,057,434	*PIAS1*	NM_016166.1	flanking_5UTR	-76,192
rs10820217	6.62E-04	-0.211	0.927	0.393	9	104,445,904	---	ENST00000374801	intron	0
rs12592075	6.63E-04	0.211	0.927	0.476	15	97,654,327	*LRRC28*	NM_144598.2	intron	-8,648
rs4671282	6.68E-04	-0.210	0.927	0.446	2	56,615,130	*EFEMP1*	NM_004105.2	flanking_5UTR	-611,448
rs11203190	6.68E-04	-0.210	0.927	0.226	21	42,158,582	*PRDM15*	NM_022115.2	intron	-1,896
rs16954325	6.70E-04	-0.210	0.927	0.105	18	8,813,577	*KIAA0802*	NM_015210	intron	0
rs596235	6.70E-04	0.210	0.927	0.440	22	19,313,592	*MED15*	NM_015889	downstream	41,674
rs10518033	6.73E-04	0.210	0.927	0.105	4	170,184,633	*CBR4*	NM_032783	upstream	16,636
rs10461608	6.73E-04	0.210	0.927	0.083	5	55,863,021	*MGC33648*	NM_153706.2	flanking_5UTR	-377,836
rs2124296	6.73E-04	0.214	0.927	0.252	8	63,961,452	*FAM77D*	NM_173688	intron	0
rs7971358	6.74E-04	-0.210	0.927	0.283	12	31,018,427	*DDX11*	NM_030655.2	flanking_5UTR	-99,650
rs13334795	6.74E-04	-0.210	0.927	0.109	16	83,524,062	*CRISPLD2*	NM_031476.1	flanking_3UTR	-23,447
rs1105263	6.77E-04	-0.210	0.927	0.053	3	43,420,133	*TMEM16K*	NM_018075.2	intron	-28,974
rs1055234	6.77E-04	-0.210	0.927	0.326	19	59,349,813	*CNOT3*	NM_014516	intron	0
rs4938144	6.78E-04	-0.211	0.927	0.064	11	114,223,280	*FAM55B*	NM_182495.3	flanking_3UTR	-138,706
rs1198416	6.78E-04	-0.211	0.927	0.247	14	81,992,678	*SEL1L*	NM_005065.3	flanking_5UTR	-922,792
rs10518707	6.79E-04	0.211	0.927	0.467	15	65,152,676	*SMAD3*	NM_005902.3	intron	-6,924
rs7740653	6.80E-04	-0.212	0.927	0.297	6	67,447,748	---	ENST00000365187	downstream	47,707
rs4848905	6.81E-04	-0.211	0.927	0.202	2	124,470,985	*CNTNAP5*	NM_138996	upstream	28,349
rs12326169	6.82E-04	0.210	0.927	0.429	18	13,632,721	*C18orf1*	NM_001003674.1	intron	-637
rs240446	6.82E-04	0.212	0.927	0.051	21	10,000,969	*TPTE*	NM_199261	intron	0
rs1201465	6.82E-04	0.211	0.927	0.454	X	135,938,515	*GPR101*	NM_054021.1	flanking_3UTR	-1,458
rs4345110	6.84E-04	0.210	0.927	0.365	3	133,078,499	*CPNE4*	NM_130808	intron	0
rs6102391	6.84E-04	0.210	0.927	0.065	20	39,441,896	*EMILIN3*	NM_052846.1	flanking_5UTR	-12,984
rs7356053	6.85E-04	-0.210	0.927	0.163	3	188,127,037	*ST6GAL1*	NM_173217	upstream	4,173
rs681634	6.85E-04	-0.210	0.927	0.219	5	98,959,050	*CHD1*	NM_001270.2	flanking_5UTR	-668,912
rs17154865	6.86E-04	-0.210	0.927	0.208	7	107,483,525	*LAMB4*	NM_007356.1	coding	[212/163]
rs17098533	6.86E-04	-0.210	0.927	0.199	14	61,026,753	*PRKCH*	NM_006255.3	intron	-4,626
rs10763801	6.87E-04	0.212	0.927	0.339	10	30,888,731	*LYZL2*	NM_183058	downstream	51,990

rs1513870	6.88E-04	0.210	0.927	0.212	2	177,424,377	*HNRPA3*	NM_194247.1	flanking_5UTR	-361,397
rs6108228	6.89E-04	0.210	0.927	0.225	20	8,926,063	*PLCB4*	NM_000933.2	flanking_5UTR	-98,869
rs636393	6.90E-04	-0.212	0.927	0.269	6	138,049,223	*TNFAIP3*	NM_006290.2	flanking_5UTR	-181,051
rs12170046	6.90E-04	-0.210	0.927	0.127	22	33,461,614	*ISX*	NM_001008494	upstream	330,515
rs840706	6.91E-04	0.210	0.927	0.226	2	177,400,818	*HNRPA3*	NM_194247.1	flanking_5UTR	-384,956
rs1792543	6.91E-04	-0.215	0.927	0.371	11	15,209,020	---	ENST00000379556	intron	0
rs2024860	6.92E-04	-0.210	0.927	0.109	1	41,334,830	*SCMH1*	NM_012236.1	intron	-16,712
rs7857382	6.94E-04	-0.210	0.927	0.210	9	122,381,010	*CDK5RAP2*	NM_018249.4	intron	-1,009
rs17079623	6.94E-04	0.210	0.927	0.190	18	64,544,220	*TXNDC10*	NM_019022.3	flanking_5UTR	-10,887
rs1737487	6.95E-04	0.210	0.927	0.120	1	165,790,011	*CREG1*	NM_003851.2	flanking_5UTR	-331
rs9932414	6.95E-04	0.211	0.927	0.234	16	66,777,470	*NFATC3*	NM_173164.1	intron	-2,700
rs6540239	6.95E-04	0.210	0.927	0.127	16	84,576,362	*IRF8*	NM_002163.2	flanking_3UTR	-62,649
rs2346064	6.98E-04	0.210	0.927	0.344	18	70,364,396	*CNDP1*	NM_032649.5	intron	-10,157
rs5941421	6.98E-04	-0.210	0.927	0.074	X	89,154,419	*TGIF2LX*	NM_138960.3	flanking_3UTR	-89,881
rs1626583	6.99E-04	0.212	0.927	0.444	11	131,440,838	*HNT*	NM_016522.2	intron	-80,548
rs508214	7.01E-04	-0.210	0.927	0.272	6	138,055,358	*OLIG3*	NM_175747	upstream	198,278
rs6491294	7.01E-04	-0.211	0.927	0.456	13	95,925,710	*HS6ST3*	NM_153456	intron	0
rs12209528	7.05E-04	0.210	0.927	0.304	6	67,575,835	*LOC442229*	XR_000273.2	flanking_3UTR	-1,019,742
rs4298331	7.05E-04	0.210	0.927	0.304	6	67,577,683	---	ENST00000388382	downstream	656,148
rs11074721	7.05E-04	0.210	0.927	0.380	16	25,692,399	*ZNF694*	NM_001012981.2	flanking_5UTR	-516,056
rs2941318	7.08E-04	0.211	0.927	0.386	16	9,220,283	---	ENST00000363598	downstream	345,465
rs11998040	7.09E-04	-0.209	0.927	0.433	8	84,221,577	*LOC138046*	NM_173848.3	flanking_5UTR	-1,036,564
rs7342253	7.09E-04	-0.210	0.927	0.053	11	108,208,507	*DDX10*	NM_004398.2	intron	-5,876
rs17786098	7.10E-04	0.209	0.927	0.100	3	1,471,821	*CNTN6*	ENST00000333351	downstream	140,637
rs626580	7.10E-04	0.211	0.927	0.233	19	15,663,751	*CYP4F12*	NM_023944.1	intron	-3,995
rs17222108	7.11E-04	0.209	0.927	0.082	9	14,881,600	---	ENST00000380875	intron	0
rs9358292	7.14E-04	0.209	0.927	0.245	6	20,103,710	*ID4*	NM_001546	downstream	154,816
rs4288125	7.15E-04	0.214	0.927	0.287	5	29,642,372	*CDH6*	NM_004932.2	flanking_5UTR	-1,587,181
rs11229898	7.15E-04	0.210	0.927	0.489	11	58,786,061	*OR5AN1*	NM_001004729.1	flanking_5UTR	-102,447
rs17267442	7.16E-04	-0.209	0.927	0.183	1	209,339,441	*KCNH1*	NM_172362	intron	0
rs10040841	7.17E-04	0.209	0.927	0.208	5	171,295,267	*FBXW11*	NM_033645	intron	0
rs10475994	7.17E-04	0.209	0.927	0.208	5	171,305,820	*FBXW11*	NM_033645	intron	0
rs11125013	7.18E-04	-0.211	0.927	0.327	2	45,318,939	---	ENST00000378479	intron	0
rs2787713	7.18E-04	-0.209	0.927	0.210	10	62,988,210	*TMEM26*	NM_178505	upstream	105,365
rs399150	7.19E-04	-0.209	0.927	0.237	3	7,866,227	*GRM7*	NM_000844	downstream	163,113
rs2118735	7.19E-04	-0.209	0.927	0.255	5	99,110,256	*UNQ1912*	NM_198507.1	flanking_5UTR	-788,767
rs9611602	7.19E-04	-0.210	0.927	0.104	22	40,238,351	*ACO2*	NM_001098.2	intron	-433
rs2492990	7.20E-04	0.209	0.927	0.319	1	239,322,204	*RGS7*	NM_002924.2	intron	-6,385
rs12151613	7.20E-04	0.209	0.927	0.239	2	111,325,856	*ACOXL*	NM_018308.1	intron	-10,403
rs10434287	7.20E-04	0.210	0.927	0.278	4	179,786,647	*AGA*	NM_000027.2	flanking_5UTR	-1,186,062
rs11877588	7.20E-04	0.209	0.927	0.058	18	24,817,492	*CDH2*	NM_001792.2	flanking_5UTR	-806,303
rs6592387	7.21E-04	-0.209	0.927	0.196	11	86,972,196	*FLJ22104*	NM_022918.2	flanking_3UTR	-259,976
rs7929162	7.21E-04	-0.209	0.927	0.196	11	86,972,967	*TMEM135*	NM_022918	downstream	260,751
rs13127074	7.23E-04	-0.209	0.927	0.304	4	181,598,174	*MGC45800*	NM_178838.3	flanking_3UTR	-1,701,623
rs9410002	7.23E-04	0.209	0.927	0.382	9	136,870,777	*COL5A1*	NM_000093.2	intron	-3,047
rs1105237	7.23E-04	0.209	0.927	0.312	18	23,248,050	*CHST9*	NM_031422.1	flanking_5UTR	-228,873
rs11647231	7.24E-04	0.209	0.927	0.400	16	9,210,819	---	ENST00000363598	downstream	354,929
rs6757997	7.27E-04	0.209	0.927	0.351	2	56,749,828	*VRK2*	NM_006296	upstream	1,377,396
rs473315	7.27E-04	-0.209	0.927	0.409	9	15,339,934	*C9orf52*	NM_152574.1	flanking_5UTR	-42,690

rs4895631	7.29E-04	0.209	0.927	0.243	6	144,470,080	*SF3B5*	NM_031287.2	flanking_5UTR	-11,633
rs12462307	7.29E-04	0.209	0.927	0.342	19	63,476,544	*ZNF8*	NM_021089.1	flanking_5UTR	-5,586
rs3779757	7.30E-04	-0.209	0.927	0.290	8	72,916,294	*MSC*	NM_005098.2	flanking_3UTR	-56
rs3013794	7.31E-04	0.211	0.927	0.059	10	47,155,597	---	ENST00000354683	upstream	43,103
rs960209	7.32E-04	-0.210	0.927	0.355	8	112,242,261	---	ENST00000364160	downstream	987,835
rs4248148	7.33E-04	0.212	0.927	0.134	6	30,850,113	---	ENST00000324771	intron	0
rs1000058	7.33E-04	0.209	0.927	0.063	7	131,747,556	*PLXNA4B*	NM_181775.2	flanking_3UTR	-72,505
rs4699029	7.34E-04	0.210	0.927	0.084	4	103,579,184	*NFKB1*	NM_003998	upstream	62,334
rs11949241	7.34E-04	0.210	0.927	0.143	5	158,989,077	*IL12B*	NM_002187	upstream	299,018
rs12902896	7.34E-04	-0.209	0.927	0.156	15	75,596,205	*LINGO1*	NM_032808	downstream	97,236
rs1559018	7.35E-04	-0.209	0.927	0.384	3	191,779,777	*IL1RAP*	NM_134470.2	intron	-14,941
rs7966115	7.36E-04	0.209	0.927	0.102	12	24,090,469	*SOX5*	NM_006940.4	intron	-96,565
rs994377	7.40E-04	0.209	0.927	0.283	6	67,448,299	*LOC442229*	XR_000273.2	flanking_3UTR	-892,206
rs2192055	7.40E-04	-0.209	0.927	0.226	7	21,029,351	*LOC222901*	XR_000588.1	flanking_3UTR	-146,162
rs11863425	7.41E-04	-0.209	0.927	0.091	16	83,517,443	*ZDHHC7*	ENST00000371878	downstream	483,852
rs12121863	7.42E-04	0.209	0.927	0.188	1	196,052,043	*C1orf53*	NM_001024594	upstream	86,357
rs7582028	7.43E-04	0.209	0.927	0.368	2	56,492,360	*EFEMP1*	NM_004105.2	flanking_5UTR	-488,678
rs1296064	7.48E-04	-0.209	0.927	0.069	3	103,842,880	*ZPLD1*	NM_175056.1	flanking_3UTR	-161,505
rs17215895	7.49E-04	-0.209	0.927	0.083	3	6,154,757	*GRM7*	NM_181875.1	flanking_5UTR	-723,170
rs2234962	7.50E-04	0.209	0.927	0.078	10	121,419,623	*BAG3*	NM_004281.3	coding	[270/56]
rs12203012	7.52E-04	0.209	0.927	0.303	6	67,564,026	---	ENST00000388382	downstream	669,805
rs12197795	7.53E-04	0.208	0.927	0.308	6	149,335,201	*UST*	NM_005715	intron	0
rs9956748	7.53E-04	-0.210	0.927	0.164	18	56,625,830	---	ENST00000328480	upstream	12,701
rs721651	7.54E-04	-0.208	0.927	0.109	1	41,335,916	*SCMH1*	NM_012236	intron	0
rs11633447	7.55E-04	0.208	0.927	0.089	15	96,568,260	*FLJ39743*	ENST00000332908	downstream	232,123
rs4822063	7.55E-04	-0.208	0.927	0.339	22	40,606,688	*SREBF2*	NM_004599.2	coding	[22/254]
rs9537854	7.56E-04	0.208	0.927	0.120	13	57,378,591	*PCDH17*	NM_014459.2	flanking_3UTR	-180,435
rs7640146	7.57E-04	0.210	0.927	0.061	3	124,093,319	*DIRC2*	NM_032839.1	flanking_3UTR	-11,868
rs7884299	7.57E-04	-0.209	0.927	0.412	X	7,468,791	*STS*	NM_000351	downstream	185,940
rs11753204	7.58E-04	0.208	0.927	0.303	6	67,598,300	---	ENST00000365187	upstream	102,781
rs11592006	7.58E-04	-0.209	0.927	0.111	10	11,696,606	*ECHDC3*	ENST00000331351	intron	0
rs1938596	7.58E-04	-0.208	0.927	0.440	11	58,138,262	*ZFP91*	NM_053023.2	intron	-16
rs2515362	7.58E-04	-0.208	0.927	0.440	11	58,149,527	*CNTF*	NM_000614.2	3UTR	[955/251]
rs8041923	7.58E-04	-0.210	0.927	0.493	15	97,656,931	*LRRC28*	NM_144598	intron	0
rs825842	7.58E-04	0.208	0.927	0.446	16	72,082,738	*C16orf47*	NM_207385.1	flanking_5UTR	-346,891
rs12705771	7.59E-04	-0.208	0.927	0.105	7	78,241,124	*MAGI2*	NM_012301.3	intron	-146,633
rs4797876	7.64E-04	0.208	0.927	0.212	18	145,566	*USP14*	NM_005151	upstream	2,917
rs2192839	7.65E-04	0.208	0.927	0.310	7	12,293,455	---	ENST00000275358	downstream	43,581
rs10265857	7.65E-04	-0.208	0.927	0.205	7	68,305,802	---	ENST00000363319	downstream	77,503
rs10516601	7.66E-04	0.210	0.927	0.354	4	115,474,727	*UGT8*	NM_003360.2	flanking_5UTR	-288,245
rs7819498	7.66E-04	0.208	0.927	0.074	8	105,628,090	*LRP12*	NM_013437.2	intron	-14,723
rs9564006	7.66E-04	-0.211	0.927	0.371	13	62,534,681	---	ENST00000365608	upstream	721,254
rs11931944	7.67E-04	0.208	0.927	0.181	4	125,939,881	*ANKRD50*	NM_020337.1	flanking_5UTR	-88,499
rs10085651	7.67E-04	0.212	0.927	0.096	7	80,230,307	*SEMA3C*	NM_006379	intron	0
rs5028648	7.67E-04	-0.210	0.927	0.098	12	118,973,019	*CCDC64*	NM_207311.1	intron	-10,878
rs197023	7.67E-04	-0.208	0.927	0.402	X	109,855,043	*CHRDL1*	NM_145234.2	intron	-3,632
rs2271148	7.68E-04	-0.208	0.927	0.132	1	210,979,791	*C1orf48*	NM_015471.2	intron	-225
rs7992197	7.69E-04	0.209	0.927	0.200	13	87,294,807	*SLITRK5*	NM_015567	downstream	164,935
rs6870397	7.70E-04	-0.208	0.927	0.223	5	101,319,859	*SLCO4C1*	NM_180991.4	flanking_3UTR	-277,730

rs4272111	7.70E-04	-0.208	0.927	0.223	5	101,347,769	*SLCO4C1*	NM_180991.4	flanking_3UTR	-249,820
rs2062545	7.70E-04	0.208	0.927	0.105	16	55,261,626	*MT1H*	NM_005951.1	intron	-293
rs6836436	7.71E-04	0.209	0.927	0.091	4	75,449,794	*EREG*	NM_001432.1	5UTR	[70/95]
rs6816145	7.71E-04	-0.208	0.927	0.136	4	164,638,444	*FLJ11184*	NM_018352.1	intron	-3,038
rs6892818	7.71E-04	-0.208	0.927	0.346	5	10,336,490	*CMBL*	ENST00000296658	intron	0
rs1510498	7.71E-04	0.209	0.927	0.277	7	118,003,972	*ANKRD7*	NM_019644.1	flanking_3UTR	-333,999
rs10511052	7.72E-04	0.208	0.927	0.152	3	76,808,593	*ROBO2*	NM_002942.1	flanking_5UTR	-364,028
rs6869688	7.72E-04	-0.209	0.927	0.422	5	158,815,605	*IL12B*	NM_002187.2	flanking_5UTR	-125,546
rs7098146	7.72E-04	-0.208	0.927	0.366	10	132,769,842	*TCERG1L*	NM_174937.1	flanking_3UTR	-10,803
rs29605	7.72E-04	0.208	0.927	0.237	16	8,355,922	*MGC2654*	NM_024109.1	flanking_5UTR	-267,127
rs2831451	7.73E-04	-0.209	0.927	0.273	21	28,362,183	*C21orf94*	NM_145180.2	flanking_3UTR	-45,039
rs1368061	7.74E-04	0.208	0.927	0.460	2	137,585,239	*HNMT*	NM_006895.2	flanking_5UTR	-853,039
rs2262425	7.76E-04	-0.210	0.927	0.081	2	238,648,868	*SCLY*	NM_016510.3	intron	-6,011
rs11135114	7.77E-04	-0.210	0.927	0.268	5	160,119,699	*PTTG1*	NM_004219.2	flanking_3UTR	-331,375
rs3008962	7.77E-04	-0.209	0.927	0.082	X	40,082,485	*BCOR*	NM_020926.2	flanking_5UTR	-235,487
rs845670	7.79E-04	0.208	0.927	0.143	6	167,143,089	---	ENST00000366867	intron	0
rs1946260	7.80E-04	0.208	0.927	0.163	5	66,310,808	*LOC375449*	NM_198828.1	intron	-7,220
rs6892152	7.80E-04	-0.208	0.927	0.221	5	98,982,133	*CHD1*	NM_001270.2	flanking_5UTR	-691,995
rs4073871	7.80E-04	-0.208	0.927	0.197	9	20,018,452	*MLLT3*	NM_004529	downstream	316,516
rs12913897	7.80E-04	0.208	0.927	0.485	15	97,655,543	*LRRC28*	NM_144598	intron	0
rs4812849	7.80E-04	0.208	0.927	0.071	20	42,744,502	*WISP2*	NM_003881	upstream	32,798
rs7045192	7.81E-04	-0.208	0.927	0.087	9	90,238,933	*SPIN*	NM_006717.1	intron	-7,607
rs1866646	7.82E-04	-0.209	0.927	0.255	2	240,718,913	*MYEOV2*	NM_138336	intron	0
rs6569759	7.82E-04	0.208	0.927	0.400	6	132,174,809	*ENPP1*	NM_006208.1	intron	-3,701
rs10444245	7.82E-04	0.211	0.927	0.341	11	11,355,274	*GALNTL4*	NM_198516.1	intron	-31
rs12901499	7.82E-04	0.208	0.927	0.457	15	65,157,499	*SMAD3*	NM_005902.3	intron	-11,747
rs991230	7.83E-04	0.208	0.927	0.122	1	168,985,811	*PRRX1*	NM_022716.2	flanking_3UTR	-10,646
rs7553354	7.83E-04	-0.208	0.927	0.078	1	242,111,433	*AKT3*	NM_005465.3	flanking_5UTR	-38,257
rs12520919	7.83E-04	-0.208	0.927	0.460	5	174,241,816	*FLJ16171*	NM_001004348	downstream	113,061
rs2928389	7.83E-04	0.208	0.927	0.160	8	63,626,273	*FAM77D*	NM_173688.1	intron	-28,379
rs11235204	7.84E-04	-0.208	0.927	0.216	11	86,953,503	*FLJ22104*	NM_022918.2	flanking_3UTR	-241,283
rs1878121	7.84E-04	-0.208	0.927	0.152	12	71,784,014	*TRHDE*	NM_013381.1	flanking_3UTR	-438,325
rs2602923	7.84E-04	-0.208	0.927	0.139	15	34,678,546	*C15orf41*	NM_032499.3	flanking_5UTR	-41,552
rs1889463	7.85E-04	-0.209	0.927	0.358	1	106,455,145	*PRMT6*	NM_018137.1	flanking_5UTR	-945,787
rs7722035	7.85E-04	-0.208	0.927	0.415	5	142,036,157	*FGF1*	NM_000800	intron	0
rs4888041	7.85E-04	-0.208	0.927	0.167	16	78,260,074	*MAF*	NM_001031804	upstream	67,962
rs1364339	7.86E-04	-0.209	0.927	0.165	16	78,257,468	*MAF*	NM_005360.3	flanking_5UTR	-65,356
rs5941420	7.86E-04	-0.208	0.927	0.085	X	89,150,023	*TGIF2LX*	NM_138960	downstream	85,485
rs12402425	7.88E-04	-0.209	0.927	0.068	1	71,292,605	*PTGER3*	NM_000957.2	flanking_5UTR	-6,526
rs1731494	7.88E-04	-0.208	0.927	0.258	12	30,349,530	*IPO8*	NM_006390.2	flanking_3UTR	-323,659
rs9898469	7.90E-04	-0.208	0.927	0.218	17	74,797,272	*HRNBP3*	NM_001025448	intron	0
rs10981895	7.91E-04	-0.208	0.927	0.117	9	97,559,604	*LOC375748*	NM_001010895	upstream	118,200
rs1077960	7.91E-04	-0.208	0.927	0.326	10	49,368,119	*ARHGAP22*	NM_021226	intron	0
rs11229896	7.91E-04	0.208	0.927	0.493	11	58,783,615	---	ENST00000387068	upstream	44,867
rs7741054	7.92E-04	-0.208	0.927	0.275	6	149,349,377	*UST*	NM_005715.1	intron	-21,985
rs956506	7.92E-04	0.208	0.927	0.195	20	15,556,581	*C20orf133*	NM_001033086.1	intron	-128,089
rs13055293	7.93E-04	0.208	0.927	0.092	22	33,162,523	*ISX*	ENST00000386687	upstream	731,616
rs3751295	7.95E-04	-0.208	0.927	0.150	14	61,054,490	*PRKCH*	NM_006255	intron	0
rs16846748	7.96E-04	0.208	0.927	0.122	3	152,920,937	*AADACL2*	NM_207365.1	flanking_5UTR	-13,468

rs4484395	7.96E-04	-0.208	0.927	0.353	5	10,169,479	*LOC134145*	NM_199133.1	flanking_3UTR	-109,966
rs1149392	7.97E-04	0.208	0.927	0.361	1	158,694,812	*SLAMF6*	NM_052931.3	flanking_3UTR	-26,632
rs4606010	7.97E-04	-0.208	0.927	0.053	7	49,184,188	*VWC2*	NM_198570	upstream	599,610
rs2039951	7.97E-04	-0.208	0.927	0.219	13	29,392,759	*LOC440131*	NM_001010918	upstream	15,909
rs1328650	7.97E-04	0.208	0.927	0.097	13	35,610,330	*DCAMKL1*	NM_004734.2	flanking_5UTR	-6,887
rs6099656	7.97E-04	-0.208	0.927	0.353	20	55,497,673	*HMG1L1*	NM_001008735.1	flanking_5UTR	-184
rs4948367	7.98E-04	-0.208	0.927	0.139	10	61,243,392	*CCDC6*	NM_005436	intron	0
rs12133463	7.99E-04	-0.207	0.927	0.101	1	91,928,796	*TGFBR3*	NM_003243.2	intron	-5,021
rs9820464	7.99E-04	-0.209	0.927	0.148	3	3,053,840	*CNTN4*	NM_175613.1	intron	-23
rs2394401	7.99E-04	0.208	0.927	0.137	6	30,832,409	*IER3*	NM_052815.1	flanking_5UTR	-12,106
rs1934221	7.99E-04	0.207	0.927	0.219	6	149,416,298	*UST*	NM_005715.1	intron	-20,364
rs13376351	8.00E-04	-0.208	0.927	0.071	1	63,162,370	*ATG4C*	NM_032852	downstream	58,959
rs1513376	8.00E-04	0.208	0.927	0.332	3	133,069,325	*CPNE4*	NM_130808	intron	0
rs10752209	8.00E-04	0.207	0.927	0.197	10	10,926,250	*CUGBP2*	NM_001025077.1	flanking_5UTR	-161,040
rs6135546	8.00E-04	0.211	0.927	0.223	20	15,738,136	*C20orf133*	NM_001033087.1	intron	-53,254
rs6743614	8.02E-04	0.208	0.927	0.284	2	227,686,137	*COL4A4*	NM_000092.3	intron	-1,451
rs7973859	8.02E-04	0.209	0.927	0.428	12	3,855,485	*PARP11*	NM_020367.3	flanking_5UTR	-2,622
rs6704467	8.03E-04	-0.207	0.927	0.386	1	161,631,136	*CDCA1*	NM_031423.2	flanking_3UTR	-38,964
rs8086883	8.03E-04	0.210	0.927	0.091	18	2,856,490	*EMILIN2*	NM_032048.2	intron	-18,472
rs7275269	8.04E-04	-0.207	0.927	0.053	21	37,062,529	*HLCS*	NM_000411	intron	0
rs12127030	8.06E-04	-0.208	0.927	0.176	1	41,410,985	*SCMH1*	NM_001031694.1	intron	-11,352
rs3019793	8.06E-04	0.208	0.927	0.416	11	128,820,796	*BARX2*	NM_003658.3	intron	-2,772
rs7915846	8.07E-04	-0.207	0.927	0.397	10	77,234,566	*C10orf11*	ENST00000297703	downstream	722,708
rs3852587	8.07E-04	-0.207	0.927	0.100	12	119,024,673	*RAB35*	NM_006861.4	intron	-1,340
rs3934476	8.08E-04	-0.207	0.927	0.283	5	74,278,608	*GCNT4*	NM_016591.1	flanking_3UTR	-80,437
rs10518705	8.08E-04	0.207	0.927	0.476	15	65,150,337	*SMAD3*	NM_005902.3	intron	-4,585
rs12002921	8.09E-04	-0.208	0.927	0.171	9	104,315,564	---	ENST00000374800	upstream	64,904
rs2293471	8.10E-04	0.208	0.927	0.223	2	178,195,975	*FLJ13946*	NM_152275.2	flanking_5UTR	-4,035
rs3912454	8.10E-04	0.207	0.927	0.442	9	7,615,060	*C9orf123*	NM_033428.1	flanking_3UTR	-171,430
rs126092	8.10E-04	-0.208	0.927	0.279	22	40,508,387	*FLJ22349*	NM_024821.2	flanking_5UTR	-18,237
rs2092545	8.10E-04	0.207	0.927	0.149	X	83,518,130	*CXorf43*	NM_144657.2	intron	-14,854
rs2256248	8.11E-04	0.207	0.927	0.098	2	124,150,030	*CNTNAP5*	NM_138996.1	flanking_5UTR	-349,304
rs13340606	8.11E-04	-0.208	0.927	0.193	8	3,940,031	---	ENST00000387211	downstream	394,530
rs2248385	8.11E-04	0.208	0.927	0.344	12	20,077,657	*PDE3A*	NM_000921.3	flanking_5UTR	-335,807
rs7225197	8.11E-04	0.207	0.927	0.254	17	61,627,721	*APOH*	NM_000042	downstream	10,856
rs11645728	8.12E-04	0.208	0.927	0.451	16	86,113,589	*ZCCHC14*	NM_015144	upstream	30,623
rs9874952	8.13E-04	0.207	0.927	0.384	3	88,464,159	*C3orf38*	NM_173824	downstream	174,358
rs16823994	8.14E-04	0.207	0.927	0.136	2	183,689,693	*NUP35*	ENST00000366147	upstream	4,303
rs7077535	8.14E-04	-0.207	0.927	0.154	10	20,349,314	*PLXDC2*	NM_032812.7	intron	-18,393
rs1160085	8.17E-04	-0.207	0.927	0.471	2	56,618,554	*VRK2*	NM_006296	upstream	1,508,670
rs17746436	8.17E-04	0.207	0.927	0.072	8	96,522,881	*C8orf37*	NM_177965	upstream	172,287
rs12315751	8.17E-04	-0.207	0.927	0.125	12	128,339,139	*KIAA1944*	NM_133448.1	intron	-48,993
rs7417542	8.18E-04	0.208	0.927	0.270	1	19,007,801	*TAS1R2*	NM_152232.1	flanking_3UTR	-30,879
rs6728372	8.18E-04	0.207	0.927	0.351	2	56,753,059	*VRK2*	NM_006296	upstream	1,374,165
rs1877268	8.18E-04	0.207	0.927	0.156	3	170,104,743	*EVI1*	NM_005241	downstream	180,501
rs4737627	8.19E-04	0.207	0.927	0.160	8	64,168,755	*TTPA*	NM_000370.2	flanking_5UTR	-7,589
rs606539	8.22E-04	-0.207	0.927	0.275	6	138,046,993	*OLIG3*	NM_175747	upstream	189,913
rs2421494	8.23E-04	0.207	0.927	0.123	1	168,982,155	*PRRX1*	NM_022716.2	flanking_3UTR	-6,990
rs13144303	8.23E-04	0.207	0.927	0.284	4	70,581,842	*UGT2A1*	NM_006798.1	flanking_5UTR	-28,339

rs907021	8.23E-04	0.208	0.927	0.381	16	9,262,257	---	ENST00000363598	downstream	303,491
rs9264904	8.24E-04	0.207	0.927	0.272	6	31,380,532	*HLA-C*	NM_002117.4	flanking_5UTR	-32,698
rs12904944	8.24E-04	0.207	0.927	0.371	15	65,148,828	*SMAD3*	NM_005902.3	intron	-3,076
rs694088	8.25E-04	0.210	0.927	0.186	12	33,969,842	---	ENST00000384299	upstream	2,160
rs326416	8.27E-04	-0.207	0.927	0.223	3	7,872,016	*GRM7*	NM_000844.2	flanking_3UTR	-113,799
---	8.27E-04	0.207	0.927	0.071	4	103,603,473	*NFKB1*	NM_003998	upstream	38,045
rs1396782	8.27E-04	0.207	0.927	0.458	18	59,314,905	*SERPINB5*	NM_002639.2	intron	-2,428
rs2100405	8.28E-04	0.207	0.927	0.062	2	108,257,528	*SULT1C3*	NM_001008743	downstream	9,289
rs7193350	8.28E-04	-0.207	0.927	0.252	16	74,962,057	*CNTNAP4*	NM_138994.1	intron	-15,148
rs284939	8.29E-04	0.207	0.927	0.264	16	75,669,077	*CNTNAP4*	NM_138994	downstream	582,481
rs4239214	8.29E-04	-0.207	0.927	0.274	17	23,352,019	*NLK*	NM_016231.2	flanking_5UTR	-41,282
rs6733319	8.31E-04	0.207	0.927	0.408	2	210,173,803	*MAP2*	NM_031845.1	intron	-20,734
rs2523500	8.31E-04	-0.207	0.927	0.299	6	31,626,333	*NFKBIL1*	NM_005007.2	intron	-2,138
rs11184213	8.32E-04	0.208	0.927	0.451	1	104,920,915	---	ENST00000332554	downstream	818,112
rs2515346	8.33E-04	-0.208	0.927	0.440	11	58,083,974	*LPXN*	NM_004811.1	intron	-4,230
rs9803864	8.36E-04	0.207	0.927	0.289	1	42,652,324	*FAM80A*	NM_173642	intron	0
rs7210857	8.36E-04	0.207	0.927	0.147	17	12,556,696	*MYOCD*	NM_153604.1	intron	-2,865
rs16896069	8.37E-04	0.207	0.927	0.156	5	66,300,797	*LOC375449*	NM_198828.1	intron	-17,231
rs11789818	8.38E-04	-0.207	0.927	0.272	9	106,587,051	*ABCA1*	NM_005502	intron	0
rs7190139	8.39E-04	0.207	0.927	0.386	16	9,263,812	*PRO0149*	NM_014117.2	flanking_3UTR	-142,756
rs8047149	8.39E-04	0.207	0.927	0.096	16	83,921,816	*KIAA0182*	NM_014615	upstream	282,609
rs11583319	8.40E-04	0.207	0.927	0.219	1	195,822,483	*DENND1B*	NM_144977	intron	0
rs252668	8.41E-04	-0.208	0.927	0.280	5	128,655,693	*ADAMTS19*	NM_133638.2	flanking_5UTR	-168,309
rs6963440	8.41E-04	0.207	0.927	0.224	7	47,479,635	*TNS3*	NM_022748	intron	0
rs4751158	8.44E-04	-0.207	0.927	0.451	10	131,700,143	*EBF3*	NM_001005463	upstream	48,048
rs7871303	8.46E-04	-0.207	0.927	0.074	9	90,301,922	*SPIN1*	NM_006717	downstream	18,493
rs2492988	8.47E-04	0.207	0.927	0.303	1	239,324,388	*RGS7*	NM_002924	intron	0
rs11056825	8.47E-04	0.207	0.927	0.215	12	16,256,151	*MGST1*	NM_145792.1	flanking_5UTR	-135,192
rs5997301	8.49E-04	0.206	0.927	0.159	22	26,496,402	*MN1*	NM_002430	intron	0
rs596252	8.50E-04	0.206	0.927	0.228	19	15,668,212	*CYP4F12*	NM_023944.1	intron	-28
rs2710287	8.51E-04	0.207	0.927	0.229	12	11,866,799	*ETV6*	NM_001987.3	intron	-16,542
rs11080970	8.52E-04	0.206	0.927	0.212	18	262,535	*THOC1*	NM_005131	upstream	4,486
rs10956241	8.53E-04	-0.207	0.927	0.422	8	126,408,069	*C8orf36*	NM_173685.1	intron	-30,574
rs13004840	8.54E-04	-0.206	0.927	0.127	2	155,705,193	*KCNJ3*	NM_002239.2	flanking_3UTR	-283,933
rs13014709	8.54E-04	-0.206	0.927	0.127	2	155,732,189	---	ENST00000385505	upstream	77,890
rs7576616	8.54E-04	-0.206	0.927	0.127	2	155,775,061	*KCNJ3*	NM_002239.2	flanking_3UTR	-353,801
rs17340123	8.54E-04	-0.206	0.927	0.092	5	60,890,673	*FLJ37543*	NM_173667.1	flanking_5UTR	-78,720
rs293244	8.56E-04	0.206	0.927	0.397	10	52,869,416	*PRKG1*	NM_006258.1	intron	-28,073
rs11127158	8.58E-04	-0.207	0.927	0.200	2	28,544,353	*PLB1*	NM_153021.3	flanking_5UTR	-28,133
rs210406	8.59E-04	0.206	0.927	0.373	6	99,034,993	*C6orf167*	ENST00000362072	intron	0
rs2110803	8.59E-04	0.207	0.927	0.342	7	12,352,046	---	ENST00000275358	intron	0
rs2238274	8.61E-04	0.206	0.927	0.123	14	71,527,941	*RGS6*	NM_004296.3	intron	-26,596
rs17766740	8.61E-04	-0.206	0.927	0.241	16	74,946,635	*CNTNAP4*	NM_138994.1	intron	-81
rs17234983	8.62E-04	-0.206	0.927	0.228	3	7,164,058	*GRM7*	NM_181875	intron	0
rs647108	8.62E-04	-0.207	0.927	0.353	6	138,064,233	*TNFAIP3*	NM_006290.2	flanking_5UTR	-166,041
rs993920	8.63E-04	-0.209	0.927	0.128	14	79,415,764	*NRXN3*	NM_004796	downstream	17,704
rs9994002	8.64E-04	0.206	0.927	0.056	4	115,025,374	*ARSJ*	NM_024590.2	flanking_3UTR	-15,514
rs6936990	8.64E-04	0.206	0.927	0.197	6	150,598,913	*PPP1R14C*	NM_030949.1	intron	-12,662
rs4292120	8.65E-04	-0.206	0.927	0.264	2	240,714,953	*MYEOV2*	NM_138336.1	coding	[300/77]

rs35874	8.65E-04	-0.206	0.927	0.112	3	120,183,473	*IGSF11*	NM_001015887	intron	0
rs11732304	8.66E-04	0.206	0.927	0.063	4	118,720,298	*NDST3*	NM_004784.1	flanking_5UTR	-454,650
rs10521215	8.66E-04	-0.207	0.927	0.095	17	13,149,401	*ELAC2*	NM_018127	upstream	287,352
rs10413189	8.66E-04	0.207	0.927	0.066	19	2,285,538	*SPPL2B*	NM_020172.1	intron	-63
rs207160	8.67E-04	0.209	0.927	0.291	1	55,575,999	---	ENST00000294383	upstream	122,372
rs6449794	8.67E-04	-0.206	0.927	0.248	5	65,175,231	*NLN*	NM_020726.2	flanking_3UTR	-20,082
rs11255338	8.67E-04	0.206	0.927	0.054	10	7,836,897	*KIN*	NM_012311.2	flanking_3UTR	-476
rs959228	8.67E-04	0.206	0.927	0.373	16	9,248,597	*PRO0149*	NM_014117.2	flanking_3UTR	-127,541
rs2838729	8.68E-04	0.206	0.927	0.391	21	45,143,845	*ITGB2*	NM_000211.1	intron	-340
rs17009194	8.69E-04	-0.206	0.927	0.350	3	2,202,373	*CNTN4*	NM_175607.1	intron	-85,050
rs10981787	8.69E-04	-0.206	0.927	0.116	9	97,556,710	*PTCH1*	NM_000264	upstream	246,058
rs17763056	8.69E-04	-0.207	0.927	0.073	20	16,807,716	*OTOR*	NM_020157.2	flanking_3UTR	-126,907
rs1332829	8.71E-04	0.206	0.927	0.288	1	79,316,159	*IFI44*	NM_006417.2	flanking_3UTR	-413,814
rs2498982	8.72E-04	0.206	0.927	0.478	1	62,289,271	*INADL*	NM_176878.1	coding	[45/47]
rs2224402	8.72E-04	0.206	0.927	0.442	1	231,118,964	*C1orf57*	NM_032324.1	flanking_5UTR	-34,029
rs1726866	8.72E-04	0.206	0.927	0.405	7	141,319,174	*TAS2R38*	NM_176817.1	coding	[217/784]
rs928636	8.73E-04	0.206	0.927	0.263	9	29,729,205	*LRRN6C*	NM_152570.1	flanking_5UTR	-1,019,902
rs9936071	8.73E-04	-0.206	0.927	0.055	16	16,873,033	*XYLT1*	NM_022166.2	flanking_3UTR	-235,807
rs2683260	8.74E-04	0.209	0.927	0.219	15	79,172,607	*MESDC1*	NM_022566	downstream	89,207
rs869829	8.74E-04	-0.207	0.927	0.425	X	40,045,873	*BCOR*	NM_020926.2	flanking_5UTR	-198,875
rs1569196	8.75E-04	-0.206	0.927	0.433	6	131,523,363	*AKAP7*	NM_016377.2	intron	-332
rs1039200	8.76E-04	0.206	0.927	0.409	3	11,705,846	*VGLL4*	ENST00000303592	intron	0
rs3804477	8.76E-04	-0.206	0.927	0.205	6	6,573,720	*LY86*	NM_004271.3	intron	-2,067
rs11145281	8.76E-04	-0.206	0.927	0.278	9	78,899,644	*VPS13A*	NM_001018037	upstream	82,537
rs1432219	8.77E-04	-0.208	0.927	0.417	2	137,585,306	*HNMT*	NM_006895.2	flanking_5UTR	-852,972
rs7226445	8.77E-04	0.206	0.927	0.214	18	8,345,048	*PTPRM*	NM_002845.2	intron	-11,530
rs5754597	8.78E-04	0.206	0.927	0.225	22	32,262,624	*LARGE*	NM_133642.2	intron	-28,210
rs16890712	8.79E-04	-0.207	0.927	0.326	5	33,118,123	*FLJ14054*	NM_024563.2	flanking_3UTR	-290,547
rs17056817	8.79E-04	0.206	0.927	0.071	13	37,377,710	*TRPC4*	NM_016179.1	flanking_5UTR	-35,775
rs33938520	8.79E-04	-0.206	0.927	0.172	19	4,935,588	*JMJD2B*	NM_015015	intron	0
rs664954	8.80E-04	0.206	0.927	0.053	3	128,964,008	*MGLL*	NM_001003794.1	intron	-19,314
rs12424042	8.81E-04	0.206	0.927	0.192	12	86,430,009	*C12orf50*	NM_152589.1	flanking_3UTR	-467,938
rs895312	8.82E-04	-0.206	0.927	0.105	5	172,697,004	*STC2*	NM_003714	upstream	7,892
rs12022925	8.83E-04	-0.208	0.927	0.339	1	104,883,436	*AMY1C*	NM_001008219.1	flanking_3UTR	-780,603
rs7666822	8.83E-04	-0.206	0.927	0.096	4	38,608,560	*FAM114A1*	ENST00000358869	intron	0
rs17049202	8.83E-04	0.208	0.927	0.149	4	138,119,059	---	ENST00000364398	downstream	514,720
rs12901446	8.83E-04	0.207	0.927	0.119	15	47,559,088	*FGF7*	NM_002009.2	intron	-3,552
rs2207232	8.86E-04	-0.206	0.927	0.205	6	152,381,981	*ESR1*	NM_000125.2	intron	-7,359
rs953758	8.86E-04	0.206	0.927	0.184	13	87,282,781	*SLITRK5*	NM_015567.1	flanking_3UTR	-152,909
rs2300831	8.86E-04	-0.206	0.927	0.134	14	32,275,252	*AKAP6*	NM_004274	intron	0
rs11880312	8.87E-04	0.206	0.927	0.067	19	11,158,434	*ANKRD25*	NM_015493	intron	0
rs2112027	8.90E-04	-0.206	0.927	0.442	2	56,690,125	*VRK2*	NM_006296	upstream	1,437,099
rs4888213	8.90E-04	-0.206	0.927	0.109	16	72,918,430	*PSMD7*	NM_002811.3	flanking_3UTR	-20,743
rs9514093	8.91E-04	0.206	0.927	0.060	13	102,513,669	*SLC10A2*	NM_000452.1	intron	-2,555
rs12677573	8.92E-04	-0.206	0.927	0.073	8	18,994,858	*PSD3*	NM_015310.2	flanking_5UTR	-79,382
rs4651005	8.94E-04	0.206	0.927	0.152	1	176,985,929	*RALGPS2*	NM_018037.1	intron	-24,752
rs7032123	8.94E-04	0.206	0.927	0.322	9	16,299,559	*BNC2*	NM_017637.4	flanking_3UTR	-106,843
rs7096194	8.94E-04	0.207	0.927	0.489	10	29,808,359	*SVIL*	NM_003174	intron	0
rs10009946	8.95E-04	-0.206	0.927	0.142	4	63,911,889	*SRD5A2L2*	NM_001010874	downstream	916,496

rs4282379	8.95E-04	0.208	0.927	0.310	6	95,398,030	*MANEA*	ENST00000364571	downstream	41,212
rs2891285	8.96E-04	0.206	0.927	0.076	1	185,789,297	*C1orf99*	NM_001012274.1	flanking_5UTR	-87,684
rs6542190	8.96E-04	0.206	0.927	0.266	2	111,354,534	*ACOXL*	NM_018308	intron	0
rs4917589	8.98E-04	-0.206	0.927	0.220	10	112,602,792	*PDCD4*	NM_014456.3	flanking_5UTR	-18,794
rs6973982	8.99E-04	-0.206	0.927	0.112	7	45,110,417	*TBRG4*	NM_004749.2	intron	-37
rs5754682	8.99E-04	0.206	0.927	0.062	22	32,532,284	*LARGE*	NM_133642.2	intron	-44,739
rs17804238	9.00E-04	0.207	0.927	0.098	7	79,545,323	*GNAI1*	NM_002069	upstream	56,753
rs12891099	9.00E-04	-0.205	0.927	0.116	14	47,115,542	*MAMDC1*	NM_182830.2	flanking_5UTR	-233,397
rs12894376	9.00E-04	-0.205	0.927	0.116	14	47,119,391	*MDGA2*	NM_182830	intron	0
rs7159861	9.00E-04	-0.205	0.927	0.225	14	50,214,410	*SAV1*	NM_021818.2	flanking_5UTR	-9,637
rs982116	9.01E-04	-0.205	0.927	0.458	2	56,593,154	*EFEMP1*	NM_004105.2	flanking_5UTR	-589,472
rs12386185	9.01E-04	-0.205	0.927	0.100	2	58,896,243	---	ENST00000340221	downstream	563,079
rs4386730	9.01E-04	0.205	0.927	0.389	5	135,178,106	*CXCL14*	NM_004887	upstream	235,238
rs6639176	9.02E-04	-0.206	0.927	0.227	X	12,345,242	*FRMPD4*	NM_014728.1	intron	-81,478
rs2195066	9.03E-04	-0.205	0.927	0.328	8	112,253,235	---	ENST00000364160	downstream	976,861
rs2828056	9.03E-04	-0.207	0.927	0.057	21	23,613,848	*C21orf74*	XR_001010.1	flanking_3UTR	-1,203,133
rs2948486	9.03E-04	-0.206	0.927	0.085	X	40,077,957	*BCOR*	NM_020926	upstream	255,791
rs4962653	9.04E-04	0.207	0.927	0.226	10	126,252,385	*LHPP*	NM_022126.2	intron	-39,438
rs11617065	9.04E-04	-0.205	0.927	0.216	13	29,386,370	*LOC440131*	NM_001010918.1	flanking_5UTR	-22,298
rs2202713	9.04E-04	0.206	0.927	0.124	15	47,748,678	*DTWD1*	NM_020234.3	flanking_3UTR	-25,524
rs2051086	9.08E-04	-0.205	0.927	0.250	1	49,877,788	*ELAVL4*	NM_021952.2	flanking_5UTR	-469,437
rs1463518	9.08E-04	0.207	0.927	0.335	3	133,070,463	*CPNE4*	NM_130808	intron	0
rs1925111	9.08E-04	-0.205	0.927	0.250	13	95,797,481	*UGCGL2*	NM_020121.2	flanking_5UTR	-293,843
rs2825256	9.08E-04	-0.207	0.927	0.390	21	19,285,346	*PPIA*	NM_203431	downstream	132,695
rs919001	9.09E-04	-0.205	0.927	0.357	15	29,144,430	*TRPM1*	NM_002420.3	intron	-1,141
rs7068881	9.10E-04	-0.205	0.927	0.411	10	16,878,872	*RSU1*	NM_152724.2	intron	-14,783
rs10490407	9.11E-04	0.206	0.927	0.449	2	56,600,028	*FANCL*	NM_018062	downstream	1,639,856
rs11742776	9.11E-04	-0.208	0.927	0.344	5	142,027,375	*FGF1*	NM_033136.1	intron	-18,331
rs7912641	9.11E-04	-0.205	0.927	0.062	10	7,808,227	*ITIH2*	NM_002216.2	intron	-692
rs4964243	9.11E-04	-0.206	0.927	0.391	12	107,222,024	*CMKLR1*	NM_004072.1	intron	-11,158
rs17464857	9.12E-04	0.205	0.927	0.080	1	220,829,332	*TAF1A*	NM_139352.1	intron	-360
rs17511318	9.12E-04	0.205	0.927	0.080	1	220,829,581	*TAF1A*	NM_139352.1	intron	-111
rs1909194	9.12E-04	0.205	0.927	0.080	1	220,837,326	*TAF1A*	NM_139352	upstream	7,448
rs1521825	9.12E-04	-0.206	0.927	0.405	8	84,246,987	*LOC138046*	NM_173848.3	flanking_5UTR	-1,011,154
rs12909292	9.12E-04	-0.205	0.927	0.147	15	75,625,037	*HMG20A*	NM_018200.2	flanking_3UTR	-60,037
rs624512	9.13E-04	0.207	0.927	0.224	19	15,669,890	*CYP4F12*	NM_023944.1	flanking_3UTR	-906
rs12267420	9.15E-04	0.205	0.927	0.111	10	79,459,663	*POLR3A*	NM_007055.2	flanking_5UTR	-398
rs8037412	9.15E-04	0.206	0.927	0.455	15	76,296,597	*ACSBG1*	NM_015162	intron	0
rs7200481	9.16E-04	-0.206	0.927	0.102	16	82,282,872	*CDH13*	NM_001257.3	intron	-13,305
rs2087889	9.17E-04	-0.205	0.927	0.400	11	128,961,654	*TMEM45B*	NM_138788	upstream	229,297
rs1565840	9.18E-04	-0.206	0.927	0.458	4	164,853,822	*1-Mar*	NM_017923.2	flanking_5UTR	-99,596
rs12778915	9.18E-04	0.205	0.927	0.453	10	77,287,563	*C10orf11*	NM_032024	intron	0
rs17105032	9.18E-04	0.205	0.927	0.132	14	71,504,284	*RGS6*	NM_004296.3	intron	-2,939
rs6844681	9.20E-04	0.206	0.927	0.475	4	143,466,690	*INPP4B*	NM_003866.1	intron	-11,325
rs1297083	9.21E-04	0.205	0.927	0.446	21	14,294,380	*C21orf81*	NM_153750	upstream	19,744
rs4654497	9.22E-04	-0.205	0.927	0.063	1	4,124,418	*AJAP1*	NM_018836	upstream	490,234
rs949781	9.23E-04	-0.207	0.927	0.132	2	155,778,244	---	ENST00000385504	upstream	50,292
rs1202987	9.23E-04	0.205	0.927	0.358	X	69,018,744	*EDA*	NM_001005611	intron	0
rs1037149	9.25E-04	-0.207	0.927	0.336	4	140,676,880	*SETD7*	NM_030648	intron	0

rs13286794	9.25E-04	-0.205	0.927	0.324	9	25,709,190	*TUSC1*	NM_001004125	upstream	40,871
rs2393949	9.27E-04	-0.205	0.927	0.230	10	43,334,305	*ZNF239*	NM_005674.1	flanking_3UTR	-37,496
rs11229497	9.27E-04	-0.205	0.927	0.332	11	58,022,525	*OR5B21*	NM_001005218	downstream	8,700
rs2166724	9.28E-04	-0.205	0.927	0.335	5	98,559,910	---	ENST00000363481	upstream	259,559
rs1813496	9.28E-04	-0.205	0.927	0.476	8	80,780,276	*STMN2*	NM_007029	downstream	39,345
rs11028743	9.29E-04	0.205	0.927	0.243	11	25,494,869	*LUZP2*	NM_001009909	downstream	438,089
rs6689991	9.32E-04	0.205	0.927	0.085	1	238,853,405	*GREM2*	NM_022469.3	flanking_5UTR	-11,320
rs11156740	9.32E-04	0.205	0.927	0.082	14	31,911,940	*AKAP6*	NM_004274.3	intron	-43,575
rs10884620	9.34E-04	-0.205	0.927	0.056	10	110,101,694	*SORCS1*	NM_052918.3	flanking_5UTR	-1,187,412
rs7170175	9.35E-04	-0.205	0.927	0.172	15	79,890,994	*RKHD3*	NM_032246.3	flanking_3UTR	-230,189
rs16892	9.36E-04	0.205	0.927	0.098	9	14,879,834	*FREM1*	NM_144966.3	intron	-20,078
rs4496439	9.37E-04	0.206	0.927	0.469	3	26,663,975	*LRRC3B*	NM_052953.2	intron	-24,243
rs8111852	9.37E-04	0.206	0.927	0.222	19	15,660,130	*CYP4F12*	NM_023944.1	intron	-3,123
rs7514372	9.41E-04	-0.206	0.927	0.111	1	235,545,332	*RYR2*	NM_001035	intron	0
rs2927324	9.41E-04	0.205	0.927	0.308	16	80,070,322	*CMIP*	NM_030629	intron	0
rs9973133	9.41E-04	-0.205	0.927	0.067	18	73,763,246	---	ENST00000362858	upstream	134,481
rs725750	9.42E-04	0.205	0.927	0.098	6	80,139,646	*C6orf152*	NM_181714.1	flanking_3UTR	-111,781
rs4481859	9.43E-04	-0.205	0.927	0.325	1	220,799,279	*TAF1A*	NM_139352.1	intron	-542
rs838528	9.44E-04	0.208	0.927	0.376	1	119,700,558	*HAO2*	NM_016527	upstream	12,426
rs2199351	9.44E-04	0.207	0.927	0.362	3	133,090,229	*CPNE4*	NM_130808.1	intron	-16,569
rs10182324	9.46E-04	-0.205	0.927	0.143	2	58,901,477	*FANCL*	NM_018062.2	flanking_5UTR	-579,459
rs6805151	9.47E-04	0.205	0.927	0.504	3	197,878,915	*C3orf34*	ENST00000386576	upstream	184,262
rs2038297	9.47E-04	0.205	0.927	0.101	6	80,138,095	*LCA5*	ENST00000365187	downstream	169,709
rs10234185	9.47E-04	0.205	0.927	0.297	7	12,264,836	*TMEM106B*	NM_018374.2	flanking_3UTR	-21,425
rs5751129	9.47E-04	-0.205	0.927	0.252	22	40,345,711	*D15Wsu75e*	NM_015704.1	intron	-991
rs9868927	9.49E-04	0.205	0.927	0.399	3	174,822,471	*NLGN1*	NM_014932.2	intron	-16,896
rs17473108	9.49E-04	-0.205	0.927	0.145	4	164,683,228	*1-Mar*	NM_017923.2	intron	-2,970
rs4083650	9.49E-04	-0.205	0.927	0.480	9	128,392,999	*FAM125B*	NM_001011703	downstream	83,858
rs906831	9.50E-04	0.205	0.927	0.278	2	210,206,302	*MAP2*	NM_002374.2	intron	-8,204
rs12408576	9.51E-04	-0.205	0.927	0.195	1	41,237,024	*CTPS*	NM_001905.1	intron	-1,239
rs2962403	9.51E-04	-0.205	0.927	0.464	5	160,703,373	*GABRB2*	NM_021911.1	intron	-7,019
rs4787367	9.51E-04	-0.205	0.927	0.438	16	26,255,281	*TNT*	NM_182831.1	flanking_5UTR	-730,248
rs12371422	9.54E-04	-0.204	0.927	0.087	12	3,231,207	*TSPAN9*	NM_006675.3	intron	-26,641
rs12978650	9.54E-04	0.204	0.927	0.114	19	36,067,601	*ZNF536*	NM_014717.1	flanking_3UTR	-326,796
rs17048238	9.55E-04	-0.206	0.927	0.344	1	216,828,304	*LYPLAL1*	NM_138794	upstream	585,505
rs7613317	9.55E-04	0.205	0.927	0.407	3	167,268,104	*BCHE*	NM_000055	upstream	230,160
rs34618866	9.55E-04	0.204	0.927	0.091	5	158,938,017	*IL12B*	NM_002187	upstream	247,958
rs2215035	9.56E-04	-0.204	0.927	0.150	7	14,317,342	*DGKB*	NM_145695	intron	0
rs4962651	9.57E-04	0.204	0.927	0.234	10	126,251,531	*LHPP*	ENST00000368842	intron	0
rs610354	9.57E-04	0.204	0.927	0.225	19	15,670,248	*CYP4F12*	NM_023944.1	flanking_3UTR	-1,264
rs6719808	9.58E-04	0.204	0.927	0.391	2	56,641,932	*VRK2*	ENST00000336735	upstream	35,592
rs2833459	9.60E-04	-0.205	0.927	0.173	21	31,879,636	*TIAM1*	NM_003253.1	flanking_5UTR	-26,475
rs3800845	9.62E-04	0.204	0.927	0.324	7	12,230,676	*TMEM106B*	NM_018374	intron	0
rs10512001	9.63E-04	0.208	0.927	0.234	9	73,345,941	*TMEM2*	NM_013390.1	flanking_3UTR	-142,162
rs11863338	9.63E-04	-0.205	0.927	0.093	16	83,517,208	*ZDHHC7*	NM_017740	downstream	48,365
rs1129763	9.63E-04	-0.207	0.927	0.078	19	8,027,369	*CCL25*	NM_005624.2	coding	[119/14]
rs16886683	9.64E-04	0.204	0.927	0.058	5	18,768,939	*CDH18*	NM_004934.2	flanking_3UTR	-739,959
rs7822592	9.64E-04	-0.204	0.927	0.409	8	126,437,557	*NSMCE2*	NM_173685	intron	0
rs1126107	9.64E-04	-0.204	0.927	0.121	9	27,274,363	*C9orf11*	NM_020641.1	flanking_3UTR	-304

rs6671694	9.65E-04	0.205	0.927	0.205	1	174,786,940	*PAPPA2*	NM_021936.1	intron	-4,226
rs9600121	9.65E-04	0.204	0.927	0.167	13	72,860,521	*KLF12*	NM_007249	downstream	297,629
rs8002929	9.65E-04	0.204	0.927	0.167	13	72,862,520	*KLF12*	NM_007249.3	flanking_3UTR	-295,630
rs17729143	9.67E-04	0.204	0.927	0.143	6	149,325,504	*UST*	NM_005715.1	intron	-1,735
rs2270181	9.67E-04	0.207	0.927	0.493	10	117,918,208	*GFRA1*	NM_005264	intron	0
rs7190503	9.67E-04	-0.205	0.927	0.270	16	7,408,730	*A2BP1*	NM_145892.1	intron	-85,640
rs11913533	9.68E-04	-0.205	0.927	0.060	22	47,983,493	*FLJ44385*	NM_207478.1	flanking_5UTR	-344,840
rs6877249	9.69E-04	-0.204	0.927	0.221	5	101,348,074	---	ENST00000388691	downstream	402,343
rs4371793	9.69E-04	-0.207	0.927	0.276	5	160,121,492	---	ENST00000327245	intron	0
rs4813881	9.69E-04	0.206	0.927	0.271	20	8,916,642	*PLCB1*	ENST00000386958	downstream	420,261
rs1093932	9.70E-04	0.204	0.927	0.219	1	243,692,901	---	ENST00000329504	downstream	91,261
rs2780293	9.71E-04	0.204	0.927	0.063	1	56,677,660	*PPAP2B*	NM_003713.3	flanking_3UTR	-55,361
rs602414	9.71E-04	-0.205	0.927	0.287	6	138,053,358	*TNFAIP3*	NM_006290.2	flanking_5UTR	-176,916
rs1348542	9.71E-04	0.209	0.927	0.324	10	52,888,464	*PRKG1*	NM_006258	intron	0
rs440499	9.72E-04	0.204	0.927	0.411	5	82,347,320	*MGC23909*	NM_174909.1	flanking_3UTR	-40,914
rs17545624	9.72E-04	0.204	0.927	0.123	19	36,032,592	*ZNF536*	NM_014717.1	flanking_3UTR	-291,787
rs8061856	9.74E-04	0.205	0.927	0.064	16	51,470,828	*CHD9*	NM_025134.3	flanking_5UTR	-276,511
rs4558912	9.75E-04	-0.205	0.927	0.196	4	66,521,854	*EPHA5*	NM_182472.1	flanking_5UTR	-303,750
rs1512883	9.75E-04	-0.204	0.927	0.370	13	62,449,023	*OR7E156P*	NR_002171.1	flanking_5UTR	-760,546
rs11768716	9.76E-04	0.206	0.927	0.062	7	135,547,018	*CHRM2*	NM_001006632	upstream	656,938
rs10886319	9.79E-04	-0.204	0.927	0.350	10	120,585,456	*C10orf46*	NM_153810.3	flanking_5UTR	-80,708
rs1012090	9.80E-04	0.208	0.927	0.238	7	29,001,365	*CPVL*	NM_019029.1	flanking_3UTR	-410
rs13428806	9.81E-04	0.204	0.927	0.275	2	178,284,927	*PDE11A*	NM_016953.2	intron	-75
rs1995274	9.81E-04	0.204	0.927	0.298	5	61,201,717	*FLJ37543*	NM_173667.1	flanking_3UTR	-163,598
rs2113866	9.82E-04	0.204	0.927	0.350	2	56,570,432	*EFEMP1*	NM_004105.2	flanking_5UTR	-566,750
rs2014301	9.83E-04	-0.204	0.927	0.268	8	16,951,688	*EFHA2*	NM_181723	intron	0
rs11774804	9.83E-04	-0.205	0.927	0.465	8	50,043,528	*SNAI2*	NM_003068.3	flanking_5UTR	-46,987
rs12960677	9.83E-04	-0.204	0.927	0.112	18	8,814,239	*KIAA0802*	NM_015210	intron	0
rs3008598	9.85E-04	0.205	0.927	0.290	1	220,846,571	*TAF1A*	NM_139352.1	flanking_5UTR	-16,693
rs5010075	9.85E-04	-0.204	0.927	0.205	11	86,962,289	*FLJ22104*	NM_022918.2	flanking_3UTR	-250,069
rs9554815	9.85E-04	-0.204	0.927	0.214	13	101,104,890	*ITGBL1*	ENST00000376180	intron	0
rs2726417	9.86E-04	-0.206	0.927	0.143	11	98,882,617	*CNTN5*	NM_175566.1	intron	-49,469
rs2645920	9.86E-04	0.204	0.927	0.134	13	55,357,235	---	ENST00000334406	upstream	1,443,763
rs2814030	9.87E-04	-0.205	0.927	0.208	10	63,007,799	---	ENST00000389639	intron	0
rs6694114	9.88E-04	-0.204	0.927	0.493	1	56,682,138	*PPAP2B*	NM_003713.3	flanking_3UTR	-50,883
rs11772975	9.88E-04	-0.204	0.927	0.205	7	103,408,503	*RELN*	NM_005045.2	intron	-8,311
rs7314782	9.88E-04	-0.204	0.927	0.444	12	17,315,231	*LMO3*	NM_001001395.1	flanking_5UTR	-662,940
rs5752972	9.89E-04	0.204	0.927	0.335	22	28,580,312	*MTMR3*	NM_153050	upstream	28,887
rs10902088	9.90E-04	-0.204	0.927	0.322	11	1,077,972	*AP2A2*	NM_012305.2	flanking_3UTR	-75,732
rs9482269	9.91E-04	0.204	0.927	0.353	6	123,069,560	*PKIB*	ENST00000368452	intron	0
rs3750263	9.92E-04	-0.204	0.927	0.158	8	29,036,982	*KIF13B*	NM_015254.2	intron	-54
rs855586	9.93E-04	-0.204	0.927	0.326	3	62,500,490	*CADPS*	NM_183393.1	intron	-3,194
rs1860105	9.93E-04	0.204	0.927	0.129	14	71,526,666	*RGS6*	NM_004296.3	intron	-25,321
rs7321919	9.94E-04	-0.204	0.927	0.337	13	29,193,287	*UBL3*	NM_007106.2	flanking_3UTR	-43,255
rs17177982	9.94E-04	-0.205	0.927	0.337	14	36,505,046	*SLC25A21*	NM_030631	intron	0
rs1326246	9.97E-04	-0.204	0.927	0.197	10	20,330,027	*PLXDC2*	NM_032812	intron	0
rs7900894	9.97E-04	-0.205	0.927	0.139	10	32,527,511	*EPC1*	NM_025209	downstream	70,354
rs6438476	9.99E-04	-0.204	0.927	0.120	3	120,101,550	*IGSF11*	NM_001015887.1	flanking_3UTR	-617
rs17152268	9.99E-04	0.204	0.927	0.118	10	12,876,488	*CAMK1D*	NM_020397.2	intron	-3,250

Supplementary Table 2. Top 1736 SNPs that were associated with docetaxel IC50 with p-values $<10^{-3}$ and top 180 SNPs that were significantly associated with docetaxel IC50 values with p-values $<10^{-4}$. R values represent correlation coefficients for association.

SNP ID	P value	R value	Q value	MAF	Chr	Position	GeneSymbol	RefSeq ID	Location	Location Relative to Gene (bp)
rs6044112	6.90E-07	0.305	0.346	0.172	20	16,484,414	*C20orf23*	NM_024704.3	intron	-17,460
rs6790433	1.15E-06	-0.298	0.346	0.278	3	14,455,782	*SLC6A6*	NM_003043.2	intron	-4,354
rs258671	1.17E-06	0.297	0.346	0.326	7	81,569,382	*CACNA2D1*	NM_000722.2	intron	-14,914
rs6977982	1.28E-06	0.298	0.346	0.324	7	81,552,976	*CACNA2D1*	NM_000722	intron	0
rs2586502	1.43E-06	0.295	0.346	0.332	17	45,644,069	*COL1A1*	NM_000088.2	flanking_5UTR	-10,077
rs10874322	2.00E-06	-0.291	0.346	0.135	1	82,827,387	*TTLL7*	NM_024686	downstream	1,275,912
rs4925540	2.42E-06	-0.289	0.346	0.217	1	245,281,501	*ZNF670*	NM_033213.2	intron	-12,039
rs11668609	2.84E-06	-0.287	0.346	0.319	19	24,147,333	*ZNF254*	NM_203282	downstream	44,711
rs11748684	3.09E-06	0.288	0.346	0.052	5	42,324,350	*GHR*	NM_000163	upstream	135,433
rs1944582	3.95E-06	0.283	0.346	0.380	18	44,069,415	*KIAA0427*	NM_014772.1	flanking_5UTR	-250,010
rs4416149	4.23E-06	-0.288	0.346	0.298	19	24,068,193	*ZNF254*	NM_004876.1	intron	-6,214
rs1412259	4.31E-06	0.282	0.346	0.308	9	1,446,797	---	ENST0000038224	upstream	294,368
rs7542852	4.34E-06	-0.282	0.346	0.218	1	245,293,609	*ZNF670*	NM_033213.2	intron	-14,910
rs1332004	4.50E-06	0.281	0.346	0.306	9	1,443,731	*DMRT2*	NM_181872.1	flanking_3UTR	-396,179
rs10924897	4.68E-06	-0.281	0.346	0.216	1	245,268,655	*ZNF670*	NM_033213.2	intron	-80
rs4634944	4.68E-06	-0.281	0.346	0.216	1	245,291,657	*ZNF670*	NM_033213.2	intron	-16,862
rs7547915	4.68E-06	-0.281	0.346	0.216	1	245,270,229	*ZNF670*	NM_033213.2	intron	-767
rs12141355	4.68E-06	-0.281	0.346	0.216	1	245,271,117	*ZNF670*	NM_033213	intron	0
rs7260280	5.67E-06	-0.279	0.395	0.320	19	24,114,662	*ZNF539*	NM_203282.1	flanking_3UTR	-10,169
rs1478912	5.96E-06	-0.278	0.395	0.389	1	235,687,862	*RYR2*	NM_001035.1	intron	-1,204
rs6887313	6.58E-06	0.276	0.395	0.062	5	42,340,710	*GHR*	NM_000163	upstream	119,073
rs34354271	6.64E-06	-0.276	0.395	0.301	19	24,074,112	*ZNF254*	NM_004876.1	intron	-6,470
rs923175	6.83E-06	0.276	0.395	0.404	11	12,029,079	*DKK3*	NM_013253	upstream	41,586
rs12954803	7.54E-06	-0.275	0.409	0.078	18	20,390,567	*HRH4*	NM_021624	downstream	79,000
rs2130017	7.69E-06	0.281	0.409	0.119	11	89,142,024	*TRIM49*	NM_020358.2	flanking_3UTR	-28,447
rs17624523	8.46E-06	-0.273	0.433	0.132	2	104,525,070	*POU3F3*	NM_006236.1	flanking_5UTR	-313,331
rs10423754	9.47E-06	-0.272	0.467	0.496	19	59,296,634	*OSCAR*	NM_206817.1	flanking_5UTR	-674
rs1852408	1.33E-05	-0.268	0.631	0.318	19	24,253,495	*ZNF254*	NM_203282	downstream	150,873
rs201460	1.42E-05	-0.267	0.635	0.246	7	101,528,291	*CUTL1*	NM_001913.2	intron	-790
rs7776703	1.43E-05	-0.267	0.635	0.317	7	122,646,012	*SLC13A1*	NM_022444.3	flanking_5UTR	-18,751
rs6760287	1.51E-05	-0.266	0.645	0.111	2	238,163,735	*RAB17*	NM_022449.1	intron	-114
rs10265730	1.55E-05	-0.266	0.645	0.392	7	81,561,048	*CACNA2D1*	NM_000722.2	intron	-8,896
rs10764483	1.71E-05	-0.264	0.670	0.067	10	18,756,352	*CACNB2*	NM_201590	intron	0
rs258679	1.73E-05	0.265	0.670	0.347	7	81,571,687	*CACNA2D1*	NM_000722.2	intron	-12,609
rs258668	1.77E-05	0.264	0.670	0.342	7	81,566,452	*CACNA2D1*	NM_000722	intron	0
rs1791261	1.90E-05	-0.265	0.670	0.132	18	20,389,042	*HRH4*	NM_021624	downstream	77,475
rs4731086	1.93E-05	-0.263	0.670	0.321	7	122,652,160	*SLC13A1*	NM_022444.3	flanking_5UTR	-24,899
rs1573897	2.00E-05	-0.263	0.670	0.256	1	245,281,677	*ZNF670*	NM_033213	intron	0
rs4867405	2.00E-05	-0.268	0.670	0.375	5	31,980,732	*PDZD2*	NM_178140	intron	0

rs643070	2.01E-05	0.264	0.670	0.197	9	21,358,609	*IFNA13*	NM_006900.2	flanking_5UTR	-534
rs10051562	2.09E-05	0.264	0.671	0.061	5	42,348,473	*GHR*	NM_000163.2	flanking_5UTR	-111,310
rs17000660	2.23E-05	-0.261	0.671	0.359	19	24,166,697	*ZNF539*	NM_203282.1	flanking_3UTR	-62,204
rs258665	2.30E-05	0.260	0.671	0.312	7	81,565,638	*CACNA2D1*	NM_000722	intron	0
rs1888087	2.35E-05	-0.260	0.671	0.150	20	801,435	*ANGPT4*	NM_015985.2	3UTR	[139/167]
rs12898337	2.44E-05	0.261	0.671	0.350	15	97,111,878	*IGF1R*	NM_000875.2	intron	-43,019
rs7260598	2.46E-05	-0.259	0.671	0.165	19	24,014,626	*ZNF254*	NM_203282	upstream	47,190
rs7586928	2.52E-05	-0.260	0.671	0.482	2	25,449,826	*DTNB*	NM_033148.2	flanking_3UTR	-3,790
rs4402920	2.60E-05	-0.259	0.671	0.467	3	14,453,235	---	ENST00000025370	intron	0
rs2776227	2.68E-05	-0.260	0.671	0.325	21	29,196,779	*C21orf127*	NM_182749.1	flanking_5UTR	-17,215
rs1344072	2.70E-05	-0.258	0.671	0.389	3	62,663,963	*CADPS*	NM_003716	intron	0
rs1469481	2.80E-05	0.258	0.671	0.491	2	141,381,349	*LRP1B*	NM_018557.1	intron	-567
rs10741058	2.82E-05	-0.258	0.671	0.069	10	18,756,151	*CACNB2*	NM_201570.1	intron	-25,173
rs1530129	2.87E-05	-0.257	0.671	0.134	2	104,530,267	*POU3F3*	NM_006236.1	flanking_5UTR	-308,134
rs16880939	2.91E-05	0.258	0.671	0.053	7	36,186,371	*KIAA1706*	NM_030636.2	intron	-25,035
rs374082	2.93E-05	-0.257	0.671	0.062	11	38,683,002	*C11orf74*	NM_138787	downstream	2,045,605
rs1224391	3.03E-05	0.258	0.671	0.168	9	21,381,698	*IFNA2*	NM_000605.2	flanking_5UTR	-6,302
rs17023229	3.08E-05	0.257	0.671	0.171	2	83,205,890	*LOC388965*	NM_001013648.3	flanking_5UTR	-1,165,428
rs10414075	3.15E-05	-0.256	0.671	0.341	19	24,030,291	*ZNF254*	NM_203282	upstream	31,525
rs2020059	3.19E-05	0.256	0.671	0.373	4	171,654,986	*AADAT*	NM_182662.1	flanking_5UTR	-407,039
rs2137683	3.20E-05	0.256	0.671	0.493	15	97,107,161	*IGF1R*	NM_000875	intron	0
rs10828679	3.22E-05	-0.257	0.671	0.078	10	18,751,294	*CACNB2*	NM_201570.1	intron	-20,316
rs4817258	3.24E-05	-0.256	0.671	0.326	21	29,161,249	*N6AMT1*	NM_013240	downstream	5,151
rs11145281	3.26E-05	-0.256	0.671	0.278	9	78,899,644	*VPS13A*	NM_001018037	upstream	82,537
rs10170474	3.27E-05	0.256	0.671	0.480	2	141,378,685	*LRP1B*	NM_018557.1	intron	-1,481
rs9305015	3.30E-05	-0.255	0.671	0.275	19	24,132,342	*ZNF539*	NM_203282.1	flanking_3UTR	-27,849
rs2542571	3.36E-05	0.255	0.671	0.453	2	53,895,487	*C2orf30*	NM_015701.2	intron	-14
rs258681	3.40E-05	0.261	0.671	0.324	7	81,572,147	*CACNA2D1*	NM_000722	intron	0
rs13090570	3.47E-05	-0.256	0.671	0.388	3	62,661,443	*CADPS*	NM_003716.2	intron	-38,334
rs6022377	3.48E-05	-0.255	0.671	0.155	20	51,343,089	*ZNF218*	NM_173485.2	intron	-36,572
rs2097915	3.80E-05	-0.258	0.677	0.245	7	81,593,392	*CACNA2D1*	NM_000722	intron	0
rs1931814	3.83E-05	0.253	0.677	0.466	6	62,647,126	*KHDRBS2*	NM_152688.1	intron	-15,373
rs2036010	3.83E-05	0.254	0.677	0.261	5	26,357,418	*CDH9*	NM_016279	downstream	559,048
rs11577282	3.91E-05	-0.253	0.677	0.397	1	224,354,245	*H3F3A*	NM_002107.3	flanking_3UTR	-27,919
rs6694114	3.97E-05	-0.253	0.677	0.493	1	56,682,138	*PPAP2B*	NM_003713.3	flanking_3UTR	-50,883
rs6801669	4.02E-05	-0.253	0.677	0.248	3	14,448,282	*SLC6A6*	NM_003043.2	intron	-11,854
rs10794843	4.06E-05	-0.254	0.677	0.473	10	2,079,841	*ADARB2*	NM_018702.1	flanking_5UTR	-310,171
rs2059287	4.06E-05	-0.253	0.677	0.344	16	85,385,659	*FOXL1*	NM_005250.1	flanking_3UTR	-214,791
rs10496859	4.26E-05	0.253	0.677	0.487	2	141,385,148	*LRP1B*	NM_018557.1	intron	-3,032
rs2436772	4.26E-05	-0.252	0.677	0.147	2	47,137,061	*TTC7A*	NM_020458.1	intron	-4,351
rs17126767	4.31E-05	-0.252	0.677	0.060	11	122,084,614	*STS-1*	NM_032873	intron	0
rs11190120	4.32E-05	0.254	0.677	0.129	10	101,250,433	*GOT1*	NM_002079	upstream	70,093
rs13337840	4.38E-05	-0.252	0.677	0.346	16	85,384,840	*FOXL1*	NM_005250	downstream	212,044
rs7549105	4.42E-05	-0.251	0.677	0.257	1	245,271,679	*ZNF670*	NM_033213.2	intron	-2,217
rs138777	4.45E-05	0.252	0.677	0.478	22	34,041,098	*TOM1*	NM_005488.1	intron	-2,772
rs2837111	4.51E-05	-0.252	0.677	0.162	21	39,963,691	*B3GALT5*	NM_033170.1	flanking_3UTR	-7,006

rs2142374	4.61E-05	-0.253	0.677	0.320	21	29,198,925	*C21orf127*	NM_182749.1	flanking_5UTR	-19,361
rs16967900	4.66E-05	0.251	0.677	0.103	13	87,675,804	*SLITRK5*	NM_015567.1	flanking_3UTR	-545,932
rs9998109	4.66E-05	-0.251	0.677	0.230	4	118,464,432	*TRAM1L1*	NM_152402.1	flanking_5UTR	-238,252
rs6902304	4.67E-05	-0.251	0.677	0.060	6	162,291,695	*PARK2*	NM_013988.1	intron	-22,629
rs10903646	4.69E-05	-0.251	0.677	0.473	10	2,077,959	*ADARB2*	NM_018702	upstream	308,289
rs2402635	4.73E-05	-0.250	0.677	0.393	7	122,607,115	*SLC13A1*	NM_022444.3	intron	-1,148
rs4390811	4.73E-05	-0.254	0.677	0.479	2	44,029,729	*LRPPRC*	NM_133259	intron	0
rs492190	4.79E-05	-0.250	0.677	0.063	2	141,071,577	*LRP1B*	NM_018557	intron	0
rs13162806	4.80E-05	-0.251	0.677	0.187	5	31,094,383	*CDH6*	NM_004932.2	flanking_5UTR	-135,170
rs1471804	4.88E-05	0.254	0.677	0.174	3	84,698,954	*IGSF4D*	NM_153184.2	flanking_5UTR	-1,159,368
rs571644	4.89E-05	0.250	0.677	0.303	6	163,397,094	*PACRG*	NM_152410.1	intron	-6,078
rs10032537	5.12E-05	-0.249	0.683	0.094	4	20,307,202	---	ENST0000038216	5UTR	0
rs7334500	5.13E-05	0.250	0.683	0.096	13	87,605,659	*SLITRK5*	NM_015567.1	flanking_3UTR	-475,787
rs12908437	5.18E-05	0.250	0.683	0.489	15	97,104,898	*IGF1R*	NM_000875.2	intron	-36,039
rs326782	5.18E-05	-0.250	0.683	0.180	11	29,589,362	*KCNA4*	NM_002233.2	flanking_3UTR	-398,979
rs1943141	5.45E-05	0.249	0.683	0.055	11	58,556,950	---	ENST0000037826	downstream	53,531
rs7783629	5.47E-05	-0.249	0.683	0.409	7	88,728,527	*FLJ32110*	NM_181646.2	intron	-42,982
rs7077248	5.49E-05	-0.248	0.683	0.062	10	18,759,447	*CACNB2*	NM_201590	intron	0
rs1897356	5.58E-05	0.249	0.683	0.146	15	24,416,628	*GABRB3*	NM_021912.2	intron	-926
rs1791510	5.64E-05	-0.248	0.683	0.109	18	20,389,114	*HRH4*	NM_021624.2	flanking_3UTR	-75,195
rs2254160	5.72E-05	-0.248	0.683	0.308	21	29,174,433	*N6AMT1*	NM_013240	intron	0
rs6009824	5.73E-05	0.248	0.683	0.221	22	48,472,377	*LOC348645*	NM_198851.1	flanking_5UTR	-67,805
rs2012378	5.84E-05	0.248	0.683	0.422	22	48,458,759	*LOC348645*	NM_198851.1	flanking_5UTR	-54,187
rs2086209	5.85E-05	-0.248	0.683	0.444	1	235,680,194	*RYR2*	NM_001035	intron	0
rs467401	5.85E-05	0.247	0.683	0.210	5	123,769,727	*ZNF608*	NM_020747	downstream	230,782
rs7120154	5.88E-05	-0.248	0.683	0.105	11	7,551,379	*PPFIBP2*	NM_003621	intron	0
rs11133515	5.89E-05	-0.247	0.683	0.196	4	58,226,143	---	ENST0000038790	upstream	172,847
rs16996589	5.95E-05	-0.248	0.683	0.091	21	38,913,016	*ERG*	NM_004449.3	intron	-34,277
rs8024723	5.96E-05	0.247	0.683	0.134	15	24,384,455	*GABRB3*	NM_000814	intron	0
rs1575836	6.02E-05	-0.247	0.683	0.058	10	18,754,711	*CACNB2*	NM_201590	intron	0
rs8111677	6.06E-05	-0.250	0.683	0.454	19	59,297,090	*NDUFA3*	NM_004542.1	flanking_5UTR	-882
rs472074	6.09E-05	-0.253	0.683	0.205	11	121,213,076	*SORL1*	NM_003105.3	flanking_3UTR	-207,455
rs8030011	6.11E-05	0.250	0.683	0.138	15	24,369,455	*GABRB3*	NM_021912.2	intron	-5,482
rs9529475	6.27E-05	-0.247	0.683	0.368	13	68,463,835	*KLHL1*	NM_020866.1	flanking_3UTR	-708,891
rs9529473	6.27E-05	-0.247	0.683	0.368	13	68,462,540	*KLHL1*	NM_020866	downstream	710,186
rs13411595	6.40E-05	0.248	0.683	0.361	2	1,266,879	*SNTG2*	NM_018968.2	flanking_3UTR	-12,955
rs7166999	6.43E-05	0.246	0.683	0.473	15	97,110,328	*IGF1R*	NM_000875.2	intron	-41,469
rs10744409	6.46E-05	0.247	0.683	0.475	12	128,349,391	*KIAA1944*	NM_133448.1	intron	-38,741
rs8189856	6.69E-05	-0.246	0.683	0.336	19	24,279,190	*ZNF539*	NM_203282.1	flanking_3UTR	-174,697
rs4925622	6.69E-05	-0.248	0.683	0.252	1	245,307,486	*ZNF670*	NM_033213	intron	0
rs2205449	6.69E-05	-0.246	0.683	0.366	21	29,173,959	*N6AMT1*	NM_013240	intron	0
rs17023252	6.70E-05	0.246	0.683	0.120	2	83,211,007	*LOC388965*	NM_001013648.3	flanking_5UTR	-1,160,311
rs17304569	6.74E-05	-0.245	0.683	0.163	19	24,032,745	*ZNF254*	NM_203282	upstream	29,071
rs256515	6.75E-05	0.246	0.683	0.207	5	89,410,921	*MEF2C*	NM_002397	upstream	1,196,103
rs12294127	6.83E-05	-0.245	0.683	0.058	11	11,354,048	*GALNTL4*	NM_198516.1	intron	-1,257
rs8134005	6.83E-05	-0.246	0.683	0.363	21	29,166,041	*C21orf127*	NM_182749.1	flanking_3UTR	-4,305

rs6688227	6.85E-05	0.245	0.683	0.341	1	245,077,357	*AHCTF1*	NM_015446.3	intron	-2,183
rs10274168	6.87E-05	0.245	0.683	0.051	7	21,647,374	*DNAH11*	NM_003777	intron	0
rs4861773	6.88E-05	0.245	0.683	0.170	4	180,530,610	*AGA*	NM_000027.2	flanking_5UTR	-1,930,025
rs5021094	7.27E-05	0.244	0.704	0.346	1	245,072,783	*AHCTF1*	NM_015446.3	intron	-82
rs6009822	7.42E-05	0.246	0.704	0.194	22	48,471,735	*C22orf34*	NM_001039473	upstream	67,139
rs180434	7.43E-05	0.245	0.704	0.149	12	45,465,841	*SLC38A4*	NM_018018.2	intron	-614
rs11670289	7.44E-05	-0.244	0.704	0.187	19	23,858,796	---	ENST0000032301	downstream	51,684
rs1372330	7.46E-05	0.244	0.704	0.147	9	118,559,409	*ASTN2*	NM_198187.2	intron	-23,786
rs6064647	7.52E-05	0.245	0.704	0.460	20	56,379,407	*RAB22A*	NM_020673.2	flanking_3UTR	-3,438
rs6685066	7.52E-05	0.244	0.704	0.342	1	245,067,789	*AHCTF1*	NM_015446.3	flanking_3UTR	-1,234
rs10502074	7.56E-05	0.245	0.704	0.111	11	106,064,623	*GUCY1A2*	NM_000855	intron	0
rs11923950	7.61E-05	-0.244	0.704	0.250	3	144,643,228	*SLC9A9*	NM_173653.1	intron	-25,341
rs10741061	7.62E-05	-0.244	0.704	0.058	10	18,761,210	*CACNB2*	NM_201570.1	intron	-30,232
rs17389027	7.72E-05	-0.249	0.709	0.100	13	108,149,963	*MYO16*	NM_015011	intron	0
rs17801638	7.95E-05	-0.243	0.724	0.335	3	54,103,615	*CACNA2D3*	NM_018398	upstream	28,118
rs7173713	8.00E-05	0.243	0.724	0.192	15	24,437,247	*GABRB3*	NM_021912.2	intron	-19,473
rs11579295	8.18E-05	-0.243	0.732	0.104	1	82,808,929	*LPHN2*	NM_012302.2	flanking_3UTR	-578,234
rs7176023	8.22E-05	-0.244	0.732	0.157	15	62,888,060	*RBPMS2*	NM_194272	upstream	33,221
rs6091627	8.31E-05	-0.243	0.732	0.158	20	51,103,779	*ZNF218*	NM_173485.2	intron	-80,500
rs7550882	8.33E-05	-0.242	0.732	0.486	1	56,675,980	*PPAP2B*	NM_003713.3	flanking_3UTR	-57,041
rs2837536	8.36E-05	0.242	0.732	0.409	21	40,546,492	*DSCAM*	NM_001389.3	intron	-23,402
rs10064948	8.50E-05	0.242	0.734	0.069	5	42,351,457	*GHR*	NM_000163.2	flanking_5UTR	-108,326
rs10472369	8.50E-05	0.242	0.734	0.069	5	42,356,768	*GHR*	NM_000163	upstream	103,015
rs7790698	8.59E-05	-0.243	0.735	0.281	7	132,236,701	*CHCHD3*	NM_017812.1	intron	-15,656
rs2039810	8.62E-05	-0.242	0.735	0.190	9	110,551,491	*ACTL7B*	NM_006686	downstream	105,201
rs2402637	8.77E-05	-0.242	0.735	0.319	7	122,659,570	*SLC13A1*	NM_022444	upstream	32,309
rs1476596	8.77E-05	0.243	0.735	0.352	17	53,840,357	*RNF43*	NM_017763	intron	0
rs13362216	8.95E-05	0.242	0.735	0.235	5	89,301,714	*CETN3*	NM_004365.2	flanking_3UTR	-423,570
rs1926026	8.96E-05	-0.241	0.735	0.056	10	18,765,665	*CACNB2*	NM_201570.1	intron	-34,687
rs6023594	9.01E-05	0.241	0.735	0.065	20	52,832,230	*DOK5*	NM_177959.2	flanking_3UTR	-131,113
rs6865426	9.04E-05	-0.242	0.735	0.113	5	79,434,465	*SERINC5*	NM_178276.2	flanking_3UTR	-8,341
rs16964247	9.14E-05	-0.241	0.735	0.433	15	34,964,979	*MEIS2*	NM_170676.2	flanking_3UTR	-5,545
rs1507680	9.34E-05	0.241	0.735	0.404	4	171,645,614	*AADAT*	NM_182662.1	flanking_5UTR	-397,667
rs8089498	9.37E-05	0.241	0.735	0.171	18	41,002,363	*SETBP1*	NM_015559.1	flanking_3UTR	-103,592
rs9876768	9.41E-05	0.241	0.735	0.118	3	17,024,969	*PLCL2*	NM_015184.2	intron	-1,201
rs11707511	9.47E-05	-0.241	0.735	0.127	3	10,438,507	*ATP2B2*	NM_001001331	intron	0
rs2016582	9.49E-05	0.243	0.735	0.443	2	141,368,709	*LRP1B*	NM_018557.1	intron	-8,250
rs11200975	9.56E-05	-0.240	0.735	0.098	10	86,048,189	*RGR*	NM_001012722.1	flanking_3UTR	-39,265
rs10135113	9.66E-05	0.241	0.735	0.444	14	46,569,136	*MAMDC1*	NM_182830.2	intron	-4,828
rs10520078	9.73E-05	-0.241	0.735	0.367	4	151,860,636	*LRBA*	NM_006726.1	intron	-15,224
rs4560562	9.73E-05	0.240	0.735	0.313	5	171,666,576	*FLJ40453*	NM_001007542.1	flanking_3UTR	-8,591
rs491862	9.74E-05	0.240	0.735	0.304	6	163,403,721	*PACRG*	NM_152410	intron	0
rs6748157	9.79E-05	0.242	0.735	0.326	2	28,586,865	*PLB1*	NM_153021	intron	0
rs932398	9.81E-05	-0.240	0.735	0.067	1	165,773,287	*CREG1*	NM_003851.2	flanking_3UTR	-3,587
rs4636276	9.87E-05	-0.242	0.735	0.190	9	110,551,437	*ACTL7B*	NM_006686	downstream	105,255
rs7803611	9.92E-05	0.240	0.735	0.133	7	6,022,232	*JTV1*	NM_006303	intron	0

rs2472209	9.94E-05	-0.240	0.735	0.322	21	29,203,291	*N6AMT1*	NM_182749	upstream	23,745
rs17216148	9.94E-05	0.240	0.735	0.049	X	15,010,665	*ASB9*	NM_001031739	downstream	161,365
rs4938769	9.95E-05	-0.243	0.735	0.149	11	119,453,776	---	NM_001001681	downstream	418,572
rs2008968	1.01E-04	-0.240	0.739	0.103	1	61,379,622	*NFIA*	NM_005595.1	intron	-52,682
rs17823562	1.01E-04	-0.240	0.739	0.339	18	41,242,086	*SLC14A2*	NM_007163	upstream	216,542
rs17750770	1.03E-04	-0.240	0.744	0.352	18	41,242,328	*SLC14A2*	NM_007163	upstream	216,300
rs7561677	1.03E-04	-0.240	0.744	0.051	2	44,982,944	*C2orf34*	NM_024766	downstream	129,709
rs2007855	1.04E-04	-0.239	0.744	0.319	6	15,896,359	*DTNBP1*	NM_183040.1	flanking_5UTR	-125,109
rs6887837	1.04E-04	0.240	0.744	0.085	5	167,601,811	*WWC1*	NM_015238.1	flanking_5UTR	-49,859
rs2996550	1.06E-04	0.239	0.751	0.344	1	245,067,371	*AHCTF1*	NM_015446.3	flanking_3UTR	-1,652
rs11901880	1.07E-04	0.239	0.751	0.440	2	141,322,453	*LRP1B*	NM_018557.1	intron	-1,688
rs11950209	1.07E-04	-0.240	0.751	0.421	5	99,162,556	---	ENST0000036348	upstream	862,205
rs7582078	1.08E-04	0.240	0.753	0.454	2	208,355,096	*FZD5*	NM_003468.2	flanking_5UTR	-12,733
rs2216768	1.08E-04	0.239	0.753	0.438	16	79,917,326	*GAN*	NM_022041	intron	0
rs7770588	1.10E-04	-0.239	0.754	0.075	6	168,048,125	*MLLT4*	NM_005936.1	intron	-2,210
rs316746	1.11E-04	0.238	0.754	0.060	5	41,718,898	*OXCT1*	NM_000436	downstream	47,026
rs138764	1.12E-04	0.238	0.754	0.466	22	34,033,128	*TOM1*	NM_005488.1	intron	-7,155
rs12427905	1.12E-04	0.239	0.754	0.229	13	60,975,427	*PCDH20*	NM_022843	upstream	88,030
rs2412493	1.14E-04	0.238	0.754	0.093	4	54,333,456	*LOC402176*	NM_001011538.1	flanking_3UTR	-212,954
rs4811423	1.14E-04	-0.240	0.754	0.097	20	51,348,131	*ZNF218*	NM_173485.2	intron	-41,614
rs6688116	1.14E-04	0.238	0.754	0.393	1	201,758,744	*OPTC*	NM_014359.3	flanking_3UTR	-14,044
rs316768	1.15E-04	0.238	0.754	0.058	5	41,747,643	*OXCT1*	NM_000436.2	flanking_3UTR	-18,281
rs13109743	1.15E-04	0.239	0.754	0.412	4	171,548,076	*AADAT*	NM_016228	upstream	300,129
rs13251311	1.17E-04	-0.239	0.754	0.451	8	69,219,699	*DEPDC2*	NM_024870.2	intron	-1,299
rs2590767	1.17E-04	0.238	0.754	0.080	4	54,313,331	*LNX1*	NM_032622.1	flanking_5UTR	-194,207
rs9458424	1.18E-04	-0.238	0.754	0.087	6	162,288,875	*PARK2*	NM_013988.1	intron	-25,449
rs12529984	1.18E-04	-0.237	0.754	0.111	6	71,161,188	*FAM135A*	NM_020819	upstream	18,802
rs10837192	1.18E-04	0.237	0.754	0.123	11	39,599,726	---	ENST0000036472	downstream	360,387
rs10405242	1.21E-04	-0.241	0.754	0.442	19	24,166,259	*ZNF539*	NM_203282.1	flanking_3UTR	-61,766
rs17059944	1.23E-04	0.238	0.754	0.235	13	60,972,307	*PCDH20*	NM_022843.2	flanking_5UTR	-85,025
rs11588591	1.23E-04	-0.237	0.754	0.447	1	235,687,577	*RYR2*	NM_001035	intron	0
rs2189702	1.23E-04	0.240	0.754	0.094	4	110,305,269	*COL25A1*	NM_198721	intron	0
rs3797023	1.25E-04	0.237	0.754	0.353	4	164,678,757	*1-Mar*	NM_017923.2	intron	-7,441
rs6877816	1.28E-04	-0.237	0.754	0.393	5	99,180,571	*UNQ1912*	NM_198507.1	flanking_5UTR	-718,452
rs12414938	1.28E-04	-0.236	0.754	0.100	10	86,046,121	*KIAA1128*	NM_018999	upstream	32,269
rs12500811	1.29E-04	0.236	0.754	0.132	4	138,071,139	*PCDH18*	NM_019035.2	flanking_3UTR	-589,146
rs12892702	1.29E-04	-0.236	0.754	0.078	14	94,946,219	*C14orf139*	NM_024633.2	flanking_5UTR	-39
rs17304953	1.29E-04	0.236	0.754	0.085	4	110,374,487	*COL25A1*	NM_198721	intron	0
rs10495448	1.30E-04	-0.236	0.754	0.236	1	238,122,096	*CHRM3*	NM_000740.2	intron	-15,260
rs2329317	1.30E-04	0.236	0.754	0.444	13	60,968,820	*PCDH20*	NM_022843.2	flanking_5UTR	-81,538
rs7924406	1.31E-04	-0.236	0.754	0.076	11	91,455,588	*CHORDC1*	NM_012124	upstream	1,859,761
rs2204294	1.32E-04	-0.236	0.754	0.351	7	122,622,978	*SLC13A1*	NM_022444.3	intron	-4,160
rs17673882	1.33E-04	-0.236	0.754	0.210	7	71,292,111	*CALN1*	NM_031468	intron	0
rs2837534	1.34E-04	0.236	0.754	0.458	21	40,544,164	*DSCAM*	NM_001389.3	intron	-25,730
rs13154264	1.35E-04	-0.235	0.754	0.493	5	75,821,020	*IQGAP2*	NM_006633	intron	0
rs7230301	1.36E-04	-0.237	0.754	0.374	18	41,244,751	*SLC14A2*	NM_007163.2	flanking_5UTR	-204,013

rs6755617	1.37E-04	-0.235	0.754	0.111	2	238,164,466	*RAB17*	NM_022449.1	UTR	[617/9]
rs2135899	1.37E-04	0.235	0.754	0.332	4	171,649,780	*AADAT*	NM_016228	upstream	401,833
rs4457010	1.37E-04	0.235	0.754	0.089	4	54,326,529	*LOC402176*	NM_001011538	downstream	220,979
rs11196739	1.38E-04	-0.236	0.754	0.252	10	116,178,475	*ABLIM1*	NM_001003408.1	flanking_3UTR	-2,384
rs11551242	1.39E-04	-0.235	0.754	0.272	10	116,182,110	*ABLIM1*	NM_001003408.1	3UTR	[1251/3898]
rs17620029	1.39E-04	-0.235	0.754	0.135	19	24,146,152	*ZNF539*	NM_203282.1	flanking_3UTR	-41,659
rs258658	1.40E-04	0.237	0.754	0.392	7	81,563,065	*CACNA2D1*	NM_000722.2	intron	-10,913
rs7113258	1.41E-04	-0.235	0.754	0.266	11	5,688,031	*TRIM5*	NM_033093	intron	0
rs1981838	1.42E-04	-0.236	0.754	0.212	5	57,510,605	*PLK2*	NM_006622.1	flanking_3UTR	-274,965
rs285198	1.42E-04	-0.235	0.754	0.260	20	41,798,148	*FAM112A*	NM_001008901.1	flanking_5UTR	-9,092
rs39823	1.42E-04	0.235	0.754	0.225	5	169,187,984	*DOCK2*	NM_004946.1	intron	-12,355
rs281526	1.42E-04	-0.236	0.754	0.277	2	225,561,862	*DOCK10*	NM_014689	intron	0
rs298525	1.44E-04	0.234	0.754	0.384	5	17,284,485	*BASP1*	NM_006317.3	intron	-13,566
rs4319365	1.44E-04	0.234	0.754	0.426	1	56,680,308	*PPAP2B*	NM_003713.3	flanking_3UTR	-52,713
rs4595481	1.44E-04	0.234	0.754	0.165	10	101,254,152	*NKX2-3*	NM_145285.1	flanking_5UTR	-28,548
rs2832137	1.44E-04	-0.234	0.754	0.312	21	29,201,887	*N6AMT1*	NM_013240	upstream	22,341
rs12467730	1.44E-04	0.235	0.754	0.445	2	141,320,821	*LRP1B*	NM_018557	intron	0
rs7254714	1.45E-04	-0.234	0.754	0.259	19	23,960,398	*ZNF254*	NM_004876.1	flanking_5UTR	-101,418
rs7716895	1.45E-04	0.234	0.754	0.339	5	11,788,134	*CTNND2*	NM_001332	intron	0
rs7111680	1.45E-04	0.235	0.754	0.237	11	89,084,402	*PSMAL*	NM_153696	downstream	12,871
rs254259	1.46E-04	0.234	0.754	0.455	19	59,298,217	*NDUFA3*	NM_004542.1	intron	-17
rs7680772	1.46E-04	0.234	0.754	0.214	4	14,520,157	*CPEB2*	NM_182485	upstream	94,463
rs2249287	1.46E-04	-0.234	0.754	0.179	1	236,004,497	*RYR2*	NM_001035	intron	0
rs2914	1.47E-04	-0.234	0.754	0.335	6	108,098,767	*FLJ10159*	NM_018013.2	flanking_3UTR	-10,700
rs746959	1.47E-04	-0.234	0.754	0.078	8	29,287,297	*DUSP4*	NM_001394.5	flanking_5UTR	-23,193
rs7250712	1.47E-04	-0.234	0.754	0.255	19	23,959,928	---	ENST0000038707	downstream	14,303
rs11203190	1.48E-04	-0.234	0.754	0.226	21	42,158,582	*PRDM15*	NM_022115.2	intron	-1,896
rs9915774	1.49E-04	-0.234	0.754	0.263	17	28,647,439	*ACCN1*	NM_183377.1	flanking_5UTR	-3,320
rs1956595	1.49E-04	-0.234	0.754	0.168	14	28,443,059	---	ENST0000038674	downstream	78,231
rs1344220	1.50E-04	-0.234	0.754	0.158	3	39,241,125	*CMYA1*	NM_194293.2	flanking_5UTR	-32,044
rs170290	1.50E-04	0.234	0.754	0.310	16	9,655,702	*GRIN2A*	NM_000833.2	flanking_3UTR	-107,221
rs7253132	1.50E-04	-0.235	0.754	0.440	19	24,170,037	*ZNF539*	NM_203282.1	flanking_3UTR	-65,544
rs8108300	1.51E-04	0.235	0.754	0.318	19	16,470,453	*FLJ21742*	NM_032207.1	intron	-2,150
rs7449116	1.51E-04	-0.234	0.754	0.395	5	99,164,462	---	ENST0000036250	upstream	220,945
rs1993109	1.52E-04	-0.235	0.754	0.367	4	151,876,039	*LRBA*	NM_006726.1	intron	-71
rs2733156	1.52E-04	0.237	0.754	0.076	18	26,648,373	---	ENST0000038480	downstream	1,617,883
rs5743401	1.52E-04	-0.234	0.754	0.259	8	6,724,992	*DEFB1*	NM_005218	upstream	2,053
rs258683	1.52E-04	0.234	0.754	0.451	7	81,572,869	*CACNA2D1*	NM_000722	intron	0
rs1022004	1.54E-04	0.233	0.754	0.375	4	171,650,402	*AADAT*	NM_182662.1	flanking_5UTR	-402,455
rs134430	1.54E-04	-0.234	0.754	0.158	22	33,918,600	*ISX*	NM_001008494	downstream	102,586
rs134426	1.54E-04	-0.234	0.754	0.158	22	33,917,480	*ISX*	NM_001008494	downstream	101,466
rs134410	1.54E-04	-0.234	0.754	0.158	22	33,910,597	*ISX*	NM_001008494	downstream	94,583
rs6569759	1.55E-04	0.234	0.754	0.400	6	132,174,809	*ENPP1*	NM_006208.1	intron	-3,701
rs2741136	1.57E-04	-0.233	0.754	0.254	8	6,724,606	*DEFB1*	NM_005218	upstream	1,667
rs759692	1.58E-04	-0.233	0.754	0.112	3	10,418,000	*ATP2B2*	NM_001001331	intron	0
rs2776252	1.59E-04	-0.235	0.754	0.225	21	29,453,576	*C21orf7*	NM_020152.2	intron	-557

rs4966024	1.59E-04	0.233	0.754	0.433	15	97,113,093	*IGF1R*	NM_000875	intron	0
rs10014019	1.60E-04	0.236	0.754	0.056	4	28,721,162	*PCDH7*	NM_002589.2	flanking_5UTR	-1,609,973
rs17237821	1.60E-04	-0.233	0.754	0.236	8	13,579,983	*C8orf48*	NM_001007090.1	flanking_3UTR	-109,816
rs2694103	1.62E-04	-0.233	0.754	0.104	2	3,222,245	*TSSC1*	NM_003310.1	intron	-17,832
rs17177982	1.62E-04	-0.234	0.754	0.337	14	36,505,046	*SLC25A21*	NM_030631	intron	0
rs7482968	1.63E-04	-0.233	0.754	0.131	11	20,387,592	*PRMT3*	NM_005788	intron	0
rs4423192	1.63E-04	-0.233	0.754	0.393	11	120,633,753	*TECTA*	NM_005422	downstream	67,028
rs6576576	1.64E-04	0.233	0.754	0.208	15	24,243,548	*GABRB3*	NM_021912.2	flanking_3UTR	-98,998
rs2733119	1.65E-04	0.232	0.754	0.087	18	26,645,010	---	ENST00000038480	downstream	1,614,520
rs3934576	1.66E-04	-0.232	0.754	0.212	1	171,665,059	*PRDX6*	NM_004905.2	flanking_5UTR	-48,050
rs7498372	1.66E-04	0.233	0.754	0.431	16	29,818,226	*SEZ6L2*	NM_201575.1	flanking_5UTR	-152
rs4294450	1.66E-04	-0.232	0.754	0.212	1	171,656,263	*LOC646870*	NM_001039790	downstream	40,266
rs3934575	1.66E-04	-0.232	0.754	0.212	1	171,665,156	*TNFSF4*	NM_003326	upstream	222,062
rs2045955	1.67E-04	-0.232	0.754	0.442	1	235,686,672	*RYR2*	NM_001035.1	intron	-14
rs739568	1.68E-04	0.232	0.754	0.259	11	43,973,557	*LOC390110*	NM_001031854.1	flanking_5UTR	-52,550
rs16937777	1.68E-04	0.232	0.754	0.259	11	43,970,888	*LOC390110*	NM_001031854	upstream	55,219
rs252272	1.68E-04	-0.233	0.754	0.095	16	29,008,903	---	ENST00000032730	downstream	5,368
rs326790	1.69E-04	-0.232	0.754	0.212	11	29,607,202	*METT5D1*	NM_152636	downstream	1,295,683
rs7960235	1.70E-04	-0.232	0.754	0.413	12	71,725,915	*TRHDE*	NM_013381	downstream	380,227
rs6809146	1.71E-04	0.232	0.754	0.159	3	20,337,443	*SGOL1*	NM_001012409.1	flanking_5UTR	-134,756
rs3754511	1.72E-04	0.232	0.754	0.187	1	17,182,606	*MFAP2*	NM_017459.1	flanking_5UTR	-1,938
rs6503238	1.72E-04	0.232	0.754	0.455	17	9,522,939	*WDRPUH*	NM_145054.2	flanking_3UTR	-35,512
rs10837198	1.73E-04	0.233	0.754	0.126	11	39,609,211	*LRRC4C*	NM_020929.1	flanking_3UTR	-483,118
rs2033871	1.73E-04	0.232	0.754	0.058	7	12,371,623	*TMEM106B*	NM_018374.2	flanking_3UTR	-128,212
rs134300	1.74E-04	-0.232	0.754	0.174	22	33,888,414	*ISX*	NM_001008494	downstream	72,400
rs6518933	1.74E-04	-0.232	0.754	0.174	22	33,890,386	*ISX*	NM_001008494	downstream	74,372
rs2413306	1.74E-04	-0.232	0.754	0.174	22	33,889,695	*ISX*	NM_001008494	downstream	73,681
rs7613994	1.75E-04	-0.232	0.754	0.054	3	120,382,545	*UPK1B*	NM_006952.3	intron	-5,706
rs4245983	1.76E-04	0.231	0.754	0.062	5	42,267,653	*GHR*	NM_000163.2	flanking_5UTR	-192,130
rs6738955	1.76E-04	0.232	0.754	0.409	2	1,267,304	*SNTG2*	NM_018968.2	flanking_3UTR	-13,380
rs2673743	1.77E-04	-0.231	0.754	0.366	12	107,329,282	*CMKLR1*	NM_004072.1	flanking_5UTR	-72,070
rs762052	1.77E-04	0.231	0.754	0.080	14	22,378,826	*MMP14*	NM_004995.2	intron	-1,714
rs17769347	1.78E-04	-0.232	0.754	0.096	18	36,989,057	*PIK3C3*	NM_002647.2	flanking_5UTR	-800,140
rs2036475	1.78E-04	-0.231	0.754	0.400	6	66,211,368	*EGFL11*	NM_198283.1	intron	-39,387
rs1161252	1.78E-04	-0.233	0.754	0.427	5	99,155,026	---	ENST00000036250	upstream	230,381
rs12882488	1.79E-04	-0.231	0.754	0.072	14	94,950,974	*C14orf49*	NM_152592.2	flanking_3UTR	-2,720
rs39964	1.79E-04	-0.232	0.754	0.229	5	5,849,906	*FLJ33360*	NM_001001702	downstream	515,577
rs17763056	1.80E-04	-0.232	0.754	0.073	20	16,807,716	*OTOR*	NM_020157.2	flanking_3UTR	-126,907
rs17764251	1.80E-04	-0.232	0.754	0.498	17	4,160,754	*UBE2G1*	NM_182682.2	intron	-3,587
rs2447045	1.80E-04	-0.232	0.754	0.221	11	29,616,020	*KCNA4*	NM_002233.2	flanking_3UTR	-372,321
rs11196740	1.80E-04	-0.231	0.754	0.254	10	116,180,264	*ABLIM1*	NM_001003407	downstream	598
rs7609295	1.82E-04	-0.231	0.754	0.107	2	105,952,444	*NCK2*	NM_001004720.1	flanking_3UTR	-75,284
rs326686	1.82E-04	0.231	0.754	0.437	11	15,780,714	*SOX6*	NM_017508	downstream	170,111
rs7220074	1.83E-04	-0.231	0.754	0.054	17	38,021,312	*TUBG1*	NM_001070.3	flanking_3UTR	-534
rs17142139	1.85E-04	-0.231	0.754	0.142	10	6,966,368	*SFMBT2*	NM_001029880.1	flanking_3UTR	-277,887
rs33811	1.85E-04	-0.231	0.754	0.121	5	94,647,539	*MCTP1*	NM_001002796	upstream	1,504

rs12890665	1.85E-04	-0.231	0.754	0.309	14	24,001,074	*CBLN3*	NM_001039771	upstream	32,503
rs12691579	1.86E-04	0.233	0.754	0.272	2	141,356,948	*LRP1B*	NM_018557.1	intron	-908
rs12416853	1.87E-04	0.231	0.754	0.115	11	39,589,082	*LRRC4C*	NM_020929.1	flanking_3UTR	-503,247
rs2235150	1.89E-04	0.230	0.754	0.480	22	34,002,843	*HMG2L1*	NM_005487.3	intron	-7,112
rs13430688	1.90E-04	0.234	0.754	0.236	2	81,275,462	*CTNNA2*	NM_004389.2	flanking_3UTR	-546,046
rs4074599	1.90E-04	-0.230	0.754	0.176	4	118,466,311	*TRAM1L1*	NM_152402.1	flanking_5UTR	-240,131
rs7637849	1.90E-04	-0.230	0.754	0.082	3	38,634,279	*SCN5A*	NM_198056.1	intron	-3,059
rs4555168	1.90E-04	-0.233	0.754	0.234	16	75,735,238	*MON1B*	NM_014940	upstream	47,099
rs17350515	1.90E-04	-0.231	0.754	0.332	7	18,908,149	*HDAC9*	NM_058176	intron	0
rs319960	1.91E-04	0.232	0.754	0.203	1	48,832,202	*AGBL4*	NM_032785.1	intron	-2,958
rs961533	1.91E-04	-0.231	0.754	0.422	4	183,641,347	*MGC45800*	NM_178838.3	flanking_5UTR	-338,734
rs17108556	1.91E-04	-0.230	0.754	0.094	1	82,825,294	*TTLL7*	NM_024686	downstream	1,278,005
rs11812365	1.95E-04	0.230	0.754	0.173	10	50,139,287	*C10orf71*	NM_199459.2	flanking_5UTR	-37,906
rs6583476	1.96E-04	-0.232	0.754	0.202	7	48,214,960	*ABCA13*	NM_152701.2	intron	-6,448
rs6959243	1.96E-04	0.230	0.754	0.201	7	108,284,468	*LOC154907*	NM_001024607.1	flanking_3UTR	-26,806
rs2591117	1.96E-04	0.230	0.754	0.107	18	26,624,221	---	ENST0000038715	upstream	193,849
rs2591116	1.96E-04	0.230	0.754	0.107	18	26,624,582	---	ENST0000038715	upstream	193,488
rs1880180	1.97E-04	-0.230	0.754	0.399	7	122,626,098	*SLC13A1*	NM_022444.3	intron	-1,040
rs568041	1.97E-04	0.230	0.754	0.297	6	163,407,675	*PACRG*	NM_152410.1	intron	-4,332
rs2250263	1.98E-04	0.232	0.754	0.245	6	116,940,700	*C6orf188*	NM_153711.1	intron	-608
rs6517072	1.98E-04	0.230	0.754	0.174	21	32,348,757	*HUNK*	NM_014586.1	flanking_3UTR	-50,509
rs7595292	1.99E-04	-0.230	0.754	0.220	2	173,892,452	*CDCA7*	NM_031942.3	flanking_5UTR	-35,342
rs2839398	1.99E-04	-0.230	0.754	0.219	21	42,159,421	*PRDM15*	NM_022115	intron	0
rs2053382	2.00E-04	0.230	0.754	0.056	7	12,367,112	*TMEM106B*	NM_018374.2	flanking_3UTR	-123,701
rs621147	2.01E-04	-0.229	0.754	0.491	19	59,303,099	*TFPT*	NM_013342.1	intron	-46
rs6598542	2.01E-04	0.232	0.754	0.369	15	97,110,579	*IGF1R*	NM_000875	intron	0
rs2939568	2.01E-04	-0.231	0.754	0.070	X	39,982,175	*BCOR*	NM_020926	upstream	160,009
rs9453572	2.02E-04	0.231	0.754	0.374	6	62,691,375	*KHDRBS2*	NM_152688.1	intron	-22,140
rs7559911	2.02E-04	0.229	0.754	0.078	2	212,894,596	*ERBB4*	NM_005235	intron	0
rs3750263	2.03E-04	-0.229	0.754	0.158	8	29,036,982	*KIF13B*	NM_015254.2	intron	-54
rs659266	2.03E-04	0.229	0.754	0.337	2	220,221,067	*SLC4A3*	NM_201574	downstream	6,131
rs5755674	2.04E-04	0.229	0.754	0.478	22	33,983,473	*HMG2L1*	NM_005487.3	flanking_5UTR	-16
rs6013656	2.04E-04	-0.229	0.754	0.156	20	51,344,606	*ZNF218*	NM_173485.2	intron	-38,089
rs10225295	2.05E-04	-0.230	0.754	0.413	7	81,562,653	*CACNA2D1*	NM_000722.2	intron	-10,501
rs4806851	2.06E-04	0.229	0.754	0.203	19	2,402,266	*LMNB2*	NM_032737.2	intron	-5,402
rs12993440	2.06E-04	0.230	0.754	0.098	2	212,896,258	*ERBB4*	NM_005235	intron	0
rs4587368	2.07E-04	-0.229	0.754	0.062	8	81,569,888	*ZBTB10*	NM_023929.2	intron	-4,396
rs16907781	2.07E-04	-0.229	0.754	0.062	8	81,598,497	*ZBTB10*	NM_023929	downstream	3,175
rs10860219	2.08E-04	-0.229	0.754	0.301	12	96,483,923	*NCRMS*	XR_000219.2	flanking_3UTR	-985
rs2073390	2.08E-04	-0.229	0.754	0.138	22	22,463,570	*MIF*	NM_002415	intron	0
rs3801148	2.09E-04	-0.229	0.754	0.391	7	139,340,969	*TBXAS1*	NM_030984.1	intron	-12,391
rs17576350	2.10E-04	-0.229	0.754	0.141	4	164,665,964	*FLJ11184*	NM_018352	downstream	4,857
rs1968758	2.11E-04	0.230	0.754	0.137	2	81,274,410	*CTNNA2*	NM_004389.2	flanking_3UTR	-544,994
rs4245150	2.11E-04	0.229	0.754	0.179	11	112,869,857	*DRD2*	NM_000795	upstream	18,754
rs6698540	2.11E-04	0.231	0.754	0.170	1	189,888,900	*RGS18*	NM_130782	upstream	505,310
rs4455394	2.12E-04	-0.229	0.754	0.367	4	151,844,811	*LRBA*	NM_006726.1	intron	-20,492

rs258684	2.15E-04	0.230	0.754	0.449	7	81,572,951	*CACNA2D1*	NM_000722.2	intron	-11,345
rs6127270	2.15E-04	0.230	0.754	0.280	20	52,788,534	*DOK5*	NM_177959.2	flanking_3UTR	-87,417
rs8048966	2.15E-04	0.229	0.754	0.107	16	67,124,288	*ZFP90*	NM_133458.1	flanking_5UTR	-6,427
rs258688	2.16E-04	0.230	0.754	0.280	7	81,589,143	*CACNA2D1*	NM_000722.2	intron	-4,718
rs2722583	2.17E-04	-0.228	0.754	0.065	2	5,859,596	*SOX11*	NM_003108.3	flanking_3UTR	-100,628
rs4363444	2.17E-04	-0.229	0.754	0.471	1	56,671,586	*PPAP2B*	NM_003713.3	flanking_3UTR	-61,435
rs2369765	2.18E-04	-0.229	0.754	0.422	5	99,171,761	*UNQ1912*	NM_198507.1	flanking_5UTR	-727,262
rs4472075	2.18E-04	-0.228	0.754	0.317	3	54,098,051	*CACNA2D3*	NM_018398.2	flanking_5UTR	-33,682
rs6594760	2.18E-04	-0.229	0.754	0.422	5	99,168,102	*UNQ1912*	NM_198507.1	flanking_5UTR	-730,921
rs4243134	2.18E-04	-0.230	0.754	0.233	16	75,734,629	*CNTNAP4*	NM_033401	downstream	584,168
rs1240769	2.19E-04	-0.228	0.754	0.384	11	116,022,801	*MGC13125*	NM_032725.2	flanking_3UTR	-101,298
rs4917139	2.20E-04	-0.228	0.754	0.203	7	48,232,695	*ABCA13*	NM_152701.2	intron	-1,247
rs7959787	2.21E-04	0.228	0.754	0.053	12	38,837,340	*SLC2A13*	NM_052885.1	flanking_5UTR	-51,412
rs6879586	2.21E-04	-0.228	0.754	0.425	5	99,178,065	---	ENST0000036348	upstream	877,714
rs4817244	2.21E-04	-0.228	0.754	0.298	21	28,993,474	*N6AMT1*	NM_182749	downstream	172,926
rs10502752	2.22E-04	-0.228	0.754	0.096	18	36,987,960	*PIK3C3*	NM_002647.2	flanking_5UTR	-801,237
rs13403000	2.22E-04	-0.228	0.754	0.333	2	13,285,676	*TRIB2*	NM_021643.1	flanking_3UTR	-485,365
rs2299059	2.22E-04	-0.230	0.754	0.206	6	15,484,973	*JARID2*	NM_004973	intron	0
rs1474558	2.23E-04	0.228	0.754	0.482	22	34,020,183	*HMG2L1*	NM_005487.3	3UTR	[538/1617]
rs741996	2.23E-04	0.228	0.754	0.482	22	34,014,059	*HMG2L1*	NM_005487.3	intron	-172
rs7923938	2.23E-04	-0.228	0.754	0.100	10	18,784,725	*CACNB2*	NM_201590	intron	0
rs16995282	2.24E-04	0.229	0.754	0.109	20	8,743,984	*PLCB1*	NM_182734.1	intron	-13,164
rs7665654	2.24E-04	-0.228	0.754	0.367	4	151,912,599	*LRBA*	NM_006726.1	intron	-10,150
rs2180154	2.24E-04	-0.228	0.754	0.408	22	42,144,401	*MPPED1*	NM_001044370	upstream	6,535
rs9544609	2.25E-04	-0.228	0.754	0.342	13	77,290,657	*FLJ30046*	NM_144595.2	flanking_3UTR	-54,292
rs16974138	2.25E-04	-0.228	0.754	0.095	18	36,989,950	*PIK3C3*	NM_002647	upstream	799,247
rs2915	2.25E-04	-0.228	0.754	0.337	6	108,098,738	*SCML4*	NM_198081	downstream	48,113
rs6796373	2.26E-04	0.228	0.754	0.076	3	51,799,207	*IQCF1*	NM_152397	downstream	104,726
rs1533777	2.26E-04	-0.230	0.754	0.482	1	231,545,978	*KIAA1804*	NM_032435	intron	0
rs13018198	2.27E-04	-0.228	0.754	0.150	2	48,678,202	*STON1*	NM_006873.2	3UTR	[2256/1347]
rs2710833	2.27E-04	0.231	0.754	0.164	4	169,646,533	*PALLD*	NM_016081.2	flanking_5UTR	-8,259
rs16880521	2.27E-04	0.228	0.754	0.105	5	8,464,410	---	ENST0000038718	downstream	207,807
rs13361808	2.28E-04	0.228	0.754	0.104	5	8,463,155	*MTRR*	NM_024010.1	flanking_3UTR	-508,918
rs661039	2.28E-04	0.228	0.754	0.337	2	220,235,167	*SLC4A3*	NM_005070	downstream	20,239
rs138726	2.30E-04	0.229	0.754	0.482	22	34,024,295	*TOM1*	NM_005488.1	flanking_5UTR	-1,566
rs628917	2.30E-04	0.227	0.754	0.335	2	220,229,846	*SLC4A3*	NM_005070.1	flanking_3UTR	-14,918
rs2251087	2.31E-04	-0.227	0.754	0.230	21	29,449,870	*C21orf7*	NM_020152.2	intron	-4,263
rs180433	2.31E-04	0.227	0.754	0.167	12	45,465,824	*SLC38A4*	NM_018018	intron	0
rs569879	2.31E-04	0.227	0.754	0.292	6	163,407,862	*PACRG*	NM_152410	intron	0
rs994912	2.33E-04	0.228	0.754	0.135	4	138,054,417	*PCDH18*	NM_019035.2	flanking_3UTR	-605,868
rs10826765	2.34E-04	0.227	0.754	0.438	10	30,447,990	*PAPD1*	NM_018109.2	flanking_3UTR	-193,775
rs991072	2.35E-04	0.227	0.754	0.341	X	141,779,863	*SPANX-N4*	NM_001009613.1	flanking_5UTR	-161,604
rs10418693	2.36E-04	-0.231	0.754	0.425	19	59,314,300	*PRPF31*	NM_015629.2	intron	-475
rs10500223	2.37E-04	-0.228	0.754	0.128	19	24,137,864	*ZNF539*	NM_203282.1	flanking_3UTR	-33,371
rs7705032	2.37E-04	-0.227	0.754	0.397	5	99,175,332	---	ENST0000036348	upstream	874,981
rs2345417	2.38E-04	0.227	0.754	0.416	4	110,354,624	*COL25A1*	NM_032518.2	intron	-86,564

rs7657997	2.38E-04	-0.227	0.754	0.338	4	151,735,416	*LRBA*	NM_006726.1	intron	-2,269
rs11165262	2.39E-04	0.227	0.754	0.109	1	91,926,562	*TGFBR3*	NM_003243.2	intron	-4,560
rs2123812	2.39E-04	-0.227	0.754	0.424	5	99,174,420	---	ENST0000036250	upstream	210,987
rs982279	2.40E-04	-0.227	0.754	0.297	21	28,982,417	*N6AMT1*	NM_182749	downstream	183,983
rs2831965	2.40E-04	-0.227	0.754	0.297	21	28,997,768	*N6AMT1*	NM_013240	downstream	168,632
rs961532	2.40E-04	-0.227	0.754	0.473	4	183,641,288	---	ENST0000038904	intron	0
rs6964905	2.42E-04	-0.227	0.754	0.444	7	107,286,997	*SLC26A3*	NM_000111	upstream	56,109
rs16967807	2.42E-04	0.228	0.754	0.099	13	87,672,374	*SLITRK5*	NM_015567	downstream	542,502
rs8092776	2.43E-04	0.227	0.754	0.262	18	51,682,730	*TCF4*	NM_003199.1	flanking_5UTR	-276,290
rs11071406	2.44E-04	0.227	0.754	0.493	15	30,016,775	---	ENST0000032399	upstream	38,100
rs17733134	2.46E-04	0.226	0.754	0.252	20	18,314,623	*C20orf12*	NM_018152.2	intron	-889
rs4294501	2.48E-04	0.227	0.754	0.196	10	134,723,178	*GPR123*	NM_032422.1	flanking_5UTR	-11,245
rs10000193	2.49E-04	-0.227	0.754	0.364	4	151,836,543	*LRBA*	NM_006726.1	intron	-12,224
rs1273433	2.49E-04	-0.228	0.754	0.137	16	8,760,561	*ABAT*	NM_020686	intron	0
rs5970876	2.49E-04	-0.227	0.754	0.331	X	22,670,209	*DDX53*	NM_182699	upstream	257,790
rs9386156	2.50E-04	-0.226	0.754	0.348	6	146,897,549	*RAB32*	NM_006834.2	flanking_5UTR	-8,972
rs10944479	2.51E-04	0.226	0.754	0.071	6	90,937,114	*BACH2*	NM_021813.1	intron	-35,895
rs1201198	2.51E-04	-0.227	0.754	0.131	4	152,079,124	*LRBA*	NM_006726.1	intron	-9,457
rs11878982	2.52E-04	-0.226	0.754	0.114	19	4,992,049	*JMJD2B*	NM_015015.1	intron	-99
rs17670403	2.52E-04	-0.226	0.754	0.179	2	104,527,260	---	ENST0000036285	downstream	11,386
rs1078772	2.54E-04	-0.226	0.754	0.424	5	99,179,276	*UNQ1912*	NM_198507.1	flanking_5UTR	-719,747
rs11955182	2.54E-04	-0.226	0.754	0.424	5	99,178,433	---	ENST0000036348	upstream	878,082
rs7705200	2.54E-04	-0.226	0.754	0.424	5	99,175,430	---	ENST0000036250	upstream	209,977
rs11134612	2.55E-04	-0.226	0.754	0.142	5	169,483,814	*FOXI1*	NM_144769.1	flanking_3UTR	-14,509
rs17703235	2.55E-04	-0.226	0.754	0.053	6	124,065,374	*TRDN*	NM_006073.1	flanking_5UTR	-65,733
rs10044143	2.56E-04	-0.226	0.754	0.129	5	94,558,969	*MCTP1*	NM_024717.3	intron	-86,347
rs4793952	2.56E-04	0.226	0.754	0.373	17	53,963,464	*4-Sep*	NM_080416.1	intron	-857
rs947420	2.56E-04	0.226	0.754	0.371	1	69,787,574	*LRRC7*	NM_020794.1	flanking_5UTR	-210,872
rs9883082	2.56E-04	-0.226	0.754	0.317	3	7,858,963	*GRM7*	NM_000844.2	flanking_3UTR	-100,746
rs1161257	2.57E-04	-0.226	0.754	0.417	5	99,157,226	*UNQ1912*	NM_198507.1	flanking_5UTR	-741,797
rs792720	2.57E-04	-0.227	0.754	0.082	10	102,909,091	*TLX1*	NM_005521	downstream	21,556
rs1430275	2.58E-04	0.226	0.754	0.161	2	83,189,782	*LOC388965*	NM_001013648.3	flanking_5UTR	-1,181,536
rs17659581	2.58E-04	-0.226	0.754	0.255	4	170,796,015	*CLCN3*	NM_173872.2	intron	-2,201
rs1581676	2.59E-04	0.226	0.754	0.389	4	171,638,800	*AADAT*	NM_182662.1	flanking_5UTR	-390,853
rs2591118	2.59E-04	0.226	0.754	0.102	18	26,621,891	*DSC3*	NM_001941.2	flanking_3UTR	-203,138
rs5965871	2.59E-04	0.226	0.754	0.098	X	144,480,774	*SLITRK2*	NM_032539	upstream	226,399
rs9324892	2.59E-04	0.226	0.754	0.228	5	142,039,584	*FGF1*	NM_000800	intron	0
rs6861611	2.60E-04	-0.226	0.754	0.139	5	169,496,161	*FOXI1*	NM_144769.1	flanking_3UTR	-26,856
rs7210943	2.60E-04	0.226	0.754	0.464	17	5,923,017	*KIAA0523*	NM_015253.1	intron	-1,398
rs6960102	2.63E-04	-0.225	0.754	0.152	7	81,575,263	*CACNA2D1*	NM_000722	intron	0
rs2024370	2.64E-04	-0.226	0.754	0.102	3	139,007,444	*SOX14*	NM_004189.2	flanking_3UTR	-40,358
rs4820189	2.64E-04	0.226	0.754	0.482	22	33,988,225	*HMG2L1*	NM_001003681.1	intron	-91
rs921963	2.65E-04	0.225	0.754	0.243	2	81,279,994	*CTNNA2*	NM_004389.2	flanking_3UTR	-550,578
rs13258742	2.66E-04	-0.226	0.754	0.084	8	128,617,860	*POU5F1P1*	NR_002304.1	flanking_3UTR	-119,487
rs17133655	2.66E-04	0.225	0.754	0.111	5	111,064,894	*C5orf13*	NM_004772.1	flanking_3UTR	-27,998
rs9427713	2.66E-04	-0.225	0.754	0.149	1	199,307,987	*CACNA1S*	NM_000069.1	intron	-519

rs17083391	2.66E-04	0.225	0.754	0.105	9	82,260,377	*TLE4*	NM_007005	downstream	728,899
rs17390122	2.66E-04	-0.225	0.754	0.054	13	22,969,322	*TNFRSF19*	NM_148957	upstream	73,401
rs2526434	2.67E-04	0.225	0.754	0.107	4	110,317,016	*COL25A1*	NM_032518.2	intron	-124,172
rs4938021	2.67E-04	0.226	0.754	0.180	11	112,870,013	*DRD2*	NM_016574.2	flanking_5UTR	-18,922
rs6589377	2.67E-04	0.226	0.754	0.180	11	112,860,946	*DRD2*	NM_016574.2	flanking_5UTR	-9,855
rs997264	2.67E-04	-0.225	0.754	0.063	10	18,743,901	*CACNB2*	NM_201590	intron	0
rs2046736	2.68E-04	0.225	0.754	0.139	1	91,947,596	*TGFBR3*	NM_003243.2	intron	-668
rs4254753	2.68E-04	0.225	0.754	0.288	4	154,019,479	*ARFIP1*	NM_001025593.1	intron	-2,086
rs749527	2.68E-04	-0.225	0.754	0.424	5	99,152,307	*UNQ1912*	NM_198507.1	flanking_5UTR	-746,716
rs8113608	2.68E-04	-0.225	0.754	0.185	19	23,852,984	*LOC388524*	NM_001005472.1	flanking_3UTR	-50,207
rs10896396	2.69E-04	0.226	0.754	0.321	11	68,561,382	*TPCN2*	NM_139075.1	flanking_5UTR	-11,559
rs6878343	2.69E-04	-0.226	0.754	0.429	5	99,181,038	---	ENST0000036250	upstream	204,369
rs9305380	2.69E-04	-0.226	0.754	0.288	21	28,997,357	*N6AMT1*	NM_013240	downstream	169,043
rs13322861	2.69E-04	-0.225	0.754	0.319	3	54,097,713	*CACNA2D3*	NM_018398	upstream	34,020
rs10071435	2.70E-04	-0.231	0.754	0.309	5	117,418,263	*DTWD2*	NM_173666.1	flanking_3UTR	-784,872
rs10822139	2.70E-04	-0.225	0.754	0.246	10	52,462,200	*PRKG1*	NM_006258.1	flanking_5UTR	-42,099
rs2053381	2.71E-04	0.225	0.754	0.056	7	12,367,235	*TMEM106B*	NM_018374.2	flanking_3UTR	-123,824
rs483682	2.71E-04	0.225	0.754	0.349	3	16,340,227	*RAFTLIN*	NM_015150.1	intron	-259
rs2185368	2.71E-04	-0.225	0.754	0.076	1	243,982,205	*SMYD3*	NM_022743	intron	0
rs11592006	2.71E-04	-0.225	0.754	0.111	10	11,696,606	*ECHDC3*	NM_024693	upstream	127,765
rs9868927	2.72E-04	0.225	0.754	0.399	3	174,822,471	*NLGN1*	NM_014932.2	intron	-16,896
rs701840	2.72E-04	-0.225	0.754	0.084	10	102,902,639	*TLX1*	NM_005521	downstream	15,104
rs7807163	2.74E-04	-0.225	0.754	0.449	7	122,665,695	*SLC13A1*	NM_022444.3	flanking_5UTR	-38,434
rs5755683	2.75E-04	0.225	0.754	0.478	22	34,005,537	*HMG2L1*	NM_001003681	intron	0
rs3909209	2.76E-04	0.225	0.754	0.089	7	29,464,155	*CHN2*	NM_004067	intron	0
rs17273267	2.77E-04	0.225	0.754	0.155	19	59,401,140	*RPS9*	NM_001013.3	intron	-816
rs985196	2.77E-04	-0.225	0.754	0.438	19	24,159,893	*ZNF539*	NM_203282.1	flanking_3UTR	-55,400
rs12297883	2.78E-04	0.224	0.754	0.080	12	128,961,683	*TMEM132D*	NM_133448	upstream	7,846
rs11857366	2.79E-04	-0.224	0.754	0.498	15	97,081,324	*IGF1R*	NM_000875.2	intron	-12,465
rs9886752	2.79E-04	-0.226	0.754	0.136	9	138,754,316	*LCN10*	NM_001001712.1	coding	[93/5]
rs6568947	2.80E-04	0.225	0.754	0.250	6	116,978,189	*FAM26D*	NM_153036	intron	0
rs4595529	2.80E-04	0.224	0.754	0.243	11	43,972,442	*LOC390110*	NM_001031854	upstream	53,665
rs10017324	2.82E-04	-0.224	0.754	0.181	4	60,911,394	*LPHN3*	NM_015236.3	flanking_5UTR	-1,134,040
rs1150433	2.82E-04	0.225	0.754	0.142	3	165,135,904	*SI*	NM_001041.1	flanking_3UTR	-1,043,476
rs6878187	2.82E-04	0.224	0.754	0.130	5	143,658,983	*KCTD16*	NM_020768.1	intron	-91,681
rs8108034	2.82E-04	0.224	0.754	0.141	19	44,505,693	*GMFG*	NM_004877.1	flanking_3UTR	-5,146
rs2834255	2.83E-04	-0.225	0.754	0.113	21	34,046,123	*ITSN1*	NM_001001132.1	intron	-42
rs138744	2.83E-04	0.225	0.754	0.478	22	34,029,883	*TOM1*	NM_005488	intron	0
rs134415	2.83E-04	-0.230	0.754	0.162	22	33,913,297	*ISX*	NM_001008494	downstream	97,283
rs4239152	2.84E-04	0.224	0.754	0.389	17	66,882,635	---	ENST0000033456	upstream	246,079
rs964059	2.85E-04	0.228	0.754	0.165	8	124,904,538	*FAM91A1*	NM_144963.1	flanking_3UTR	-10,275
rs1016512	2.86E-04	-0.224	0.754	0.076	8	8,919,230	*THEX1*	NM_153332.2	intron	-3,846
rs1411192	2.86E-04	-0.225	0.754	0.124	20	799,769	*ANGPT4*	NM_015985.2	flanking_3UTR	-1,527
rs4783101	2.86E-04	-0.224	0.754	0.389	16	83,535,498	*ZDHHC7*	NM_017740.1	flanking_3UTR	-30,075
rs17023205	2.86E-04	0.224	0.754	0.255	2	83,194,134	---	ENST0000030779	downstream	255,738
rs9807288	2.86E-04	0.224	0.754	0.085	18	41,226,152	*SLC14A2*	NM_007163	upstream	232,476

rs2830988	2.87E-04	0.224	0.754	0.255	21	27,799,163	---	ENST0000032498	upstream	508,409
rs16994418	2.87E-04	-0.224	0.754	0.154	20	797,993	*FAM110A*	NM_031424	downstream	23,071
rs4141899	2.88E-04	-0.225	0.754	0.474	1	104,349,140	---	ENST0000038870	downstream	2,798,589
rs10483481	2.89E-04	-0.224	0.754	0.496	14	36,515,582	*SLC25A21*	NM_030631	intron	0
rs17000162	2.89E-04	-0.224	0.754	0.225	19	24,159,989	*ZNF254*	NM_203282	downstream	57,367
rs511715	2.92E-04	0.224	0.754	0.289	6	163,408,927	*PACRG*	NM_152410.1	intron	-5,584
rs6688289	2.92E-04	0.224	0.754	0.451	1	195,881,681	*DENND1B*	NM_144977.1	intron	-185
rs961534	2.92E-04	-0.227	0.754	0.416	4	183,641,562	---	ENST0000038904	intron	0
rs2043074	2.93E-04	0.229	0.754	0.230	2	83,154,608	*LOC388965*	NM_001013648.3	flanking_5UTR	-1,216,710
rs2159245	2.93E-04	0.228	0.754	0.350	4	10,159,291	*MIST*	NM_052964.1	intron	-6,289
rs491391	2.96E-04	-0.224	0.754	0.069	2	141,078,528	*LRP1B*	NM_018557.1	intron	-2,850
rs1893154	2.97E-04	-0.223	0.754	0.199	18	895,125	*ADCYAP1*	NM_001117.2	flanking_5UTR	-262
rs4831119	2.98E-04	-0.223	0.754	0.236	3	117,107,130	*LSAMP*	NM_002338	intron	0
rs870041	2.99E-04	-0.223	0.754	0.371	10	2,075,671	*ADARB2*	NM_018702	upstream	306,001
rs16861578	2.99E-04	-0.225	0.754	0.088	1	18,320,077	*IGSF21*	NM_032880	intron	0
rs6861373	3.00E-04	0.223	0.754	0.380	5	11,773,017	*CTNND2*	NM_001332.2	intron	-12,231
rs2833626	3.00E-04	0.223	0.754	0.168	21	32,349,723	*C21orf45*	NM_018944	downstream	212,680
rs2247524	3.01E-04	-0.223	0.754	0.069	1	243,999,415	*SMYD3*	NM_022743	intron	0
rs501537	3.02E-04	-0.224	0.754	0.412	19	16,286,503	*KLF2*	NM_016270.2	flanking_5UTR	-10,148
rs16869915	3.04E-04	-0.224	0.754	0.077	4	20,339,878	*KCNIP4*	NM_147183.2	flanking_3UTR	-264
rs2010447	3.04E-04	0.223	0.754	0.228	2	81,280,698	*CTNNA2*	NM_004389.2	flanking_3UTR	-551,282
rs2831966	3.04E-04	-0.225	0.754	0.284	21	28,998,651	*N6AMT1*	NM_182749	downstream	167,749
rs9966361	3.06E-04	-0.223	0.754	0.373	18	26,646,487	*DSC3*	NM_001941.2	flanking_3UTR	-178,542
rs2071163	3.07E-04	-0.224	0.754	0.203	12	11,928,348	*ETV6*	NM_001987.3	intron	-298
rs2977826	3.07E-04	-0.223	0.754	0.266	8	6,727,484	*DEFB1*	NM_005218.3	flanking_5UTR	-4,545
rs7657084	3.08E-04	0.223	0.754	0.089	4	180,518,626	*AGA*	NM_000027.2	flanking_5UTR	-1,918,041
rs7810239	3.08E-04	-0.223	0.754	0.348	7	122,633,447	*SLC13A1*	NM_022444.3	flanking_5UTR	-6,186
rs7855326	3.09E-04	-0.223	0.754	0.411	9	112,108,614	*TXNDC8*	NM_001003936	intron	0
rs11740912	3.10E-04	-0.224	0.754	0.396	5	99,189,846	*UNQ1912*	NM_198507.1	flanking_5UTR	-709,177
rs2960765	3.10E-04	0.223	0.754	0.136	7	141,619,238	*TRY1*	NM_001001317.1	flanking_5UTR	-14,891
rs473758	3.10E-04	0.223	0.754	0.053	15	53,169,882	*C15orf15*	NM_016304.2	flanking_3UTR	-90,922
rs10903651	3.11E-04	-0.223	0.754	0.484	10	2,079,982	*ADARB2*	NM_018702.1	flanking_5UTR	-310,312
rs6833291	3.11E-04	-0.223	0.754	0.180	4	60,909,102	*LPHN3*	NM_015236.3	flanking_5UTR	-1,136,332
rs9369494	3.11E-04	0.224	0.754	0.301	6	44,606,450	*CDC5L*	NM_001253.2	flanking_3UTR	-83,692
rs9303662	3.11E-04	-0.223	0.754	0.282	17	28,657,314	*ACCN1*	NM_001094	intron	0
rs1009776	3.13E-04	0.223	0.754	0.148	X	144,696,669	*SLITRK2*	NM_032539.2	flanking_5UTR	-10,373
rs4533267	3.13E-04	-0.223	0.754	0.339	15	98,603,794	*ADAMTS17*	NM_139057.1	intron	-8,070
rs4821392	3.13E-04	0.223	0.754	0.484	22	34,020,532	*HMG2L1*	NM_005487.3	3UTR	[887/1268]
rs4936275	3.14E-04	0.224	0.754	0.181	11	112,870,294	*DRD2*	NM_000795	upstream	19,191
rs1027348	3.15E-04	0.223	0.754	0.123	5	41,822,023	*OXCT1*	NM_000436.2	intron	-7,839
rs11659943	3.15E-04	-0.223	0.754	0.085	18	20,396,088	*HRH4*	NM_021624.2	flanking_3UTR	-82,169
rs11999442	3.15E-04	-0.223	0.754	0.080	9	6,644,050	*GLDC*	NM_000170.1	flanking_5UTR	-8,400
rs12904944	3.15E-04	0.223	0.754	0.371	15	65,148,828	*SMAD3*	NM_005902.3	intron	-3,076
rs8029174	3.15E-04	0.225	0.754	0.343	15	59,323,714	*RORA*	NM_134261.1	flanking_5UTR	-14,920
rs9292826	3.15E-04	0.223	0.754	0.123	5	41,864,426	*OXCT1*	NM_000436.2	intron	-11,884
rs17060069	3.16E-04	-0.223	0.754	0.082	8	29,295,757	*DUSP4*	NM_057158	upstream	33,516

rs157566	3.17E-04	0.223	0.754	0.242	5	89,414,482	*CETN3*	NM_004365.2	flanking_3UTR	-310,802
rs11653885	3.17E-04	0.222	0.754	0.373	17	66,896,712	---	ENST0000038852	downstream	77,740
rs12135374	3.17E-04	-0.222	0.754	0.138	1	245,318,076	*ZNF669*	NM_024804	downstream	11,811
rs10520423	3.18E-04	0.223	0.754	0.109	4	180,498,192	*AGA*	NM_000027.2	flanking_5UTR	-1,897,607
rs2235148	3.18E-04	0.222	0.754	0.480	22	33,994,941	*HMG2L1*	NM_005487.3	intron	-3,345
rs894274	3.18E-04	0.225	0.754	0.467	18	49,383,487	*DCC*	NM_005215.1	flanking_3UTR	-72,201
rs9391949	3.18E-04	-0.223	0.754	0.389	6	2,397,023	*C6orf195*	NM_152554.1	flanking_3UTR	-170,123
rs965036	3.18E-04	-0.223	0.754	0.219	6	20,099,022	*ID4*	NM_001546.2	flanking_3UTR	-150,128
rs760513	3.18E-04	0.222	0.754	0.480	22	33,999,809	*HMG2L1*	NM_001003681	intron	0
rs1575188	3.18E-04	-0.222	0.754	0.143	6	86,014,415	---	ENST0000036966	upstream	483,459
rs2591119	3.19E-04	0.223	0.754	0.104	18	26,621,746	*DSC3*	NM_001941.2	flanking_3UTR	-203,283
rs9816541	3.20E-04	0.222	0.754	0.292	3	189,218,381	*FLJ42393*	NM_207488.1	flanking_5UTR	-160,644
rs5970594	3.20E-04	0.223	0.754	0.369	X	23,536,394	*PRDX4*	NM_006406	upstream	59,090
rs189189	3.21E-04	0.222	0.754	0.054	5	122,520,422	*PPIC*	NM_000943.4	flanking_5UTR	-120,098
rs335194	3.21E-04	0.222	0.754	0.054	5	122,548,108	*PPIC*	NM_000943.4	flanking_5UTR	-147,784
rs163041	3.21E-04	0.222	0.754	0.054	5	122,540,598	---	ENST0000026136	intron	0
rs35503	3.23E-04	0.222	0.754	0.359	12	113,988,903	*TBX3*	NM_005996.3	flanking_5UTR	-382,551
rs3754741	3.24E-04	0.222	0.754	0.212	2	173,761,465	*ZAK*	NM_016653.1	intron	-868
rs7164440	3.24E-04	0.224	0.754	0.261	15	91,385,619	*RGMA*	NM_020211.1	flanking_3UTR	-2,022
rs17541472	3.24E-04	-0.222	0.754	0.103	2	47,133,660	*TTC7A*	NM_020458	intron	0
rs2289399	3.24E-04	0.222	0.754	0.212	2	173,763,977	*ZAK*	NM_133646	intron	0
rs17117177	3.25E-04	0.223	0.754	0.143	15	24,416,725	*GABRB3*	NM_021912.2	intron	-829
rs3801032	3.25E-04	0.222	0.754	0.134	7	6,029,054	*JTV1*	NM_006303.2	intron	-406
rs13200840	3.26E-04	0.225	0.754	0.084	6	74,827,659	*CD109*	NM_133493.1	flanking_3UTR	-236,150
rs1422715	3.26E-04	0.222	0.754	0.115	5	143,668,829	*KCTD16*	NM_020768.1	intron	-101,527
rs1444211	3.26E-04	0.222	0.754	0.158	12	10,820,218	*TAS2R7*	NM_023919.2	flanking_3UTR	-25,180
rs1477320	3.26E-04	-0.222	0.754	0.111	5	169,472,782	*FOXI1*	NM_144769.1	flanking_3UTR	-3,477
rs2278865	3.26E-04	-0.222	0.754	0.473	4	122,371,347	*TNIP3*	NM_024873.2	flanking_5UTR	-66,421
rs1219400	3.27E-04	-0.222	0.754	0.221	11	121,259,498	*BRCC2*	NM_001001786.1	flanking_3UTR	-231,774
rs11155475	3.27E-04	-0.222	0.754	0.330	6	146,914,378	*RAB32*	NM_006834	intron	0
rs17512269	3.27E-04	0.222	0.754	0.134	1	91,949,650	*TGFBR3*	NM_003243	intron	0
rs12794600	3.27E-04	0.222	0.754	0.150	11	11,344,289	*GALNTL4*	NM_198516	intron	0
rs10253693	3.29E-04	-0.222	0.754	0.460	7	122,613,192	*SLC13A1*	NM_022444.3	intron	-4,801
rs11808911	3.29E-04	0.222	0.754	0.055	1	207,938,600	*HSD11B1*	NM_181755	intron	0
rs477397	3.31E-04	-0.222	0.754	0.442	18	41,665,188	*CD33L3*	NM_213602.1	intron	-5,445
rs6733728	3.31E-04	-0.222	0.754	0.223	2	104,536,785	*POU3F3*	NM_006236.1	flanking_5UTR	-301,616
rs6846710	3.31E-04	0.222	0.754	0.275	4	187,619,536	*MTNR1A*	NM_005958.3	flanking_3UTR	-72,267
rs1014764	3.31E-04	-0.222	0.754	0.076	8	92,689,050	*RUNX1T1*	NM_004349	downstream	351,278
rs17079623	3.32E-04	0.222	0.754	0.190	18	64,544,220	*TXNDC10*	NM_019022.3	flanking_5UTR	-10,887
rs318720	3.32E-04	0.222	0.754	0.243	19	11,358,104	*EPOR*	NM_000121.2	flanking_5UTR	-2,085
rs11536481	3.33E-04	-0.222	0.754	0.172	7	8,705,890	*NXPH1*	NM_152745.1	intron	-51,273
rs4589058	3.33E-04	0.222	0.754	0.357	1	69,766,100	*DEPDC1*	NM_017779	upstream	1,030,714
rs2092029	3.36E-04	0.222	0.754	0.480	22	33,982,241	*HMG2L1*	NM_005487.3	flanking_5UTR	-1,248
rs9511057	3.36E-04	0.222	0.754	0.303	13	23,576,732	*SPATA13*	NM_153023.1	flanking_5UTR	-56,155
rs9651864	3.36E-04	0.222	0.754	0.411	12	13,376,607	*C12orf36*	NM_182558.1	flanking_3UTR	-38,683
---	3.36E-04	0.222	0.754	0.102	4	156,804,916	*GUCY1A3*	NM_000856	upstream	2,884

rs2215362	3.38E-04	-0.221	0.754	0.462	7	48,944,106	*ABCA13*	NM_152701.2	flanking_3UTR	-286,469
rs766045	3.38E-04	-0.222	0.754	0.084	8	3,968,386	*CSMD1*	NM_033225.3	intron	-91,357
rs6460942	3.43E-04	0.227	0.754	0.065	7	12,387,514	*TMEM106B*	NM_018374.2	flanking_3UTR	-144,103
rs9889419	3.43E-04	0.221	0.754	0.355	17	53,828,258	*RNF43*	NM_017763	intron	0
rs7296684	3.43E-04	-0.221	0.754	0.292	12	14,598,395	*FLJ22662*	NM_024829	intron	0
rs2810131	3.44E-04	-0.222	0.754	0.244	13	71,496,562	*DACH1*	NM_004392.4	flanking_5UTR	-157,231
rs1337441	3.44E-04	0.227	0.754	0.401	1	163,840,474	*MGST3*	NM_004528	upstream	26,248
rs3744093	3.45E-04	0.222	0.754	0.349	17	53,847,799	*RNF43*	NM_017763.3	coding	[113/138]
rs7521536	3.46E-04	-0.222	0.754	0.285	1	171,731,888	*SLC9A11*	NM_178527.2	flanking_3UTR	-4,346
rs13377643	3.47E-04	0.221	0.754	0.100	12	130,083,746	*GPR133*	NM_198827.2	intron	-18,908
rs780354	3.49E-04	-0.221	0.754	0.192	3	59,747,500	*FHIT*	NM_002012.1	intron	-34,413
rs1499088	3.49E-04	-0.221	0.754	0.462	3	3,351,689	*TRNT1*	NM_016000	downstream	184,147
rs11023482	3.49E-04	-0.221	0.754	0.076	11	15,214,865	---	ENST0000037955	intron	0
rs714753	3.52E-04	-0.221	0.754	0.154	2	104,534,320	*POU3F3*	NM_006236.1	flanking_5UTR	-304,081
rs7162912	3.53E-04	0.222	0.754	0.372	15	65,148,362	*SMAD3*	NM_005902.3	intron	-2,610
rs9394373	3.53E-04	-0.221	0.754	0.095	6	36,773,617	*FLJ43093*	NM_207498.1	5UTR	[11/53]
rs9980601	3.53E-04	-0.221	0.754	0.295	21	28,998,303	*N6AMT1*	NM_013240	downstream	168,097
rs17436486	3.54E-04	0.222	0.754	0.162	11	11,344,431	*GALNTL4*	NM_198516.1	flanking_3UTR	-6,207
rs7772997	3.54E-04	-0.223	0.754	0.061	6	168,080,731	*MLLT4*	NM_001040000	intron	0
rs7723992	3.55E-04	0.221	0.754	0.122	5	41,889,271	*OXCT1*	NM_000436.2	intron	-7
rs420350	3.55E-04	-0.221	0.754	0.322	6	15,871,772	*DTNBP1*	NM_183041	upstream	100,693
rs7869617	3.55E-04	-0.221	0.754	0.409	9	112,100,260	*TXN*	NM_003329	upstream	41,519
rs10128586	3.56E-04	-0.221	0.754	0.190	11	35,202,483	*CD44*	NM_001001392.1	intron	-2,628
rs7221046	3.57E-04	0.221	0.754	0.397	17	66,878,906	---	ENST0000033456	upstream	249,808
rs134381	3.57E-04	-0.221	0.754	0.156	22	33,901,497	*ISX*	NM_001008494	downstream	85,483
rs2440327	3.58E-04	-0.221	0.754	0.333	15	50,848,448	*ONECUT1*	NM_004498.1	intron	-11,112
rs12851714	3.58E-04	-0.221	0.754	0.256	X	144,792,680	*CXorf1*	NM_004709	downstream	73,620
rs17200269	3.59E-04	-0.221	0.754	0.204	3	16,373,801	*RFTN1*	NM_015150	intron	0
rs9916773	3.59E-04	-0.221	0.754	0.101	17	74,382,213	*TIMP2*	NM_003255	intron	0
rs1867099	3.59E-04	-0.220	0.754	0.371	9	131,489,350	*PRRX2*	NM_016307	intron	0
rs4448975	3.60E-04	-0.220	0.754	0.083	16	73,259,755	*RFWD3*	NM_018124.3	flanking_5UTR	-1,475
rs4816322	3.60E-04	-0.222	0.754	0.295	21	28,994,789	*N6AMT1*	NM_013240	downstream	171,611
rs12065426	3.61E-04	-0.220	0.754	0.286	1	171,736,728	*SLC9A11*	NM_178527.2	3UTR	[494/127]
rs1743829	3.61E-04	-0.220	0.754	0.087	10	102,918,121	*TLX1*	NM_005521.2	flanking_3UTR	-30,585
rs6656271	3.61E-04	0.220	0.754	0.480	1	209,148,026	*KCNH1*	NM_172362.1	intron	-11,579
rs10487324	3.62E-04	-0.222	0.754	0.297	7	109,823,837	*IMMP2L*	NM_032549.1	flanking_3UTR	-266,509
rs17135170	3.63E-04	0.220	0.754	0.174	16	1,075,404	*C1QTNF8*	NM_207419.1	flanking_3UTR	-2,823
rs6075069	3.63E-04	0.220	0.754	0.103	20	16,476,914	*C20orf23*	NM_024704.3	intron	-19,829
rs1843784	3.63E-04	0.221	0.754	0.147	11	11,343,170	*GALNTL4*	NM_198516	intron	0
rs2028486	3.63E-04	-0.220	0.754	0.243	8	28,515,387	*FZD3*	NM_017412	downstream	27,680
rs1060700	3.64E-04	0.221	0.754	0.407	7	12,242,343	*TMEM106B*	NM_018374.2	3UTR	[4216/1068]
rs6023583	3.66E-04	0.220	0.754	0.063	20	52,817,438	*DOK5*	NM_177959.2	flanking_3UTR	-116,321
rs856411	3.66E-04	0.220	0.754	0.491	20	50,692,583	*ZNF218*	NM_173485.2	flanking_5UTR	-329,770
rs10206724	3.68E-04	-0.221	0.754	0.485	2	44,038,849	*LRPPRC*	NM_133259.2	intron	-755
rs2704788	3.68E-04	0.220	0.754	0.429	2	146,227,765	*ZFHX1B*	NM_014795.2	flanking_5UTR	-1,233,379
rs289471	3.68E-04	-0.221	0.754	0.315	15	96,009,224	*ARRDC4*	NM_183376.1	flanking_5UTR	-295,723

rs4713334	3.68E-04	0.220	0.754	0.147	6	11,378,921	---	ENST0000033924	intron	0
rs7936320	3.68E-04	-0.220	0.754	0.076	11	93,900,605	*FUT4*	NM_002033	upstream	16,170
rs9427712	3.70E-04	-0.220	0.754	0.149	1	199,307,821	*CACNA1S*	NM_000069.1	intron	-685
rs4451090	3.70E-04	-0.221	0.754	0.385	5	99,177,452	---	ENST0000036250	upstream	207,955
rs6547678	3.70E-04	0.223	0.754	0.137	2	86,466,686	*JMJD1A*	NM_018433	upstream	55,268
rs13022923	3.70E-04	-0.220	0.754	0.053	2	51,533,843	---	ENST0000037825	upstream	424,934
rs11167785	3.71E-04	0.220	0.754	0.222	5	142,043,913	*FGF1*	NM_033136.1	intron	-1,793
rs10511903	3.72E-04	-0.222	0.754	0.140	9	32,334,825	*ACO1*	NM_002197.1	flanking_5UTR	-39,825
rs11597635	3.73E-04	-0.220	0.754	0.245	10	131,601,355	*EBF3*	NM_001005463.1	intron	-35,252
rs932680	3.73E-04	0.221	0.754	0.350	6	44,609,851	*RUNX2*	NM_001015051	upstream	888,041
rs12667678	3.74E-04	-0.221	0.754	0.489	7	132,184,203	*CHCHD3*	NM_017812.1	intron	-10,434
rs2436365	3.74E-04	0.220	0.754	0.502	5	104,610,740	*RAB9P1*	NR_000039.1	flanking_3UTR	-147,042
rs9829153	3.74E-04	0.224	0.754	0.310	3	7,880,601	*GRM7*	NM_000844	downstream	177,487
rs10866835	3.75E-04	-0.220	0.754	0.325	8	23,663,678	*NKX3-1*	NM_006167.2	flanking_5UTR	-67,283
rs12778834	3.75E-04	-0.220	0.754	0.125	10	130,792,327	*MGMT*	NM_002412.2	flanking_5UTR	-363,129
rs2875764	3.75E-04	-0.220	0.754	0.056	3	97,594,869	*ARL6*	NM_032146.3	flanking_5UTR	-1,371,416
rs6797214	3.75E-04	-0.220	0.754	0.056	3	97,604,076	---	ENST0000033211	upstream	1,464,518
rs6502114	3.75E-04	-0.220	0.754	0.385	17	78,042,965	*FOXK2*	NM_004514	upstream	27,918
rs1451635	3.76E-04	-0.220	0.754	0.355	4	151,887,976	*LRBA*	NM_006726.1	intron	-12,008
rs4888213	3.77E-04	-0.221	0.754	0.109	16	72,918,430	*PSMD7*	NM_002811.3	flanking_3UTR	-20,743
rs7912109	3.77E-04	0.220	0.754	0.145	10	101,225,803	*GOT1*	NM_002079	upstream	45,463
rs4904117	3.78E-04	-0.220	0.754	0.067	14	83,011,020	*SEL1L*	NM_005065.3	flanking_5UTR	-1,941,134
rs3754744	3.80E-04	0.223	0.754	0.205	2	173,710,310	*ZAK*	NM_133646	intron	0
rs12898502	3.82E-04	0.220	0.754	0.322	15	97,111,975	*IGF1R*	NM_000875.2	intron	-43,116
rs1623581	3.82E-04	0.220	0.754	0.356	11	91,946,276	*MTNR1B*	NM_005959.3	flanking_5UTR	-396,161
rs3138191	3.82E-04	-0.220	0.754	0.069	12	90,089,474	*DCN*	NM_001920	intron	0
rs4932745	3.83E-04	-0.219	0.754	0.438	19	24,092,561	*ZNF254*	NM_004876.1	intron	-8,335
rs9322971	3.83E-04	0.219	0.754	0.480	14	36,516,110	*SLC25A21*	NM_030631.1	intron	-102,150
rs17046742	3.84E-04	-0.219	0.754	0.083	2	25,019,329	*RBJ*	NM_016544.1	flanking_3UTR	-680
rs254850	3.84E-04	-0.219	0.754	0.076	5	158,599,309	*UBLCP1*	NM_145049.1	flanking_5UTR	-23,567
rs2186435	3.84E-04	-0.219	0.754	0.116	21	24,806,299	---	ENST0000035518	downstream	22,664
rs6998067	3.84E-04	0.220	0.754	0.442	8	118,020,702	*LOC441376*	NM_001025357	intron	0
rs7857056	3.84E-04	-0.219	0.754	0.212	9	32,357,887	*ACO1*	NM_002197	upstream	16,731
rs1944586	3.85E-04	-0.220	0.754	0.401	18	44,040,204	*KIAA0427*	NM_014772.1	flanking_5UTR	-279,221
rs2039372	3.86E-04	-0.220	0.754	0.126	10	130,790,379	*MGMT*	NM_002412.2	flanking_5UTR	-365,077
rs4740396	3.86E-04	-0.219	0.754	0.130	9	132,907,628	*LAMC3*	NM_006059.2	intron	-685
rs10203313	3.87E-04	0.220	0.754	0.325	2	28,568,254	*PLB1*	NM_153021.3	flanking_5UTR	-4,232
rs10736960	3.87E-04	0.219	0.754	0.246	10	2,821,743	*PFKP*	NM_002627	upstream	277,969
rs10869954	3.88E-04	-0.219	0.754	0.322	9	79,481,827	*GNA14*	NM_004297.2	flanking_5UTR	-28,784
rs10734762	3.88E-04	0.219	0.754	0.154	12	27,736,441	*PPFIBP1*	NM_003622	intron	0
rs2488401	3.89E-04	0.219	0.754	0.217	1	195,969,024	*DENND1B*	NM_144977.1	intron	-2,315
rs4045101	3.89E-04	-0.219	0.754	0.433	19	24,238,309	*ZNF539*	NM_203282.1	flanking_3UTR	-133,816
rs487419	3.89E-04	-0.221	0.754	0.066	2	141,094,077	*LRP1B*	NM_018557.1	intron	-18,399
rs10223190	3.91E-04	0.220	0.754	0.089	5	171,511,985	*STK10*	NM_005990.2	intron	-4,248
rs7190693	3.91E-04	0.219	0.754	0.297	16	6,815,405	*A2BP1*	NM_018723.2	intron	-170,753
rs7244876	3.91E-04	-0.220	0.754	0.177	18	4,384,577	*DLGAP1*	NM_004746.2	flanking_5UTR	-514,442

rs17178928	3.91E-04	0.219	0.754	0.368	17	66,876,277	---	ENST0000038852	downstream	57,305
rs9853758	3.92E-04	-0.219	0.754	0.237	3	117,107,546	*LSAMP*	NM_002338	intron	0
rs10234372	3.94E-04	-0.219	0.754	0.197	7	81,602,655	*CACNA2D1*	NM_000722.2	intron	-1,232
rs7724279	3.94E-04	-0.220	0.754	0.292	5	31,993,642	*PDZK3*	NM_015022.2	intron	-25,376
rs7932035	3.94E-04	0.222	0.754	0.154	11	39,576,707	---	ENST0000038693	downstream	168,297
rs2643365	3.95E-04	-0.220	0.754	0.414	15	31,658,779	*RYR3*	NM_001036.2	intron	-690
rs11814493	3.95E-04	-0.221	0.754	0.096	10	123,307,175	*FGFR2*	NM_022970	intron	0
rs17740678	3.95E-04	0.219	0.754	0.060	17	53,581,844	---	ENST0000026891	upstream	5,844
rs2573286	3.96E-04	-0.220	0.754	0.381	13	45,382,941	*KIAA0853*	NM_015070.2	flanking_3UTR	-51,374
rs9503185	3.96E-04	0.221	0.754	0.185	6	2,358,910	*GMDS*	NM_001500.2	flanking_5UTR	-168,065
rs10032685	3.97E-04	-0.219	0.754	0.054	4	100,610,340	*ADH7*	NM_000673.3	flanking_5UTR	-34,792
rs12967400	3.97E-04	0.219	0.754	0.297	18	44,033,665	*KIAA0427*	NM_014772.1	flanking_5UTR	-285,760
rs1795033	3.97E-04	0.219	0.754	0.199	1	212,360,617	*PROX1*	NM_002763.3	flanking_3UTR	-84,228
rs9435730	3.97E-04	0.219	0.754	0.200	1	17,178,490	*MFAP2*	NM_017459.1	intron	-1,094
rs6014161	3.98E-04	0.219	0.754	0.062	20	52,815,620	*DOK5*	NM_177959.2	flanking_3UTR	-114,503
rs9307870	3.98E-04	-0.219	0.754	0.171	4	151,349,501	*DCAMKL2*	NM_152619.1	intron	-5,010
rs17327860	3.98E-04	-0.219	0.754	0.168	3	55,437,613	*LRTM1*	NM_020678	upstream	500,501
rs10061965	3.99E-04	0.221	0.754	0.275	5	26,346,575	*CDH9*	NM_016279.2	flanking_3UTR	-569,891
rs3801748	3.99E-04	-0.219	0.754	0.462	7	81,597,444	*CACNA2D1*	NM_000722.2	intron	-6,443
rs17019466	4.00E-04	0.219	0.754	0.085	3	2,745,509	*CNTN4*	NM_175607.1	intron	-7,390
rs4731919	4.00E-04	-0.219	0.754	0.284	7	132,250,526	*CHCHD3*	NM_017812	intron	0
rs370971	4.01E-04	-0.219	0.754	0.263	20	41,804,509	*FAM112A*	NM_001008901.1	flanking_5UTR	-15,453
rs7045765	4.01E-04	0.219	0.754	0.322	9	34,964,974	*DNAJB5*	NM_012266.3	flanking_5UTR	-14,811
rs6013611	4.01E-04	-0.220	0.754	0.126	20	51,087,206	*TSHZ2*	NM_173485	intron	0
rs1474557	4.02E-04	0.219	0.754	0.480	22	34,020,179	*HMG2L1*	NM_005487.3	3UTR	[534/1621]
rs10500224	4.02E-04	-0.219	0.754	0.440	19	24,148,147	*ZNF254*	NM_203282	downstream	45,525
rs2385424	4.02E-04	-0.220	0.754	0.330	X	144,811,845	*CXorf1*	NM_004709	downstream	92,785
rs1010094	4.04E-04	0.224	0.755	0.124	12	27,735,190	*PPFIBP1*	NM_003622.2	intron	-759
rs2369754	4.04E-04	-0.219	0.755	0.423	5	99,184,261	*UNQ1912*	NM_198507.1	flanking_5UTR	-714,762
rs3768286	4.05E-04	0.219	0.756	0.300	1	232,648,514	*TARBP1*	NM_005646.2	intron	-658
rs4706229	4.06E-04	-0.219	0.756	0.141	6	86,010,579	*NT5E*	NM_002526.1	flanking_5UTR	-205,949
rs740400	4.07E-04	-0.218	0.756	0.147	19	62,378,616	*DUXA*	NM_001012729.1	flanking_5UTR	-7,948
rs6710284	4.09E-04	0.218	0.756	0.225	2	81,294,037	*CTNNA2*	NM_004389	downstream	565,529
rs10494113	4.10E-04	0.218	0.756	0.418	1	110,485,810	*SLC6A17*	NM_001010898.1	flanking_5UTR	-8,821
rs4235251	4.10E-04	-0.218	0.756	0.357	4	151,838,479	*LRBA*	NM_006726.1	intron	-14,160
rs4727563	4.10E-04	0.219	0.756	0.084	7	102,615,805	*DPY19L2P2*	NM_182634	intron	0
rs4861770	4.11E-04	0.218	0.756	0.451	4	180,529,655	*AGA*	NM_000027.2	flanking_5UTR	-1,929,070
rs4861771	4.11E-04	0.218	0.756	0.451	4	180,529,785	---	ENST0000036485	downstream	964,827
rs3754743	4.12E-04	0.218	0.756	0.201	2	173,710,338	*ZAK*	NM_016653.1	intron	-32,441
rs1864152	4.14E-04	-0.218	0.756	0.304	16	86,199,574	*JPH3*	NM_020655.2	intron	-4,939
rs6816145	4.14E-04	-0.219	0.756	0.136	4	164,638,444	*FLJ11184*	NM_018352.1	intron	-3,038
rs9827155	4.14E-04	-0.218	0.756	0.239	3	117,110,045	*LSAMP*	NM_002338.2	intron	-55,891
rs10137783	4.16E-04	-0.218	0.756	0.067	14	96,955,972	*VRK1*	NM_003384.2	flanking_3UTR	-538,268
rs6494074	4.16E-04	0.219	0.756	0.493	15	30,025,001	*CHRNA7*	NM_000746.2	flanking_5UTR	-85,017
rs431852	4.17E-04	-0.219	0.756	0.128	6	95,073,137	---	ENST0000036408	downstream	427,471
rs2305699	4.18E-04	0.219	0.756	0.134	16	55,707,146	*CPNE2*	NM_152727.4	intron	-183

rs17255321	4.19E-04	0.218	0.756	0.069	1	182,827,164	*C1orf21*	NM_030806	intron	0
rs3912371	4.20E-04	-0.218	0.756	0.069	14	96,957,242	*VRK1*	NM_003384	downstream	539,539
rs10203818	4.20E-04	-0.218	0.756	0.192	2	13,285,084	---	ENST0000038800	downstream	80,363
rs6959577	4.21E-04	0.218	0.756	0.433	7	106,380,831	*PIK3CG*	NM_002649	downstream	46,030
rs12510298	4.23E-04	0.218	0.756	0.309	4	96,955,501	*UNC5C*	NM_003728	upstream	266,470
rs1901968	4.25E-04	-0.218	0.756	0.426	5	99,173,642	---	ENST0000036348	upstream	873,291
rs1376368	4.25E-04	0.221	0.756	0.459	1	105,250,662	---	ENST0000038870	downstream	1,897,067
rs1031881	4.26E-04	-0.219	0.756	0.193	6	124,962,839	*TCBA1*	NM_153355.2	intron	-58,192
rs10762361	4.26E-04	0.218	0.756	0.449	10	71,732,868	*LRRC20*	NM_018239.2	intron	-1,598
rs4398231	4.26E-04	0.218	0.756	0.337	2	28,581,758	*PLB1*	NM_153021	intron	0
rs12621347	4.26E-04	0.218	0.756	0.337	2	28,579,944	*PLB1*	NM_153021	intron	0
rs1715282	4.26E-04	0.219	0.756	0.269	11	20,687,337	*NELL1*	NM_006157	intron	0
rs564719	4.26E-04	0.218	0.756	0.062	15	53,195,599	*C15orf15*	NM_016304	downstream	65,214
rs10517360	4.27E-04	-0.218	0.756	0.058	4	56,330,641	*EXOC1*	NM_018261.3	flanking_5UTR	-83,932
rs2451039	4.27E-04	0.219	0.756	0.244	4	169,597,133	*PALLD*	NM_016081.2	flanking_5UTR	-57,659
rs12639090	4.28E-04	-0.218	0.756	0.311	3	151,597,194	*TSC22D2*	NM_014779	upstream	11,618
rs10420762	4.29E-04	-0.218	0.756	0.440	19	24,074,071	*ZNF254*	NM_004876.1	intron	-6,511
rs2293847	4.29E-04	-0.218	0.756	0.365	14	79,396,835	*NRXN3*	NM_004796.3	intron	-924
rs10781417	4.30E-04	-0.218	0.756	0.475	9	78,892,495	*LOC442425*	NM_001013735.1	flanking_3UTR	-66,806
rs4688077	4.31E-04	0.218	0.756	0.231	3	119,313,783	*IGSF11*	NM_001015887.1	flanking_3UTR	-788,384
rs17083352	4.32E-04	0.218	0.756	0.112	9	82,242,760	*TLE4*	NM_007005.3	flanking_3UTR	-711,282
rs9357230	4.32E-04	-0.220	0.756	0.273	6	36,963,360	*C6orf89*	NM_152734	intron	0
rs905089	4.33E-04	-0.218	0.756	0.333	X	144,811,785	*CXorf1*	NM_004709	downstream	92,725
rs7295355	4.34E-04	-0.218	0.756	0.404	12	126,558,379	*SLC15A4*	NM_145648.1	flanking_3UTR	-1,285,334
rs9673444	4.34E-04	-0.218	0.756	0.407	16	19,095,757	*SYT17*	NM_016524.2	intron	-3,356
rs10748985	4.34E-04	-0.221	0.756	0.122	10	110,260,794	---	ENST0000036320	downstream	1,049,377
rs7530860	4.35E-04	0.217	0.756	0.353	1	245,084,807	*AHCTF1*	NM_015446.3	intron	-848
rs929381	4.36E-04	0.217	0.756	0.442	7	81,594,249	*CACNA2D1*	NM_000722	intron	0
rs7220651	4.37E-04	0.217	0.756	0.457	17	9,531,044	*WDRPUH*	NM_145054.2	flanking_3UTR	-43,617
rs1328384	4.38E-04	-0.217	0.756	0.475	6	39,399,785	*KCNK16*	NM_032115.2	flanking_5UTR	-1,491
rs4395097	4.38E-04	-0.219	0.756	0.079	16	73,259,931	*RFWD3*	NM_018124.3	flanking_5UTR	-1,651
rs2160954	4.38E-04	-0.217	0.756	0.357	3	7,861,502	*GRM7*	NM_181875	downstream	153,596
rs10426952	4.39E-04	-0.217	0.756	0.234	19	61,698,665	*ZNF471*	NM_020813.1	flanking_5UTR	-12,359
rs7593737	4.39E-04	-0.218	0.756	0.495	2	192,348,869	*SDPR*	NM_004657.4	flanking_3UTR	-58,412
rs2697299	4.39E-04	-0.217	0.756	0.395	2	197,789,323	---	ENST0000033720	intron	0
rs7876326	4.40E-04	0.219	0.756	0.363	X	23,536,189	*PRDX4*	NM_006406	upstream	59,295
rs271742	4.41E-04	0.217	0.756	0.332	1	232,732,942	*TARBP1*	NM_005646.2	flanking_5UTR	-51,470
rs6942458	4.41E-04	-0.218	0.756	0.255	7	81,620,450	*CACNA2D1*	NM_000722.2	intron	-16,522
rs7355998	4.41E-04	-0.217	0.756	0.161	3	131,249,017	*LOC650293*	NM_001040071	upstream	24,867
rs8113266	4.43E-04	-0.217	0.756	0.197	19	23,852,820	*LOC388524*	NM_001005472.1	flanking_3UTR	-50,043
rs6759184	4.43E-04	-0.218	0.756	0.424	2	44,085,473	*LRPPRC*	NM_133259	upstream	8,883
rs7177058	4.43E-04	0.218	0.756	0.215	15	30,018,232	---	ENST0000038412	downstream	10,303
rs4072059	4.43E-04	-0.217	0.756	0.130	18	43,818,960	*ZBTB7C*	NM_001039360	intron	0
rs1155491	4.44E-04	0.217	0.756	0.107	8	69,262,624	*DEPDC2*	NM_024870.2	intron	-3,887
rs7184854	4.44E-04	0.217	0.756	0.237	16	15,666,407	*NDE1*	NM_017668.1	intron	-188
rs11200981	4.44E-04	-0.217	0.756	0.089	10	86,049,212	*RGR*	NM_001012722	downstream	40,288

rs3823612	4.44E-04	0.221	0.756	0.404	7	12,225,280	*TMEM106B*	NM_018374	intron	0
rs10103263	4.45E-04	-0.217	0.756	0.118	8	20,729,747	*LZTS1*	NM_021020.1	flanking_5UTR	-572,664
rs10924899	4.47E-04	-0.217	0.756	0.071	1	245,292,967	*ZNF670*	NM_033213.2	intron	-15,552
rs1343065	4.47E-04	-0.217	0.756	0.171	10	54,346,417	*MBL2*	NM_000242.1	flanking_5UTR	-144,951
rs13431612	4.47E-04	-0.217	0.756	0.171	2	69,654,083	*AAK1*	NM_014911.2	intron	-16,469
rs17390362	4.47E-04	-0.217	0.756	0.351	2	197,803,016	*ANKRD44*	NM_153697.1	flanking_5UTR	-32,009
rs758642	4.47E-04	-0.218	0.756	0.303	17	3,733,656	*CAMKK1*	NM_032294.2	intron	-133
rs17040798	4.48E-04	0.217	0.756	0.272	2	83,131,744	*LOC388965*	NM_001013648.3	flanking_5UTR	-1,239,574
rs17162040	4.48E-04	0.217	0.756	0.103	1	27,755,814	*AHDC1*	NM_001029882.1	intron	-1,990
rs7928669	4.49E-04	0.217	0.756	0.382	11	120,626,658	*SC5DL*	NM_006918.3	flanking_5UTR	-41,979
rs4668712	4.50E-04	-0.217	0.756	0.123	2	11,143,958	*FLJ33534*	NM_182586.1	flanking_3UTR	-12,619
rs1931805	4.50E-04	-0.217	0.756	0.482	6	62,688,822	*KHDRBS2*	NM_152688	intron	0
rs17072378	4.52E-04	-0.217	0.756	0.138	4	182,990,518	---	ENST0000031530	downstream	309,283
rs6949033	4.53E-04	-0.217	0.756	0.426	7	107,282,453	*DLD*	NM_000108	upstream	36,394
rs903850	4.54E-04	-0.217	0.756	0.185	5	167,552,828	*WWC1*	NM_015238.1	flanking_5UTR	-98,842
rs877394	4.54E-04	-0.217	0.756	0.232	19	61,693,949	---	ENST0000029999	intron	0
rs2612366	4.55E-04	-0.217	0.756	0.337	18	71,657,068	*SDCCAG33*	NM_005786.3	flanking_3UTR	-526,402
rs3857700	4.55E-04	-0.217	0.756	0.405	7	70,726,671	*WBSCR17*	NM_022479	intron	0
rs1017747	4.55E-04	-0.217	0.756	0.337	18	71,657,150	*LOC284274*	NM_001037331	upstream	388,504
rs12527264	4.56E-04	-0.217	0.756	0.134	6	37,607,820	*C6orf129*	NM_138493.1	flanking_5UTR	-32,144
rs1368320	4.56E-04	-0.217	0.756	0.480	5	155,460,803	*SGCD*	NM_172244	upstream	225,531
rs4239153	4.57E-04	0.218	0.756	0.388	17	66,882,690	*SOX9*	NM_000346.2	flanking_5UTR	-746,066
rs10496293	4.58E-04	0.217	0.756	0.121	2	83,195,665	*LOC388965*	NM_001013648.3	flanking_5UTR	-1,175,653
rs13147065	4.58E-04	0.217	0.756	0.136	4	128,888,256	*SLC25A31*	NM_031291.1	intron	-2,852
rs5771104	4.58E-04	0.217	0.756	0.411	22	48,993,966	*SELO*	NM_031454.1	intron	-2,307
rs513186	4.59E-04	0.217	0.756	0.245	18	9,872,587	*TXNDC2*	NM_032243.4	flanking_5UTR	-3,215
rs5957888	4.59E-04	0.219	0.756	0.111	X	121,070,765	---	ENST0000038602	upstream	731,195
rs8129139	4.59E-04	-0.217	0.756	0.292	21	28,980,804	*N6AMT1*	NM_182749	downstream	185,596
rs13247910	4.60E-04	-0.217	0.756	0.464	7	48,943,489	*ABCA13*	NM_152701.2	flanking_3UTR	-285,852
rs4817242	4.61E-04	-0.218	0.756	0.295	21	28,985,620	*C21orf100*	NM_145033.1	flanking_3UTR	-151,072
rs11597093	4.63E-04	-0.218	0.756	0.126	10	130,793,014	*MKI67*	NM_002417	upstream	978,375
rs17089603	4.64E-04	-0.217	0.756	0.307	8	23,668,901	*NKX3-1*	NM_006167.2	flanking_5UTR	-72,506
rs3807325	4.64E-04	-0.216	0.756	0.306	7	132,174,002	*CHCHD3*	NM_017812	intron	0
rs7692637	4.65E-04	-0.217	0.756	0.303	4	139,809,649	*CCRN4L*	NM_012118.2	flanking_5UTR	-346,744
rs3097865	4.65E-04	-0.216	0.756	0.161	5	155,460,663	*SGCD*	NM_172244	upstream	225,671
rs28374096	4.66E-04	-0.217	0.756	0.270	9	135,224,921	*SURF4*	NM_033161	intron	0
rs2612420	4.67E-04	0.217	0.756	0.487	4	171,640,357	*AADAT*	NM_182662.1	flanking_5UTR	-392,410
rs6555888	4.67E-04	-0.216	0.756	0.156	5	169,468,728	*FOXI1*	NM_144769.1	3UTR	[534/577]
rs7548727	4.67E-04	0.216	0.756	0.130	1	2,855,638	---	ENST0000032139	intron	0
rs16833439	4.67E-04	-0.217	0.756	0.333	3	183,936,809	*ATP11B*	NM_014616	upstream	57,176
rs1492483	4.68E-04	0.221	0.756	0.402	3	113,330,675	*GCET2*	NM_152785.3	intron	-1,077
rs7385225	4.68E-04	-0.216	0.756	0.063	7	101,828,684	*PRKRIP1*	NM_024653.1	intron	-1,584
rs3744108	4.69E-04	0.217	0.756	0.360	17	53,940,871	*MTMR4*	NM_004687.3	coding	[43/53]
rs11609302	4.70E-04	-0.216	0.756	0.087	12	118,014,078	*KIAA1853*	NM_194286	intron	0
rs4270965	4.71E-04	0.217	0.756	0.356	8	83,791,597	*SNX16*	NM_152837.1	flanking_5UTR	-874,607
rs6585765	4.71E-04	-0.216	0.756	0.094	10	123,596,492	*ATE1*	NM_001001976.1	intron	-5,724

rs17029662	4.72E-04	-0.217	0.756	0.302	4	101,066,128	*DNAJB14*	NM_024920.3	UTR	[1138/1143]
rs4781679	4.73E-04	0.217	0.756	0.238	16	15,668,934	*NDE1*	NM_017668.1	intron	-137
rs9729834	4.73E-04	-0.217	0.756	0.114	1	82,811,926	*LPHN2*	NM_012302	downstream	581,231
rs17117178	4.73E-04	0.217	0.756	0.109	15	24,418,202	*GABRB3*	NM_000814	intron	0
rs7568410	4.73E-04	0.216	0.756	0.190	2	147,246,805	---	ENST0000035946	downstream	181,777
rs7232189	4.74E-04	-0.216	0.756	0.100	18	4,151,831	---	ENST0000032010	downstream	1,075,532
rs1344164	4.75E-04	-0.217	0.756	0.155	4	64,381,774	*SRD5A2L2*	NM_001010874.2	flanking_3UTR	-444,241
rs12355928	4.76E-04	0.216	0.756	0.082	10	101,237,541	*NKX2-3*	NM_145285.1	flanking_5UTR	-45,159
rs2039371	4.78E-04	-0.216	0.756	0.123	10	130,790,196	*MKI67*	NM_002417	upstream	975,557
rs6968293	4.78E-04	-0.216	0.756	0.464	7	48,934,801	---	ENST0000032499	exon	0
rs6854952	4.79E-04	-0.217	0.756	0.146	4	162,005,231	*FSTL5*	NM_020116.2	flanking_3UTR	-519,268
rs7802995	4.79E-04	0.217	0.756	0.203	7	43,611,958	*STK17A*	NM_004760.1	intron	-2,422
rs1423858	4.80E-04	0.216	0.756	0.402	16	74,688,256	*CNTNAP4*	NM_138994.1	flanking_5UTR	-212,994
rs7004769	4.80E-04	0.216	0.756	0.141	8	9,225,005	*PPP1R3B*	NM_024607.1	flanking_5UTR	-179,389
rs7276179	4.80E-04	-0.216	0.756	0.297	21	28,994,319	*C21orf100*	NM_145033.1	flanking_3UTR	-159,771
rs854342	4.80E-04	0.216	0.756	0.245	14	24,409,289	*STXBP6*	NM_014178.6	intron	-13,086
rs1468804	4.80E-04	0.216	0.756	0.408	7	12,242,033	*TMEM106B*	NM_018374	3UTR	0
rs6075068	4.80E-04	0.217	0.756	0.104	20	16,465,950	*C20orf23*	NM_024704	intron	0
rs10047474	4.81E-04	0.216	0.756	0.484	11	11,352,065	*GALNTL4*	NM_198516.1	intron	-1,313
rs17046168	4.81E-04	0.219	0.756	0.069	12	77,911,935	*SYT1*	NM_005639	intron	0
rs134409	4.82E-04	-0.216	0.756	0.155	22	33,910,096	*HMG2L1*	NM_005487.3	flanking_5UTR	-73,393
rs191997	4.82E-04	-0.216	0.756	0.179	21	26,755,155	*CYYR1*	NM_052954	downstream	5,247
rs5972371	4.82E-04	0.216	0.756	0.092	X	31,247,616	*DMD*	NM_004010	intron	0
rs867560	4.82E-04	0.218	0.756	0.452	9	128,505,054	*LMX1B*	NM_002316	downstream	6,503
rs2031351	4.83E-04	-0.216	0.756	0.274	13	25,457,034	*ATP8A2*	NM_016529.3	intron	-16,217
rs548726	4.83E-04	0.216	0.756	0.203	1	5,836,208	*NPHP4*	NM_015102.2	flanking_3UTR	-9,249
rs10121536	4.83E-04	0.216	0.756	0.288	9	1,438,017	---	ENST0000038224	upstream	303,148
rs6442013	4.83E-04	0.216	0.756	0.136	3	46,675,717	*ALS2CL*	NM_182775	downstream	9,966
rs4884983	4.84E-04	-0.216	0.756	0.484	13	71,501,002	*DACH1*	NM_004392.4	flanking_5UTR	-161,671
rs4917187	4.84E-04	-0.216	0.756	0.462	7	48,943,898	*ABCA13*	NM_152701.2	flanking_3UTR	-286,261
rs10063424	4.85E-04	-0.216	0.756	0.074	5	169,468,100	*FOXI1*	NM_144769.1	coding	[184/93]
rs16927566	4.85E-04	0.220	0.756	0.073	8	62,647,414	*ASPH*	NM_004318	intron	0
rs16914891	4.86E-04	0.216	0.757	0.147	9	29,488,319	*LINGO2*	NM_152570	upstream	828,036
rs13393256	4.89E-04	-0.216	0.757	0.258	2	47,140,263	*TTC7A*	NM_020458.1	intron	-1,149
rs3739873	4.89E-04	-0.216	0.757	0.362	9	34,968,431	*DNAJB5*	NM_012266.3	flanking_5UTR	-11,354
rs6010202	4.89E-04	0.218	0.757	0.428	22	48,986,304	*SELO*	NM_031454.1	intron	-569
rs6859984	4.89E-04	0.216	0.757	0.405	5	75,805,324	*IQGAP2*	NM_006633.1	intron	-12,074
rs11120921	4.90E-04	-0.216	0.757	0.131	1	7,407,893	*CAMTA1*	NM_015215.1	intron	-42,584
rs6589877	4.90E-04	0.216	0.757	0.101	11	120,701,161	*SC5DL*	NM_006918.3	flanking_3UTR	-16,582
rs2100281	4.91E-04	0.216	0.757	0.129	4	187,617,051	*MTNR1A*	NM_005958.3	flanking_3UTR	-74,752
rs12667662	4.92E-04	-0.215	0.757	0.085	7	8,477,489	*NXPH1*	NM_152745.1	intron	-35,567
rs3861395	4.92E-04	-0.215	0.757	0.243	6	139,664,238	*TXLNB*	NM_153235.2	flanking_5UTR	-9,389
rs6779517	4.92E-04	0.215	0.757	0.290	3	48,359,392	*FBXW12*	NM_207102.1	flanking_5UTR	-29,321
rs10435414	4.92E-04	-0.215	0.757	0.270	7	132,145,839	*CHCHD3*	NM_017812	intron	0
rs5972370	4.93E-04	0.215	0.757	0.089	X	31,247,458	*DMD*	NM_004010	intron	0
rs4845626	4.96E-04	0.215	0.758	0.237	1	152,690,109	*IL6R*	NM_181359.1	intron	-1,029

rs359439	4.96E-04	-0.217	0.758	0.429	5	173,230,505	*CPEB4*	NM_030627	upstream	18,290
rs10402888	4.97E-04	-0.219	0.758	0.256	19	23,944,575	*ZNF254*	NM_004876.1	flanking_5UTR	-117,241
rs12505720	4.97E-04	0.215	0.758	0.449	4	180,538,373	---	ENST0000036485	downstream	973,415
rs10743140	4.97E-04	-0.215	0.758	0.091	11	10,381,993	*AMPD3*	NM_000480	upstream	46,807
rs326789	4.97E-04	-0.215	0.758	0.199	11	29,598,856	*METT5D1*	NM_152636	downstream	1,287,337
rs1991139	4.98E-04	-0.216	0.758	0.140	8	3,965,896	---	ENST0000038721	downstream	420,395
rs3765097	4.99E-04	-0.215	0.758	0.429	1	235,684,380	*RYR2*	NM_001035.1	coding	[66/117]
rs4850776	5.00E-04	-0.215	0.758	0.361	2	197,840,696	---	ENST0000033720	intron	0
rs11666543	5.01E-04	0.215	0.758	0.152	19	59,403,793	*RPS9*	NM_001013	downstream	470
rs726107	5.02E-04	-0.216	0.758	0.171	X	131,487,656	*MBNL3*	NM_133486.1	flanking_5UTR	-86,257
rs4775378	5.03E-04	0.216	0.758	0.420	15	59,353,507	*RORA*	NM_134261.1	flanking_5UTR	-44,713
rs6665230	5.03E-04	-0.215	0.758	0.471	1	231,544,491	*KIAA1804*	NM_032435.1	intron	-4,320
rs17641214	5.04E-04	0.221	0.758	0.065	14	46,536,895	*MAMDC1*	NM_182830.2	intron	-37,069
rs6830371	5.04E-04	-0.215	0.758	0.496	4	4,818,596	*MSX1*	NM_002448.1	flanking_5UTR	-93,711
rs13028463	5.04E-04	-0.215	0.758	0.058	2	48,700,678	*SALF*	NM_172311	intron	0
rs2412986	5.06E-04	0.215	0.758	0.236	22	29,191,640	*SEC14L3*	NM_174975.3	intron	-681
rs5753161	5.06E-04	0.215	0.758	0.236	22	29,192,839	*SEC14L3*	NM_174975.3	intron	-130
rs757663	5.06E-04	0.215	0.758	0.236	22	29,192,763	*SEC14L3*	NM_174975.3	intron	-206
rs5753152	5.06E-04	0.215	0.758	0.236	22	29,176,623	*SEC14L3*	NM_174975	downstream	8,595
rs4820851	5.06E-04	0.215	0.758	0.236	22	29,184,663	*SEC14L3*	NM_174975	downstream	555
rs11612353	5.07E-04	0.215	0.758	0.150	12	13,875,865	*GRIN2B*	NM_000834.2	intron	-34,134
rs2612561	5.07E-04	0.215	0.758	0.482	18	41,236,957	*SLC14A2*	NM_007163.2	flanking_5UTR	-211,807
rs2829689	5.08E-04	0.215	0.759	0.482	21	25,596,347	*C21orf42*	NM_058184.1	flanking_3UTR	-83,663
rs2100969	5.08E-04	-0.215	0.759	0.409	1	235,679,897	*RYR2*	NM_001035	intron	0
rs17347021	5.10E-04	-0.215	0.759	0.065	X	103,951,525	*IL1RAPL2*	NM_017416.1	intron	-161,193
rs675785	5.10E-04	0.215	0.759	0.072	5	41,684,092	*OXCT1*	NM_000436.2	flanking_3UTR	-81,832
rs7602691	5.11E-04	0.215	0.759	0.062	2	184,933,283	*C2orf10*	NM_194250.1	flanking_5UTR	-238,055
rs12519026	5.12E-04	0.215	0.759	0.495	5	20,856,652	*CDH18*	NM_004934.2	flanking_5UTR	-839,606
rs16827514	5.12E-04	0.215	0.759	0.178	2	231,254,994	*CAB39*	NM_016289.2	flanking_5UTR	-30,915
rs1963788	5.12E-04	0.215	0.759	0.275	7	81,570,650	*CACNA2D1*	NM_000722.2	intron	-13,646
rs12375315	5.13E-04	0.215	0.759	0.486	8	13,598,057	*C8orf48*	NM_001007090.1	flanking_3UTR	-127,890
rs7294678	5.14E-04	0.215	0.759	0.136	12	7,357,208	*M160*	NM_174941.3	flanking_3UTR	-41,618
rs11761043	5.14E-04	-0.215	0.759	0.389	7	16,026,726	---	ENST0000038212	upstream	68,579
rs11799574	5.16E-04	0.215	0.759	0.105	1	182,657,902	*C1orf21*	NM_030806.3	intron	-34,777
rs12145326	5.16E-04	0.215	0.759	0.105	1	182,661,579	*C1orf21*	NM_030806	intron	0
rs9637198	5.17E-04	-0.217	0.759	0.280	21	45,644,510	*COL18A1*	NM_130445.1	flanking_5UTR	-5,015
rs1561513	5.20E-04	-0.215	0.759	0.187	2	104,539,560	*POU3F3*	NM_006236.1	flanking_5UTR	-298,841
rs2310956	5.20E-04	0.215	0.759	0.173	4	131,527,035	---	ENST0000036302	downstream	120,240
rs8078327	5.21E-04	0.215	0.759	0.489	17	9,542,596	*WDRPUH*	NM_145054.2	flanking_3UTR	-55,169
rs10516088	5.22E-04	-0.214	0.759	0.194	5	171,083,028	*FBXW11*	NM_012300.2	flanking_3UTR	-138,133
rs2291845	5.23E-04	0.214	0.759	0.129	11	5,686,156	*TRIM22*	NM_006074.2	intron	-77
rs4860053	5.23E-04	-0.217	0.759	0.174	4	60,907,604	---	ENST0000036490	downstream	525,124
rs10477189	5.24E-04	0.214	0.759	0.281	5	142,028,899	*FGF1*	NM_033136.1	intron	-16,807
rs7708257	5.24E-04	0.214	0.759	0.281	5	142,035,721	*FGF1*	NM_000800	intron	0
rs12851264	5.24E-04	-0.215	0.759	0.260	X	144,792,510	*CXorf1*	NM_004709	downstream	73,450
rs1981868	5.24E-04	0.214	0.759	0.464	16	79,505,497	*CENPN*	NM_018455	upstream	92,161

rs7765276	5.25E-04	0.214	0.759	0.183	6	159,469,377	*FNDC1*	NM_032532.1	flanking_5UTR	-41,040
rs9372465	5.25E-04	0.214	0.759	0.221	6	116,968,576	*FAM26D*	NM_153036	intron	0
rs3930151	5.25E-04	0.215	0.759	0.104	3	9,348,721	*SRGAP3*	NM_001033116	upstream	82,410
rs5956545	5.25E-04	-0.214	0.759	0.109	X	122,423,179	*GRIA3*	NM_000828	intron	0
rs17769226	5.26E-04	-0.215	0.759	0.096	18	36,985,835	*PIK3C3*	NM_002647.2	flanking_5UTR	-803,362
rs3733870	5.26E-04	-0.215	0.759	0.198	5	159,411,119	*TTC1*	NM_003314.1	intron	-351
rs9835698	5.26E-04	-0.216	0.759	0.304	3	11,711,267	*VGLL4*	NM_014667	intron	0
rs11977093	5.28E-04	0.215	0.759	0.087	7	18,452,438	*HDAC9*	NM_014707.1	flanking_5UTR	-49,456
rs7014732	5.29E-04	-0.214	0.759	0.174	8	90,089,280	*RIPK2*	NM_003821	upstream	749,893
rs2816428	5.30E-04	0.214	0.759	0.246	8	5,811,749	*MCPH1*	NM_024596.1	flanking_5UTR	-464,731
rs37037	5.31E-04	0.215	0.759	0.178	16	57,222,103	*CNOT1*	NM_016284.3	flanking_5UTR	-852
rs6911665	5.31E-04	0.216	0.759	0.188	6	2,359,739	---	ENST0000036637	upstream	42,766
rs2278443	5.32E-04	-0.214	0.759	0.440	19	24,138,372	*ZNF539*	NM_203282.1	flanking_3UTR	-33,879
rs8100720	5.32E-04	-0.214	0.759	0.440	19	24,150,231	*ZNF539*	NM_203282.1	flanking_3UTR	-45,738
rs7242620	5.32E-04	-0.215	0.759	0.335	18	41,244,929	*SETBP1*	NM_015559	downstream	346,158
rs1830180	5.32E-04	-0.214	0.759	0.243	19	24,247,803	*ZNF254*	NM_203282	downstream	145,181
rs7596196	5.32E-04	0.214	0.759	0.196	2	192,169,542	*MYO1B*	NM_012223	downstream	171,255
rs2829702	5.34E-04	0.214	0.759	0.482	21	25,620,980	*C21orf42*	NM_058184.1	flanking_3UTR	-59,030
rs2829703	5.34E-04	0.214	0.759	0.482	21	25,621,099	*C21orf42*	NM_058184.1	flanking_3UTR	-58,911
rs10511968	5.34E-04	0.214	0.759	0.428	9	71,535,423	---	ENST000003772C	intron	0
rs9300589	5.34E-04	0.214	0.759	0.150	13	99,584,749	*PCCA*	NM_000282	intron	0
rs2243409	5.35E-04	-0.215	0.759	0.135	13	77,797,158	*POU4F1*	NM_006237.2	flanking_3UTR	-274,070
rs7508396	5.35E-04	-0.215	0.759	0.440	19	24,140,765	*ZNF539*	NM_203282.1	flanking_3UTR	-36,272
rs11146253	5.36E-04	0.215	0.759	0.254	10	133,876,202	*STK32C*	NM_173575.2	intron	-3,607
rs1497111	5.37E-04	-0.214	0.759	0.129	11	82,115,140	*MGC33846*	NM_175885.3	flanking_3UTR	-5,554
rs4629032	5.37E-04	0.214	0.759	0.129	18	11,158,361	*C18orf58*	NM_173817.1	flanking_5UTR	-277,671
rs10106829	5.39E-04	0.214	0.759	0.142	8	9,222,038	*PPP1R3B*	NM_024607.1	flanking_5UTR	-176,422
rs9678154	5.40E-04	-0.216	0.759	0.239	2	13,301,637	*TRIB2*	NM_021643.1	flanking_3UTR	-501,326
rs2540879	5.41E-04	0.214	0.759	0.316	14	90,777,948	*GPR68*	NM_003485.3	intron	-6,672
rs2391704	5.41E-04	0.216	0.759	0.196	7	28,912,352	*CREB5*	NM_001011666	downstream	80,316
rs1943536	5.42E-04	-0.214	0.759	0.391	18	26,622,034	*DSC3*	NM_001941.2	flanking_3UTR	-202,995
rs7695454	5.42E-04	-0.216	0.759	0.389	4	83,155,674	*RASGEF1B*	NM_152545	upstream	543,589
rs2830987	5.42E-04	0.215	0.759	0.245	21	27,798,286	---	ENST0000032498	upstream	509,286
rs7036096	5.43E-04	0.214	0.759	0.195	9	134,988,444	*RALGDS*	NM_006266.2	flanking_5UTR	-2,062
rs4077347	5.44E-04	-0.215	0.759	0.314	16	28,944,416	*LAT*	NM_001014988.1	flanking_3UTR	-34,811
rs6998928	5.44E-04	-0.214	0.759	0.475	8	23,649,933	*NKX3-1*	NM_006167.2	flanking_5UTR	-53,538
rs7737426	5.44E-04	-0.214	0.759	0.279	5	114,536,487	*TRIM36*	NM_018700	intron	0
rs10935749	5.45E-04	-0.214	0.759	0.389	3	150,582,265	*TM4SF1*	NM_014220.2	flanking_5UTR	-4,007
rs11899367	5.45E-04	0.214	0.759	0.347	2	134,510,726	*MGAT5*	NM_002410.2	flanking_5UTR	-214,912
rs3818509	5.45E-04	0.214	0.759	0.308	9	129,710,353	*ST6GALNAC4*	NM_175040.1	UTR	[33/138]
rs2063570	5.45E-04	-0.214	0.759	0.053	9	2,653,788	*VLDLR*	NM_001018056	downstream	9,303
rs895649	5.45E-04	0.214	0.759	0.304	11	119,256,795	*PVRL1*	NM_203285	upstream	152,150
rs12363999	5.47E-04	-0.214	0.759	0.391	11	120,649,324	*TECTA*	NM_005422	downstream	82,599
rs1359176	5.48E-04	0.218	0.759	0.331	9	9,740,139	*PTPRD*	NM_130393.1	flanking_5UTR	-731,404
rs962088	5.49E-04	-0.214	0.759	0.319	5	24,904,174	*CDH10*	NM_006727.2	flanking_5UTR	-223,506
rs4850779	5.49E-04	-0.214	0.759	0.360	2	197,842,433	---	ENST000003372C	intron	0

rs10520425	5.50E-04	0.214	0.759	0.085	4	180,522,429	---	ENST00000385760	downstream	1,120,525
rs10512566	5.51E-04	0.214	0.759	0.361	17	66,891,676	*SOX9*	NM_000346.2	flanking_5UTR	-737,080
rs4910841	5.52E-04	-0.216	0.759	0.330	11	5,690,170	*TRIM22*	NM_006074.2	flanking_3UTR	-1,501
rs9929993	5.52E-04	0.214	0.759	0.322	16	7,604,876	*A2BP1*	NM_145893	intron	0
rs10816609	5.52E-04	-0.214	0.759	0.440	9	109,819,240	---	ENST0000029781	upstream	182,309
rs1060126	5.53E-04	-0.215	0.759	0.295	12	21,546,098	*GOLT1B*	NM_016072.2	5UTR	[31/26]
rs571352	5.53E-04	-0.214	0.759	0.469	3	120,841,735	*POPDC2*	NM_022135.2	flanking_3UTR	-1,861
rs10065640	5.54E-04	0.214	0.759	0.158	5	59,857,355	*PART1*	NM_016590.2	flanking_3UTR	-34,506
rs2011057	5.54E-04	-0.214	0.759	0.085	1	82,830,743	*LPHN2*	NM_012302.2	flanking_3UTR	-600,048
rs12368715	5.55E-04	0.214	0.759	0.133	12	91,873,257	---	ENST0000038463	upstream	66,256
rs2058943	5.56E-04	-0.213	0.759	0.283	7	132,253,843	*CHCHD3*	NM_017812.1	intron	-32,798
rs7790787	5.56E-04	-0.213	0.759	0.283	7	132,257,711	*CHCHD3*	NM_017812	intron	0
rs9638627	5.56E-04	-0.213	0.759	0.380	7	70,693,047	*WBSCR17*	NM_022479	intron	0
rs17389549	5.57E-04	0.213	0.759	0.130	7	15,570,378	*FLJ16237*	NM_001004320.1	flanking_5UTR	-2,213
rs1956918	5.57E-04	-0.214	0.759	0.311	14	24,050,151	*CMA1*	NM_001836.2	flanking_5UTR	-2,840
rs2240345	5.57E-04	0.213	0.759	0.236	22	29,187,373	*SEC14L3*	NM_174975.3	coding	[76/93]
rs10056275	5.59E-04	0.213	0.759	0.111	5	59,865,222	*PART1*	NM_016590.2	flanking_3UTR	-42,373
rs4258212	5.59E-04	-0.213	0.759	0.417	1	235,684,022	*RYR2*	NM_001035	intron	0
rs10408143	5.60E-04	0.213	0.759	0.480	19	8,493,782	*MYO1F*	NM_012335.2	intron	-69
rs11718192	5.60E-04	-0.213	0.759	0.123	3	74,070,595	*PDZRN3*	NM_015009.1	flanking_5UTR	-313,833
rs1201214	5.60E-04	-0.214	0.759	0.133	4	152,035,233	*LRBA*	NM_006726.1	intron	-1,482
rs10267804	5.61E-04	-0.214	0.759	0.087	7	12,974,723	*ARL4*	NM_005738.2	flanking_3UTR	-277,639
rs10512565	5.61E-04	0.214	0.759	0.398	17	66,891,355	*SOX9*	NM_000346.2	flanking_5UTR	-737,401
rs6518289	5.61E-04	-0.217	0.759	0.119	21	46,611,430	*PCNT2*	NM_006031.3	coding	[503/52]
rs1384307	5.61E-04	0.214	0.759	0.264	3	84,508,585	*CADM2*	NM_153184	upstream	1,349,737
rs2052085	5.62E-04	-0.214	0.759	0.073	17	53,597,229	*OR4D2*	NM_001004707	upstream	4,532
rs4266582	5.64E-04	0.214	0.760	0.084	7	102,615,573	*FLJ36166*	NM_182634.2	intron	-2,353
rs9377003	5.67E-04	-0.214	0.760	0.331	6	146,902,882	*RAB32*	NM_006834.2	flanking_5UTR	-3,639
rs2413305	5.68E-04	-0.213	0.760	0.170	22	33,889,579	*ISX*	NM_001008494	downstream	73,565
rs10751505	5.68E-04	-0.213	0.760	0.299	9	135,185,195	*SURF4*	NM_033161	downstream	32,951
rs1997752	5.69E-04	0.213	0.760	0.118	20	8,735,329	*PLCB1*	NM_182734	intron	0
rs11127160	5.70E-04	0.213	0.760	0.330	2	28,570,459	*PLB1*	NM_153021.3	flanking_5UTR	-2,027
rs1384300	5.70E-04	0.213	0.760	0.104	3	84,439,336	*IGSF4D*	NM_153184.2	flanking_5UTR	-1,418,986
rs254256	5.70E-04	0.216	0.760	0.437	19	59,300,982	*NDUFA3*	NM_004542.1	intron	-71
rs623078	5.71E-04	0.213	0.760	0.071	5	41,702,394	*OXCT1*	NM_000436.2	flanking_3UTR	-63,530
rs10502557	5.72E-04	-0.214	0.760	0.238	18	26,882,254	*DSC3*	NM_024423.1	flanking_5UTR	-5,567
rs1472261	5.72E-04	0.213	0.760	0.199	5	32,923,367	*FLJ14054*	NM_024563.2	flanking_3UTR	-95,791
rs9828272	5.73E-04	0.213	0.760	0.138	3	165,194,191	*SI*	NM_001041.1	flanking_3UTR	-985,189
rs10114417	5.74E-04	0.213	0.760	0.395	9	1,449,663	*DMRT2*	NM_181872.1	flanking_3UTR	-402,111
rs9481069	5.74E-04	-0.213	0.760	0.112	6	110,857,679	*SLC22A16*	NM_033125.2	intron	-1,388
rs10013280	5.75E-04	0.214	0.760	0.330	4	148,008,159	*NYD-SP14*	NM_031956.1	coding	[59/26]
rs11220314	5.75E-04	0.213	0.760	0.197	11	98,904,400	*CNTN5*	NM_175566.1	intron	-27,686
rs16933641	5.75E-04	-0.216	0.760	0.082	10	77,967,980	*C10orf11*	NM_032024.2	intron	-18,993
rs10808296	5.76E-04	-0.213	0.760	0.322	7	81,565,714	*CACNA2D1*	NM_000722.2	intron	-13,562
rs4436381	5.76E-04	-0.213	0.760	0.444	1	235,701,726	*RYR2*	NM_001035	intron	0
rs17674942	5.79E-04	0.213	0.760	0.112	20	16,461,316	*C20orf23*	NM_024704.3	intron	-4,231

rs11649927	5.80E-04	0.213	0.760	0.154	17	13,707,875	*COX10*	NM_001303.2	flanking_5UTR	-205,569
rs12443134	5.80E-04	-0.213	0.760	0.078	15	66,795,305	*CORO2B*	NM_006091.1	intron	-601
rs2255598	5.80E-04	-0.214	0.760	0.242	19	24,248,960	*ZNF539*	NM_203282.1	flanking_3UTR	-144,467
rs390480	5.80E-04	0.213	0.760	0.469	2	32,363,576	*YIPF4*	NM_032312.2	intron	-5,480
rs13026867	5.81E-04	-0.213	0.760	0.219	2	151,639,685	*FLJ45645*	NM_198557.1	flanking_3UTR	-174,471
rs6815467	5.81E-04	-0.213	0.760	0.181	4	60,909,243	---	ENST0000036490	downstream	526,763
rs1391077	5.82E-04	-0.213	0.760	0.261	17	28,663,615	*ACCN1*	NM_183377.1	flanking_5UTR	-19,496
rs17560594	5.84E-04	0.213	0.760	0.071	14	46,530,953	*MAMDC1*	NM_182830.2	intron	-34,357
rs4363435	5.84E-04	-0.214	0.760	0.094	1	83,840,040	*TTLL7*	NM_024686.3	flanking_3UTR	-267,991
rs1803183	5.85E-04	-0.214	0.760	0.094	4	122,954,592	*EXOSC9*	NM_005033.1	coding	[121/60]
rs7868176	5.85E-04	-0.213	0.760	0.091	9	115,942,603	*COL27A1*	NM_032888.2	flanking_5UTR	-15,449
rs12338900	5.85E-04	-0.213	0.760	0.091	9	115,938,517	*COL27A1*	NM_032888	upstream	19,535
rs2281728	5.86E-04	-0.213	0.760	0.442	9	101,169,610	*NR4A3*	NM_173199	upstream	454,348
rs915391	5.86E-04	-0.213	0.760	0.072	10	121,055,375	*GRK5*	NM_005308	intron	0
rs10265711	5.88E-04	-0.213	0.760	0.458	7	122,615,738	*SLC13A1*	NM_022444.3	intron	-7,347
rs159606	5.88E-04	-0.213	0.760	0.333	5	17,427,898	*BASP1*	NM_006317.3	flanking_3UTR	-97,955
rs4732421	5.88E-04	0.215	0.760	0.409	7	81,574,203	*CACNA2D1*	NM_000722	intron	0
rs4807265	5.89E-04	0.214	0.760	0.240	19	2,392,106	*LMNB2*	NM_032737.2	intron	-2,577
rs7199746	5.89E-04	0.213	0.760	0.475	16	85,379,849	*FOXL1*	NM_005250.1	flanking_3UTR	-208,981
rs10488158	5.90E-04	0.213	0.760	0.051	7	12,475,419	*SCIN*	NM_033128.1	flanking_5UTR	-120,254
rs1135029	5.90E-04	0.213	0.760	0.098	11	71,966,939	*PDE2A*	NM_002599.1	coding	[14/92]
rs7898539	5.93E-04	-0.212	0.760	0.259	10	133,036,920	*TCERG1L*	NM_174937.1	flanking_5UTR	-36,944
rs12727642	5.94E-04	-0.213	0.760	0.067	1	7,969,259	*PARK7*	NM_007262.3	flanking_3UTR	-1,333
rs4952863	5.94E-04	0.214	0.760	0.485	2	47,111,481	*TTC7A*	NM_020458.1	intron	-1,454
rs6723119	5.94E-04	-0.213	0.760	0.493	2	44,040,708	*LRPPRC*	NM_133259.2	intron	-476
rs1631237	5.95E-04	0.213	0.760	0.360	17	54,089,694	*TEX14*	NM_198393.2	intron	-5,332
rs9868994	5.95E-04	-0.214	0.760	0.458	3	176,215,775	*NAALADL2*	NM_207015.1	intron	-81,499
rs4350155	5.95E-04	0.212	0.760	0.111	1	73,684,008	---	ENST0000038829	upstream	253,880
rs4435730	5.97E-04	-0.213	0.760	0.339	4	151,738,682	*LRBA*	NM_006726.1	intron	-927
rs6914206	5.97E-04	-0.214	0.760	0.458	6	156,505,013	*ARID1B*	NM_175863.2	flanking_5UTR	-635,765
rs10432046	5.98E-04	-0.212	0.760	0.275	17	28,661,025	*ACCN1*	NM_183377.1	flanking_5UTR	-16,906
rs7608097	5.98E-04	-0.212	0.760	0.429	2	173,008,299	*ITGA6*	NM_000210.1	intron	-7,355
rs6958721	5.98E-04	0.212	0.760	0.054	7	11,854,202	*TMEM106B*	NM_018374	upstream	366,751
rs2409318	5.99E-04	-0.213	0.760	0.271	21	29,021,276	*C21orf127*	NM_182749.1	flanking_3UTR	-149,070
rs9899718	5.99E-04	0.212	0.760	0.116	17	8,942,992	*NTN1*	NM_004822.1	intron	-63,863
rs11622479	5.99E-04	0.212	0.760	0.060	14	47,529,960	*MDGA2*	NM_182830	upstream	316,418
rs2143300	6.00E-04	-0.212	0.760	0.074	1	165,777,620	*CREG1*	NM_003851.2	3UTR	[746/538]
rs2860662	6.00E-04	-0.212	0.760	0.074	1	165,777,461	*CREG1*	NM_003851.2	3UTR	[587/697]
rs4341231	6.02E-04	0.215	0.760	0.337	9	136,732,782	*COL5A1*	NM_000093.2	intron	-56
rs386503	6.03E-04	0.212	0.760	0.321	5	157,992,095	*EBF*	NM_024007.2	flanking_3UTR	-65,911
rs2927457	6.05E-04	-0.212	0.760	0.065	19	49,948,787	*BCL3*	NM_005178.2	intron	-2,310
rs10023694	6.06E-04	-0.212	0.760	0.241	4	165,028,475	*1-Mar*	NM_017923	upstream	274,249
rs17014711	6.07E-04	-0.212	0.760	0.107	2	34,180,442	*MYADML*	NM_207329.1	flanking_5UTR	-373,667
rs7137120	6.07E-04	0.213	0.760	0.146	12	7,346,864	*PEX5*	NM_000319	downstream	91,522
rs6101283	6.07E-04	0.212	0.760	0.076	20	59,267,891	*CDH4*	NM_001794	intron	0
rs6101282	6.07E-04	0.212	0.760	0.076	20	59,267,601	*CDH4*	NM_001794	intron	0

rs1202582	6.07E-04	0.212	0.760	0.167	1	229,067,856	*C1orf198*	NM_032800	intron	0
rs635837	6.08E-04	-0.212	0.760	0.172	6	124,952,438	*TCBA1*	NM_153355.2	intron	-68,593
rs1893248	6.09E-04	-0.212	0.760	0.416	11	120,625,965	*SC5DL*	NM_006918.3	flanking_5UTR	-42,672
rs7556672	6.09E-04	0.212	0.760	0.315	2	2,584,764	*MYT1L*	NM_015025.2	flanking_5UTR	-270,712
rs7111498	6.09E-04	-0.212	0.760	0.167	11	109,409,990	---	ENST0000036615	upstream	93,386
rs11203189	6.10E-04	-0.212	0.760	0.276	21	42,158,495	*PRDM15*	NM_022115.2	intron	-1,983
rs7028285	6.10E-04	0.212	0.760	0.080	9	11,029,910	---	ENST0000036478	downstream	1,260,360
rs11708430	6.11E-04	-0.212	0.760	0.100	3	139,015,963	*CLDN18*	NM_001002026	upstream	184,385
rs3890761	6.12E-04	-0.212	0.760	0.205	4	165,049,201	*ANP32C*	NM_012403.1	flanking_3UTR	-288,408
rs313047	6.12E-04	-0.212	0.760	0.411	4	127,845,944	*FAT4*	NM_024582	downstream	1,213,571
rs739659	6.15E-04	0.212	0.760	0.418	2	130,042,855	*LOC151121*	NM_001033657.1	flanking_5UTR	-326,026
rs7707671	6.15E-04	-0.212	0.760	0.460	5	74,323,498	*GCNT4*	NM_016591.1	flanking_3UTR	-35,547
rs7808758	6.15E-04	0.212	0.760	0.440	7	70,008,405	*AUTS2*	NM_015570.1	flanking_3UTR	-112,615
rs10040935	6.15E-04	0.212	0.760	0.129	5	143,727,518	*KCTD16*	NM_020768	intron	0
rs975512	6.16E-04	0.213	0.760	0.378	1	110,492,100	*SLC6A17*	NM_001010898.1	flanking_5UTR	-2,531
rs16841426	6.16E-04	-0.212	0.760	0.254	2	157,825,546	*GALNT5*	NM_014568	intron	0
rs16841428	6.16E-04	-0.212	0.760	0.254	2	157,825,651	*GALNT5*	NM_014568	intron	0
rs2028383	6.17E-04	0.212	0.760	0.464	2	223,531,376	*ACSL3*	NM_004457.3	flanking_3UTR	-15,013
rs2158836	6.17E-04	-0.212	0.760	0.281	7	107,368,075	*LAMB1*	NM_002291.1	intron	-36
rs940794	6.18E-04	-0.212	0.760	0.498	2	217,164,812	*IGFBP2*	NM_000597.2	flanking_5UTR	-41,560
rs12121863	6.18E-04	0.212	0.760	0.188	1	196,052,043	*C1orf53*	NM_001024594	upstream	86,357
rs12102021	6.18E-04	0.213	0.760	0.066	15	22,491,886	*C15orf2*	NM_018958	downstream	16,308
rs10252772	6.20E-04	0.212	0.760	0.199	7	108,277,896	*LOC154907*	NM_001024607.1	flanking_3UTR	-33,378
rs6013938	6.20E-04	0.212	0.760	0.420	20	52,289,279	*PFDN4*	NM_002623.3	flanking_3UTR	-19,380
rs7942167	6.20E-04	0.212	0.760	0.380	11	120,626,711	*SC5DL*	NM_006918	upstream	41,979
rs10024417	6.21E-04	-0.212	0.760	0.143	4	20,269,504	*SLIT2*	NM_004787	downstream	39,618
rs4772269	6.22E-04	0.212	0.760	0.185	13	99,628,308	*PCCA*	NM_000282.2	intron	-20,713
rs7839608	6.22E-04	-0.212	0.760	0.359	8	69,220,526	*DEPDC2*	NM_024870.2	intron	-472
rs10947075	6.22E-04	0.212	0.760	0.152	6	11,377,452	---	ENST0000033924	intron	0
rs644917	6.23E-04	0.212	0.760	0.326	9	22,739,869	*DMRTA1*	NM_022160.1	flanking_3UTR	-297,397
rs1946554	6.23E-04	0.212	0.760	0.161	2	83,190,191	---	ENST0000036242	upstream	548,179
rs10742437	6.23E-04	-0.212	0.760	0.167	11	38,657,485	*RAG2*	NM_000536	upstream	2,081,123
rs7563874	6.24E-04	-0.212	0.760	0.130	2	215,033,939	---	ENST0000031271	upstream	236,765
rs3849698	6.26E-04	-0.212	0.760	0.332	5	42,989,559	*LOC389289*	NM_001014279.1	flanking_3UTR	-85,380
rs2143256	6.26E-04	-0.212	0.760	0.393	20	9,612,539	*PAK7*	NM_177990	intron	0
rs165533	6.27E-04	-0.212	0.760	0.424	5	173,225,583	*CPEB4*	NM_030627	upstream	23,212
rs4479268	6.27E-04	-0.212	0.760	0.078	16	73,782,234	*ZFP1*	NM_153688	downstream	18,754
rs1371357	6.30E-04	0.211	0.760	0.091	4	94,842,672	*GRID2*	NM_001510.1	intron	-66,712
rs4821387	6.30E-04	0.213	0.760	0.472	22	33,999,441	*HMG2L1*	NM_001003681	intron	0
rs1176943	6.31E-04	0.213	0.760	0.135	3	165,141,277	*SI*	NM_001041.1	flanking_3UTR	-1,038,103
rs1588322	6.32E-04	0.212	0.760	0.362	17	54,344,881	*PPM1E*	NM_014906.3	intron	-42,911
---	6.32E-04	0.211	0.760	0.176	16	57,149,779	*CNOT1*	NM_016284	intron	0
rs17199729	6.34E-04	-0.211	0.760	0.085	13	39,979,310	*FOXO1A*	NM_002015.2	flanking_3UTR	-48,507
rs17703063	6.34E-04	-0.211	0.760	0.094	7	123,291,556	*HYAL4*	NM_012269.1	intron	-3,957
rs7283848	6.35E-04	0.211	0.760	0.476	21	25,614,242	*C21orf42*	NM_058184.1	flanking_3UTR	-65,768
rs12455229	6.35E-04	-0.211	0.760	0.063	18	54,514,888	*MALT1*	NM_006785	intron	0

rs6738747	6.37E-04	-0.211	0.760	0.185	2	104,510,922	*POU3F3*	NM_006236.1	flanking_5UTR	-327,479
rs9920377	6.37E-04	0.211	0.760	0.167	15	81,926,142	*SH3GL3*	NM_003027	intron	0
rs7693278	6.38E-04	-0.212	0.760	0.369	4	152,029,075	*LRBA*	NM_006726.1	intron	-4,579
rs12802656	6.39E-04	0.211	0.760	0.418	11	16,490,991	*SOX6*	NM_033326.2	flanking_5UTR	-36,497
rs2104880	6.39E-04	-0.211	0.760	0.074	9	21,409,723	*IFNA8*	NM_002170.2	flanking_3UTR	-9,549
rs4426325	6.39E-04	-0.211	0.760	0.187	15	85,250,134	*AGBL1*	NM_152336	downstream	609,684
rs2073387	6.40E-04	-0.211	0.760	0.141	22	22,459,005	*SMARCB1*	NM_003073.3	flanking_5UTR	-145
rs2267029	6.40E-04	-0.211	0.760	0.141	22	22,447,916	*MMP11*	NM_005940.3	intron	-2,751
rs11077561	6.40E-04	0.211	0.760	0.366	17	66,892,501	---	ENST0000033456	upstream	236,213
rs5760022	6.40E-04	-0.211	0.760	0.141	22	22,464,394	*MIF*	NM_002415	intron	0
rs981230	6.41E-04	0.212	0.760	0.370	5	59,075,615	*PDE4D*	NM_006203.3	flanking_5UTR	-157,583
rs2561012	6.41E-04	0.212	0.760	0.173	19	57,639,479	---	ENST0000030108	intron	0
rs9898649	6.41E-04	-0.211	0.760	0.116	17	78,530,594	*B3GNTL1*	NM_001009905	intron	0
rs5000314	6.41E-04	0.212	0.760	0.218	2	147,248,064	---	ENST0000038551	upstream	549,945
rs2172491	6.42E-04	-0.211	0.760	0.100	11	129,928,515	*C11orf44*	NM_173580	upstream	119,569
rs10190161	6.43E-04	-0.212	0.760	0.475	2	44,041,333	*LRPPRC*	NM_133259	intron	0
rs7058595	6.44E-04	-0.211	0.760	0.260	X	144,790,928	*CXorf1*	NM_004709	downstream	71,868
rs10198351	6.45E-04	-0.212	0.760	0.473	2	10,442,231	*HPCAL1*	NM_002149.2	intron	-12,181
rs9907940	6.45E-04	0.211	0.760	0.364	17	54,018,841	*TEX14*	NM_198393.2	intron	-399
rs17146997	6.47E-04	0.211	0.760	0.319	7	22,561,034	*IL6*	NM_000600.1	flanking_5UTR	-172,311
rs3752596	6.47E-04	0.213	0.760	0.487	22	33,920,706	*HMG2L1*	NM_005487.3	flanking_5UTR	-62,783
rs28657937	6.47E-04	0.213	0.760	0.483	7	48,934,296	---	ENST0000032499	upstream	407
rs7190070	6.50E-04	0.213	0.760	0.094	16	66,911,484	*PRMT7*	NM_019023.1	intron	-1,346
rs2225749	6.50E-04	0.211	0.760	0.152	9	117,070,741	*1-Dec*	NM_017418	intron	0
rs1995274	6.51E-04	0.211	0.760	0.298	5	61,201,717	*FLJ37543*	NM_173667.1	flanking_3UTR	-163,598
rs6928850	6.51E-04	0.212	0.760	0.440	6	156,510,099	---	ENST0000038494	downstream	68,150
rs4729887	6.53E-04	0.211	0.760	0.085	7	102,615,906	*FLJ36166*	NM_182634.2	intron	-2,686
rs10491860	6.53E-04	0.212	0.760	0.337	9	1,439,360	---	ENST0000036551	downstream	115,407
rs17136078	6.53E-04	0.211	0.760	0.085	7	102,637,962	*DPY19L2P2*	NM_182634	CDS	0
rs842246	6.53E-04	0.211	0.760	0.457	3	16,268,429	*DPH3*	NM_206831	downstream	6,067
rs7695609	6.54E-04	0.211	0.760	0.134	4	138,106,284	*PCDH18*	NM_019035.2	flanking_3UTR	-554,001
rs10125991	6.55E-04	-0.214	0.760	0.346	9	131,487,840	*PRRX2*	NM_016307	intron	0
rs11929542	6.55E-04	0.211	0.760	0.054	3	84,477,491	*CADM2*	NM_153184	upstream	1,380,831
rs1019537	6.56E-04	-0.211	0.760	0.089	16	73,788,139	*CTRB2*	NM_001025200.2	flanking_3UTR	-7,363
rs1019539	6.56E-04	-0.211	0.760	0.089	16	73,788,240	*CTRB2*	NM_001025200.2	flanking_3UTR	-7,262
rs1630829	6.56E-04	0.211	0.760	0.359	1	245,090,489	*AHCTF1*	NM_015446.3	intron	-335
rs6495794	6.56E-04	-0.211	0.760	0.471	15	33,768,606	*ATPBD4*	NM_080650.2	flanking_5UTR	-142,950
rs17227696	6.56E-04	-0.211	0.760	0.082	5	80,523,878	*RASGRF2*	NM_006909	intron	0
rs1875402	6.58E-04	-0.211	0.760	0.274	2	95,212,081	*ZNF2*	NM_021088.2	3UTR	[499/1711]
rs11174055	6.59E-04	0.211	0.760	0.272	12	60,195,641	*FAM19A2*	NM_178539.3	flanking_3UTR	-192,666
rs2700842	6.59E-04	0.211	0.760	0.482	3	183,939,612	*ATP11B*	NM_014616.1	flanking_5UTR	-54,373
rs817691	6.59E-04	-0.211	0.760	0.179	20	59,189,035	*CDH4*	NM_001794.2	flanking_5UTR	-71,917
rs912767	6.59E-04	-0.211	0.760	0.281	1	171,723,068	*PRDX6*	NM_004905.2	intron	-429
rs7586619	6.59E-04	-0.211	0.760	0.243	2	104,543,267	---	ENST0000038452	upstream	172,857
rs6458503	6.59E-04	-0.211	0.760	0.212	6	12,967,542	*PHACTR1*	NM_030948	intron	0
rs17744700	6.61E-04	0.211	0.760	0.290	18	44,048,553	---	ENST0000032466	downstream	15,347

rs9892702	6.61E-04	0.211	0.760	0.203	17	5,512,605	*WSCD1*	NM_015253	upstream	402,053
rs2508467	6.62E-04	0.215	0.760	0.215	11	105,263,314	*GRIA4*	NM_000829.1	intron	-141
rs10067703	6.62E-04	-0.211	0.760	0.444	5	74,328,450	*GCNT4*	NM_016591	downstream	31,807
rs17404627	6.63E-04	-0.211	0.760	0.096	8	3,957,010	*CSMD1*	NM_033225.3	intron	-79,981
rs7786495	6.63E-04	0.215	0.760	0.209	7	12,185,673	*TMEM106B*	NM_018374.2	flanking_5UTR	-31,778
rs13264886	6.64E-04	0.211	0.760	0.106	8	4,253,570	*CSMD1*	NM_033225.3	intron	-11,313
rs36003087	6.65E-04	-0.211	0.760	0.198	5	31,089,141	*CDH6*	NM_004932	upstream	140,412
rs12915581	6.67E-04	0.210	0.760	0.089	15	66,795,369	*CORO2B*	NM_006091.1	intron	-665
rs8078216	6.67E-04	-0.211	0.760	0.267	17	56,192,719	*BCAS3*	NM_017679.2	intron	-13,297
rs17070023	6.69E-04	0.210	0.760	0.083	5	167,607,050	*WWC1*	NM_015238.1	flanking_5UTR	-44,620
rs11695952	6.69E-04	0.210	0.760	0.433	2	47,116,199	*TTC7A*	NM_020458	intron	0
rs12955986	6.70E-04	-0.213	0.760	0.403	18	63,299,314	*C18orf4*	NM_032160.2	flanking_3UTR	-25,485
rs8109218	6.70E-04	0.211	0.760	0.318	19	16,477,420	*FLJ21742*	NM_032207.1	intron	-1,092
rs4961258	6.70E-04	0.210	0.760	0.257	8	142,302,060	---	ENST0000002406	intron	0
rs313069	6.72E-04	-0.211	0.760	0.411	4	127,830,786	*PDZD6*	NM_015693.2	flanking_5UTR	-942,784
rs1231682	6.73E-04	-0.211	0.760	0.166	11	29,545,917	*KCNA4*	NM_002233.2	flanking_3UTR	-442,424
rs1362838	6.73E-04	-0.210	0.760	0.384	18	41,228,180	*SLC14A2*	NM_007163.2	flanking_5UTR	-220,584
rs16935567	6.74E-04	0.210	0.760	0.080	10	79,468,925	*RPS24*	NM_033022	intron	0
rs1880316	6.75E-04	-0.214	0.760	0.371	7	149,722,964	*MGC33584*	NM_173680.2	intron	-1,570
rs1992113	6.75E-04	-0.210	0.760	0.362	16	57,532,372	*GOT2*	NM_002080.2	flanking_5UTR	-206,625
rs817697	6.75E-04	-0.210	0.760	0.139	20	59,190,618	*CDH4*	NM_001794.2	flanking_5UTR	-70,334
rs7539255	6.76E-04	-0.211	0.760	0.080	1	8,055,939	*ERRFI1*	NM_018948.2	flanking_5UTR	-46,996
rs11802794	6.78E-04	0.210	0.760	0.246	1	195,638,873	*CRB1*	NM_012076.2	intron	-17,880
rs7455141	6.78E-04	0.211	0.760	0.084	7	102,612,968	*FLJ36166*	NM_182634.2	intron	-153
rs10922215	6.78E-04	0.210	0.760	0.246	1	195,630,571	*CRB1*	NM_201253	intron	0
rs1219396	6.79E-04	-0.210	0.760	0.252	11	121,265,814	*BRCC2*	NM_001001786.1	flanking_3UTR	-225,458
rs359440	6.79E-04	-0.210	0.760	0.435	5	173,231,068	*CPEB4*	NM_030627	upstream	17,727
rs8129616	6.79E-04	-0.215	0.760	0.242	21	29,441,956	*C21orf7*	NM_020152	intron	0
rs7517345	6.79E-04	0.210	0.760	0.150	1	199,523,156	*PKP1*	NM_001005337	intron	0
rs11054481	6.80E-04	-0.211	0.760	0.204	12	11,926,549	*ETV6*	NM_001987	intron	0
rs10034843	6.80E-04	-0.210	0.760	0.074	4	142,034,539	*RNF150*	NM_020724	intron	0
rs933528	6.81E-04	0.211	0.760	0.198	14	90,774,941	*GPR68*	NM_003485.3	intron	-3,665
rs7033608	6.83E-04	-0.214	0.760	0.463	9	136,513,419	*RXRA*	NM_002957.3	flanking_3UTR	-41,167
rs2353567	6.84E-04	0.210	0.760	0.361	14	94,548,576	*DICER1*	NM_177438.1	flanking_3UTR	-73,743
rs7147709	6.84E-04	0.210	0.760	0.361	14	94,550,677	*DICER1*	NM_177438.1	flanking_3UTR	-71,642
rs7628626	6.85E-04	-0.210	0.760	0.178	3	120,727,111	*CD80*	NM_005191.2	3UTR	[1281/175]
rs10953555	6.86E-04	-0.210	0.760	0.284	7	107,368,582	*LAMB1*	NM_002291	intron	0
rs1400064	6.87E-04	0.210	0.760	0.069	2	124,445,481	*CNTNAP5*	NM_138996.1	flanking_5UTR	-53,853
rs1867663	6.87E-04	0.210	0.760	0.295	5	142,039,088	*FGF1*	NM_000800	intron	0
rs6939403	6.87E-04	0.210	0.760	0.111	6	123,895,255	*TRDN*	NM_006073	intron	0
rs11162772	6.89E-04	0.212	0.760	0.500	1	79,888,726	*IFI44*	NM_006417.2	flanking_3UTR	-986,381
rs1504575	6.89E-04	-0.210	0.760	0.261	17	28,680,917	*ACCN1*	NM_183377.1	flanking_5UTR	-36,798
rs5922971	6.91E-04	-0.210	0.760	0.143	X	83,505,688	*CXorf43*	NM_144657.2	intron	-2,412
rs16939972	6.93E-04	0.210	0.760	0.431	12	108,053,180	---	ENST0000033843	intron	0
rs7214766	6.94E-04	-0.210	0.760	0.060	17	36,823,444	*KRTHA7*	NM_003770.4	flanking_3UTR	-6,891
rs154463	6.95E-04	0.210	0.760	0.237	5	123,782,537	*ZNF608*	NM_020747.1	flanking_3UTR	-217,972

rs16874544	6.95E-04	-0.210	0.760	0.118	6	85,184,648	*C6orf84*	NM_014895.1	flanking_5UTR	-190,615
rs7159075	6.95E-04	-0.211	0.760	0.480	14	97,244,232	*VRK1*	NM_003384.2	flanking_3UTR	-826,528
rs1317472	6.96E-04	-0.210	0.760	0.467	8	105,478,652	*DPYS*	NM_001385.1	intron	-4,257
rs17116594	6.96E-04	0.212	0.760	0.183	1	97,473,991	*DPYD*	NM_000110.2	intron	-853
rs12651293	6.99E-04	-0.211	0.760	0.357	4	188,714,276	*ZFP42*	NM_174900.2	flanking_5UTR	-439,643
rs6438535	6.99E-04	-0.210	0.760	0.178	3	120,701,404	*C3orf1*	NM_016589.3	intron	-828
rs9478395	6.99E-04	-0.210	0.760	0.098	6	153,545,079	*RGS17*	NM_012419.4	flanking_5UTR	-50,997
rs1368319	7.01E-04	-0.210	0.760	0.496	5	155,472,889	*SGCD*	NM_000337.4	flanking_5UTR	-213,456
rs1464217	7.02E-04	-0.210	0.760	0.207	18	24,719,204	*CDH2*	NM_001792.2	flanking_5UTR	-708,015
rs3935039	7.03E-04	0.210	0.760	0.167	18	43,845,323	*SMAD2*	NM_001003652.1	flanking_5UTR	-134,102
rs1379387	7.03E-04	0.210	0.760	0.208	2	81,295,549	*LRRTM1*	NM_178839	upstream	910,520
rs9324542	7.04E-04	0.213	0.760	0.251	8	142,297,036	---	ENST0000002406	intron	0
rs10793139	7.05E-04	-0.210	0.760	0.333	11	75,542,845	*UVRAG*	NM_003369.2	flanking_3UTR	-11,503
rs9501836	7.05E-04	0.210	0.760	0.190	6	2,359,212	---	ENST0000036637	downstream	2,349
rs6511680	7.06E-04	-0.210	0.760	0.447	19	24,017,416	*ZNF254*	NM_004876.1	flanking_5UTR	-44,400
rs7526011	7.06E-04	-0.210	0.760	0.453	1	231,545,687	*KIAA1804*	NM_032435	intron	0
rs11785907	7.06E-04	-0.211	0.760	0.141	8	13,580,393	*SGCZ*	NM_139167	downstream	411,351
rs10501215	7.07E-04	0.210	0.760	0.168	11	39,567,741	*LRRC4C*	NM_020929.1	flanking_3UTR	-524,588
rs6905952	7.07E-04	-0.210	0.760	0.120	6	134,440,751	*SLC2A12*	NM_145176.1	flanking_5UTR	-25,307
rs3814477	7.08E-04	0.209	0.760	0.399	7	1,535,944	*MAFK*	NM_002360.2	flanking_5UTR	-950
rs10055809	7.08E-04	0.210	0.760	0.082	5	143,746,439	*KCTD16*	NM_020768	intron	0
rs3786673	7.10E-04	0.210	0.760	0.142	19	55,194,724	*VRK3*	NM_001025778.1	intron	-1,135
rs17273407	7.10E-04	-0.213	0.760	0.233	19	61,688,407	---	ENST0000029999	intron	0
rs4622609	7.10E-04	-0.210	0.760	0.335	18	41,252,974	*SETBP1*	NM_015559	downstream	354,203
rs3809241	7.11E-04	0.210	0.760	0.133	12	5,926,711	*TMEM16B*	NM_020373.1	flanking_5UTR	-1,052
rs4635459	7.11E-04	-0.210	0.760	0.193	19	23,973,662	*ZNF254*	NM_004876.1	flanking_5UTR	-88,154
rs136	7.12E-04	0.209	0.760	0.292	7	24,902,623	*OSBPL3*	NM_145324.1	intron	-3,858
rs9839168	7.13E-04	0.209	0.760	0.167	3	133,023,719	*CPNE4*	NM_130808.1	intron	-83,079
rs13071252	7.14E-04	-0.209	0.760	0.418	3	22,569,059	*UBE2E2*	NM_152653.1	flanking_5UTR	-650,753
rs3796105	7.14E-04	-0.209	0.760	0.205	2	159,733,885	*TANC1*	NM_033394.1	intron	-122
rs6925549	7.15E-04	0.209	0.760	0.350	6	62,617,687	*KHDRBS2*	NM_152688	intron	0
rs1505792	7.15E-04	0.209	0.760	0.234	4	85,003,689	---	ENST0000036323	upstream	370,145
rs6597883	7.16E-04	-0.210	0.760	0.198	10	126,827,934	*CTBP2*	NM_001329.1	intron	-10,944
rs7564374	7.16E-04	-0.210	0.760	0.462	2	236,181,219	*CENTG2*	NM_014914.2	intron	-101,343
rs7942850	7.16E-04	0.210	0.760	0.287	11	1,058,900	*AP2A2*	NM_012305.2	flanking_3UTR	-56,660
rs12485178	7.16E-04	0.210	0.760	0.475	22	33,997,486	*HMG2L1*	NM_001003681	intron	0
rs7754879	7.17E-04	-0.209	0.760	0.495	6	108,101,814	*FLJ10159*	NM_018013.2	flanking_3UTR	-13,747
rs11812741	7.18E-04	-0.209	0.760	0.237	10	116,179,920	*ABLIM1*	NM_001003408.1	flanking_3UTR	-939
rs4237026	7.18E-04	-0.209	0.760	0.491	8	59,334,498	*C8orf72*	NM_147189.1	flanking_3UTR	-110,601
rs6428282	7.18E-04	-0.209	0.760	0.469	1	193,533,695	*KCNT2*	NM_198503.2	flanking_3UTR	-927,841
rs1872236	7.18E-04	0.210	0.760	0.375	17	6,104,618	*AIPL1*	NM_001033055	downstream	163,166
rs10505873	7.19E-04	-0.209	0.760	0.418	12	21,619,612	*GYS2*	NM_021957.2	intron	-455
rs9690914	7.20E-04	0.210	0.760	0.198	7	108,284,715	*DNAJB9*	NM_012328	downstream	282,185
rs1918662	7.21E-04	0.209	0.760	0.083	15	44,881,142	*SEMA6D*	NM_153619.1	flanking_5UTR	-916,836
rs3509	7.21E-04	0.211	0.760	0.395	1	77,798,139	*AK5*	NM_012093.2	3UTR	[1195/103]
rs35824	7.21E-04	-0.209	0.760	0.083	5	122,626,041	*FLJ36090*	NM_153223.1	flanking_3UTR	-83,493

rs950528	7.23E-04	0.210	0.760	0.400	3	113,334,507	*GCET2*	NM_001008756	intron	0
rs4590012	7.23E-04	-0.209	0.760	0.062	4	84,380,546	*COQ2*	NM_015697	downstream	23,457
rs10230291	7.24E-04	-0.209	0.760	0.203	7	103,379,058	*RELN*	NM_005045.2	intron	-34,190
rs1061627	7.24E-04	-0.209	0.760	0.290	12	21,545,674	*RECQL*	NM_032941.1	5UTR	[60/122]
rs12459711	7.24E-04	0.209	0.760	0.366	19	16,475,665	*FLJ21742*	NM_032207.1	intron	-474
rs7115326	7.24E-04	-0.209	0.760	0.359	11	58,034,925	*OR5B21*	NM_001005218.1	flanking_5UTR	-2,771
rs728116	7.24E-04	0.209	0.760	0.366	19	16,472,978	*FLJ21742*	NM_032207.1	coding	[374/384]
rs10912870	7.25E-04	-0.211	0.760	0.414	1	173,254,639	*MRPS14*	NM_022100.1	intron	-304
rs6583204	7.26E-04	-0.212	0.760	0.271	3	198,564,223	*DLG1*	NM_004087	upstream	54,379
rs8104657	7.28E-04	-0.209	0.760	0.444	19	24,008,904	*ZNF254*	NM_004876.1	flanking_5UTR	-52,912
rs2064878	7.28E-04	0.211	0.760	0.244	6	116,966,486	*FAM26D*	NM_153036	intron	0
rs4462524	7.29E-04	-0.209	0.760	0.234	14	28,443,034	*FOXG1B*	NM_005249.3	flanking_3UTR	-134,412
rs1529849	7.29E-04	-0.209	0.760	0.399	15	31,630,628	*RYR3*	NM_001036	intron	0
rs846987	7.29E-04	-0.209	0.760	0.263	6	108,098,586	*SOBP*	NM_018013	downstream	10,519
rs1402499	7.30E-04	-0.209	0.760	0.234	4	28,537,139	*FLJ45721*	NM_207490.1	flanking_5UTR	-1,708,015
rs10149128	7.31E-04	0.209	0.760	0.465	14	97,247,326	*VRK1*	NM_003384.2	flanking_3UTR	-829,622
rs3737484	7.31E-04	-0.209	0.760	0.279	4	101,004,003	*DAPP1*	NM_014395.1	coding	[53/32]
rs6993652	7.31E-04	-0.210	0.760	0.117	8	52,154,155	*PXDNL*	NM_144651	downstream	240,543
rs2223307	7.33E-04	-0.209	0.760	0.487	1	185,111,549	*PLA2G4A*	NM_024420.1	intron	-5,278
rs4883903	7.33E-04	-0.209	0.760	0.476	13	71,508,262	---	ENST0000036241	upstream	550,557
rs10418417	7.34E-04	-0.209	0.760	0.185	19	23,872,647	*LOC388524*	NM_001005472.1	flanking_3UTR	-69,870
rs1202580	7.34E-04	0.209	0.760	0.495	1	229,067,414	*C1orf198*	NM_032800	intron	0
rs4318628	7.35E-04	-0.210	0.760	0.381	4	151,821,568	*LRBA*	NM_006726.1	intron	-2,585
rs13122568	7.36E-04	0.209	0.760	0.170	4	128,954,925	*HSPA4L*	NM_014278.2	intron	-2,684
rs74111	7.36E-04	-0.209	0.760	0.295	6	15,894,581	*DTNBP1*	NM_183040.1	flanking_5UTR	-123,331
rs3095505	7.36E-04	-0.210	0.760	0.137	17	52,552,913	*AKAP1*	NM_003488	3UTR	0
rs6721270	7.37E-04	-0.209	0.760	0.254	2	10,438,577	*HPCAL1*	NM_002149.2	intron	-15,835
rs11784552	7.39E-04	0.209	0.760	0.120	8	9,262,317	*TNKS*	NM_003747.1	flanking_5UTR	-188,538
rs7604334	7.39E-04	-0.209	0.760	0.143	2	48,685,937	*STON1*	NM_006873.2	flanking_3UTR	-6,388
rs2831980	7.39E-04	-0.209	0.760	0.275	21	29,014,976	*N6AMT1*	NM_182749	downstream	151,424
rs8061376	7.41E-04	0.209	0.760	0.245	16	15,661,969	*NDE1*	NM_017668.1	intron	-4,125
rs10509737	7.41E-04	0.209	0.760	0.096	10	101,253,118	*NKX2-3*	NM_145285	upstream	29,582
rs8041909	7.41E-04	-0.210	0.760	0.068	15	61,440,532	*CA12*	NM_001218	intron	0
rs1394799	7.42E-04	0.209	0.760	0.143	2	212,939,124	*ERBB4*	NM_005235.1	intron	-172,294
rs865608	7.42E-04	0.209	0.760	0.502	20	50,696,776	*ZNF218*	NM_173485.2	flanking_5UTR	-325,577
rs12146687	7.43E-04	0.209	0.760	0.082	11	127,838,425	*ETS1*	NM_005238.2	intron	-189
rs837134	7.43E-04	0.212	0.760	0.142	15	35,003,147	*MEIS2*	NM_170677	intron	0
rs179805	7.44E-04	0.209	0.760	0.108	5	122,541,713	---	ENST0000026136	intron	0
rs9323049	7.44E-04	-0.209	0.760	0.382	14	41,022,910	*FBXO33*	NM_203301	upstream	2,051,539
rs12884090	7.44E-04	-0.209	0.760	0.382	14	41,023,512	*FBXO33*	NM_203301	upstream	2,052,141
rs10067053	7.45E-04	-0.209	0.760	0.385	5	99,169,537	*UNQ1912*	NM_198507.1	flanking_5UTR	-729,486
rs28634276	7.45E-04	-0.209	0.760	0.451	1	109,412,849	*TAF13*	NM_005645.3	intron	-2,472
rs784136	7.45E-04	0.209	0.760	0.149	6	117,060,879	*RSHL3*	NM_001010892.1	flanking_3UTR	-45
rs4674958	7.45E-04	-0.209	0.760	0.250	2	225,553,733	*DOCK10*	NM_014689	intron	0
rs7566475	7.45E-04	-0.209	0.760	0.250	2	225,555,912	*DOCK10*	NM_014689	intron	0
rs9967872	7.45E-04	-0.209	0.760	0.250	2	225,553,380	*DOCK10*	NM_014689	intron	0

rs7632529	7.45E-04	-0.212	0.760	0.050	3	192,698,805	---	ENST0000038202	downstream	36,866
rs11655927	7.47E-04	-0.209	0.760	0.279	17	56,183,184	*BCAS3*	NM_017679.2	intron	-3,762
rs16967789	7.48E-04	-0.209	0.760	0.127	17	70,840,584	*GRB2*	NM_203506.1	intron	-111
rs9643503	7.48E-04	-0.209	0.760	0.491	8	59,337,130	*FAM110B*	NM_147189	downstream	114,318
rs7314453	7.49E-04	-0.209	0.760	0.085	12	118,016,813	*KIAA1853*	NM_194286.2	intron	-7,611
rs16914398	7.50E-04	-0.209	0.760	0.078	8	92,692,156	*SLC26A7*	NM_052832.2	flanking_3UTR	-212,602
rs4348408	7.50E-04	-0.209	0.760	0.413	7	107,296,509	*DLD*	NM_000108.2	flanking_5UTR	-22,338
rs4911305	7.50E-04	-0.209	0.760	0.178	20	31,276,795	*C20orf71*	NM_178466.2	intron	-81
rs16975962	7.51E-04	0.209	0.760	0.058	15	53,199,152	*C15orf15*	NM_016304.2	flanking_3UTR	-61,652
rs9872064	7.52E-04	-0.208	0.760	0.301	3	26,777,924	*LRRC3B*	NM_052953	downstream	50,658
rs1148645	7.53E-04	-0.208	0.760	0.130	4	152,044,416	*LRBA*	NM_006726.1	intron	-2,024
rs7870690	7.54E-04	0.211	0.760	0.191	9	15,208,947	*C9orf52*	NM_152574.1	intron	-4,700
rs7705950	7.54E-04	0.208	0.760	0.058	5	122,501,861	---	ENST0000026136	intron	0
rs1332841	7.55E-04	-0.208	0.760	0.176	6	12,983,040	*PHACTR1*	NM_030948.1	intron	-125,032
rs10269135	7.56E-04	-0.208	0.760	0.103	7	123,372,032	*SPAM1*	NM_003117.3	intron	-5,130
rs11692215	7.56E-04	-0.208	0.760	0.353	2	100,113,005	*AFF3*	NM_002285.2	intron	-12,373
rs9479998	7.56E-04	0.209	0.760	0.125	6	155,359,461	*TIAM2*	NM_012454	intron	0
rs11242465	7.57E-04	-0.208	0.760	0.384	5	138,790,204	*DNAJC18*	NM_152686.2	intron	-259
rs3135402	7.57E-04	0.211	0.760	0.290	6	33,132,632	*HLA-DPA1*	NM_033554.2	flanking_3UTR	-8,140
rs10200581	7.58E-04	0.209	0.760	0.104	2	33,714,797	*FAM98A*	NM_015475	upstream	36,927
rs17160491	7.59E-04	-0.208	0.760	0.299	19	8,418,940	*HNRPM*	NM_005968.2	intron	-2,945
rs1334814	7.60E-04	0.208	0.760	0.313	1	232,651,549	*TARBP1*	NM_005646	intron	0
rs1040463	7.61E-04	0.208	0.760	0.127	X	29,417,079	*IL1RAPL1*	NM_014271	intron	0
rs11676226	7.63E-04	-0.209	0.760	0.445	2	236,179,508	*CENTG2*	NM_014914.2	intron	-103,054
rs2190707	7.63E-04	0.208	0.760	0.382	17	66,884,459	*SOX9*	NM_000346.2	flanking_5UTR	-744,297
rs10794839	7.64E-04	0.208	0.760	0.332	10	2,076,923	*ADARB2*	NM_018702.1	flanking_5UTR	-307,253
rs2822964	7.64E-04	-0.208	0.760	0.467	21	15,151,796	*NRIP1*	NM_003489.2	flanking_3UTR	-103,631
rs830994	7.64E-04	0.208	0.760	0.263	2	169,837,774	*LRP2*	NM_004525.1	coding	[91/49]
rs11725452	7.65E-04	0.214	0.760	0.378	4	164,155,635	*LOC92345*	NM_138386	downstream	113,640
rs2246650	7.66E-04	-0.209	0.760	0.467	8	105,478,004	*DPYS*	NM_001385.1	intron	-3,609
rs5771204	7.66E-04	0.209	0.760	0.409	22	48,956,603	*PANX2*	NM_052839.2	intron	-892
rs6080286	7.66E-04	0.208	0.760	0.111	20	16,458,024	*C20orf23*	NM_024704.3	intron	-939
rs8078518	7.66E-04	0.209	0.760	0.371	17	6,115,984	*KIAA0523*	NM_015253.1	flanking_3UTR	-147,513
rs4547414	7.67E-04	-0.208	0.760	0.377	18	26,638,723	*DSC3*	NM_001941.2	flanking_3UTR	-186,306
rs6714525	7.67E-04	0.208	0.760	0.045	2	2,678,847	*MYT1L*	NM_015025	upstream	364,795
rs17078934	7.68E-04	-0.208	0.760	0.150	13	23,026,199	*TNFRSF19*	NM_148957.2	flanking_5UTR	-16,524
rs7792702	7.68E-04	-0.209	0.760	0.229	7	151,921,469	*XRCC2*	NM_005431.1	flanking_3UTR	-53,047
rs1860116	7.68E-04	0.208	0.760	0.152	7	150,628,246	*SMARCD3*	NM_003078	upstream	24,294
rs4856502	7.69E-04	0.208	0.760	0.105	3	84,440,325	*CADM2*	NM_153184	upstream	1,417,997
rs1343635	7.70E-04	-0.208	0.760	0.351	1	58,203,723	*DAB1*	NM_021080.3	intron	-81,301
rs6512116	7.71E-04	0.208	0.760	0.365	19	16,474,476	*FLJ21742*	NM_032207.1	intron	-400
rs10089616	7.72E-04	0.208	0.760	0.129	8	1,719,262	*CLN8*	NM_018941.2	3UTR	[3121/600]
rs1866555	7.72E-04	-0.208	0.760	0.188	2	104,508,220	*POU3F3*	NM_006236.1	flanking_5UTR	-330,181
rs3770939	7.72E-04	-0.208	0.760	0.156	2	36,471,032	*CRIM1*	NM_016441.1	intron	-6,229
rs4751158	7.72E-04	-0.208	0.760	0.451	10	131,700,143	*EBF3*	NM_001005463	upstream	48,048
rs12277079	7.73E-04	-0.208	0.760	0.192	11	8,222,228	*LMO1*	NM_002315.1	intron	-13,601

rs17473108	7.73E-04	-0.208	0.760	0.145	4	164,683,228	*1-Mar*	NM_017923.2	intron	-2,970
rs2241439	7.75E-04	-0.208	0.760	0.257	7	132,131,435	*CHCHD3*	NM_017812.1	intron	-308
rs9955009	7.76E-04	-0.208	0.760	0.482	18	50,866,173	*CCDC68*	NM_025214.1	flanking_5UTR	-88,538
rs8021868	7.76E-04	-0.209	0.760	0.172	14	56,861,066	*NAT12*	NM_001011713	upstream	65,958
rs8082807	7.78E-04	0.208	0.760	0.489	18	49,264,900	*DCC*	NM_005215.1	intron	-2,265
rs9435736	7.78E-04	0.208	0.760	0.182	1	17,196,036	*ATP13A2*	NM_022089.1	intron	-66
rs6954725	7.78E-04	-0.208	0.760	0.170	7	156,234,982	*LMBR1*	NM_022458	intron	0
rs16958589	7.79E-04	0.209	0.760	0.438	17	9,531,134	*WDRPUH*	NM_145054.2	flanking_3UTR	-43,707
rs6843038	7.82E-04	0.208	0.760	0.365	4	182,162,059	*MGC45800*	NM_178838.3	flanking_3UTR	-1,137,738
rs16947830	7.83E-04	-0.208	0.760	0.069	15	91,678,261	*UNQ9370*	NM_207447.1	flanking_3UTR	-125,980
rs2955279	7.83E-04	-0.208	0.760	0.473	2	43,965,926	*LRPPRC*	NM_133259.2	flanking_3UTR	-2,465
rs11703673	7.83E-04	0.211	0.760	0.253	22	48,994,554	*RP3-402G11.5*	NM_031454	intron	0
rs4731018	7.84E-04	0.208	0.760	0.074	7	120,910,659	*FAM3C*	NM_014888.1	flanking_5UTR	-87,082
rs7251978	7.84E-04	0.209	0.760	0.365	19	16,473,365	*FLJ21742*	NM_032207.1	intron	-3
rs11079337	7.85E-04	-0.208	0.760	0.338	17	53,517,495	*DYNLL2*	NM_080677.1	intron	-1,452
rs11601054	7.85E-04	0.208	0.760	0.167	11	112,841,382	*DRD2*	NM_000795	intron	0
rs11802191	7.86E-04	0.208	0.760	0.174	1	101,607,311	*EDG1*	NM_001400	downstream	127,649
rs475323	7.87E-04	0.209	0.760	0.130	7	6,057,126	*EIF2AK1*	NM_014413.2	intron	-924
rs6660804	7.89E-04	-0.208	0.760	0.292	1	171,705,022	*PRDX6*	NM_004905.2	flanking_5UTR	-8,087
rs12317807	7.89E-04	0.209	0.760	0.082	12	128,961,863	*TMEM132D*	NM_133448	upstream	8,026
rs231888	7.90E-04	0.208	0.760	0.475	11	2,695,686	*KCNQ1*	NM_181798.1	intron	-50,964
rs588469	7.90E-04	0.208	0.760	0.076	5	41,737,375	*OXCT1*	NM_000436.2	flanking_3UTR	-28,549
rs600645	7.90E-04	0.208	0.760	0.076	5	41,736,994	*OXCT1*	NM_000436.2	flanking_3UTR	-28,930
rs685396	7.90E-04	0.208	0.760	0.076	5	41,724,544	*OXCT1*	NM_000436.2	flanking_3UTR	-41,380
rs10840403	7.90E-04	-0.208	0.760	0.089	11	10,381,515	*AMPD3*	NM_000480	upstream	47,285
rs3732051	7.91E-04	-0.208	0.760	0.125	2	51,280,678	*NRXN1*	NM_004801.2	flanking_5UTR	-171,571
rs6860492	7.91E-04	-0.208	0.760	0.478	5	74,328,239	*GCNT4*	NM_016591	downstream	32,018
rs6949082	7.92E-04	-0.208	0.760	0.104	7	123,302,132	*HYAL4*	NM_012269.1	coding	[81/8]
rs1504232	7.92E-04	-0.208	0.760	0.134	11	38,695,259	*C11orf74*	NM_138787	downstream	2,057,862
rs5965863	7.92E-04	0.208	0.760	0.060	X	144,408,021	*SLITRK2*	NM_032539	upstream	299,360
rs2388214	7.94E-04	0.208	0.760	0.121	11	89,340,497	*TRIM49*	NM_020358.2	flanking_5UTR	-159,106
rs17050312	7.94E-04	-0.208	0.760	0.127	4	120,455,134	*LOC401152*	NM_001001701	upstream	10,086
rs17696757	7.95E-04	-0.208	0.760	0.094	18	36,990,740	*PIK3C3*	NM_002647.2	flanking_5UTR	-798,457
rs4741887	7.95E-04	-0.210	0.760	0.433	9	4,069,470	*GLIS3*	NM_152629.2	intron	-38,298
rs1148652	7.95E-04	-0.208	0.760	0.134	4	152,021,388	*LRBA*	NM_006726	intron	0
rs11564122	7.97E-04	0.208	0.760	0.058	12	38,834,017	*LRRK2*	NM_198578	upstream	71,184
rs11564116	7.97E-04	0.208	0.760	0.058	12	38,856,044	*SLC2A13*	NM_052885	upstream	70,116
rs1866131	7.98E-04	-0.209	0.760	0.064	2	177,418,780	*HNRPA3*	NM_194247.1	flanking_5UTR	-366,994
rs7140726	7.98E-04	-0.208	0.760	0.380	14	41,029,534	*FBXO33*	NM_203301	upstream	2,058,163
rs17126584	7.98E-04	-0.208	0.760	0.404	14	72,824,499	*NUMB*	NM_001005743	intron	0
rs4893555	8.00E-04	-0.207	0.760	0.303	X	28,782,000	*IL1RAPL1*	NM_014271	intron	0
rs4893599	8.00E-04	-0.207	0.760	0.303	X	28,782,529	*IL1RAPL1*	NM_014271	intron	0
rs7679888	8.01E-04	-0.209	0.760	0.086	4	151,363,620	*DCAMKL2*	NM_152619.1	intron	-1,233
rs255957	8.01E-04	-0.207	0.760	0.100	5	94,666,129	*MCTP1*	NM_001002796	upstream	20,094
rs752787	8.02E-04	0.207	0.760	0.232	2	2,588,720	*MYT1L*	NM_015025	upstream	274,668
rs11985119	8.02E-04	-0.207	0.760	0.357	8	23,668,083	---	ENST0000032501	upstream	48,027

rs11099780	8.03E-04	-0.208	0.760	0.365	4	152,108,059	*LRBA*	NM_006726	intron	0
rs12892792	8.04E-04	-0.208	0.760	0.305	14	24,004,750	*C14orf124*	NM_020195.1	flanking_5UTR	-22,928
rs17133649	8.04E-04	0.207	0.760	0.112	5	111,061,321	---	ENST00000038493	upstream	141,747
rs10420981	8.06E-04	-0.208	0.760	0.250	19	23,904,617	*LOC388524*	NM_001005472.1	flanking_3UTR	-101,840
rs7050282	8.06E-04	-0.208	0.760	0.344	X	144,810,567	*CXorf1*	NM_004709	downstream	91,507
rs10807735	8.07E-04	-0.212	0.760	0.472	7	70,718,334	*WBSCR17*	NM_022479	intron	0
rs8033246	8.07E-04	0.207	0.760	0.134	15	66,763,936	*CORO2B*	NM_006091	intron	0
rs900102	8.08E-04	-0.209	0.760	0.347	8	32,239,730	*NRG1*	NM_013958.1	flanking_5UTR	-285,565
rs13120637	8.08E-04	0.207	0.760	0.266	4	171,654,807	*AADAT*	NM_016228	upstream	406,860
rs11155474	8.09E-04	-0.207	0.760	0.348	6	146,896,790	*RAB32*	NM_006834.2	flanking_5UTR	-9,731
rs2346689	8.09E-04	-0.207	0.760	0.357	17	28,650,968	*ACCN1*	NM_183377.1	flanking_5UTR	-6,849
rs899828	8.09E-04	-0.208	0.760	0.100	18	30,566,650	*DTNA*	NM_001390.3	intron	-3,142
rs10141817	8.10E-04	-0.207	0.760	0.391	14	43,515,660	*C14orf155*	NM_032135.2	flanking_3UTR	-527,634
rs11265618	8.10E-04	0.208	0.760	0.207	1	152,696,716	*IL6R*	NM_000565.2	intron	-3,035
rs4811685	8.10E-04	-0.207	0.760	0.283	20	54,273,201	*MC3R*	NM_019888.2	flanking_3UTR	-14,923
rs11069734	8.11E-04	0.207	0.760	0.446	13	107,800,359	*TNFSF13B*	NM_006573.3	flanking_3UTR	-42,993
rs2188909	8.11E-04	0.208	0.760	0.413	2	130,052,695	---	ENST00000038569	downstream	151,129
rs1811661	8.11E-04	-0.212	0.760	0.093	11	82,112,525	---	ENST00000031309	downstream	7,774
rs4593456	8.12E-04	0.208	0.760	0.084	7	102,619,011	*FLJ36166*	NM_182634.2	intron	-5,791
rs16841450	8.12E-04	-0.207	0.760	0.230	2	157,831,816	*GALNT5*	NM_014568	intron	0
rs6982145	8.12E-04	0.207	0.760	0.074	8	50,104,256	*C8orf22*	NM_001007176	upstream	43,210
rs3776084	8.13E-04	0.208	0.760	0.409	5	149,548,286	*SLC6A7*	NM_014228.2	flanking_5UTR	-1,427
rs4632856	8.14E-04	0.208	0.760	0.111	6	79,269,676	*IRAK1BP1*	NM_001010844.1	flanking_5UTR	-364,232
rs4684510	8.15E-04	-0.207	0.760	0.141	3	5,856,625	*EDEM1*	NM_014674.1	flanking_3UTR	-619,983
rs10033127	8.15E-04	-0.208	0.760	0.115	4	139,819,531	---	ENST00000035756	upstream	55,327
rs4774702	8.17E-04	0.208	0.760	0.491	15	52,754,901	*C15orf15*	NM_016304.2	flanking_3UTR	-505,903
rs1459302	8.18E-04	0.210	0.760	0.169	4	131,616,142	*LOC132321*	NM_173487.1	flanking_3UTR	-1,362,849
rs4471801	8.18E-04	-0.208	0.760	0.403	18	63,271,192	*C18orf4*	NM_032160.2	flanking_3UTR	-53,607
rs874881	8.18E-04	0.207	0.760	0.428	1	17,533,086	*PADI4*	NM_012387.1	coding	[61/5]
rs17174296	8.19E-04	-0.207	0.760	0.049	X	153,194,171	*TKTL1*	NM_012253.2	coding	[46/147]
rs11640979	8.20E-04	-0.207	0.760	0.322	16	85,383,464	*FOXL1*	NM_005250.1	flanking_3UTR	-212,596
rs16983858	8.20E-04	0.207	0.760	0.060	20	58,385,068	*LOC284757*	NM_001004305	downstream	52,076
rs11931954	8.21E-04	0.208	0.760	0.120	4	170,701,015	*NEK1*	NM_012224.1	intron	-5,378
rs12625514	8.21E-04	-0.208	0.760	0.219	20	15,972,095	*MACROD2*	NM_001033086	intron	0
rs4867917	8.21E-04	0.209	0.760	0.482	5	169,470,766	*FOXI1*	NM_012188	downstream	1,475
rs12927800	8.21E-04	-0.207	0.760	0.364	16	57,531,468	---	ENST00000038767	downstream	169,243
rs1958299	8.22E-04	-0.207	0.760	0.096	14	23,376,204	---	ENST00000038671	downstream	45,022
rs6730684	8.24E-04	-0.208	0.760	0.425	2	44,085,422	*LRPPRC*	NM_133259	upstream	8,832
rs881176	8.24E-04	-0.209	0.760	0.264	7	107,360,490	*LAMB1*	NM_002291	intron	0
rs9542806	8.25E-04	-0.207	0.760	0.476	13	71,481,059	---	ENST00000036241	upstream	577,760
rs12699060	8.26E-04	-0.209	0.760	0.491	7	70,731,193	*WBSCR17*	NM_022479	intron	0
rs2761355	8.27E-04	0.208	0.760	0.347	14	89,189,379	*C14orf143*	NM_145231.1	flanking_3UTR	-141,715
rs6719811	8.27E-04	0.207	0.760	0.069	2	212,902,650	*ERBB4*	NM_005235.1	intron	-204,777
rs2561018	8.27E-04	0.207	0.760	0.174	19	57,641,380	---	ENST00000030108	intron	0
rs3027575	8.29E-04	0.207	0.760	0.074	X	100,553,492	*HNRPH2*	NM_001032393.1	intron	-88
rs2830991	8.29E-04	0.207	0.760	0.255	21	27,808,439	---	ENST00000032498	upstream	499,133

rs926543	8.30E-04	0.207	0.760	0.078	22	35,319,273	*CACNG2*	NM_006078.2	intron	-5,732
rs7962117	8.31E-04	-0.208	0.760	0.318	12	96,443,183	*NCRMS*	XR_000219.2	intron	-5,381
rs2423395	8.32E-04	0.207	0.760	0.360	20	9,593,420	*PAK7*	NM_020341.2	intron	-20,433
rs1041620	8.32E-04	-0.207	0.760	0.362	13	77,307,535	*EDNRB*	NM_003991	downstream	60,082
rs2202722	8.33E-04	0.207	0.760	0.386	4	171,628,169	*AADAT*	NM_182662.1	flanking_5UTR	-380,222
rs2395932	8.33E-04	-0.207	0.760	0.296	7	107,282,381	*DLD*	NM_000108.2	flanking_5UTR	-36,466
rs2135900	8.33E-04	0.207	0.760	0.386	4	171,625,085	*AADAT*	NM_016228	upstream	377,138
rs11071512	8.34E-04	0.208	0.760	0.147	15	30,132,431	*CHRFAM7A*	NM_148911	intron	0
rs10510398	8.35E-04	0.208	0.760	0.102	3	9,349,170	*THUMPD3*	NM_015453.1	flanking_5UTR	-30,566
rs7290877	8.35E-04	-0.207	0.760	0.069	22	48,070,422	*FLJ44385*	NM_207478.1	flanking_5UTR	-257,911
rs12403322	8.36E-04	0.207	0.760	0.212	1	83,474,316	*TTLL7*	NM_024686.3	flanking_3UTR	-633,715
rs16845288	8.36E-04	-0.207	0.760	0.109	4	71,733,369	*ENAM*	NM_031889.1	flanking_3UTR	-1,969
rs4586406	8.36E-04	0.207	0.760	0.196	15	89,722,238	*SV2B*	NM_014848.2	flanking_3UTR	-82,721
rs10819657	8.36E-04	-0.207	0.760	0.409	9	101,168,330	*NR4A3*	NM_173200	upstream	460,500
rs6081555	8.36E-04	-0.207	0.760	0.272	20	19,193,723	*SLC24A3*	NM_020689	intron	0
rs320629	8.37E-04	0.207	0.760	0.134	17	28,678,796	*ACCN1*	NM_001094	intron	0
rs9891803	8.38E-04	0.207	0.760	0.349	17	28,633,928	*ACCN1*	NM_183377	intron	0
rs12699376	8.38E-04	-0.207	0.760	0.232	7	12,465,360	---	ENST0000036633	upstream	31,979
rs2836574	8.39E-04	-0.207	0.760	0.101	21	38,913,763	*ERG*	NM_004449.3	intron	-35,024
rs6902875	8.39E-04	-0.207	0.760	0.471	6	132,658,765	*MOXD1*	NM_015529.1	flanking_3UTR	-136
rs7157405	8.39E-04	-0.207	0.760	0.337	14	70,359,878	*MAP3K9*	NM_033141.2	flanking_5UTR	-14,237
rs4513770	8.40E-04	0.207	0.760	0.330	6	62,608,387	*KHDRBS2*	NM_152688	intron	0
rs325183	8.41E-04	-0.207	0.760	0.091	11	38,659,243	*LRRC4C*	NM_020929.1	flanking_3UTR	-1,433,086
rs2608543	8.41E-04	0.207	0.760	0.172	19	57,641,492	---	ENST0000030108	intron	0
rs903389	8.41E-04	-0.207	0.760	0.364	5	35,863,422	*IL7R*	NM_002185	upstream	29,326
rs6534494	8.42E-04	0.207	0.760	0.093	4	126,724,051	*FAT4*	NM_024582.2	flanking_3UTR	-91,678
rs7168357	8.42E-04	0.207	0.760	0.199	15	94,819,479	*NR2F2*	NM_021005.2	flanking_3UTR	-136,431
rs11927211	8.42E-04	0.207	0.760	0.136	3	155,974,672	*GPR149*	NM_001038705	upstream	344,574
rs10935990	8.42E-04	0.207	0.760	0.136	3	155,976,075	*MME*	NM_007287	upstream	308,576
rs2759662	8.44E-04	0.207	0.760	0.384	1	195,628,194	*CRB1*	NM_201253	intron	0
rs157563	8.45E-04	0.207	0.760	0.153	5	89,418,259	*CETN3*	NM_004365.2	flanking_3UTR	-307,025
rs2064265	8.45E-04	0.207	0.760	0.223	X	33,179,680	---	ENST0000028844	intron	0
rs890000	8.46E-04	0.207	0.760	0.281	8	4,058,846	---	ENST0000038721	downstream	513,345
rs2753977	8.47E-04	0.207	0.760	0.150	6	66,176,184	*EGFL11*	NM_198283.1	intron	-4,203
rs583523	8.47E-04	0.210	0.760	0.457	18	2,864,156	*EMILIN2*	NM_032048.2	intron	-10,806
rs990748	8.47E-04	0.209	0.760	0.122	11	89,318,458	*TRIM49*	NM_020358.2	flanking_5UTR	-137,067
rs10496673	8.47E-04	-0.207	0.760	0.091	2	129,311,391	---	ENST0000036282	upstream	392,149
rs3803476	8.48E-04	0.206	0.760	0.368	15	97,074,093	*IGF1R*	NM_000875.2	intron	-5,234
rs907133	8.51E-04	0.207	0.760	0.111	8	28,750,468	*RC74*	NM_018250.1	intron	-605
rs11694213	8.51E-04	-0.206	0.760	0.168	2	55,942,171	*EFEMP1*	NM_001039348	downstream	4,430
rs2429369	8.53E-04	0.206	0.760	0.342	17	53,856,419	*RNF43*	NM_017763.3	flanking_5UTR	-6,526
rs2279269	8.55E-04	-0.207	0.760	0.478	18	63,330,809	*C18orf4*	NM_032160.2	coding	[1622/2046]
rs1003634	8.55E-04	-0.207	0.760	0.416	7	24,063,026	---	ENST0000038757	upstream	161,748
rs220896	8.56E-04	-0.208	0.760	0.344	6	15,877,517	*MYLIP*	NM_013262	upstream	359,779
rs17045555	8.57E-04	-0.206	0.760	0.091	4	114,233,814	*ANK2*	NM_001148.3	intron	-43,397
rs3112229	8.57E-04	-0.210	0.760	0.248	2	95,202,401	*ZNF2*	NM_021088.2	intron	-3,259

rs882806	8.57E-04	-0.208	0.760	0.118	20	805,271	*ANGPT4*	NM_015985	intron	0
rs4329503	8.57E-04	0.206	0.760	0.495	1	235,702,187	*RYR2*	NM_001035	intron	0
rs7035608	8.58E-04	-0.207	0.760	0.135	9	2,054,507	*SMARCA2*	NM_003070.3	intron	-3,521
rs887918	8.58E-04	-0.206	0.760	0.203	4	31,388,233	*PCDH7*	NM_032457	downstream	634,664
rs1418926	8.58E-04	0.206	0.760	0.315	20	52,260,675	*PFDN4*	NM_002623	intron	0
rs13169373	8.59E-04	-0.208	0.760	0.238	5	166,277,666	*WWC1*	NM_015238.1	flanking_5UTR	-1,374,004
rs2407548	8.59E-04	-0.206	0.760	0.364	4	152,069,974	*LRBA*	NM_006726.1	intron	-307
rs3112996	8.59E-04	-0.207	0.760	0.271	2	95,176,099	*ZNF514*	NM_032788.1	flanking_3UTR	-1,028
rs7055613	8.59E-04	0.207	0.760	0.097	X	4,655,454	---	ENST0000038747	downstream	442,027
rs10001300	8.60E-04	-0.206	0.760	0.121	4	8,173,528	*ABLIM2*	NM_032432	intron	0
rs11799515	8.60E-04	0.208	0.760	0.245	1	195,639,060	*CRB1*	NM_201253	intron	0
rs1589127	8.61E-04	0.207	0.760	0.135	4	138,105,032	*PCDH18*	NM_019035.2	flanking_3UTR	-555,253
rs9698139	8.61E-04	0.208	0.760	0.476	X	122,799,241	*BIRC4*	NM_001167.2	flanking_5UTR	-22,488
rs12706565	8.62E-04	-0.206	0.760	0.197	7	123,397,313	*SPAM1*	NM_003117.3	intron	-175
rs13408032	8.62E-04	-0.206	0.760	0.283	2	242,139,331	*BOK*	NM_032515.3	flanking_5UTR	-7,534
rs5749104	8.62E-04	0.207	0.760	0.236	22	29,187,448	*SEC14L3*	NM_174975.3	coding	[151/18]
rs6532834	8.62E-04	-0.206	0.760	0.275	4	101,009,351	*DAPP1*	NM_014395.1	3UTR	[1044/983]
rs6743733	8.62E-04	-0.206	0.760	0.094	2	180,219,874	*ZNF533*	NM_152520	intron	0
rs9292828	8.63E-04	0.207	0.760	0.118	5	41,873,989	*OXCT1*	NM_000436	intron	0
rs1534965	8.65E-04	0.208	0.760	0.122	X	29,416,942	*IL1RAPL1*	NM_014271.2	intron	-89,596
rs2871344	8.65E-04	-0.206	0.760	0.370	2	100,205,862	*LONRF2*	NM_198461	downstream	60,574
rs1499424	8.67E-04	0.207	0.760	0.265	8	118,391,268	*SLC30A8*	NM_173851.2	flanking_3UTR	-133,134
rs4625270	8.67E-04	0.206	0.760	0.496	1	235,702,039	*RYR2*	NM_001035.1	intron	-2,929
rs738800	8.67E-04	-0.207	0.760	0.236	22	22,488,315	*SMARCB1*	NM_003073.3	intron	-642
rs4397648	8.67E-04	0.206	0.760	0.496	1	235,701,944	*RYR2*	NM_001035	intron	0
rs4329502	8.67E-04	0.206	0.760	0.496	1	235,701,926	*RYR2*	NM_001035	intron	0
rs2083350	8.67E-04	-0.207	0.760	0.223	2	104,532,188	---	ENST0000036285	downstream	16,314
rs10514459	8.68E-04	0.206	0.760	0.054	16	78,119,642	*MAF*	NM_005360.3	flanking_3UTR	-66,090
rs12217099	8.68E-04	0.206	0.760	0.092	1	160,222,733	*OLFML2B*	NM_015441	intron	0
rs13403315	8.69E-04	-0.206	0.760	0.185	2	157,643,349	*GALNT5*	NM_014568.1	flanking_5UTR	-179,237
rs887343	8.69E-04	0.206	0.760	0.364	5	149,529,016	*CDX1*	NM_001804.1	intron	-1,939
rs7557024	8.69E-04	0.206	0.760	0.239	2	81,314,530	*LRRTM1*	NM_178839	upstream	929,501
rs4852611	8.69E-04	0.208	0.760	0.236	2	81,278,948	*CTNNA2*	NM_004389	downstream	550,440
rs1430282	8.70E-04	0.206	0.760	0.116	2	83,270,194	*LOC388965*	NM_001013648.3	flanking_5UTR	-1,101,124
rs2568225	8.70E-04	-0.206	0.760	0.346	2	85,233,425	*TCF7L1*	NM_031283.1	intron	-18,341
rs10164675	8.71E-04	0.206	0.760	0.096	2	212,895,279	*ERBB4*	NM_005235.1	intron	-197,406
rs10235277	8.71E-04	0.206	0.760	0.279	7	6,002,771	*PMS2*	NM_000535.3	intron	-712
rs4313914	8.71E-04	-0.206	0.760	0.197	19	23,968,836	*ZNF254*	NM_004876.1	flanking_5UTR	-92,980
rs9297228	8.72E-04	-0.206	0.760	0.112	8	35,019,363	*UNC5D*	NM_080872.1	flanking_5UTR	-502,089
rs7292688	8.72E-04	-0.206	0.760	0.136	22	47,406,839	*FAM19A5*	NM_015381	intron	0
rs967076	8.72E-04	0.206	0.760	0.184	4	131,513,112	---	ENST0000034179	downstream	468,925
rs16871186	8.73E-04	0.206	0.760	0.082	6	11,379,978	---	ENST0000033924	intron	0
rs35600708	8.73E-04	-0.206	0.760	0.054	9	96,396,064	*FBP2*	NM_003837	upstream	168
rs11024586	8.73E-04	0.207	0.760	0.112	11	18,232,912	*SAA2*	NM_030754	upstream	6,154
rs134434	8.74E-04	0.208	0.760	0.437	22	33,921,035	*HMG2L1*	NM_005487.3	flanking_5UTR	-62,454
rs17058247	8.74E-04	-0.206	0.760	0.491	5	160,134,319	*PTTG1*	NM_004219.2	flanking_3UTR	-345,995

rs524361	8.74E-04	0.208	0.760	0.307	9	22,687,535	*ELAVL2*	NM_004432	downstream	994,274
rs2875342	8.74E-04	0.206	0.760	0.322	3	7,863,645	*GRM7*	NM_000844	downstream	160,531
rs4900070	8.75E-04	-0.207	0.760	0.089	14	90,660,741	*C14orf159*	NM_024952.4	intron	-9,423
rs2832067	8.75E-04	0.206	0.760	0.482	21	29,058,739	*N6AMT1*	NM_013240	downstream	107,661
rs584591	8.75E-04	-0.206	0.760	0.217	11	30,718,540	*MPPED2*	NM_001584	upstream	153,427
rs12031508	8.75E-04	-0.206	0.760	0.325	1	104,374,145	---	ENST0000038870	downstream	2,773,584
rs2461144	8.76E-04	-0.206	0.760	0.478	10	18,144,556	*MRC1*	NM_002438.1	intron	-6,035
rs8106506	8.77E-04	-0.206	0.760	0.138	19	62,378,023	*DUXA*	NM_001012729.1	flanking_5UTR	-7,355
rs10865195	8.77E-04	-0.206	0.760	0.406	2	44,043,111	*LRPPRC*	NM_133259	intron	0
rs4687747	8.77E-04	0.206	0.760	0.189	3	53,840,473	*CHDH*	NM_018397	intron	0
rs17069280	8.78E-04	-0.206	0.760	0.069	6	108,555,304	*OSTM1*	NM_014028	upstream	52,670
rs314272	8.79E-04	0.206	0.760	0.500	6	105,568,697	*LIN28B*	NM_001004317.2	intron	-12,169
rs4330101	8.79E-04	-0.206	0.760	0.145	2	223,525,267	*KCNE4*	NM_080671	upstream	99,904
rs873429	8.79E-04	0.208	0.760	0.065	3	25,040,133	---	ENST0000038726	upstream	3,191
rs7566527	8.79E-04	-0.206	0.760	0.346	2	100,178,402	*AFF3*	NM_001025108	upstream	89,925
rs10836343	8.80E-04	-0.206	0.760	0.156	11	35,201,463	*CD44*	NM_000610	intron	0
rs10732349	8.81E-04	0.206	0.760	0.172	9	27,715,305	*C9orf72*	NM_018325	upstream	151,829
rs12449358	8.81E-04	-0.207	0.760	0.267	17	28,671,154	*ACCN1*	NM_001094	intron	0
rs5770980	8.82E-04	0.207	0.760	0.279	22	49,233,086	*SBF1*	NM_002972.1	intron	-182
rs890220	8.82E-04	0.206	0.760	0.142	8	41,597,518	*AGPAT6*	NM_178819.2	intron	-51
rs1866769	8.83E-04	-0.206	0.760	0.264	11	133,779,886	*B3GAT1*	NM_018644.2	intron	-7,025
rs4396035	8.83E-04	0.206	0.760	0.361	X	138,387,235	*F9*	NM_000133.2	flanking_5UTR	-53,326
rs7608295	8.84E-04	-0.206	0.760	0.216	2	104,524,614	---	ENST0000036285	downstream	8,740
rs16889986	8.87E-04	-0.206	0.760	0.154	8	40,795,594	*ZMAT4*	NM_024645	intron	0
rs13388743	8.88E-04	0.206	0.760	0.210	2	188,281,489	*TFPI*	NM_006287.3	flanking_5UTR	-154,192
rs7634895	8.88E-04	-0.211	0.760	0.320	3	16,372,882	*RAFTLIN*	NM_015150.1	intron	-1,561
rs6017164	8.89E-04	0.208	0.760	0.461	20	41,815,520	*FAM112A*	NM_001008901.1	flanking_5UTR	-26,464
rs17014465	8.89E-04	0.206	0.760	0.065	1	207,666,881	*PLXNA2*	NM_025179	upstream	1,182,593
rs1788421	8.90E-04	-0.206	0.760	0.401	21	42,895,327	*SLC37A1*	NM_018964.3	flanking_3UTR	-20,708
rs8076615	8.90E-04	-0.208	0.760	0.270	17	56,183,231	*BCAS3*	NM_017679.2	intron	-3,809
rs17657594	8.90E-04	0.206	0.760	0.100	18	37,624,965	*PIK3C3*	NM_002647	upstream	164,232
rs6097867	8.90E-04	0.206	0.760	0.190	20	52,301,850	*DOK5*	NM_018431	upstream	223,738
rs11240358	8.91E-04	-0.206	0.760	0.101	1	203,337,196	*RBBP5*	NM_005057.2	intron	-155
rs7281173	8.93E-04	0.206	0.760	0.367	21	13,866,986	*ANKRD21*	NM_174981.2	flanking_5UTR	-37,383
rs3739238	8.94E-04	0.206	0.760	0.243	8	142,291,627	*DENND3*	NM_014957.2	flanking_3UTR	-16,544
rs6101284	8.95E-04	0.206	0.760	0.075	20	59,269,784	*CDH4*	NM_001794.2	intron	-6,396
rs6430138	8.95E-04	0.206	0.760	0.203	2	147,258,462	*ACVR2A*	NM_001616.3	flanking_5UTR	-1,060,578
rs7339421	8.95E-04	-0.206	0.760	0.455	13	101,576,026	*FGF14*	NM_004115.2	intron	-209,030
rs832174	8.95E-04	0.206	0.760	0.318	1	199,525,142	*PKP1*	NM_000299.2	intron	-4,551
rs7190251	8.95E-04	0.206	0.760	0.126	16	52,768,636	*IRX3*	NM_024336	downstream	106,077
rs4959663	8.96E-04	0.208	0.760	0.178	6	2,357,227	*GMDS*	NM_001500.2	flanking_5UTR	-166,382
rs10416645	8.97E-04	0.206	0.760	0.393	19	16,498,009	*CHERP*	NM_006387.4	intron	-521
rs2887115	8.97E-04	0.207	0.760	0.416	7	81,582,368	*CACNA2D1*	NM_000722.2	intron	-1,928
rs7783032	8.97E-04	0.206	0.760	0.393	7	77,189,641	*RSBN1L*	NM_198467.1	intron	-14,022
rs4851266	8.97E-04	-0.206	0.760	0.353	2	100,184,911	*LONRF2*	NM_198461	downstream	81,525
rs17023156	8.98E-04	0.206	0.760	0.205	2	83,186,494	*LOC388965*	NM_001013648.3	flanking_5UTR	-1,184,824

rs12748993	9.00E-04	-0.206	0.760	0.084	1	8,052,094	*ERRFI1*	NM_018948	upstream	43,139
rs11792221	9.01E-04	0.205	0.760	0.043	9	22,680,660	*ELAVL2*	NM_004432	downstream	1,001,149
rs3769203	9.02E-04	0.205	0.760	0.208	2	173,663,308	*ZAK*	NM_016653.1	intron	-664
rs10070440	9.03E-04	0.206	0.760	0.460	5	75,463,691	*SV2C*	NM_014979.1	coding	[359/220]
rs9666486	9.03E-04	-0.205	0.760	0.129	11	82,127,254	*MGC33846*	NM_175885.3	flanking_5UTR	-4,700
rs16828751	9.03E-04	0.205	0.760	0.129	3	119,862,969	*IGSF11*	NM_001015887	downstream	240,722
rs6829912	9.04E-04	-0.206	0.760	0.275	4	101,011,506	*DAPP1*	NM_014395.1	flanking_3UTR	-1,172
rs10964862	9.04E-04	0.207	0.760	0.293	9	21,141,553	*IFNW1*	NM_002177	upstream	9,409
rs1510973	9.05E-04	0.205	0.760	0.212	5	118,117,697	*DTWD2*	NM_173666.1	flanking_3UTR	-85,438
rs424513	9.05E-04	-0.206	0.760	0.211	21	41,633,548	*FAM3B*	NM_058186.3	intron	-1,250
rs7431008	9.05E-04	-0.207	0.760	0.310	3	19,829,535	*EFHB*	NM_144715.2	flanking_3UTR	-66,433
rs16937251	9.05E-04	-0.205	0.760	0.139	11	19,911,770	*NAV2*	NM_145117	CDS	0
rs9953346	9.07E-04	-0.205	0.760	0.054	18	63,574,561	*C18orf4*	NM_032160.2	flanking_5UTR	-239,614
rs4921336	9.08E-04	0.205	0.760	0.486	5	160,140,855	*PTTG1*	NM_004219.2	flanking_3UTR	-352,531
rs4820187	9.08E-04	0.205	0.760	0.478	22	33,977,722	*HMG2L1*	NM_001003681	upstream	5,767
rs12026645	9.09E-04	-0.205	0.760	0.476	1	104,363,904	*AMY1C*	NM_001008219.1	flanking_3UTR	-261,071
rs12536957	9.11E-04	0.205	0.760	0.123	7	38,603,053	*AMPH*	NM_001635	intron	0
rs750625	9.12E-04	0.205	0.760	0.212	8	41,645,071	*ANK1*	NM_020476.2	coding	[129/168]
rs6627955	9.12E-04	0.206	0.760	0.363	X	23,536,904	*PRDX4*	NM_006406	upstream	58,580
rs11097358	9.13E-04	0.205	0.760	0.379	4	94,283,082	*GRID2*	NM_001510.1	intron	-31,955
rs888060	9.15E-04	-0.205	0.760	0.069	14	76,796,486	*TMEM63C*	NM_020431.1	flanking_3UTR	-895
rs10138951	9.16E-04	0.206	0.760	0.062	14	80,096,067	*C14orf145*	NM_152446.2	intron	-668
rs1942637	9.16E-04	0.206	0.760	0.476	7	15,848,157	*MEOX2*	NM_005924.3	flanking_5UTR	-155,338
rs6503890	9.16E-04	0.206	0.760	0.349	17	54,418,949	*PPM1E*	NM_014906.3	flanking_3UTR	-1,630
rs13260349	9.17E-04	0.205	0.760	0.332	8	72,299,415	*EYA1*	NM_000503.3	intron	-7,603
rs9493891	9.17E-04	-0.205	0.760	0.082	6	134,665,533	*SGK*	NM_005627.2	flanking_5UTR	-127,838
rs1872235	9.17E-04	0.205	0.760	0.380	17	6,104,279	*AIPL1*	NM_001033054	downstream	163,505
rs17624303	9.17E-04	-0.206	0.760	0.180	2	104,514,850	*TMEM182*	NM_144632	downstream	1,714,540
rs8080955	9.17E-04	0.207	0.760	0.377	17	66,894,194	---	ENST0000038852	downstream	75,222
rs11633014	9.18E-04	0.205	0.760	0.259	15	76,333,244	*DNAJA4*	NM_018602.2	flanking_5UTR	-10,307
rs248940	9.18E-04	0.206	0.760	0.122	19	19,744,691	*FLJ46230*	NM_207463.1	flanking_5UTR	-27,626
rs6009820	9.18E-04	0.205	0.760	0.187	22	48,471,460	*LOC348645*	NM_198851.1	flanking_5UTR	-66,888
rs12787100	9.19E-04	0.206	0.760	0.144	11	11,342,916	*GALNTL4*	NM_198516.1	flanking_3UTR	-7,722
rs915832	9.19E-04	-0.205	0.760	0.366	21	42,148,564	*PRDM15*	NM_022115	intron	0
rs933408	9.19E-04	0.205	0.760	0.107	12	5,928,997	*VWF*	NM_000552	intron	0
rs10052226	9.20E-04	0.205	0.760	0.056	5	41,991,503	*FBXO4*	NM_012176.2	flanking_3UTR	-14,074
rs10053522	9.20E-04	0.205	0.760	0.056	5	41,997,379	*FBXO4*	NM_012176.2	flanking_3UTR	-19,950
rs9729540	9.20E-04	-0.205	0.760	0.150	1	105,310,874	*AMY1C*	NM_001008219.1	flanking_3UTR	-1,208,041
rs991024	9.20E-04	-0.205	0.760	0.150	1	105,309,678	---	ENST0000037007	downstream	1,206,843
rs10491499	9.21E-04	0.205	0.760	0.100	5	114,051,686	*KCNN2*	NM_021614.2	flanking_3UTR	-191,590
rs3807769	9.22E-04	0.205	0.760	0.228	7	77,517,414	*MAGI2*	NM_012301	intron	0
rs10199639	9.22E-04	-0.205	0.760	0.232	2	43,200,631	*ZFP36L2*	NM_006887	downstream	103,579
rs1386987	9.23E-04	0.205	0.760	0.316	3	7,880,067	*GRM7*	NM_000844.2	flanking_3UTR	-121,850
rs3105104	9.23E-04	-0.205	0.760	0.272	2	95,180,092	*ZNF514*	NM_032788.1	intron	-353
rs3105105	9.23E-04	-0.205	0.760	0.272	2	95,211,745	*ZNF2*	NM_021088.2	3UTR	[163/2047]
rs2540875	9.24E-04	0.205	0.760	0.204	14	90,781,224	*GPR68*	NM_003485.3	intron	-8,588

rs320637	9.24E-04	0.205	0.760	0.272	17	28,689,441	*ACCN1*	NM_183377.1	flanking_5UTR	-45,322
rs650073	9.24E-04	0.206	0.760	0.080	5	41,623,042	*PLCXD3*	NM_001005473.1	flanking_5UTR	-76,555
rs4327424	9.25E-04	-0.205	0.760	0.471	3	120,873,534	*COX17*	NM_005694	intron	0
rs7421044	9.26E-04	0.206	0.760	0.451	2	1,270,103	*SNTG2*	NM_018968	intron	0
rs1477869	9.27E-04	0.205	0.760	0.138	11	39,589,929	*LRRC4C*	NM_020929.1	flanking_3UTR	-502,400
rs12911494	9.27E-04	0.206	0.760	0.359	15	65,128,864	*SMAD3*	NM_005902	upstream	16,385
rs2916612	9.28E-04	0.205	0.760	0.173	5	89,614,530	*CETN3*	NM_004365.2	flanking_3UTR	-110,754
rs1408254	9.28E-04	0.205	0.760	0.332	6	15,012,306	---	ENST0000037914	upstream	208,629
rs203691	9.29E-04	-0.205	0.760	0.431	6	137,909,141	*OLIG3*	NM_175747.2	flanking_5UTR	-51,917
rs7157820	9.29E-04	-0.206	0.760	0.057	14	85,965,407	*FLRT2*	NM_013231.4	flanking_3UTR	-801,384
rs4835726	9.30E-04	0.210	0.760	0.363	5	138,761,710	*PACAP*	NM_016459.2	flanking_5UTR	-8,209
rs2205423	9.30E-04	-0.208	0.760	0.303	21	28,983,402	*N6AMT1*	NM_182749	downstream	182,998
rs1077009	9.30E-04	0.207	0.760	0.382	17	6,120,653	*AIPL1*	NM_001033054	downstream	147,131
rs6460895	9.31E-04	0.206	0.760	0.425	7	12,219,065	*TMEM106B*	NM_018374	upstream	1,888
rs11938985	9.32E-04	-0.205	0.760	0.067	4	56,474,015	*EXOC1*	NM_018261.3	flanking_3UTR	-8,014
rs2320624	9.32E-04	-0.205	0.760	0.260	2	95,220,338	*ZNF2*	NM_021088.2	flanking_3UTR	-6,546
rs11211641	9.33E-04	0.205	0.760	0.389	2	1,266,929	*SNTG2*	NM_018968.2	flanking_3UTR	-13,005
rs2407549	9.34E-04	-0.205	0.760	0.365	4	152,009,990	*LRBA*	NM_006726.1	intron	-1,083
rs1465882	9.34E-04	0.208	0.760	0.470	2	223,531,898	*KCNE4*	NM_080671	upstream	93,273
rs1945150	9.35E-04	-0.206	0.760	0.405	18	20,391,452	*HRH4*	NM_021624.2	flanking_3UTR	-77,533
rs486935	9.35E-04	0.205	0.760	0.248	11	79,375,627	*NARS2*	NM_024678.3	flanking_5UTR	-1,412,260
rs750087	9.36E-04	0.205	0.760	0.316	1	203,076,784	*NFASC*	NM_001005387.1	intron	-12,251
rs993870	9.36E-04	0.205	0.760	0.458	7	48,964,571	*ABCA13*	NM_152701.2	flanking_3UTR	-306,934
rs2042066	9.37E-04	-0.205	0.760	0.301	7	132,262,221	*CHCHD3*	NM_017812.1	intron	-41,176
rs313048	9.37E-04	-0.206	0.760	0.405	4	127,844,385	*PDZD6*	NM_015693.2	flanking_5UTR	-929,185
rs1978877	9.38E-04	0.205	0.760	0.369	15	91,378,387	*RGMA*	NM_020211.1	flanking_3UTR	-9,254
rs2829700	9.38E-04	0.205	0.760	0.464	21	25,616,795	*C21orf42*	NM_058184.1	flanking_3UTR	-63,215
rs6720541	9.38E-04	-0.205	0.760	0.304	2	76,000,263	*C2orf3*	NM_003203.3	flanking_5UTR	-208,433
rs12577327	9.39E-04	-0.205	0.760	0.107	11	24,355,673	*LUZP2*	NM_001009909.2	flanking_5UTR	-119,459
rs5926097	9.40E-04	0.205	0.760	0.371	X	22,766,000	*DDX53*	NM_182699.2	flanking_5UTR	-162,008
rs7737905	9.41E-04	0.205	0.760	0.480	5	59,684,473	---	ENST0000036514	upstream	48,738
rs12669338	9.42E-04	-0.205	0.760	0.237	7	12,464,663	*SCIN*	NM_033128.1	flanking_5UTR	-131,010
rs7725061	9.42E-04	-0.205	0.760	0.071	5	26,987,439	*CDH9*	NM_016279.2	intron	-35,650
rs8089034	9.42E-04	0.205	0.760	0.269	18	43,831,199	*SMAD2*	NM_001003652.1	flanking_5UTR	-119,978
rs7934715	9.43E-04	-0.205	0.760	0.127	11	20,480,233	*PRMT3*	NM_005788	intron	0
rs2697306	9.44E-04	0.205	0.760	0.317	2	197,783,919	*ANKRD44*	NM_153697.1	flanking_5UTR	-12,912
rs7242469	9.44E-04	-0.205	0.760	0.462	18	20,027,095	*OSBPL1A*	NM_080597.2	intron	-2,992
rs313063	9.44E-04	-0.205	0.760	0.402	4	127,840,301	*FAT4*	NM_024582	downstream	1,207,928
rs1944323	9.44E-04	-0.205	0.760	0.404	18	31,933,579	*P15RS*	NM_018170	upstream	32,088
rs11606889	9.45E-04	-0.205	0.760	0.111	11	6,526,472	*DNHD1*	NM_144666.1	flanking_5UTR	-17,439
rs8071916	9.45E-04	0.205	0.760	0.362	17	54,015,278	*TEX14*	NM_198393.2	intron	-1,188
rs9582378	9.45E-04	-0.205	0.760	0.125	13	99,676,485	*PCCA*	NM_000282.2	intron	-9,612
rs1473148	9.46E-04	0.206	0.760	0.456	7	48,970,448	*ABCA13*	NM_152701.2	flanking_3UTR	-312,811
rs7712871	9.46E-04	-0.205	0.760	0.405	5	14,105,904	*TRIO*	NM_007118.2	flanking_5UTR	-90,925
rs528204	9.46E-04	0.205	0.760	0.328	9	22,731,961	*DMRTA1*	NM_022160	downstream	289,489
rs277552	9.46E-04	0.205	0.760	0.295	2	121,348,924	*GLI2*	NM_005270	intron	0

rs10498817	9.47E-04	-0.205	0.760	0.076	6	62,647,284	*KHDRBS2*	NM_152688.1	intron	-15,215
rs7726919	9.47E-04	-0.205	0.760	0.342	5	32,002,949	*PDZK3*	NM_015022.2	intron	-16,069
rs2191299	9.48E-04	0.205	0.760	0.457	7	48,968,438	*ABCA13*	NM_152701.2	flanking_3UTR	-310,801
rs3806782	9.48E-04	0.205	0.760	0.115	4	156,807,337	*GUCY1A3*	NM_000856.2	5UTR	[9/230]
rs7075770	9.48E-04	-0.205	0.760	0.067	10	86,962,818	*GRID1*	NM_017551.1	flanking_3UTR	-386,474
rs7357214	9.48E-04	0.205	0.760	0.457	7	48,968,194	*VWC2*	NM_198570	upstream	815,604
rs10745532	9.48E-04	0.205	0.760	0.366	12	89,776,623	---	ENST0000038811	downstream	1,131,693
rs12944948	9.49E-04	0.205	0.760	0.360	17	54,306,256	*PPM1E*	NM_014906.3	intron	-81,536
rs2555561	9.49E-04	0.205	0.760	0.065	8	67,594,660	*C8orf46*	NM_152765.2	flanking_3UTR	-1,366
rs8071280	9.49E-04	0.205	0.760	0.360	17	54,365,007	*PPM1E*	NM_014906.3	intron	-22,785
rs9971900	9.49E-04	-0.205	0.760	0.087	12	90,056,456	*LUM*	NM_002345	upstream	26,783
rs13390640	9.50E-04	-0.205	0.760	0.211	2	52,185,961	*NRXN1*	NM_004801.2	flanking_5UTR	-1,076,854
rs4843460	9.50E-04	0.205	0.760	0.471	16	85,382,973	*FOXL1*	NM_005250	downstream	210,177
rs4574807	9.52E-04	0.205	0.760	0.288	8	82,135,814	*PAG1*	NM_018440	intron	0
rs1202584	9.53E-04	0.205	0.760	0.346	1	229,068,773	*C1orf198*	NM_032800.1	intron	-1,776
rs7745875	9.53E-04	0.205	0.760	0.083	6	132,459,257	*CTGF*	NM_001901.1	flanking_5UTR	-145,102
rs11134782	9.53E-04	0.205	0.760	0.306	5	172,635,604	---	ENST0000036440	upstream	21,704
rs429294	9.54E-04	-0.204	0.760	0.406	19	16,287,889	*KLF2*	NM_016270.2	flanking_5UTR	-8,762
rs16957284	9.55E-04	0.205	0.760	0.067	15	41,034,859	*UBR1*	NM_174916.1	intron	-2,644
rs3824026	9.55E-04	-0.204	0.760	0.332	7	122,628,811	*SLC13A1*	NM_022444.3	flanking_5UTR	-1,550
rs4245154	9.55E-04	0.204	0.760	0.301	11	112,893,884	*DRD2*	NM_016574	upstream	42,781
rs10028040	9.55E-04	-0.204	0.760	0.138	4	151,884,242	*LRBA*	NM_006726	intron	0
rs1400142	9.57E-04	0.205	0.760	0.344	12	71,728,762	*TRHDE*	NM_013381.1	flanking_3UTR	-383,073
rs6081870	9.57E-04	-0.205	0.760	0.162	20	20,018,642	*C20orf26*	NM_015585.2	intron	-846
rs6589865	9.57E-04	0.205	0.760	0.378	11	120,620,416	*SC5DL*	NM_006918.3	flanking_5UTR	-48,221
rs4850777	9.57E-04	-0.205	0.760	0.358	2	197,840,769	---	ENST0000033720	intron	0
rs1966272	9.58E-04	-0.204	0.760	0.270	2	95,289,881	*PROM2*	NM_144707.1	flanking_5UTR	-14,047
rs6748967	9.58E-04	-0.204	0.760	0.270	2	95,277,822	*PROM2*	NM_144707.1	flanking_5UTR	-26,106
rs3138262	9.59E-04	-0.204	0.760	0.087	12	90,071,161	*DCN*	NM_133504.2	intron	-64
rs3138288	9.59E-04	-0.204	0.760	0.087	12	90,064,234	*DCN*	NM_133507.2	intron	-74
rs10875467	9.60E-04	0.204	0.760	0.263	8	142,302,578	---	ENST0000002406	intron	0
rs10260109	9.61E-04	-0.205	0.760	0.467	7	14,822,880	*DGKB*	NM_004080.1	intron	-24,464
rs1929239	9.62E-04	-0.204	0.761	0.295	10	30,164,623	*SVIL*	NM_003174.2	flanking_5UTR	-99,887
rs7190134	9.62E-04	0.204	0.761	0.092	16	66,939,017	*PRMT7*	NM_019023.1	intron	-18
rs10859101	9.63E-04	-0.206	0.761	0.100	12	89,968,576	*KERA*	NM_007035.3	3UTR	[177/677]
rs13145112	9.65E-04	-0.205	0.761	0.055	4	161,426,162	*FSTL5*	NM_020116.2	flanking_3UTR	-1,098,337
rs3793260	9.65E-04	0.204	0.761	0.082	7	29,464,881	*CHN2*	NM_004067.1	intron	-21,539
rs11109114	9.66E-04	-0.204	0.761	0.295	12	96,494,269	*NCRMS*	XR_000219.2	flanking_3UTR	-11,331
rs2822965	9.66E-04	-0.204	0.761	0.395	21	15,163,739	*NRIP1*	NM_003489.2	flanking_3UTR	-91,688
rs7686545	9.66E-04	-0.204	0.761	0.371	4	94,222,747	*GRID2*	NM_001510.1	intron	-2,422
rs10038589	9.66E-04	0.204	0.761	0.391	5	75,805,014	*IQGAP2*	NM_006633	intron	0
rs724302	9.67E-04	0.204	0.761	0.207	2	83,169,061	*LOC388965*	NM_001013648.3	flanking_5UTR	-1,202,257
rs9992474	9.69E-04	-0.206	0.761	0.097	4	152,100,215	*LRBA*	NM_006726.1	intron	-30,548
rs1445442	9.69E-04	-0.205	0.761	0.395	12	63,577,561	---	ENST0000022908	downstream	19,211
rs1123250	9.70E-04	0.204	0.762	0.413	1	110,487,879	*SLC6A17*	NM_001010898.1	flanking_5UTR	-6,752
rs1338035	9.72E-04	0.204	0.763	0.165	1	189,893,627	*RGS18*	NM_130782.2	flanking_5UTR	-500,588

rs167132	9.72E-04	-0.205	0.763	0.117	5	57,941,231	*RAB3C*	NM_138453.2	intron	-7,996
rs3130712	9.74E-04	-0.204	0.764	0.471	6	31,317,489	*HLA-C*	NM_002117.4	flanking_3UTR	-27,019
rs10947600	9.76E-04	-0.204	0.764	0.230	6	36,377,581	*PNPLA1*	NM_173676.1	intron	-35
rs13429262	9.76E-04	-0.204	0.764	0.179	2	104,520,811	*POU3F3*	NM_006236.1	flanking_5UTR	-317,590
rs12428099	9.76E-04	0.204	0.764	0.210	13	22,248,341	---	ENST0000036298	upstream	26,943
rs7244057	9.78E-04	0.204	0.764	0.196	18	43,833,274	*SMAD2*	NM_001003652.1	flanking_5UTR	-122,053
rs11693317	9.78E-04	-0.205	0.764	0.143	2	48,685,918	*SALF*	NM_172311	intron	0
rs8048956	9.78E-04	-0.205	0.764	0.078	16	73,812,467	*CTRB1*	NM_001906	intron	0
rs16848229	9.80E-04	0.204	0.764	0.149	1	199,523,996	*PKP1*	NM_001005337.1	intron	-4,341
rs2188910	9.80E-04	0.204	0.764	0.409	2	130,052,599	---	ENST0000037598	downstream	224,953
rs1525183	9.81E-04	0.204	0.764	0.236	7	108,290,244	*LOC154907*	NM_001024607.1	flanking_3UTR	-21,030
rs1525181	9.81E-04	0.204	0.764	0.236	7	108,292,444	*DNAJB9*	NM_012328	downstream	289,914
rs2168006	9.82E-04	-0.206	0.764	0.389	5	99,187,494	---	ENST0000036348	upstream	887,143
rs1156560	9.83E-04	-0.204	0.764	0.239	2	157,845,249	*GALNT5*	NM_014568	intron	0
rs2830992	9.84E-04	0.204	0.764	0.264	21	27,810,551	*C21orf94*	NM_145180.2	flanking_5UTR	-497,021
rs640786	9.84E-04	-0.204	0.764	0.105	11	121,606,766	*BRCC2*	NM_001001786.1	flanking_5UTR	-114,633
rs6085256	9.85E-04	0.204	0.764	0.462	20	5,655,548	*FLJ25067*	NM_152504.2	flanking_5UTR	-23,495
rs900114	9.85E-04	-0.207	0.764	0.465	8	68,690,444	*CPA6*	NM_020361.2	intron	-8,521
rs963064	9.85E-04	0.205	0.764	0.159	2	83,189,326	*LOC388965*	NM_001013648.3	flanking_5UTR	-1,181,992
rs331538	9.86E-04	0.206	0.764	0.363	11	4,423,409	*OR52K2*	NM_001005172	upstream	3,737
rs7610524	9.87E-04	0.204	0.764	0.295	3	48,370,376	*NME6*	NM_005793	upstream	52,524
rs6874394	9.87E-04	-0.205	0.764	0.361	5	27,028,573	*CDH9*	NM_016279	intron	0
rs10488060	9.88E-04	-0.204	0.764	0.138	7	156,241,406	*LMBR1*	NM_022458.2	intron	-442
rs2101367	9.88E-04	0.204	0.764	0.175	3	119,360,100	*IGSF11*	NM_001015887	downstream	743,591
rs4129152	9.89E-04	-0.205	0.764	0.099	4	136,232,687	*PCDH10*	NM_032961.1	flanking_3UTR	-1,900,505
rs6475619	9.89E-04	-0.204	0.764	0.208	9	22,197,255	*CDKN2A*	NM_058195	upstream	212,765
rs10490835	9.90E-04	0.204	0.764	0.098	3	24,621,112	*THRB*	NM_000461.2	flanking_5UTR	-109,795
rs7149928	9.91E-04	-0.204	0.764	0.149	14	53,168,209	*BMP4*	NM_001202.2	flanking_3UTR	-317,998
rs1516709	9.92E-04	0.204	0.764	0.203	6	62,740,028	*KHDRBS2*	NM_152688.1	intron	-5,902
rs7745661	9.93E-04	0.204	0.764	0.257	6	130,955,547	*EPB41L2*	NM_001431	downstream	246,636
rs7719291	9.93E-04	-0.207	0.764	0.078	5	50,941,736	*ISL1*	NM_002202	downstream	215,712
rs4375154	9.94E-04	0.204	0.764	0.304	X	23,547,037	*PRDX4*	NM_006406.1	flanking_5UTR	-48,529
rs5972373	9.94E-04	0.204	0.764	0.100	X	31,249,023	*DMD*	NM_000109.2	intron	-2,613
rs7913086	9.94E-04	-0.204	0.764	0.098	10	65,892,699	*ANXA2P3*	NR_001446.1	flanking_5UTR	-362,587
rs7920723	9.94E-04	-0.204	0.764	0.098	10	65,876,810	*REEP3*	NM_001001330	downstream	821,923
rs1296757	9.95E-04	-0.204	0.764	0.096	22	16,369,378	*SLC25A18*	NM_031481.1	flanking_5UTR	-53,805
rs1537994	9.95E-04	-0.204	0.764	0.171	13	84,444,563	*SLITRK6*	NM_032229.2	flanking_3UTR	-820,360
rs3913369	9.96E-04	-0.204	0.764	0.096	3	55,456,115	*WNT5A*	NM_003392.3	flanking_3UTR	-18,668
rs13034178	9.97E-04	-0.204	0.765	0.228	2	151,610,847	*RBM43*	NM_198557	downstream	203,309
rs12566014	9.98E-04	-0.204	0.765	0.205	1	207,148,108	*PLXNA2*	NM_025179.2	flanking_5UTR	-664,369
rs4814837	9.98E-04	-0.204	0.765	0.272	20	19,189,680	*SLC24A3*	NM_020689.3	intron	-19,923

Supplementary Table 3. 76 SNPs were associated with IC50s for both paclitaxel and docetaxel with p-values $<10^{-3}$.

SNP ID	P value (Pac)	R value (Pac)	Q value (Pac)	P value (Doc)	R value (Doc)	Q value (Doc)	MAF	Chr	Position	GeneSymbol	RefSeq ID	Location	Location Relative to Gene (bp)
rs548726	4.78E-07	0.307	0.163	4.83E-04	0.216	0.756	0.203	1	5,836,208	*NPHP4*	NM_015102.2	flanking_3UTR	-9,249
rs7260598	6.27E-06	-0.277	0.702	2.46E-05	-0.259	0.671	0.165	19	24,014,626	*ZNF254*	NM_203282	upstream	47,190
rs915832	6.76E-06	-0.276	0.702	9.19E-04	-0.205	0.760	0.366	21	42,148,564	*PRDM15*	NM_022115	intron	0
rs9698139	9.54E-06	0.274	0.702	8.61E-04	0.208	0.760	0.476	X	122,799,241	*BIRC4*	NM_001167.2	flanking_5UTR	-22,488
rs9883082	1.75E-05	-0.264	0.864	2.56E-04	-0.226	0.754	0.317	3	7,858,963	*GRM7*	NM_000844.2	flanking_3UTR	-100,746
rs12898337	2.08E-05	0.263	0.864	2.44E-05	0.261	0.671	0.35	15	97,111,878	*IGF1R*	NM_000875.2	intron	-43,019
rs6460895	2.90E-05	0.259	0.864	9.31E-04	0.206	0.760	0.425	7	12,219,065	*TMEM106B*	NM_018374	upstream	1,888
rs6589877	5.93E-05	0.247	0.864	4.90E-04	0.216	0.757	0.101	11	120,701,161	*SC5DL*	NM_006918.3	flanking_3UTR	-16,582
rs12527264	6.67E-05	-0.246	0.864	4.56E-04	-0.217	0.756	0.134	6	37,607,820	*C6orf129*	NM_138493.1	flanking_5UTR	-32,144
rs17304569	7.76E-05	-0.243	0.864	6.74E-05	-0.245	0.683	0.163	19	24,032,745	*ZNF254*	NM_203282	upstream	29,071
rs7498372	8.18E-05	0.243	0.864	1.66E-04	0.233	0.754	0.431	16	29,818,226	*SEZ6L2*	NM_201575.1	flanking_5UTR	-152
rs766045	1.03E-04	-0.240	0.904	3.38E-04	-0.222	0.754	0.084	8	3,968,386	*CSMD1*	NM_033225.3	intron	-91,357
rs6598542	1.07E-04	0.241	0.904	2.01E-04	0.232	0.754	0.369	15	97,110,579	*IGF1R*	NM_000875	intron	0
rs4917139	1.12E-04	-0.238	0.904	2.20E-04	-0.228	0.754	0.203	7	48,232,695	*ABCA13*	NM_152701.2	intron	-1,247
rs3823612	1.24E-04	0.241	0.927	4.44E-04	0.221	0.756	0.404	7	12,225,280	*TMEM106B*	NM_018374	intron	0
rs1468804	1.42E-04	0.235	0.927	4.80E-04	0.216	0.756	0.408	7	12,242,033	*TMEM106B*	NM_018374	3UTR	0
rs1400142	1.51E-04	0.234	0.927	9.57E-04	0.205	0.760	0.344	12	71,728,762	*TRHDE*	NM_013381.1	flanking_3UTR	-383,073
rs17404627	1.54E-04	-0.234	0.927	6.63E-04	-0.211	0.760	0.096	8	3,957,010	*CSMD1*	NM_033225.3	intron	-79,981
rs6583476	1.78E-04	-0.233	0.927	1.96E-04	-0.232	0.754	0.202	7	48,214,960	*ABCA13*	NM_152701.2	intron	-6,448
rs6101282	1.82E-04	0.231	0.927	6.07E-04	0.212	0.760	0.076	20	59,267,601	*CDH4*	NM_001794	intron	0
rs6101283	1.82E-04	0.231	0.927	6.07E-04	0.212	0.760	0.076	20	59,267,891	*CDH4*	NM_001794	intron	0
rs2488401	1.88E-04	0.230	0.927	3.89E-04	0.219	0.754	0.217	1	195,969,024	*DENND1B*	NM_144977.1	intron	-2,315
rs3733870	1.89E-04	-0.231	0.927	5.26E-04	-0.215	0.759	0.198	5	159,411,119	*TTC1*	NM_003314.1	intron	-351
rs7115326	2.09E-04	-0.229	0.927	7.24E-04	-0.209	0.760	0.359	11	58,034,925	*OR5B21*	NM_001005218.	flanking_5UTR	-2,771
rs7176023	2.11E-04	-0.230	0.927	8.22E-05	-0.244	0.732	0.157	15	62,888,060	*RBPMS2*	NM_194272	upstream	33,221
rs17160491	2.60E-04	-0.226	0.927	7.59E-04	-0.208	0.760	0.299	19	8,418,940	*HNRPM*	NM_005968.2	intron	-2,945
rs17620029	2.63E-04	-0.226	0.927	1.39E-04	-0.235	0.754	0.135	19	24,146,152	*ZNF539*	NM_203282.1	flanking_3UTR	-41,659
rs10500223	2.80E-04	-0.226	0.927	2.37E-04	-0.228	0.754	0.128	19	24,137,864	*ZNF539*	NM_203282.1	flanking_3UTR	-33,371
rs4893555	2.98E-04	-0.223	0.927	8.00E-04	-0.207	0.760	0.303	X	28,782,000	*IL1RAPL1*	NM_014271	intron	0
rs4893599	2.98E-04	-0.223	0.927	8.00E-04	-0.207	0.760	0.303	X	28,782,529	*IL1RAPL1*	NM_014271	intron	0
rs2830987	3.02E-04	0.225	0.927	5.42E-04	0.215	0.759	0.245	21	27,798,286	---	ENST00000324	upstream	509,286
rs7724279	3.04E-04	-0.224	0.927	3.94E-04	-0.220	0.754	0.292	5	31,993,642	*PDZK3*	NM_015022.2	intron	-25,376
rs2160954	3.06E-04	-0.223	0.927	4.38E-04	-0.217	0.756	0.357	3	7,861,502	*GRM7*	NM_181875	downstream	153,596
rs6101284	3.08E-04	0.223	0.927	8.95E-04	0.206	0.760	0.075	20	59,269,784	*CDH4*	NM_001794.2	intron	-6,396
rs17162040	3.18E-04	0.222	0.927	4.48E-04	0.217	0.756	0.103	1	27,755,814	*AHDC1*	NM_001029882.	intron	-1,990
rs932680	3.36E-04	0.222	0.927	3.73E-04	0.221	0.754	0.35	6	44,609,851	*RUNX2*	NM_001015051	upstream	888,041
rs1060700	3.38E-04	0.222	0.927	3.64E-04	0.221	0.754	0.407	7	12,242,343	*TMEM106B*	NM_018374.2	3UTR	[4216/1068]
rs2830991	3.84E-04	0.219	0.927	8.29E-04	0.207	0.760	0.255	21	27,808,439	---	ENST00000324	upstream	499,133
rs2830992	3.92E-04	0.219	0.927	9.84E-04	0.204	0.764	0.264	21	27,810,551	*C21orf94*	NM_145180.2	flanking_5UTR	-497,021
rs7637849	3.95E-04	-0.219	0.927	1.90E-04	-0.230	0.754	0.082	3	38,634,279	*SCN5A*	NM_198056.1	intron	-3,059
rs11878982	3.97E-04	-0.219	0.927	2.52E-04	-0.226	0.754	0.114	19	4,992,049	*JMJD2B*	NM_015015.1	intron	-99
rs2839398	4.06E-04	-0.219	0.927	1.99E-04	-0.230	0.754	0.219	21	42,159,421	*PRDM15*	NM_022115	intron	0
rs17576350	4.09E-04	-0.218	0.927	2.10E-04	-0.229	0.754	0.141	4	164,665,964	*FLJ11184*	NM_018352	downstream	4,857
rs2130017	4.09E-04	0.224	0.927	7.69E-06	0.281	0.409	0.119	11	89,142,024	*TRIM49*	NM_020358.2	flanking_3UTR	-28,447
rs7166999	4.12E-04	0.218	0.927	6.43E-05	0.246	0.683	0.473	15	97,110,328	*IGF1R*	NM_000875.2	intron	-41,469
rs6843038	4.17E-04	0.219	0.927	7.82E-04	0.208	0.760	0.365	4	182,162,059	*MGC45800*	NM_178838.3	flanking_3UTR	-1,137,738
rs201460	4.18E-04	-0.218	0.927	1.42E-05	-0.267	0.635	0.246	7	101,528,291	*CUTL1*	NM_001913.2	intron	-790
rs12954803	4.22E-04	-0.218	0.927	7.54E-06	-0.275	0.409	0.078	18	20,390,567	*HRH4*	NM_021624	downstream	79,000
rs3913369	4.59E-04	-0.217	0.927	9.96E-04	-0.204	0.764	0.096	3	55,456,115	*WNT5A*	NM_003392.3	flanking_3UTR	-18,668
rs8021868	4.65E-04	-0.218	0.927	7.76E-04	-0.209	0.760	0.172	14	56,861,066	*NAT12*	NM_001011713	upstream	65,958

rs17327860	4.89E-04	-0.216	0.927	3.98E-04	-0.219	0.754	0.168	3	55,437,613	*LRTM1*	NM_020678	upstream	500,501
rs7582078	4.91E-04	0.216	0.927	1.08E-04	0.240	0.753	0.454	2	208,355,096	*FZD5*	NM_003468.2	flanking_5UTR	-12,733
rs10483481	4.92E-04	-0.215	0.927	2.89E-04	-0.224	0.754	0.496	14	36,515,582	*SLC25A21*	NM_030631	intron	0
rs11079337	5.10E-04	-0.215	0.927	7.85E-04	-0.208	0.760	0.338	17	53,517,495	*DYNLL2*	NM_080677.1	intron	-1,452
rs2830988	5.27E-04	0.215	0.927	2.87E-04	0.224	0.754	0.255	21	27,799,163	---	ENST00000324[illegible]	upstream	508,409
rs10516088	5.57E-04	-0.213	0.927	5.22E-04	-0.214	0.759	0.194	5	171,083,028	*FBXW11*	NM_012300.2	flanking_3UTR	-138,133
rs4319365	5.59E-04	0.213	0.927	1.44E-04	0.234	0.754	0.426	1	56,680,308	*PPAP2B*	NM_003713.3	flanking_3UTR	-52,713
rs9998109	5.74E-04	-0.213	0.927	4.66E-05	-0.251	0.677	0.23	4	118,464,432	*TRAM1L1*	NM_152402.1	flanking_5UTR	-238,252
rs7111680	5.85E-04	0.213	0.927	1.45E-04	0.235	0.754	0.237	11	89,084,402	*PSMAL*	NM_153696	downstream	12,871
rs11203190	6.68E-04	-0.210	0.927	1.48E-04	-0.234	0.754	0.226	21	42,158,582	*PRDM15*	NM_022115.2	intron	-1,896
rs17079623	6.94E-04	0.210	0.927	3.32E-04	0.222	0.754	0.19	18	64,544,220	*TXNDC10*	NM_019022.3	flanking_5UTR	-10,887
rs12121863	7.42E-04	0.209	0.927	6.18E-04	0.212	0.760	0.188	1	196,052,043	*C1orf53*	NM_001024594	upstream	86,357
rs11592006	7.58E-04	-0.209	0.927	2.71E-04	-0.225	0.754	0.111	10	11,696,606	*ECHDC3*	NM_024693	upstream	127,765
rs6816145	7.71E-04	-0.208	0.927	4.14E-04	-0.219	0.756	0.136	4	164,638,444	*FLJ11184*	NM_018352.1	intron	-3,038
rs6569759	7.82E-04	0.208	0.927	1.55E-04	0.234	0.754	0.4	6	132,174,809	*ENPP1*	NM_006208.1	intron	-3,701
rs12904944	8.24E-04	0.207	0.927	3.15E-04	0.223	0.754	0.371	15	65,148,828	*SMAD3*	NM_005902.3	intron	-3,076
rs4751158	8.44E-04	-0.207	0.927	7.72E-04	-0.208	0.760	0.451	10	131,700,143	*EBF3*	NM_001005463	upstream	48,048
rs17763056	8.69E-04	-0.207	0.927	1.80E-04	-0.232	0.754	0.073	20	16,807,716	*OTOR*	NM_020157.2	flanking_3UTR	-126,907
rs11145281	8.76E-04	-0.206	0.927	3.26E-05	-0.256	0.671	0.278	9	78,899,644	*VPS13A*	NM_001018037	upstream	82,537
rs4888213	8.90E-04	-0.206	0.927	3.77E-04	-0.221	0.754	0.109	16	72,918,430	*PSMD7*	NM_002811.3	flanking_3UTR	-20,743
rs9868927	9.49E-04	0.205	0.927	2.72E-04	0.225	0.754	0.399	3	174,822,471	*NLGN1*	NM_014932.2	intron	-16,896
rs17473108	9.49E-04	-0.205	0.927	7.73E-04	-0.208	0.760	0.145	4	164,683,228	*March01gene*	NM_017923.2	intron	-2,970
rs1995274	9.81E-04	0.204	0.927	6.51E-04	0.211	0.760	0.298	5	61,201,717	*FLJ37543*	NM_173667.1	flanking_3UTR	-163,598
rs6694114	9.88E-04	-0.204	0.927	3.97E-05	-0.253	0.677	0.493	1	56,682,138	*PPAP2B*	NM_003713.3	flanking_3UTR	-50,883
rs3750263	9.92E-04	-0.204	0.927	2.03E-04	-0.229	0.754	0.158	8	29,036,982	*KIF13B*	NM_015254.2	intron	-54
rs17177982	9.94E-04	-0.205	0.927	1.62E-04	-0.234	0.754	0.337	14	36,505,046	*SLC25A21*	NM_030631	intron	0

Supplementary Table 4. Results of Cox regression analysis with overall survival for either SCLC or NSCLC patients. The SNPs associated with overall survival with p-value < 0.05 are highlighted.

SNP ID	NSCLC				SCLC				MAF	Chr	Position	GeneSymbol	RefSeqID	Location	Location Relative to Gene (bp)	Call Rate	HWE	drug
	P value	HR	HR_lcl	HR_ucl	P value	HR	HR_lcl	HR_ucl										
rs10120811	0.911	0.993	0.871	1.131	0.225	1.245	0.874	1.775	0.254	9	106,278,970	*OR13F1*	NM_001004485	upstream	27,395	0.996	0.658	Paclitaxel
rs10193067	0.160	1.201	0.930	1.550	0.039	0.372	0.145	0.953	0.044	2	52,543,917	*ASB3*	NM_145863.1	flanking_3UTR	-1,206,705	0.997	0.137	Paclitaxel
rs1019307	0.781	0.984	0.877	1.103	0.600	1.099	0.771	1.567	0.425	7	12,218,315	*TMEM106B*	ENST00000262042	upstream	575,476	0.996	0.238	Paclitaxel
rs1030334	0.404	0.953	0.852	1.067	0.875	0.969	0.657	1.431	0.485	2	56,554,449	*FANCL*	NM_018062	downstream	1,685,435	0.997	0.885	Paclitaxel
rs1037576	0.730	1.021	0.908	1.149	0.198	1.270	0.883	1.827	0.291	6	67,429,648	---	ENST00000364559	downstream	597,833	0.997	0.048	Paclitaxel
rs10483481	0.225	1.075	0.957	1.207	0.747	0.941	0.652	1.359	0.331	14	36,515,582	*SLC25A21*	NM_030631	intron	0	0.997	0.786	Dox&Pac
rs10495194	0.143	1.121	0.962	1.305	0.557	1.154	0.715	1.862	0.159	1	220,800,594	*TAF1A*	NM_139352.1	intron	-735	0.996	1.000	Paclitaxel
rs10500223	0.111	0.866	0.726	1.033	0.969	0.991	0.631	1.558	0.129	19	24,137,864	*ZNF539*	NM_203282.1	flanking_3UTR	-33,371	0.993	0.010	Dox&Pac
rs10516088	0.758	1.020	0.901	1.153	0.194	0.816	0.600	1.109	0.310	5	171,083,028	*FBXW11*	NM_012300.2	flanking_3UTR	-138,133	0.996	0.501	Dox&Pac
rs10521792	0.654	0.968	0.839	1.117	0.703	0.920	0.600	1.412	0.113	X	137,945,075	*FGF13*	NM_033642.1	flanking_5UTR	-295,894	0.997	0.109	Paclitaxel
rs1060700	0.790	0.984	0.877	1.105	0.693	1.075	0.751	1.540	0.430	7	12,242,343	*TMEM106B*	NM_018374.2	3UTR	[4216/1068]	0.997	0.117	Dox&Pac
rs1098182	0.637	0.950	0.767	1.177	0.107	0.635	0.365	1.104	0.080	1	43,558,355	*TIE1*	NM_005424	intron	0	0.997	0.870	Paclitaxel
rs1106697	0.016	1.237	1.041	1.469	0.007	1.875	1.186	2.964	0.106	7	155,365,705	*SHH*	NM_000193	upstream	67,977	0.981	0.612	Paclitaxel
rs11079337	0.007	1.168	1.043	1.307	0.462	1.155	0.786	1.698	0.366	17	53,517,495	*DYNLL2*	NM_080677.1	intron	-1,452	0.997	0.959	Dox&Pac
rs11203190	0.702	0.967	0.812	1.150	0.548	1.169	0.703	1.944	0.096	21	42,158,582	*PRDM15*	NM_022115.2	intron	-1,896	0.997	0.404	Dox&Pac
rs11222869	0.940	1.004	0.898	1.123	0.165	0.787	0.561	1.103	0.441	11	131,406,958	*HNT*	NM_016522	intron	0	0.996	0.381	Paclitaxel
rs1154818	0.506	0.933	0.762	1.143	0.514	1.270	0.619	2.603	0.084	12	67,127,543	*MDM1*	NM_020128.1	flanking_5UTR	-115,193	0.997	0.536	Paclitaxel
rs1159899	0.824	1.018	0.869	1.193	0.781	1.064	0.688	1.645	0.155	3	25,399,432	---	ENST00000264330	intron	0	0.997	0.066	Paclitaxel
rs11629576	0.124	1.090	0.977	1.217	0.011	0.637	0.450	0.902	0.447	15	76,294,371	*ACSBG1*	NM_015162.3	intron	-6,872	0.997	0.382	Paclitaxel
rs11647148	0.643	0.962	0.818	1.132	0.857	1.048	0.627	1.753	0.127	16	81,995,895	*CDH13*	NM_001257.3	intron	-59,783	0.997	0.914	Paclitaxel
rs11744434	0.784	0.982	0.860	1.120	0.251	0.788	0.524	1.184	0.228	5	10,164,180	*LOC134145*	NM_199133.1	flanking_3UTR	-115,265	0.997	0.633	Paclitaxel
rs11822830	0.440	1.046	0.932	1.174	0.309	1.237	0.822	1.862	0.413	11	128,966,226	*BARX2*	NM_003658	downstream	139,212	0.997	0.621	Paclitaxel
rs11878982	0.668	1.031	0.896	1.187	0.572	0.884	0.578	1.354	0.217	19	4,992,049	*JMJD2B*	NM_015015.1	intron	-99	0.996	0.572	Dox&Pac
rs12121863	0.058	1.154	0.995	1.338	0.067	1.573	0.969	2.556	0.185	1	196,052,043	*C1orf53*	NM_001024594	upstream	86,357	0.997	0.874	Dox&Pac
rs12194460	0.618	1.031	0.916	1.160	0.198	1.270	0.883	1.827	0.289	6	67,392,335	---	ENST00000365187	downstream	103,120	0.996	0.080	Paclitaxel
rs12201163	0.752	0.981	0.870	1.106	0.172	0.766	0.522	1.124	0.333	6	67,545,449	*LOC442229*	XR_000273.2	flanking_3UTR	-989,356	0.997	0.914	Paclitaxel
rs1227969	0.699	0.978	0.873	1.095	0.687	1.080	0.744	1.567	0.380	10	70,933,270	*TSPAN15*	NM_012339	intron	0	0.996	0.760	Paclitaxel
rs12367762	0.378	0.924	0.775	1.102	0.226	0.677	0.361	1.272	0.129	12	39,900,866	---	ENST00000380795	intron	0	0.997	0.165	Paclitaxel
rs1236904	0.701	0.978	0.873	1.095	0.687	1.080	0.744	1.567	0.381	10	70,937,855	*TSPAN15*	ENST00000242462	downstream	63,605	0.997	0.760	Paclitaxel
rs12377344	0.555	1.036	0.921	1.165	0.415	0.886	0.663	1.184	0.350	9	19,871,986	*SLC24A2*	NM_020344.1	flanking_5UTR	-95,060	0.997	0.597	Paclitaxel
rs12527264	0.659	0.959	0.798	1.153	0.182	1.395	0.856	2.276	0.108	6	37,607,820	*C6orf129*	NM_138493.1	flanking_5UTR	-32,144	0.997	0.617	Dox&Pac
rs12657996	0.617	1.033	0.910	1.172	0.691	1.078	0.746	1.557	0.237	5	158,836,891	*IL12B*	ENST00000306675	upstream	439,427	0.997	0.389	Paclitaxel
rs12703414	0.129	1.119	0.968	1.295	0.457	1.218	0.724	2.049	0.163	7	141,336,135	*MGAM*	NM_004668.1	flanking_5UTR	-6,013	0.997	1.000	Paclitaxel
rs12787230	0.433	1.047	0.933	1.176	0.309	1.237	0.822	1.862	0.414	11	128,965,050	*BARX2*	NM_003658.3	flanking_3UTR	-138,025	0.997	0.692	Paclitaxel
rs1281400	0.339	0.947	0.847	1.059	0.836	0.966	0.695	1.342	0.487	1	82,828,213	*LPHN2*	NM_012302	downstream	597,518	0.996	0.885	Paclitaxel
rs12898337	0.168	1.086	0.966	1.222	0.093	0.747	0.531	1.050	0.347	15	97,111,878	*IGF1R*	NM_000875.2	intron	-43,019	0.997	0.874	Dox&Pac
rs12904944	0.638	1.028	0.917	1.152	0.493	0.884	0.622	1.257	0.339	15	65,148,828	*SMAD3*	NM_005902.3	intron	-3,076	0.995	0.520	Dox&Pac
rs12956238	0.368	1.085	0.908	1.296	0.981	0.993	0.556	1.775	0.118	18	70,654,268	*ZNF407*	NM_017757.1	intron	-31,822	0.997	0.492	Paclitaxel
rs1349497	0.872	0.979	0.752	1.273	0.830	1.097	0.471	2.557	0.039	2	173,300,909	*RAPGEF4*	NM_007023	upstream	7,933	0.997	0.013	Paclitaxel
rs1400142	0.788	1.016	0.904	1.142	0.905	0.978	0.677	1.413	0.352	12	71,728,762	*TRHDE*	NM_013381.1	flanking_3UTR	-383,073	0.997	0.430	Dox&Pac
rs1468804	0.790	0.984	0.877	1.105	0.693	1.075	0.751	1.540	0.430	7	12,242,033	*TMEM106B*	NM_018374	3UTR	0	0.997	0.117	Dox&Pac
rs1528657	0.160	0.920	0.818	1.034	0.182	1.310	0.881	1.947	0.352	11	13,947,120	*SPON1*	NM_006108.1	intron	-5,855	0.997	0.792	Dox&Pac
rs1532326	0.802	1.020	0.873	1.193	0.575	1.185	0.656	2.141	0.136	12	64,203,455	*MSRB3*	NM_001031679	downstream	56,510	0.993	0.102	Paclitaxel
rs1561092	0.065	0.900	0.804	1.006	0.986	1.003	0.708	1.422	0.477	12	94,728,384	*NTN4*	NM_021229.2	flanking_5UTR	-19,717	0.997	0.810	Paclitaxel
rs1652804	0.800	0.986	0.881	1.102	0.660	1.088	0.748	1.584	0.384	10	70,934,331	*TSPAN15*	NM_012339.3	intron	-84	0.995	0.648	Paclitaxel
rs16838023	0.121	0.898	0.784	1.029	0.507	0.874	0.588	1.300	0.229	2	206,761,661	*GPR1*	NM_005279.2	intron	-11,417	0.997	1.000	Paclitaxel
rs17038123	0.753	0.982	0.877	1.100	0.535	0.880	0.588	1.317	0.450	2	56,482,575	*EFEMP1*	NM_004105.2	flanking_5UTR	-478,893	0.997	0.846	Paclitaxel
rs17047986	0.588	0.969	0.865	1.086	0.585	0.895	0.603	1.331	0.438	2	56,555,161	*VRK2*	NM_006296	upstream	1,572,063	0.997	0.733	Paclitaxel
rs17048108	0.647	0.975	0.874	1.087	0.617	0.897	0.584	1.376	0.470	2	56,620,042	*EFEMP1*	NM_004105.2	flanking_5UTR	-616,360	0.997	0.112	Paclitaxel
rs17069617	0.555	1.048	0.897	1.223	0.587	0.874	0.539	1.419	0.159	5	167,376,943	---	ENST00000388904	intron	0	0.997	0.531	Paclitaxel
rs17079623	0.016	1.234	1.039	1.466	0.215	1.519	0.784	2.940	0.122	18	64,544,220	*TXNDC10*	NM_019022.3	flanking_5UTR	-10,887	0.994	0.145	Dox&Pac
rs17162040	0.596	0.942	0.755	1.175	0.133	1.893	0.824	4.351	0.075	1	27,755,814	*AHDC1*	NM_001029882.1	intron	-1,990	0.997	0.729	Dox&Pac
rs17177982	0.183	1.081	0.964	1.212	0.791	0.953	0.670	1.357	0.348	14	36,505,046	*SLC25A21*	NM_030631	intron	0	0.997	0.526	Dox&Pac
rs17304569	0.019	0.818	0.692	0.967	0.768	0.937	0.607	1.445	0.161	19	24,032,745	*ZNF254*	NM_203282	upstream	29,071	0.994	0.594	Dox&Pac
rs17327860	0.505	1.047	0.915	1.197	0.281	1.290	0.812	2.048	0.219	3	55,437,613	*LRTM1*	NM_020678	upstream	500,501	0.997	0.944	Dox&Pac
rs17404627	0.427	0.943	0.814	1.091	0.858	1.038	0.692	1.555	0.194	8	3,957,010	*CSMD1*	NM_033225.3	intron	-79,981	0.995	0.400	Dox&Pac
rs17473108	0.423	0.934	0.789	1.104	0.178	1.439	0.847	2.444	0.133	4	164,683,228	*March01 gene*	NM_017923.2	intron	-2,970	0.997	0.144	Dox&Pac
rs17527707	0.736	1.021	0.907	1.148	0.198	1.270	0.883	1.827	0.291	6	67,424,871	*LOC442229*	XR_000273.2	flanking_3UTR	-868,778	0.997	0.055	Paclitaxel
rs17576350	0.466	0.938	0.790	1.114	0.360	1.290	0.748	2.226	0.124	4	164,665,964	*FLJ11184*	NM_018352	downstream	4,857	0.997	0.097	Dox&Pac
rs17620029	0.201	0.899	0.763	1.058	0.656	1.100	0.722	1.676	0.155	19	24,146,152	*ZNF539*	NM_203282.1	flanking_3UTR	-41,659	0.996	0.312	Dox&Pac
rs17763056	0.095	0.880	0.757	1.023	0.346	1.232	0.799	1.900	0.168	20	16,807,716	*OTOR*	NM_020157.2	flanking_3UTR	-126,907	0.997	0.608	Dox&Pac
rs1778335	0.947	0.996	0.884	1.123	0.019	1.602	1.081	2.375	0.308	10	22,972,154	*PIP4K2A*	NM_005028	intron	0	0.997	0.043	Paclitaxel
rs1793640	0.883	0.991	0.885	1.111	0.454	0.872	0.609	1.248	0.389	11	131,426,908	*HNT*	NM_016522.2	intron	-94,478	0.996	0.840	Paclitaxel
rs184234	0.430	1.054	0.925	1.202	0.678	0.923	0.633	1.346	0.264	15	99,069,242	*ASB7*	NM_198243.1	flanking_3UTR	-62,068	0.997	0.497	Paclitaxel
rs1885822	0.356	0.948	0.847	1.062	0.760	1.055	0.748	1.488	0.327	10	77,284,010	*C10orf11*	NM_032024.2	intron	-71,224	0.997	0.326	Paclitaxel
rs1939717	0.988	1.001	0.894	1.120	0.165	0.787	0.561	1.103	0.445	11	131,405,946	*HNT*	NM_016522.2	intron	-115,440	0.997	0.356	Paclitaxel
rs196074	0.782	1.033	0.819	1.304	0.636	0.814	0.349	1.903	0.070	22	37,162,458	*KCNJ4*	NM_004981.1	intron	-7,464	0.996	0.266	Paclitaxel
rs1995274	0.839	1.016	0.868	1.190	0.361	0.790	0.476	1.310	0.153	5	61,201,717	*FLJ37543*	NM_173667.1	flanking_3UTR	-163,598	0.995	0.517	Paclitaxel
rs201460	0.933	0.995	0.878	1.127	0.892	1.030	0.671	1.581	0.242	7	101,528,291	*CUTL1*	NM_001913.2	intron	-790	0.995	0.115	Dox&Pac

rs2018361	0.804	1.014	0.907	1.134	0.174	0.776	0.539	1.119	0.398	11	131,407,387	*HNT*	NM_016522.2	intron	-113,999	0.996	0.548	Paclitaxel
rs2036203	0.960	0.997	0.891	1.116	0.226	1.247	0.873	1.781	0.438	4	125,894,505	*ANKRD50*	NM_020337.1	flanking_5UTR	-43,123	0.997	0.329	Paclitaxel
rs2130017	0.741	0.980	0.868	1.105	0.432	0.886	0.656	1.197	0.302	11	89,142,024	*TRIM49*	NM_020358.2	flanking_3UTR	-28,447	0.994	0.123	Dox&Pac
rs2334207	0.504	0.960	0.852	1.082	0.816	1.041	0.740	1.466	0.489	16	86,138,699	*JPH3*	NM_020655.2	flanking_5UTR	-55,301	0.996	0.061	Paclitaxel
rs2430363	0.997	1.000	0.888	1.127	0.722	0.943	0.681	1.304	0.345	14	91,434,804	*FBLN5*	NM_006329.2	intron	-3,635	0.996	0.338	Paclitaxel
rs243037	0.193	0.924	0.820	1.041	0.751	0.933	0.610	1.428	0.330	2	60,452,604	---	ENST0000038656	downstream	677,114	0.997	1.000	Paclitaxel
rs243039	0.240	0.931	0.826	1.049	0.649	0.897	0.561	1.434	0.314	2	60,451,069	*BCL11A*	NM_138559.1	flanking_3UTR	-80,737	0.997	1.000	Paclitaxel
rs243044	0.268	0.935	0.829	1.054	0.649	0.897	0.561	1.434	0.313	2	60,449,297	*BCL11A*	NM_138559.1	flanking_3UTR	-82,509	0.995	1.000	Paclitaxel
rs243050	0.254	0.932	0.825	1.052	0.916	1.026	0.641	1.640	0.297	2	60,445,531	*BCL11A*	NM_138559.1	flanking_3UTR	-86,275	0.985	0.248	Paclitaxel
rs243052	0.225	0.926	0.819	1.048	0.922	0.976	0.599	1.590	0.312	2	60,443,761	*BCL11A*	NM_138559.1	flanking_3UTR	-88,045	0.956	0.569	Paclitaxel
rs2482863	0.617	0.959	0.815	1.129	0.740	1.076	0.697	1.663	0.132	1	62,160,186	*INADL*	NM_176878.1	intron	-5,804	0.997	0.295	Paclitaxel
rs2509920	0.408	0.953	0.850	1.068	0.779	1.061	0.702	1.603	0.425	11	58,093,120	*LPXN*	NM_004811	intron	0	0.997	0.116	Paclitaxel
rs2510866	0.322	0.944	0.841	1.058	0.779	1.061	0.702	1.603	0.424	11	58,012,339	*OR5B12*	NM_001004733	upstream	48,139	0.997	0.169	Paclitaxel
rs2515368	0.343	0.946	0.843	1.061	0.779	1.061	0.702	1.603	0.425	11	58,020,757	*OR5B21*	NM_001005218.1	flanking_3UTR	-10,468	0.997	0.128	Paclitaxel
rs2606094	0.526	0.964	0.862	1.079	0.590	1.091	0.796	1.494	0.339	10	62,994,853	*C10orf107*	NM_173554.1	flanking_5UTR	-97,872	0.997	0.108	Paclitaxel
rs2650717	0.493	0.961	0.859	1.076	0.590	1.091	0.796	1.494	0.341	10	62,978,075	*TMEM26*	NM_178505	upstream	95,230	0.997	0.165	Paclitaxel
rs2650741	0.512	0.963	0.860	1.078	0.590	1.091	0.796	1.494	0.339	10	63,002,133	*C10orf107*	NM_173554.1	flanking_5UTR	-90,592	0.993	0.097	Paclitaxel
rs2662411	0.723	0.980	0.878	1.094	0.039	0.666	0.453	0.980	0.417	5	10,186,704	---	ENST0000036292	downstream	372,586	0.996	0.805	Paclitaxel
rs2700868	0.028	1.193	1.019	1.397	0.622	1.114	0.726	1.710	0.157	3	183,922,829	*ATP11B*	NM_014616	upstream	71,156	0.997	0.716	Paclitaxel
rs2814027	0.611	1.030	0.918	1.156	0.389	1.154	0.832	1.601	0.430	10	63,012,118	*C10orf107*	NM_173554.1	flanking_5UTR	-80,607	0.995	0.186	Paclitaxel
rs2830987	0.427	0.951	0.841	1.076	0.321	0.805	0.523	1.237	0.335	21	27,798,286	---	ENST0000032498	upstream	509,286	0.997	0.957	Dox&Pac
rs2830988	0.380	0.946	0.837	1.070	0.321	0.805	0.523	1.237	0.334	21	27,799,163	---	ENST0000032498	upstream	508,409	0.997	0.914	Dox&Pac
rs2830991	0.427	0.951	0.842	1.076	0.321	0.805	0.523	1.237	0.336	21	27,808,439	---	ENST0000032498	upstream	499,133	0.997	0.830	Dox&Pac
rs2830992	0.449	0.954	0.843	1.078	0.321	0.805	0.523	1.237	0.335	21	27,810,551	*C21orf94*	NM_145180.2	flanking_5UTR	-497,021	0.995	0.872	Dox&Pac
rs313997	0.973	0.998	0.892	1.117	0.226	1.247	0.873	1.781	0.438	4	125,902,380	*ANKRD50*	NM_020337.1	flanking_5UTR	-50,998	0.997	0.329	Paclitaxel
rs338466	0.710	1.021	0.914	1.142	0.105	0.760	0.546	1.059	0.472	1	109,287,719	*CLCC1*	NM_015127.2	coding	[99/41]	0.997	0.700	Paclitaxel
rs3733870	0.657	0.972	0.858	1.102	0.571	0.886	0.583	1.347	0.256	5	159,411,119	*TTC1*	NM_003314.1	intron	-351	0.996	0.146	Dox&Pac
rs3750263	0.538	0.952	0.815	1.113	0.805	1.066	0.642	1.768	0.156	8	29,036,982	*KIF13B*	NM_015254.2	intron	-54	0.997	1.000	Dox&Pac
rs3774921	0.877	0.991	0.882	1.114	0.245	0.800	0.549	1.166	0.474	4	23,419,745	*PPARGC1A*	NM_013261.2	intron	-3,723	0.996	0.700	Paclitaxel
rs3823612	0.846	0.989	0.881	1.110	0.693	1.075	0.751	1.540	0.429	7	12,225,280	*TMEM106B*	NM_018374	intron	0	0.997	0.129	Dox&Pac
rs3913369	0.827	1.015	0.886	1.164	0.281	1.290	0.812	2.048	0.213	3	55,456,115	*WNT5A*	NM_003392.3	flanking_3UTR	-18,668	0.990	1.000	Dox&Pac
rs4319365	0.930	1.005	0.895	1.129	0.874	1.028	0.733	1.442	0.445	1	56,680,308	*PPAP2B*	NM_003713.3	flanking_3UTR	-52,713	0.996	0.496	Dox&Pac
rs4614971	0.611	0.971	0.867	1.087	0.703	0.926	0.623	1.376	0.433	2	56,541,133	*EFEMP1*	NM_004105.2	flanking_5UTR	-537,451	0.995	0.732	Paclitaxel
rs4787483	0.193	1.082	0.961	1.218	0.391	1.166	0.821	1.656	0.328	16	29,792,948	*SEZ6L2*	NM_012410.1	intron	-397	0.997	0.550	Paclitaxel
rs4893555	0.539	1.031	0.936	1.136	0.849	0.971	0.719	1.312	0.366	X	28,782,000	*IL1RAPL1*	NM_014271	intron	0	0.997	0.816	Dox&Pac
rs4893599	0.460	1.037	0.942	1.142	0.988	0.998	0.738	1.349	0.377	X	28,782,529	*IL1RAPL1*	ENST0000030005	upstream	10,101	0.997	0.540	Dox&Pac
rs4917139	0.261	0.933	0.828	1.053	0.189	0.801	0.575	1.115	0.369	7	48,232,695	*ABCA13*	NM_152701.2	intron	-1,247	0.997	0.797	Dox&Pac
rs548726	0.632	0.970	0.856	1.099	0.079	1.481	0.956	2.295	0.270	1	5,836,208	*NPHP4*	NM_015102.2	flanking_3UTR	-9,249	0.996	0.077	Dox&Pac
rs580204	0.106	1.097	0.980	1.227	0.809	0.961	0.696	1.327	0.412	22	19,321,062	*PCQAP*	NM_001003891.1	flanking_3UTR	-49,143	0.996	0.552	Paclitaxel
rs6101283	0.966	1.004	0.841	1.199	0.406	1.252	0.737	2.125	0.114	20	59,267,891	*CDH4*	NM_001794	intron	0	0.996	0.635	Dox&Pac
rs6101284	0.947	1.006	0.843	1.201	0.406	1.252	0.737	2.125	0.113	20	59,269,784	*CDH4*	NM_001794.2	intron	-6,396	0.997	0.719	Dox&Pac
rs612051	0.921	0.990	0.812	1.207	0.529	0.830	0.465	1.482	0.088	3	15,050,364	*NR2C2*	NM_003298	intron	0	0.997	0.291	Paclitaxel
rs6460895	0.925	0.995	0.887	1.115	0.693	1.075	0.751	1.540	0.430	7	12,219,065	*TMEM106B*	NM_018374	upstream	1,888	0.997	0.117	Dox&Pac
rs6569759	0.686	0.978	0.876	1.091	0.484	1.134	0.797	1.614	0.484	6	132,174,809	*ENPP1*	NM_006208.1	intron	-3,701	0.997	0.665	Dox&Pac
rs6583476	0.399	0.950	0.842	1.071	0.189	0.801	0.575	1.115	0.366	7	48,214,960	*ABCA13*	NM_152701.2	intron	-6,448	0.997	0.796	Dox&Pac
rs6589877	0.435	0.928	0.769	1.120	0.154	0.684	0.406	1.153	0.113	11	120,701,161	*SC5DL*	NM_006918.3	flanking_3UTR	-16,582	0.997	0.812	Dox&Pac
rs6598542	0.207	1.079	0.959	1.214	0.063	0.719	0.507	1.018	0.350	15	97,110,579	*IGF1R*	NM_000875	intron	0	0.997	0.833	Dox&Pac
rs661440	0.085	1.109	0.986	1.246	0.823	0.958	0.661	1.390	0.347	22	19,320,057	*PCQAP*	NM_001003891.1	flanking_3UTR	-48,138	0.996	0.204	Paclitaxel
rs6662529	0.371	0.947	0.840	1.067	0.921	1.018	0.715	1.448	0.331	1	209,289,328	*KCNH1*	NM_002238.2	intron	-30,107	0.997	0.159	Paclitaxel
rs667520	0.303	0.938	0.830	1.059	0.706	0.937	0.667	1.316	0.402	6	138,057,818	*OLIG3*	ENST0000036913	downstream	1,464,907	0.997	0.689	Paclitaxel
rs6694114	0.807	0.985	0.876	1.109	0.534	1.106	0.805	1.520	0.350	1	56,682,138	*PPAP2B*	NM_003713.3	flanking_3UTR	-50,883	0.992	0.634	Dox&Pac
rs6708612	0.944	1.015	0.674	1.528	0.795	1.303	0.177	9.598	0.020	2	147,882,193	---	ENST0000038449	upstream	187	0.997	1.000	Paclitaxel
rs6746117	0.944	1.015	0.674	1.528	0.795	1.303	0.177	9.598	0.020	2	147,871,631	*ACVR2A*	NM_001616.3	flanking_5UTR	-447,409	0.997	1.000	Paclitaxel
rs6794522	0.210	0.910	0.785	1.055	0.553	0.876	0.564	1.359	0.180	3	115,120,114	*GRAMD1C*	NM_017577.2	intron	-2,739	0.995	0.222	Paclitaxel
rs6816145	0.626	0.954	0.788	1.154	0.136	1.533	0.874	2.689	0.100	4	164,638,444	*FLJ11184*	NM_018352.1	intron	-3,038	0.993	0.083	Dox&Pac
rs6843038	0.510	1.041	0.924	1.172	0.071	1.583	0.962	2.606	0.313	4	182,162,059	*MGC45800*	NM_178838.3	flanking_3UTR	-1,137,738	0.997	0.956	Dox&Pac
rs6914925	0.659	1.028	0.910	1.161	0.552	0.904	0.647	1.262	0.366	6	149,411,678	*UST*	NM_005715.1	intron	-24,984	0.997	0.379	Paclitaxel
rs6967385	0.915	1.006	0.898	1.127	0.296	0.811	0.548	1.201	0.416	7	12,357,844	*TMEM106B*	NM_018374.2	flanking_3UTR	-114,433	0.996	0.882	Paclitaxel
rs7008176	0.958	0.997	0.888	1.119	0.835	1.043	0.701	1.554	0.335	8	61,994,553	*CHD7*	NM_017780.2	flanking_3UTR	-52,534	0.997	0.451	Paclitaxel
rs7028484	0.656	0.970	0.850	1.108	0.971	1.007	0.689	1.471	0.234	9	23,536,461	*ELAVL2*	NM_004432.1	flanking_3UTR	-145,523	0.997	0.638	Paclitaxel
rs7260598	0.020	0.821	0.694	0.970	0.768	0.937	0.607	1.445	0.162	19	24,014,626	*ZNF254*	NM_203282	upstream	47,190	0.996	0.428	Dox&Pac
rs745382	0.368	1.054	0.940	1.183	0.309	1.237	0.822	1.862	0.414	11	128,967,443	*BARX2*	NM_003658.3	flanking_3UTR	-140,418	0.997	0.520	Paclitaxel
rs7519667	0.753	1.025	0.881	1.191	0.028	0.543	0.315	0.935	0.178	1	239,951,930	*WDR64*	NM_144625.2	intron	-1,292	0.997	0.327	Paclitaxel
rs7582078	0.268	1.067	0.951	1.198	0.852	1.031	0.747	1.424	0.457	2	208,355,096	*FZD5*	NM_003468.2	flanking_5UTR	-12,733	0.997	0.498	Dox&Pac
rs7625117	0.965	0.996	0.820	1.208	0.452	0.796	0.439	1.444	0.088	3	15,137,581	*ZFYVE20*	NM_022340.2	flanking_5UTR	-21,922	0.997	0.174	Paclitaxel
rs7630682	0.629	1.051	0.859	1.285	0.520	1.221	0.665	2.242	0.081	3	76,824,325	*ROBO2*	NM_002942	upstream	348,302	0.996	0.074	Paclitaxel
rs7653262	0.725	0.979	0.872	1.100	0.685	1.090	0.718	1.656	0.351	3	30,418,873	*TGFBR2*	NM_003242.4	flanking_5UTR	-204,125	0.997	0.528	Paclitaxel
rs766045	0.569	0.956	0.821	1.115	0.965	0.990	0.635	1.543	0.175	8	3,968,386	*CSMD1*	NM_033225.3	intron	-91,357	0.995	0.561	Dox&Pac
rs7705332	0.926	1.006	0.892	1.133	0.342	1.183	0.836	1.674	0.320	5	128,623,393	*ISOC1*	NM_016048.1	flanking_3UTR	-145,663	0.997	0.783	Paclitaxel
rs7724279	0.428	1.052	0.929	1.190	0.750	1.052	0.772	1.433	0.314	5	31,993,642	*PDZK3*	NM_015022.2	intron	-25,376	0.997	0.781	Dox&Pac
rs7789880	0.094	1.108	0.983	1.250	0.062	1.367	0.985	1.898	0.276	7	27,883,619	*JAZF1*	NM_175061	intron	0	0.997	0.589	Paclitaxel
rs7812453	0.074	0.902	0.806	1.010	0.983	0.997	0.729	1.362	0.499	8	72,917,594	*MSC*	NM_005098.2	intron	-58	0.996	0.001	Paclitaxel
rs7857311	0.345	0.907	0.741	1.111	0.900	1.044	0.533	2.045	0.086	9	22,955,874	*ELAVL2*	ENST0000038013	downstream	513,402	0.996	0.539	Paclitaxel

rs7931234	0.714	1.022	0.909	1.150	0.065	1.321	0.982	1.776	0.391	11	58,771,325	---	ENST00000387068	upstream	32,577	0.997	0.087	Paclitaxel
rs7995709	0.076	1.128	0.988	1.289	0.527	0.877	0.583	1.318	0.210	13	105,810,213	---	ENST00000378993	intron	0	0.996	0.279	Paclitaxel
rs8021868	0.672	1.061	0.807	1.395	0.770	1.138	0.479	2.704	0.039	14	56,861,066	*NAT12*	NM_001011713	upstream	65,958	0.997	0.040	Dox&Pac
rs891562	0.112	0.887	0.765	1.028	0.130	1.386	0.908	2.115	0.186	10	70,944,743	*TSPAN15*	NM_012339.3	flanking_3UTR	-7,314	0.996	0.177	Paclitaxel
rs9510315	0.785	1.020	0.886	1.173	0.844	1.048	0.658	1.669	0.192	13	22,218,655	*FTHL7*	NR_002202.1	flanking_3UTR	-49,745	0.997	0.438	Paclitaxel
rs980038	0.063	1.124	0.994	1.271	0.376	1.195	0.805	1.774	0.260	4	31,279,013	*PCDH7*	NM_032457.1	flanking_3UTR	-525,162	0.997	0.319	Paclitaxel
rs986787	0.670	0.976	0.871	1.093	0.585	0.895	0.603	1.331	0.437	2	56,529,867	*EFEMP1*	NM_004105.2	flanking_5UTR	-526,185	0.997	0.696	Paclitaxel
rs9868927	0.878	1.009	0.902	1.129	0.311	1.190	0.850	1.668	0.439	3	174,822,471	*NLGN1*	NM_014932.2	intron	-16,896	0.997	0.495	Dox&Pac
rs9883082	0.277	0.937	0.833	1.054	0.415	0.857	0.591	1.242	0.371	3	7,858,963	*GRM7*	NM_000844.2	flanking_3UTR	-100,746	0.997	0.471	Dox&Pac
rs9961704	0.421	1.051	0.931	1.185	0.646	1.090	0.755	1.573	0.303	18	46,072,686	*CXXC1*	NM_014593.2	flanking_5UTR	-4,500	0.995	0.306	Paclitaxel
rs9998109	0.797	1.018	0.890	1.164	0.475	0.845	0.534	1.340	0.203	4	118,464,432	*TRAM1L1*	NM_152402.1	flanking_5UTR	-238,252	0.997	0.334	Dox&Pac
